# Antioxidant enzymes that target hydrogen peroxide are conserved across the animal kingdom, from sponges to mammals

**DOI:** 10.1038/s41598-023-29304-6

**Published:** 2023-02-13

**Authors:** Olivia H. Hewitt, Sandie M. Degnan

**Affiliations:** School of Biological Sciences and Centre for Marine Science, University of Queensland, St Lucia, QLD 4072 Australia

**Keywords:** Evolution, Evolutionary genetics, Phylogenetics, Comparative genomics

## Abstract

Oxygen is the sustenance of aerobic life and yet is highly toxic. In early life, antioxidants functioned solely to defend against toxic effects of reactive oxygen species (ROS). Later, as aerobic metabolisms evolved, ROS became essential for signalling. Thus, antioxidants are multifunctional and must detoxify, but also permit ROS signalling for vital cellular processes. Here we conduct metazoan-wide genomic assessments of three enzymatic antioxidant families that target the predominant ROS signaller, hydrogen peroxide: namely, monofunctional catalases (CAT), peroxiredoxins (PRX), and glutathione peroxidases (GPX). We reveal that the two most evolutionary ancient families, CAT and PRX, exhibit metazoan-wide conservation. In the basal animal lineage, sponges (phylum Porifera), we find all three antioxidant families, but with GPX least abundant. Poriferan CATs are distinct from bilaterian CATs, but the evolutionary divergence is small. Amongst PRXs, subfamily PRX6 is the most conserved, whilst subfamily AhpC-PRX1 is the largest; PRX4 is the only core member conserved from sponges to mammals and may represent the ancestral animal AhpC-PRX1. Conversely, for GPX, the most recent family to arise, only the cysteine-dependent subfamily GPX7 is conserved across metazoans, and common across Porifera. Our analyses illustrate that the fundamental functions of antioxidants have resulted in gene conservation throughout the animal kingdom.

## Introduction

A universal and ancient challenge for all life forms is to deal with the toxifying effects of molecular oxygen^1^. Reactive derivatives of oxygen, termed reactive oxygen species (ROS), are toxic for non-target molecules^2^, causing oxidative damage to nucleic acids, proteins and lipids^3^, reviewed by^4,5^. To prevent this damage, organisms from all domains of life have antioxidants that function to detoxify and regulate ROS in reduction reactions^6^. The first antioxidants are estimated to have arisen between 3.5 and 4.1 billion years ago (bya), soon after the origin of life on earth, and far predating aerobic metabolism and the rise of atmospheric oxygen^6,7,8,9^. For the anaerobic life forms that inhabited the anoxic and sulfidic atmosphere of early Earth, antioxidants provided an evolutionary advantage by protecting against localised or trace oxygen levels^1,9,10^. By 2.4 bya, in occurrence with the Great Oxidation event, ROS had been recruited into early redox signalling systems^1,11,12,13^, and thus the roles of antioxidants had expanded to include regulators of redox signalling. The most ancient of these antioxidants still exist in extant organisms across all domains, as evidence of shared evolutionary history and their necessity for the survival of aerobic life^6,13,14^.

In extant aerobic organisms, redox signalling together with its regulation by antioxidants is critical to a vast array of life-sustaining cellular functions^15^. These functions include, but are not limited to, innate immunity^16^, cell cycle transition^17,18^, neurodevelopment and regeneration^19,20^, cell differentiation/proliferation^2^, and the circadian redox clock^21^. Antioxidants can be either non-enzymatic (e.g., vitamins E, C, A, selenium, transferrin, and lactoferrin, ascorbic acid, glutathione, melatonin, carotenoids, flavonoids, proline), or specialised enzymes that vary in subcellular localisation, predominant substrate, and rate of reactivity^7,22,23^. The specialist enzymatic antioxidant families include those that target hydrogen peroxide (H_2_O_2_), which is the major redox signalling ROS reviewed by^5,24,25^, and those that target superoxide anion radical (O_2_^•-^) for the generation of H_2_O_2_ as a product[reviewed by^26,27^.

**Figure 1:**
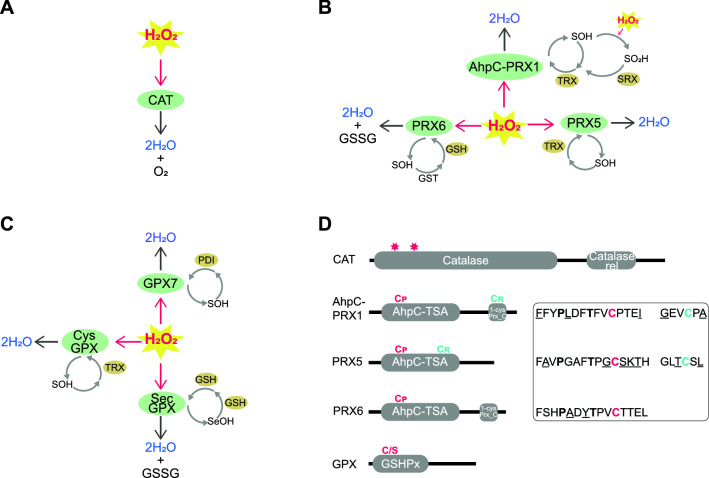
Summary of reaction mechanisms for (**A**): CAT, (**B**): PRX, and (**C**): GPX**.** In the first step of the reaction mechanism of all PRXs and CysGPXs, and GPX7, H_2_O_2_ reacts with the peroxidatic cysteine (C_P_) to form a sulfenic acid (SOH) intermediate. Whilst in SecGPXs, a catalytic selenocysteine first reacts to form a selenic acid (SeOH). If a second, resolving cysteine (C_R_) is present (i.e., in AhpC-PRX1, PRX5 & CysGPX), this quickly reacts with the SOH to form either an inter- or intramolecular disulfide bond that is then most commonly reduced by thioredoxin (TRX), reactivating the enzyme. PRX6 instead forms a disulfide with another molecule, commonly GST, and is then recycled by glutathione (GSH), generating oxidised glutathione (GSSG). In SecGPXs the SeOH is similarly reduced by two GSH generating GSSG, whilst GPX7 is described to be reactivated via the ER protein disulfide isomerase (PDI). For PRXs under high concentrations of H_2_O_2_, SOH reacts with another molecule of H_2_O_2_ to form a sulfenic acid (SO_2_H), resulting in hyperoxidation. Enzymes within the subfamily, AhpC-PRX1 only may then be slowly re-activated via the enzyme sulfiredoxin (SRX) via the inactivation loop. (**D**): Generalised domain structure for CAT, PRX, and GPX enzyme families. CAT enzymes comprise a catalase domain and catalase-related immune -responsive (catalase_rel). Stars denote presence of active site, His and Asn respectively. All PRX enzymes comprise the domain, Alkyl hydroperoxide reductase-Thiol specific antioxidant (AphC-TSA). The subfamilies AhpC-PRX1 and PRX6 additionally commonly encode the Peroxiredoxin, C-terminal domain (1-cysPrx_C). C_P_ (red) and C_R_ (blue) conserved active site are displayed, residues in bold denote absolutely conserved, and underlined residues denotes amino acids that deviated from that displayed within more than one metazoan sequence. GPX enzymes comprise a single GSHPx domain. GPX enzymes may encode either C_P_ or a catalytic Sec (S) within the active site.

The three major families of antioxidants that target H_2_O_2_ are monofunctional catalase (CAT), peroxiredoxin (PRX), and glutathione peroxidase (GPX) (Fig. 1A–C)^28,29,30,31^. Of these, CAT that uses iron as its electron acceptor, and PRX that uses sulfur-based cysteine^32^, both represent some of the most evolutionary ancient antioxidant enzymes targeting H_2_O_2_; both predate the great oxidation event (GOE)^7^. The third family, GPX that uses glutathione (GSH) as a reductant, has a more recent evolutionary origin, after the GOE^7^. Both the PRX and GPX enzyme families have been further classified into multiple subfamilies, but because these subfamily classifications have been based largely on mammalian gene complements, their relevance to the rest of the animal kingdom is not clear.

In this study, we conduct a comparative genomic assessment of these three major antioxidant enzyme families—CAT, PRX, and GPX—in 19 species, with high-quality genomes that span 10 metazoan phyla, from sponges to chordates. In doing so, we provide the first assessment of these enzymes in sponges (phylum Porifera), including four marine and one freshwater species of three different classes. Sponges evolved at least 700 million years ago^33^ and are widely considered to be the oldest of the extant animal phyletic lineages^34,35^. As probable sister to all other animal phyla, traits shared by sponges and the rest of animal kingdom can logically be traced back to the last common animal ancestor^36^. Thus, sponges can provide unique insight into the evolutionary history of these ancient enzymatic antioxidant families that play a critical role throughout the animal kingdom.

## Results and discussion

**Figure 2:**
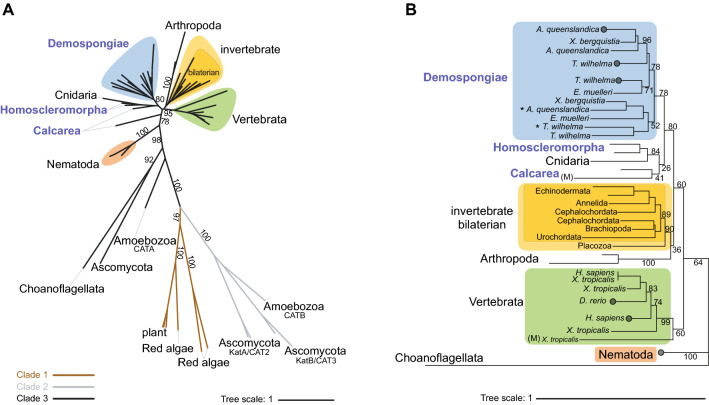
Maximum likelihood phylogenetic tree of monofunctional CAT enzyme family. (**A**): Unrooted tree displaying three main CAT clades indicated by branch colour: clade 1 (dark grey), clade 2 (light grey), clade 3 (black). Within clade 3 coloured shapes indicate identified evolutionary groups; Nematoda (orange), Demospongiae (blue), Invertebrate (yellow), inner dashed line indicates bilaterian-invertebrate species only, and Vertebrata (green). (**B**): Rooted phylogenetic tree using metazoan CAT protein sequences and the choanoflagellate *M. brevicollis* CAT as an outgroup. (M) denotes mitochondrial localised CAT sequences. Asterisk (*) next to A*. queenslandic*a and *T. wilhelma* denotes full length CAT gene sequences for these two species. Labels coloured blue denote sequences encoded by phylum Porifera. Black numbers on branches indicate bootstrap support. Circles denote collapse tree nodes. Coloured shapes in B correspond to those displayed in (**A**). Branch lengths represent evolutionary distances, indicated by tree scale. Constructed based on edited alignment, 1000 bb and the evolutionary models, A: LG + I + G4 and B: LG + G4.

Our metazoan-wide survey has provided the most comprehensive analysis to date of gene number and phylogenetic distribution of three key antioxidant gene families across the animal kingdom. Genes encoding all three families were observed in 18 metazoan species; the exception is the ctenophore *Mnemiopsis leidyi* that has PRX and GPX, but not CAT (Table 1). Our findings demonstrate that the antioxidants CAT and PRX both are evolutionary ancient and highly conserved enzyme families (Figs. 2 and 4). By comparison, the GPX family is less conserved, with total gene numbers and functional types varying considerably among metazoan species (Table 1). Below we discuss our expanded analysis, detailing substantial gene conservation across evolutionary diverse bilaterian and non-bilaterian phyletic lineages, and for the first time reporting a suite of H_2_O_2_-targeting enzymatic antioxidants in the basal metazoan phylum Porifera (the sponges).

**Table 1:** Total counts of antioxidant enzymes identified from genome sequences of 19 metazoan species. CAT includes monofunctional catalases only. PRXs separated by subfamily, AhpC-PRX1 (typical 2-Cys PRX), PRX5 (atypical 2-cys PRXs), PRX6 (1-Cys PRX). GPX includes subfamilies GPX 1- 8, with gene sequences obtained from the six species assessed here, as well as from 13 species obtained previously by^37^ and those assessed. Large numbers indicate total number of unique sequences identified, including isoforms, splice variants. Superscript numbers indicate number of additional identical protein sequences (exact sequence variants).

Species	Phylum	CAT	AhpC-PRX1	PRX5	PRX6	GPX
Amphimedon queenslandica	Porifera	4	1	1	1	*2*
Xestospongia bergquistia	Porifera	2	1	2	1	1
Tethya wilhelma	Porifera	6	2	1	2	3
Ephydatia muelleri	Porifera	2	2	2^2^	2^2^	2
Oscarella carmela	Porifera	2	2	1	2	2
Sycon ciliatum	Porifera	2	3	1	**1**	1
Mnemiopsis leidyi	Ctenophora	–	3	**–**	1	*2*
Nematostella vectensis	Cnidaria	1	2	1	1	*5*
Capitella teleta	Annelida	1	3	3	1	3
Lingula anatina	Brachipoda	1	4	1	2	*5*
Drosophila melanogaster	Arthropoda	2	3	1	4	*2*
Caenorhabditis elegans	Nematoda	4^2^	4	**–**	1	*7*
Strongylocentrotus purpuratus	Echinodermata	1	2	**–**	1	*4*
Acanthaster planci	Echinodermata	1	3	**–**	1	*4*
Branchiostoma floridae	Chordata	2	3	2	3	*7*
Ciona intestinalis	Chordata	1	3	**–**	2	5
Danio rerio	Chordata	2^1^	4	1	1	*8*
Xenopus tropicalis	Chordata	4	4	1	1	*6*
Homo sapiens	Chordata	2^1^	8^2^	2	1	*8*

### Monofunctional catalase (CAT)

The monofunctional (i.e., “typical”) CAT enzyme family are one of the most evolutionary ancient antioxidants targeting hydrogen peroxide^7^; the other is the PRX family. The metazoan CATs comprise a relatively small family; most metazoans that we assessed encode just one full length CAT sequence, and the evolutionary divergence among them is relatively small compared with the PRX and GPX enzyme families (Table 1 and Fig. 2).

HMM scans, based on hidden Markov probabilistic models, across coding sequences from 19 metazoan species revealed a total of 56 unique protein sequences encoding at least one CAT-associated domain. Filtering these after sequence alignment and protein structure reduced this number to 44 (Fig. 1D; Supplementary file 2). On this basis, we identified CAT protein sequences in 18 of the 19 metazoans; the exception was the ctenophore *M. leidyi,* that likely represents evidence of gene loss, given the ancient origins of the CAT family.

CAT enzymes can broadly be assigned to one of three main clades, comprising either small subunit sizes (55–69 kDa) with heme b as the prosthetic group (Clade 3, or clade 1), or large subunit sizes (75–84 kDa) with heme d as the prosthetic group and an additional ‘flavodoxin like’ domain (Clade 2)^14,29,38^. Of these, Clade 3 enzymes that use NADPH as cofactor are the most widely distributed—all 44 of the animal CAT enzymes we identified belong to Clade 3 and are distinct from the 15 non-metazoan sequences (Fig. 2A). Notably, there are relatively short evolutionary distances among genes within clade 3 compared to genes within the non-metazoan clades 1 and 2. This reflects the relatively recent diversification of metazoan CATs within the much older evolutionary history of this enzyme family (Fig. 2A).

### Porifera and cnidaria CAT are phylogenetically distinct from other metazoans

Phylogenetic assessment of 44 animal CATs reveals three well-supported clades. These are Vertebrata (Fig. 2B: 99%), a predominantly bilaterian-invertebrate (Fig. 2B; 90%), and a Poriferan/Cnidarian clade (Fig. 2B; 80%). Our findings are consistent with^14,38^, but our expanded analysis provides additional evolutionary insight at the base of the metazoan CAT tree. We show that CATs in the basal metazoan phyla Cnidaria and Porifera are evolutionarily distinct from the rest of the metazoan CATs, including those of the phylum Placozoa that sit within an otherwise bilaterian invertebrate clade (Fig. 2B). Moreover, this Poriferan/Cnidarian clade includes two strongly supported subclades of Demospongiae (78%) and Homoscleromorpha/Cnidaria (84%), indicating diversification of these genes before sponges diverged from the metazoan stem*.* The exception to this is *S. ciliatum* (class Calcarea) CATs that display greater divergence and low support within Poriferan/Cnidarian clade (Fig. 2b. 26%).

Also consistent with Zámocký et al.^14,38^, we find that CATs of *Caenorhabditis elegans* (phylum Nematoda) and *Drosophila melanogaster* (Arthropoda) are both evolutionarily separated from the rest of the Bilateria, each forming independent monophyletic clades (Fig. 2A,B 100%). Of these, the nematode clade displays the greatest evolutionary divergence, and sits as sister to all other metazoans (Fig. 2B). Notably, observed evolutionary distances within CAT clade 3 are comparatively shorter than within the non-metazoan clades 1 and 2 (Fig. 2A). Considering this and the evolutionary divergence of Nematoda, we hypothesise that diversification of CAT within metazoans is relatively recent compared to the long evolutionarily history of this enzyme.

**Figure 3:**
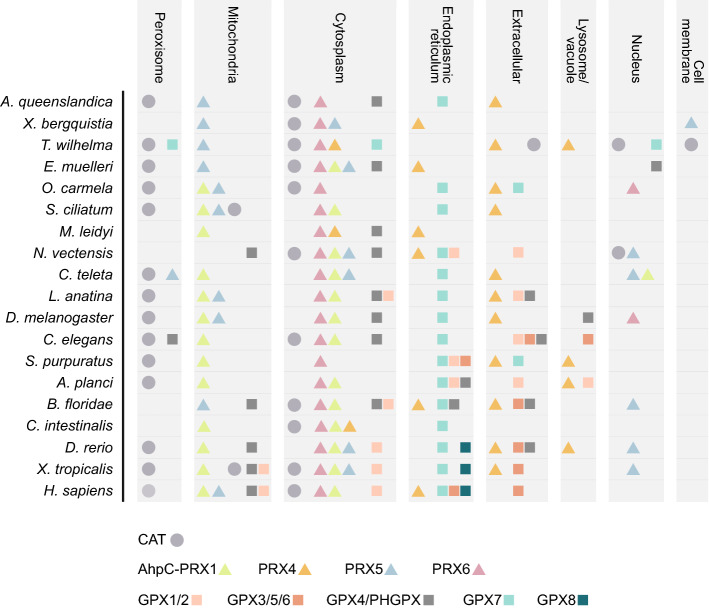
Presence of antioxidant enzymes within 8 different subcellular compartments of 19 metazoan species. Shapes denote enzyme family, namely CAT (circle), PRX (triangle), or GPX (square). Subcellular compartments indicated are predictions based on amino acid sequence analysis by DeepLoc-2.0, https://services.healthtech.dtu.dk/service.php?DeepLoc-2.0^39,40^. Colours denote individual PRX and GPX enzyme subfamilies, based on phylogeny corresponding to Figs. 4 and 5. Total number of CAT, PRX, and GPX gene sequences encoded by each species in Table 1.

Consistent with previous descriptions, we found that metazoan CATs from 14 species are predicted to localise to the peroxisome (20 sequences) (Fig. 3; Supplementary file 3)^29^. However, 17 species have CAT enzymes that localise to multiple subcellular compartments. Overall, we most commonly predicted CATs sequences that localise to the cytoplasm (24 sequences), but also the mitochondria (2 sequences), nucleus, cell membrane and extracellular space (Fig. 3; Supplementary file 3). Moreover, we identified four species, (*Xestospongia bergquistia*, *Nematostella vectensis*, *Ciona intestinalis*, and *Branchiostoma floridae*) that do not have any peroxisomal CAT but instead encode a cytoplasmic CAT (Fig. 3). That said, 9 of the 24 cytoplasm-localised sequences do encode a peroxisomal targeting signal, whilst 10 sequences encode a nuclear targeting signal (Supplementary file 3). The phylogenetic distribution of cytoplasmic- or peroxisomal-localised CATs has no obvious pattern. However, the two mitochondrial-localised sequences, found in *Xenopus tropicalis* (Vertebrata) and *Sycon ciliatum* (Calcarea), are each the most divergent within their respective clades (indicated on Fig. 2b). It has been hypothesised that having multiple CATs localised to various subcellular compartments may confer additional benefits against diseases such as cancer^29^. However, we cannot assume that all CAT enzymes localised to various subcellular regions are functionally active. For instance, the sponges *Amphimedon queenslandica* and *Tethya wilhelma* (class Demospongiae) each encode only one full length CAT sequence localised within the cytoplasm, indicated by asterisks (Fig. 2B), and their additional CAT sequences are of reduced length and thus perhaps non-functional.

**Figure 4:**
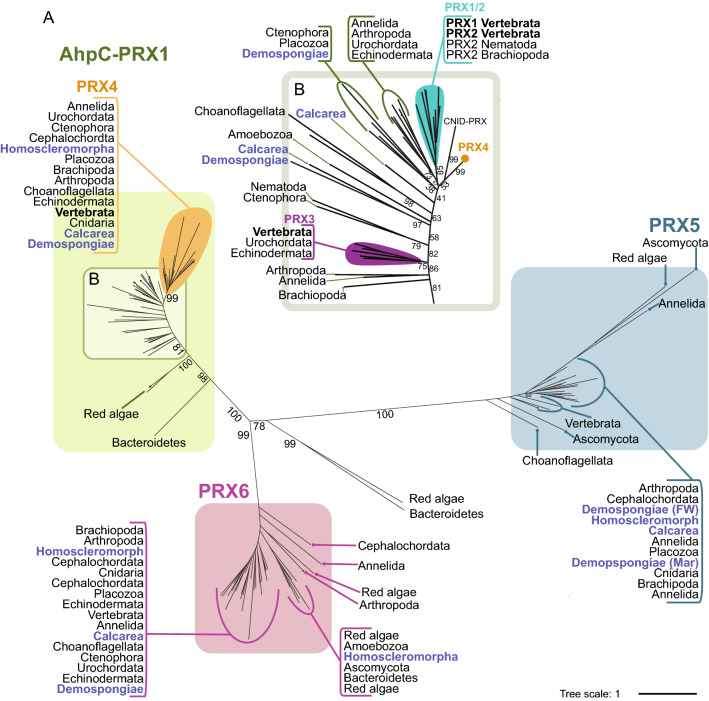
(**A**): Maximum likelihood phylogenetic tree of PRX enzyme family. Unrooted tree displaying PRX animal subfamilies; AhpC-PRX1 (green), PRX5 (blue), and PRX6 (pink) that correspond with the three broad classes, typical 2-Cys PRX, atypical 2-Cys PRX, and 1-Cys PRX, respectively. Within AhpC-PRX1 orange shape denotes strongly supported monophyletic clade named PRX4. (**B**): Depicts zoomed in region of AhpC-PRX1 clade. Monophyletic clades comprising isoforms, PRX1/2 (turquoise) and PRX3 (purple) of Vertebrata are indicated. These isoforms were previously used to classify PRXs across all metazoan species until a revision of PRX system of classification^41,42^. CNID-PRX denotes recently established subfamily found only within species belonging to phylum Cnidaria^43^. Orange circle indicates collapsed nodes of PRX4 clade. Labels coloured blue denote sequences encoded by phylum Porifera. Black numbers on branches indicate bootstrap support. Branch lengths represent evolutionary distances, indicated by tree scale. Constructed based on edited alignment, 1000 bb and the WAG + I + G4 evolutionary model. FW: fresh water, SW: sea water.

### Peroxiredoxins (PRX)

The PRXs are a large yet highly conserved enzyme family amongst metazoans. Across all 19 metazoan species, we identified a total of 799 unique protein sequences encoding at least one PRX-associated domain. Filtering these by presence of the strictly conserved C_P_ motif (**P**XXX(**T**/**S**)XX**C**) required for catalytic activity reduced this number to 110, uncovering multiple subfamilies encoded by all 19 metazoan species (Table 1; Supplementary file 2). Our phylogenetic analysis is largely consistent with previous assessments based on smaller numbers of taxa PRX^44,43^, revealing high support (99–100%) for three monophyletic clades corresponding to the animal subfamilies, AhpC-PRX1, PRX5, and PRX6 (Fig. 4A). However, our taxonomic expansion highlights PRX diversity and supports use of the most recent system of PRX classification based on the peroxidatic cysteine (C_P_) active site sequence^41,42^.

### AhpC-PRX1 is the largest metazoan PRX subfamily

The PRXs comprise three animal subfamilies, of which AhpC-PRX1 is the largest. For 12 of the 19 metazoan species, we find at least three AhpC-PRX1 genes each, compared to just one or two genes in subfamilies PRX5 and PRX6 (Table 1).

In a previous system of classification that used homology to mammalian PRX isoforms, subfamily AhpC-PRX was subdivided into isoforms PRX1-4^41,45^. However, this system was later deemed insufficient to accurately describe PRXs across diverse animal species^45^. Indeed, here we show only small subclades of sequences that share similarity to the mammalian isoforms PRX1/2 (turquoise) and PRX3 (purple) (85% & 75%; Fig. 4B). Instead, AhpC-PRX1 comprises multiple independent branches such as the recently described CNID-PRX that is a lineage specific divergence within phylum Cnidaria^43^ (Fig. 4B). That said, sequences sharing similarity to mammalian isoform PRX4 (orange) do form a strongly supported subclade that is widespread across the animal kingdom, being absent only in *C. elegans* (99%; Fig. 4A). Indeed, the only AhpC-PRX1 we find in the three marine species of demosponge are these PRX4-like sequences. Additionally, the non-metazoan, *M. breviocolis* choanoflagellate encodes a single sequence that falls within the PRX4 subclade, indicating that PRX4 may predate the origin of metazoans. Subsequently we propose that PRX4 may be the closest animal orthologue of the ancestral AhpC-PRX1.

Here we use the most recently proposed PRX classification system, that identifies six subfamilies, of which three occur in animals, based on protein sequence similarities at the peroxidatic cysteine (C_P_) active site^41,42^ (Table S3). Consistent with this classification, we report highly conserved **P**XXX(**T**/**S**)XX**C** (C_P_) active site motifs for each of the three subfamilies across both metazoans and non-metazoans (Fig. 1D). Within metazoans, we find three variable residues within this motif among AhpC-PRX1 sequences, five variable residues among PRX5 sequences and only two variable residues among PRX6 sequences (underlined residues in Fig. 1D). However, we also find that non-metazoan sequences typically display more variability, particularly for subfamilies, PRX5 and PRX6.

We note that PRX classification based on active site profiles has been adopted in recent literature, such as^46^ and^47^, although there still are exceptions, such as^48,49,50,51^. Continuing challenges are the incorrect, vague or ambiguous annotations in online gene databases, in addition to annotations based on older nomenclature thus does not easily correspond with current literature^41,45^. Antioxidant or peroxiredoxin-specific online databases have been developed in attempts to address these challenges (e.g. PREX: http://csb.wfu.edu/PREX/, or RedoxiBase: http://peroxibase.toulouse.inra.fr/), but are not updated frequently enough to be as useful as larger databases (e.g., NCBI) that capture a greater, and constantly-growing, breadth of PRX sequence diversity. Thus, it is often the less accurate annotations that are most commonly used. Few studies have described PRXs across diverse metazoan phyla, and even less so in an evolutionary context^44^. Consequently, we suggest that numerous apparently inaccurate online data base annotations may underestimate the true extent of metazoan PRX diversity, and we predict that a greater breadth of PRX research will reveal further lineage-specific PRXs.

### PRX5 is the least conserved animal PRX subfamily

The subfamily PRX5 is considered to be the closest animal orthologue to the ancestral, prokaryotic subfamily, PRXQ^52^; in our study, it also appears to be the least conserved PRX subfamily. PRX5 displays greater sequence diversity at the C_R_ active site than subfamily AhpC-PRX1. Notably, in three sequences from two sponges (phylum Porifera; *X. bergquistia* and *A. queenslandica*), the catalytic cysteine of C_R_ is replaced by a Valine (V) residue. Further, the bilaterians *B. floridae* and *Capitella teleta* both encode shortened PRX5 sequences in which the C_R_ motif is absent altogether (Supplementary file 1, Fig. S2). Similarly, amongst PRX5 encoded by non-metazoans, we find that the catalytic C_R_ is substituted in all sequences except for that of the choanoflagellate, *M. brevicollis*. This is consistent with other studies that have noted that C_R_ is not always present within atypical 2-Cys PRXs^41^. Additionally, PRX5 is absent from five species, making it the only subfamily with evidence of metazoan gene losses (Table 1). However, these five species do encode alternative sequences that are mitochondrially localised, as PRX5 typically is. Indeed, all metazoans except *N. vectensis* encode at least one mitochondrially-localised PRX (Fig. 3). In mammals, mitochondrially-localised PRX3 is predicted to compensate PRX5 functioning (Table 1)^53^, and *D. melanogaster* mutants lacking PRX3 show few effects, supporting a functional redundancy of PRX5 and PRX3^54^.

In phylum Porifera, PRX5 is the only PRX subfamily for which we do not recover a monophyletic demosponge clade, but rather the freshwater (FW) demosponge *Ephydatia muelleri* branches independently from the three marine (Mar) demosponges (Fig. 4).

In contrast, PRX6 is present in all 19 metazoan species, and is the most consistently localised PRX subfamily; all species have PRX6 genes predicted to localise to the cytoplasm (Fig. 3). Only *Oscarella carmela* and *D. melanogaster* that encode multiple PRX6 genes have one of these localised to nuclei as well as to the cytoplasm (Fig. 3; Supplementary file 3). PRX6 is unique amongst the PRXs in that it lacks a resolving cysteine (C_R_) and is multifunctional, additionally exhibiting both phospholipase, and PLA_2_ activity^55^. Of the 28 metazoan PRX6 sequences, we found that 19 encode the full PLA_2_ catalytic triad, H… S… D, and nine encode the full **G**X**S**X**G** with no substitutions (Purple residues and purple box, respectively; Supplementary file 1, Fig. S3). PRX6 uses the most abundant free radical scavenger, glutathione, and is considered to “moonlight” as a PHGPX^55^. Its ubiquitous presence and metazoan-wide conservation suggests strong selection for specific PRX6 activity and function.

### Phylum porifera encode PRXs sensitive to hyperoxidation, but lack SRX

For each of the 19 metazoan species, including phylum Porifera, we identified at least one AhpC-PRX1 sequence encoding the full motifs (GGLG and YF) that confer sensitivity to hyperoxidation (SO_2_H) under high concentrations of H_2_O_2_ (Fig. 1A; Supplementary file 1 Fig. S1). Sensitivity to hyperoxidation has so far been observed only in animal AhpC-PRX1 (i.e., typical 2-Cys PRXs), and causes its temporary inactivation until reactivated by the ATP-dependent enzyme Sulfiredoxin (SRX) (Fig. 1b)^56,57,58^. To date, no other mechanisms for reactivation have been described. Thus, it is surprising to find 10 species that encode sensitive PRXs but not the SRX-like reductant; these are the six sponge species, the ctenophore *M. leidyi*, and the bilaterians *C. teleta*, *C. elegans*, and *X. tropicalis* (Supplementary file 1, Table S4)*.* Specifically, we identified seven species encoding the SRX ParBc domain (PF02195; IPR003115) but that lacked the strictly conserved SRX-N terminal binding motif **F**(**S**/**G**)**GCHR** required for catalytic activity (Supplementary file 1, Table S4). SRX has not been widely studied, thus SRX sequence structure may exhibit greater diversity than has currently been described. However, for *X. bergquistia*, *O. carmela*, and *C. elegans*, we could not find even the SRX domain ParBc (PF02195) (Supplementary file 1, Table S4). One possible explanation is that, despite encoding the GGLG and YF motifs, the susceptibility to hyperoxidation for each of these 10 species may in fact be sufficiently low that AhpC-PRX1 inactivation does not occur.

Indeed, it is known in mammals that not all AhpC-PRX1 genes are equally sensitive to hyperoxidation; isoforms PRX1, PRX2, and PRX3 are most susceptible^59,60,61^, whilst PRX4 and PRX5 are more resistant, with PRX4 being protected within the ER^53,62,63^. In marine demosponges, PRX4 is the only AhpC-PRX1 that we identified, and in two of these species it was predicted to localise extracellularly so would not be protected within the ER (Fig. 3; Supplementary file 3). Recently, Bolduc et al.^64^ described how substitution of residues within *a* and *b* motifs increases susceptibility to hyperoxidation. Our assessment of PRX4 sequences revealed that at least one residue is substituted within these motifs across all species except for *C. intestinalis*. Most commonly, the missing residue is His from motif *a*, except for *D. melanogaster* that instead is missing residue Asn/Gly; five species are also missing additional residues (Supplementary file 1, Fig. S1; Table S5). Furthermore, *E. muelleri* and *C. teleta* that lack SRX encode substitutions for two residues (Supplementary file 2, Table S5; *C. elegans* does not encode PRX4, but PRX1/2 that lacks 3 residues). These substitutions suggest that the PRX4 genes of the species lacking SRX are at least somewhat susceptible to hyperoxidation, even if not to the same degree as PRX1-3.

Alternatively, species may encode PRXs that are sensitive to hyperoxidation but that are not reactivated, given that reactivation may not always confer increased fitness. In SRX-depleted *D. melanogaster*, McGinnis et al.^53^ demonstrated that PRX hyperoxidation does not adversely affect resistance to oxidative stress or fly lifespan, but instead results in increased physical fitness and endurance. This result was very surprising given the number of studies that have demonstrated reduced fitness from SRX under expression in cell cultures, plants, and mammals^65,66,67^. One possible explanation is that hyperoxidized PRXs in the SRX mutant could either signal as damage associated molecular patterns (DAMPs) themselves or alter post-translation modifications of other proteins that in turn signal as DAMPs, to induce beneficial response pathways^53^. DAMPs serve as alarm signals within the innate immune system, alerting cells to any damage or to the presence of non-native microbes, which in turn activates host immune responses^68^. Thus, perhaps species that encode sensitive PRX, but not SRX, use hyperoxidized PRXs for other diverse important signalling functions.

### The glutathione peroxidase family

**Figure 5:**
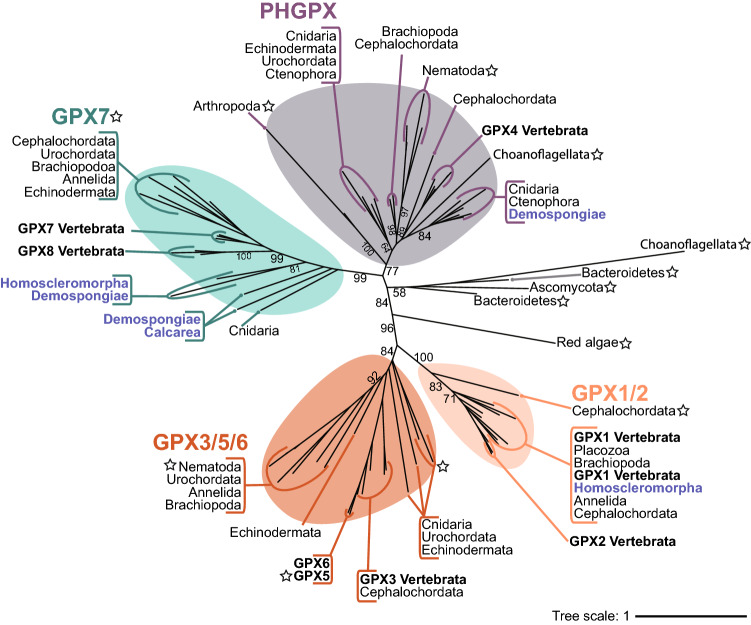
Maximum likelihood phylogenetic tree of GPX enzyme family. Unrooted tree displaying GPX subfamilies, GPX1/2 (peach), GPX3/GPX5/GPX6 (orange), GPX7/GPX8 (turquoise), and PHGPX/GPX4 (grey). Labels in blue denote sequences encoded by phylum Porifera. Labels with a star indicate cysteine GPXs, where cysteine is the catalytic residue. Subfamily GPX7 is exclusively cysteine dependant for all species. Black numbers on branches indicate bootstrap support. Branch lengths represent evolutionary distances, indicated by tree scale. Constructed based on edited alignment, 1000 bb and the LG + G5 evolutionary model.

GPX, the most evolutionary recent antioxidant family to emerge, is considerably less conserved than CAT or PRX. Here we expand on previous assessments^69,37^ by surveying an additional four species of Porifera, as well as the annelid *C. teleta* and urochordate *C. intestinalis*, not included by Trenz et al.^37^. In these six species, we identified 19 unique protein sequences encoding the GSHPx domain. Filtering by domain structure characteristic of a GPX reduced this number to 15 (Fig. 1D; Supplementary file 2). Aside from subfamily GPX7/8, selenocysteine GPXs are widespread across the other six GPX subfamilies. The only exceptions to this include cysteine-dependent GPXs in *D. melanogaster* GPX4, *C. elegans* GPX4 and GPX3/5/6, *S trongylocentrotus purpuratus* GPX3, *B. floridae* GPX1/2, and *Homo sapiens* GPX5 (starred sequences Fig. 5). We find the total number and functional subfamilies of GPX genes encoded by each species is variable, with multiple cases of gene loss. Typically, we find fewer GPX genes in non-bilaterian species, and indeed GPX represents the smallest of the three antioxidant families within phylum Porifera (Table 1). Specifically, we find that GPX7 is most common within phylum Porifera, encoded by four species of classes Homoscleromorpha, Calcarea, and two marine species of Demospongiae, but absent from *X. bergquistia* and the freshwater demosponge *E. muelleri* (Fig. 5). Aside from this, we reveal class Demospongiae encode GPX4-like sequences, and class Homoscleromorpha encodes a putative GPX1/2 but for which only partial sequences were obtained.

Phylogenetic analysis revealed support for four main evolutionary groups, namely GPX1/GPX2 (100%), GPX3/GPX5/GPX6 (84%), GPX7/GPX8 (99%), and PHGPX (i.e., Vertebrata GPX4) (77%; Fig. 5). All metazoan GPX sequences fell into one of these four clades, whilst non-metazoan sequences were paraphyletic and phylogenetically distinct. Only one sequence encoded by the choanoflagellate *M. brevicollis* clustered together with animal GPXs from basal metazoans (phyla Porifera, Ctenophora and Cnidaria) within the subfamily PHGPX/GPX4 (Fig. 5). We found the subfamilies GPX4 and GPX7 are the most abundant across the metazoans.

### Subfamily GPX7 is most conserved across the animal kingdom

Subfamily GPX7, which is exclusively cysteine dependent, is the most commonly encoded GPX in metazoans (Fig. 5). It also shows highly conserved subcellular localisation, being predicted to localise to the ER in 13 of the 14 metazoans that encode it (Fig. 3); this finding is consistent with previous observations^70^.

GPX7 is an animal-specific subfamily that has a key role in facilitating ER protein folding^71^ and has been described as the novel GPX^72^. GPX7 is similar to typical CysGPXs in more efficiently using thiols as its reductant rather than GSH, but different in lacking the second resolving cysteine within the canonical site (Fig. 1C)^73,74^. Instead, GPX7 uses the endoplasmic reticulum (ER) protein disulfide isomerase (PDI) as its reductant, thus helping to recycle it^73^, reviewed by^75^. Within the ER, newly synthesised proteins are oxidised by PDI, which in turn are again re-oxidised by ER oxidoreductase 1 (ERO1α) in a reaction that generates H_2_O_2_^72^, reviewed by^76^. GPX7 can increase PDI-oxidising activity of ERO1α^70,76^, which promotes the refolding of misfolded proteins, and prevents ER oxidative stress response through H_2_O_2_ scavenging^76^. This unique function may explain the strong conservation of GPX7 gene number and localisation across the Metazoa.

In contrast, subfamilies GPX1/2, GPX3/5/6, and GPX4, all of which are predominantly Sec-dependent, are comparatively less conserved. One explanation for this may be their functional redundancy shared with certain PRXs. The subfamily PRX6 that is so well conserved across metazoans is known to “moonlight” as a PHGPX with its similar dependence on GSH^54^. Moreover, typical CysGPXs share a similar catalytic cycle to 2-Cys PRXs and are hypothesised to function in the same way^54,73^ (Fig. 1b,c). Interestingly, GPXs1-6 show positive selection at residues located at or close to active sites, or at the dimer interface^77^. Notably, the catalytic residue within the active site, Sec (U), is encoded by the nucleotide sequence UGA that also encodes the STOP codon^78,79^. It thus requires additional, energetically costly machinery to be encoded^80,81,82^. However, selenocysteine GPXs do exhibit significantly greater efficiency than Cys because of their higher nucleophilic activity, and capacity of Sec to efficiently catalyse both one-electron, as well as two-electron reactions^83,84,85^. Thus, we hypothesise that selection on GPX1-6 may favour seleno-dependent GPXs (i.e., extreme phenotype) that is harder to encode but exhibits greater efficiency. However, without supporting Sec machinery, Sec may not be maintained in the protein, leading to a loss of function. Indeed, in selenocysteine-dependent subfamilies, GPX gene duplications and partial sequences are notably common, particularly within larger genomes of species such as *H. sapiens* (Supplementary file 3) ^77,86^, likely reflecting a more rapid rate of evolution.

## Conclusions

In our survey of 19 species spanning 10 animal phyla, we find that gene number and distribution are highly conserved in the antioxidant families CAT and PRX, but much less so in the GPX family. We reveal for the first time that all three families—CAT, PRX, and GPX—are encoded by the six species of the basal metazoan phylum Porifera, considered sister to all other animal phyletic lineages. From this we can infer the distribution of these three ancient antioxidant families in the last common animal ancestor (LCAA).

Monofunctional CAT comprises a comparatively small and conserved family in animals; its diversification since the LCAA is recent compared to the very long evolutionary history of this enzyme family. We find both peroxisomal and cytoplasmic forms are common among metazoans; the exceptions are that we did not find any of the peroxisomal form in the marine demosponges or cnidarians surveyed in our study. This suggests that the peroxisomal form may have arisen after the cnidarian-bilaterian split, with the addition of signal peptides.

In contrast, the PRXs comprise a large enzyme family. Subfamilies AhpC-PRX1 and PRX6 are the most widely distributed and conserved, whilst PRX5 exhibits notable gene losses. Interestingly, PRX5, the closest animal orthologue to ancestral PRXQ, appears to have been lost in several species that exhibit gene expansion of subfamily AhpC-PRX1. We show that phylum Porifera encode all three animal PRX subfamilies. However, marine demosponges encode just a single AhpC-PRX1, belonging to PRX4, which is the only subclade conserved across the animal kingdom. This indicates that PRX4, which is also found within non-metazoan choanoflagellate, may be the ancestral AhpC-PRX1.

GPX is the most evolutionary recent origin of all the antioxidant enzyme families, is the least conserved among metazoans, and is the least abundant in phylum Porifera. The subfamilies GPX4 and cysteine-dependent GPX7 are the most common in poriferans, with GPX7 present in all three classes, and GPX4 in Demospongiae only. We find strong conservation across the animal kingdom of ER-localised GPX7, which may reflect its unique role of preventing oxidative damage during protein folding within the ER.

That the enzyme families CAT and PRX have been so widely conserved since their ancient origins predating the evolution of aerobic life suggest a core role that is conserved across the animal kingdom. Thus, our comparative genomic analyses illustrate that the fundamental functions of antioxidants have resulted in gene conservation throughout the animal kingdom, paving the way for functional analyses on these enzyme families in diverse animal phyla.

## Methods

### Enzyme identification and subfamily classification

We searched for gene sequences encoding candidate members of the CAT, PRX, and GPX families in high quality genomes of 19 metazoan species representing 10 phyla (Supplementary file 1, Table S1). Specifically, predicted coding sequences were scanned against the Pfam A database using hmmscan in HMMER v3.1b2 (hmmer.org) for sequences encoding domains specific to each enzyme family, and their respective subfamilies (Fig. 1D)^39^. Specifically, HMMER allows us to identify protein sequences encoding functional domains through implementing probabilistic Hidden Markov Models (HMM) to search for protein sequence homologs against a profile database such as Pfam. The number and position of all identified domains was determined. For all identified candidate gene sequences, we predicted protein subcellular localisation regions using DeepLoc-2.0, https://services.healthtech.dtu.dk/service.php?DeepLoc-2.0^40,87^, which uses protein sequences as input for Neural Networks algorithm trained on Uniprot proteins with experimental evidence. The algorithm incorporates the importance (“attention”) of particular amino acids and those within adjacent positions of the region. Positions in the sequence with high “attention” give more weight to the final prediction of the model. DeepLoc-2.0 is able to predict proteins that are located in more than one compartment. The methodology for enzyme identification was cross-validated by comparing the number and type of CAT, PRX, and GPX genes identified through our analysis with those that have previously been described.

Candidate CAT amino acid sequences were identified based on the presence of both of the CAT associated domains PF00199 (catalase) and PF06628 (catalase_rel) (Fig. 1D). No further criteria were applied.

Candidate PRX amino acid sequences were identified based on the presence of at least the PRX domain PF00578, in addition to one or both of PF08534 (AhpC-TSA) and PF10417 (1-cysPrx_C) (Fig. 1D). Candidates were then scanned and filtered based on the presence of the strictly conserved C_P_ active site motif, **P**XXX(**T**/**S**)XX**C** (where X may be any amino acid Wood et al. 2003). The C_P_ motif is required for PRX catalytic activity on H_2_O_2_, so sequences that did not contain this motif were excluded from further analysis. Additionally, we scanned for the presence of subfamily-specific motifs. For subfamily PRX6, this included the catalytic tetrad His, Ser, Asp encoding phospholipase A2 (PLA_2_) and **G**X**S**X**G** motif encoding phospholipase/ esterase (lipase) activity^55^. For subfamily AhpC-PRX1, we searched for the motifs GGLG and YF that encode sensitivity to hyperoxidation, as well as *a* and *b* motifs that contribute to determining the degree of PRX sensitivity to hyperoxidation^56,57,58,64^. Accordingly, we also searched for the presence of enzymatic reductant sulfiredoxin (SRX) that can reactivate hyperoxidized PRXs (Fig. 1B). To do this, we first scanned predicted coding sequences for the presence of the ParB-like nuclease domain (PF02195; IPR003115) and then filtered based on the presence of the strictly conserved SRX-N terminal binding motif **F**(**S**/**G**)**GCHR** that is required for SRX catalytic activity^88^.

Candidate GPX amino acid sequences considered in our study include 61 sequences obtained previously by^37^ from 13 metazoan species indicated in Table S1 (Supplementary file 1). In addition to these, we assessed protein coding sequences of six species, that includes four sponges, that were not assessed by^37^. For these six species, we retained candidate GPX enzyme sequences encoding the domain GSHPx (PF00255; Fig. 1D) and scanned these for the presence of the conserved GPX catalytic tetrad, Sec/Cys (U/C), Gln (Q), Trp (W), and Asn (N) required for GPX activity^89,90^. Sequences encoding Sec at the first residue of the catalytic tetrad were classified as selenium-dependent and those encoding Cys at the first residue of the catalytic tetrad were classified as cysteine dependant GPXs^90^.

### Multiple sequence alignment and phylogenetic assessment

For each enzyme family, candidate sequences were aligned using MAFFT^91,92^ (https://mafft.cbrc.jp/alignment/software/) with default parameters and visualised in the multiple sequence alignment editor AliView v1.27^93^ (https://ormbunkar.se/aliview). To provide evolutionary context to the metazoan phylogenetic relationships, we also incorporated non-metazoan sequences; these included the phylum Choanoflagellata that is closest extant animal relative, as well as other non-metazoan eukaryotics representing Amoebozoa, Red algae, and fungi. Sequences were obtained from UniProt (https://www.uniprot.org/) with specific details provided in Table S2 (Supplementary file 1).

To assess phylogenetic relationships, alignments were manually edited in AliView v1.27^93^ (https://ormbunkar.se/aliview) to remove regions containing more than 50% gaps. Edited alignments were then imported to IQ-TREE^94^ to construct maximum likelihood trees using ultrafast bootstrap^95^, based on 1000 bb and the most appropriate evolutionary model as identified by ModelFinder^96^. Models identified and used to construct each enzyme family tree were as follows: CAT, LG + I + G4, and CAT metazoan LG + G4; PRX, WAG + I + G4; GPX, LG + G5. Resultant phylogenetic trees were first visualised in iTOL v.6.2.1^97^ before annotating in Adobe Illustrator. Classification of PRX and GPX gene subfamilies were inferred from the relative placing of putative sequences within known subfamily clades of phylogenetic trees.

## Supplementary Information

Supplementary Information 1.

Supplementary Information 2.

Supplementary Information 3.

Supplementary Information 4.

## Data Availability

All data generated or analysed during this study are included in this published article, its supplementary information files, and publicly available repositories.

## Abbreviations

Bya: Billion years ago
CAT: Catalase
C_P_: Peroxidatic cysteine
C_R_: Resolving cysteine
DAMPs: Damage associated molecular patterns
DASP: Deacon active site profiler
ER: Endoplasmic reticulum
ERO1α: ER oxidoreductase 1
GOE: Great oxidation event
GPX: Glutathione peroxidase
GSH: Glutathione
H_2_O_2_: Hydrogen peroxide
LCAA: Last common animal ancestor
O_2_^•-^: Superoxide anion radical
PDI: Protein disulfide isomerase
PRX: Peroxiredoxin
ROS: Reactive oxygen species
SRX: Sulfiredoxin
