# Supplementary material for: Antioxidant enzymes that target hydrogen peroxide are conserved across the animal kingdom, from sponges to mammals
Source: Sci Rep. 2023 Feb 13;13:2510. doi: 10.1038/s41598-023-29304-6 (PMC9925728; doi:10.1038/s41598-023-29304-6)
Supplement: Supplementary file 1 — Supplementary Information 1. [file 41598_2023_29304_MOESM1_ESM.pdf]

Table S1. Details of the genomes used in this study.

Species	Phyla	Website/source
Amphimedon queenslandica	Porifera	http://metazoa.ensembl.org/Amphimedon_queenslandica/Info/Index
Xestospongia bergquistia	Porifera	Data available from the authors on request
Tethya wilhelma	Porifera	http://tethya.spongebase.net/
Ephydatia muelleri	Porifera	https://spaces.facsci.ualberta.ca/ephybase/
Oscarella carmela	Porifera	http://compagen.unit.oist.jp/index.html
Sycon ciliatum	Porifera	http://compagen.unit.oist.jp/index.html
Mnemiopsis leidyi	Ctenophora	https://research.nhgri.nih.gov/mnemiopsis/
Nematostella vectensis	Cnidaria	https://www.ebi.ac.uk/ena/browser/view/GCA_000209225.1?show=blobtoolkit
Acanthaster planci	Echinoderm	https://marinegenomics.oist.jp/cots/viewer/info?project_id=46
Strongylocentrotus purpuratus	Echinoderm	https://www.ebi.ac.uk/ena/browser/view/GCA_000002235.4?show=blobtoolkit
Branchiostoma floridae	Chordata	https://mycocosm.jgi.doe.gov/Brafl1/Brafl1.home.html
Ciona intestinalis	Chordata	https://www.ebi.ac.uk/ena/browser/view/GCA_000224145.1
Capitella teleta	Annelida	https://www.ebi.ac.uk/ena/browser/view/GCA_000328365.1?show=blobtoolkit
Lingula anatina	Brachipoda	https://www.ebi.ac.uk/ena/browser/view/GCA_001039355.1
Caenorhabditis elegans	Nematoda	https://downloads.wormbase.org/releases/WS279/species/c_elegans/PRJNA13758/
Drosophila melanogaster	Arthropoda	https://flybase.org/ (release dmel_r6.32 (FB2020_01))
Danio rerio	Chordata	https://www.ncbi.nlm.nih.gov/grc/zebrafish
Xenopus tropicalis	Chordata	http://www.xenbase.org/
Homo sapiens	Chordata	https://www.ncbi.nlm.nih.gov/grc/human

Table S2. Details of sequences used from species for evolutionary comparison. Sequences were obtained from the UniProt data base https://www.uniprot.org/

ID	Phylum	Gene	Accession (UniProt)
Algoriphagus.machipongonensis_1	Bacteroidetes	PRX	A3HSR4_9BACT
Algoriphagus.machipongonensis_2	Bacteroidetes	PRX	A3HY26_9BACT
Algoriphagus.machipongonensis_3	Bacteroidetes	PRX	A3HRY5_9BACT
Algoriphagus.machipongonensis_1_GPx	Bacteroidetes	GPX	A3HYP7_9BACT
Algoriphagus.machipongonensis_2_GPx	Bacteroidetes	GPX	A3HTQ2_9BACT
Arabidopsis.thaliana_CAT3	Spermatophyta	CAT	CATA3_ARATH
Arabidopsis.thaliana_CAT2	Spermatophyta	CAT	CATA2_ARATH
Arabidopsis.thaliana_CAT1	Spermatophyta	CAT	CATA1_ARATH
Podospora.anserina_CAT_B	Ascomycota	KatB	Q9HDP5_PODAS
Podospora.anserina_CAT_A	Ascomycota	KatA	Q9HDP6_PODAS
Podospora.anserina_CAT	Ascomycota	CAT	B2AVW1_PODAN
Podospora.anserina_1	Ascomycota	PRX	B2AMP3_PODAN
Podospora.anserina_2	Ascomycota	PRX	B2AKR1_PODAN
Podospora.anserina_3	Ascomycota	PRX	B2AT30_PODAN
Podospora.anserina_GPx	Ascomycota	GPX	B2B6Q1_PODAN
Phaeosphaeria.nodorum_1_CAT	Ascomycota	CAT	Q0U013_PHANO
Phaeosphaeria.nodorum_2_CAT	Ascomycota	CAT3	Q0UYJ1_PHANO
Phaeosphaeria.nodorum_3_CAT	Ascomycota	CAT2	Q0U1W6_PHANO
Chondrus.crispus_1_CAT	Rhodophyta	CAT	R7QFJ3_CHOCR
Chondrus.crispus_2_CAT	Rhodophyta	CAT	S0F3T7_CHOCR
Chondrus.crispus_PRX3	Rhodophyta	PRX	R7Q3P5_CHOCR
Chondrus.crispus_PRX1	Rhodophyta	PRX	R7Q3N0_CHOCR
Chondrus.crispus_1	Rhodophyta	PRX	M5DDG0_CHOCR
Chondrus.crispus_2	Rhodophyta	PRX	R7Q5I9_CHOCR
Chondrus.crispus_3	Rhodophyta	PRX	R7QGR8_CHOCR
Chondrus.crispus_4	Rhodophyta	PRX	R7QBP3_CHOCR
Chondrus.crispus_1_GPx	Rhodophyta	GPX	R7QCZ5_CHOCR
Pyropia.yezoensis_CAT	Rhodophyta	CAT	E7DDY7_PYRYE
Pyropia.yezoensis_1	Rhodophyta	PRX	YCF42_PYRYE
Pyropia.yezoensis_2	Rhodophyta	PRX	M4QIZ7_PYRYE
Pyropia.haitanensis_3	Rhodophyta	PRX	M9PQP2_PYRHA
Dictyostelium.discoideum_CAT_A	Amoebozoa	CATA	CATA_DICDI
Dictyostelium.discoideum_CAT_B	Amoebozoa	CATB	CATB_DICDI
Dictyostelium.discoideum_PRX4	Amoebozoa	PRX	PRDX4_DICDI
Dictyostelium.discoideum_1Cys	Amoebozoa	PRX	PRDXL_DICDI
Monosiga.brevicollis_CAT	Choanoflagellata	CAT	A9UW79_MONBE
Monosiga.brevicollis_1	Choanoflagellata	PRX	A9VEN1_MONBE
Monosiga.brevicollis_2	Choanoflagellata	PRX	A9USG5_MONBE
Monosiga.brevicollis_3	Choanoflagellata	PRX	A9UXC8_MONBE
Monosiga.brevicollis_4	Choanoflagellata	PRX	A9VBU1_MONBE
Monosiga brevicollis_5	Choanoflagellata	PRX	A9VD18_MONBE
Monosiga.brevicollis_6	Choanoflagellata	PRX	A9V1X1_MONBE
Monosiga.brevicollis_1_GPx	Choanoflagellata	GPX	A9UXZ2_MONBE
Monosiga.brevicollis_2_GPx	Choanoflagellata	GPX	A9V6S1_MONBE
Trichoplax.adhaerens_CAT	Placozoa	CAT	B3SC13_TRIAD
Trichoplax.adhaerens_PRX5a	Placozoa	PRX	B3RM02_TRIAD

Trichoplax.adhaerens_PRX5b	Placozoa	PRX	B3RLY4_TRIAD
Trichoplax.adhaerens_3	Placozoa	PRX	B3RP43_TRIAD
Trichoplax.adhaerens_4	Placozoa	PRX	B3S1P8_TRIAD
Trichoplax.adhaerens_5	Placozoa	PRX	B3RX50_TRIAD
Trichoplax.adhaerens_6	Placozoa	PRX	B3RUE8_TRIAD
Trichoplax.adhaerens_GPx	Placozoa	GPX	B3RVW7_TRIAD

Table S3. PRX classes and subfamilies named according to both profiling of the C_P active site and that used previously (i.e., homology to mammalian named isoforms) classification systems. Conserved residues around the peroxidatic (C_P) and resolving cysteine (C_R) active sites based on 19 assessed metazoan species are indicated. Active sites shown are highly conserved across metazoan species assessed in this study, except for C_R in PRX5, where there is some notable variation within phylum Porifera. Residues that deviated from the amino acid displayed within more than one metazoan sequence are underlined. Residues in bold are absolutely conserved residues. Motifs are not shown for non-animal PRX subclasses, denoted in italicised grey font.

Class	Enzyme Classification	Subfamily classification: Active site profile	Subfamily classification: Mammalian homology	C_P	C_R
**Typical 2-Cys**	EC 1.11.1.24	AhpC-PRX1	PRX1	FFY**P**LDF**T**FV**C**PTEI	GEV**C**PA
	EC 1.11.1.24	AhpC-PRX1	PRX2	FFY**P**LDF**T**FV**C**PTEI	GEV**C**PA
	EC 1.11.1.25	AhpC-PRX1	PRX3	FFY**P**LDF**T**FV**C**PTEI	GEV**C**PA
	EC 1.11.1.24	AhpC-PRX1	PRX4	FFY**P**LDF**T**FV**C**PTEI	GEV**C**PA
	EC 1.11.1.24	AhpC-PRX1	CNID-PRX*	FFY**P**LDF**T**FV**C**PTEI	GEV**C**PA
	EC 1.11.1.26	*AhpC-PRX1*	*AhpC*		
**Atypical 2-Cys**	EC 1.11.1.24	PRX5	PRX5	FAV**P**GAF**T**PG**C**SKTH	GLT**C**SL**
	EC 1.11.1.24	*PRXQ*	*BCP*		
	EC 1.11.1.24	*TPX*	*TPX*		
**1-Cys**	EC 1.11.1.27	PRX6	PRX6	FSH**P**ADY**T**PV**C**TTEL	-
	EC 1.11.1.29	*AhpE*	*AhpE*		

*Recently established subfamily by [39], separated from human PRX4, based on presence in Cnidaria

** For A. queenslandica and X. bergquistia PRX5, the C_R is absent

***Previously EC 1.11.1.15, which only became redundant in 2020 and is still the main EC used in online database annotations.

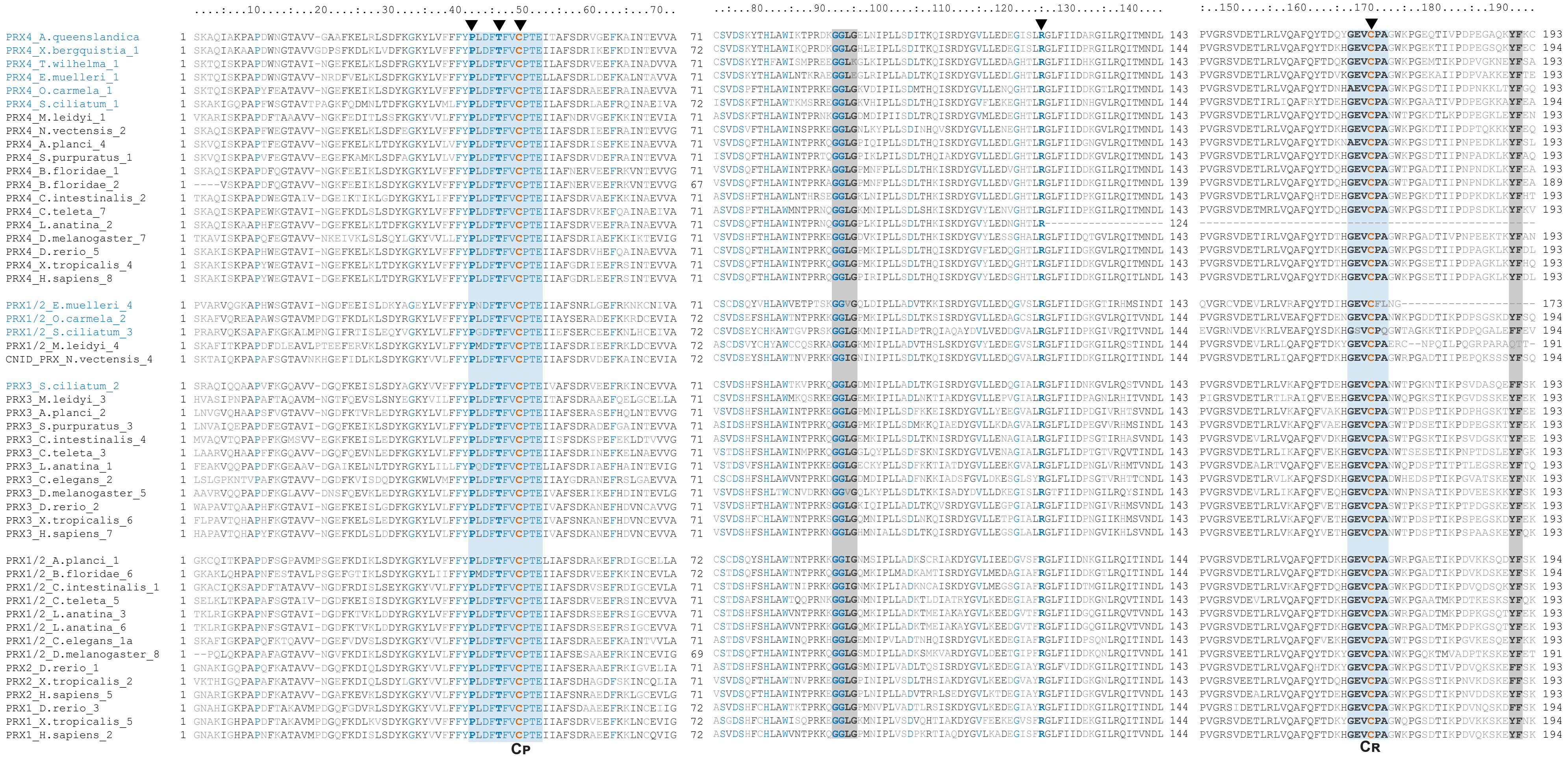

Figure S1. Multiple sequence alignment for AhpC-PRX1 sequences identified from 19 metazoan species. Blue: identical residues, Orange: conserved Peroxidatic (C_P) and resolving (C_R) Cysteine residues, Black: residues with 90% similarity. C_P and C_R conserved motifs highlighted in blue box. Motifs encoding "sensitive" PRX highlighted in bold in dark grey boxes, GGLG…. YF for which 41 of 51 metazoan AhpC-PRX1 sequences had the full motif. Arrowhead denotes absolutely conserved Arginine residue. Species names belong to phylum Porifera are in blue. Sequences aligned using MAFFT.

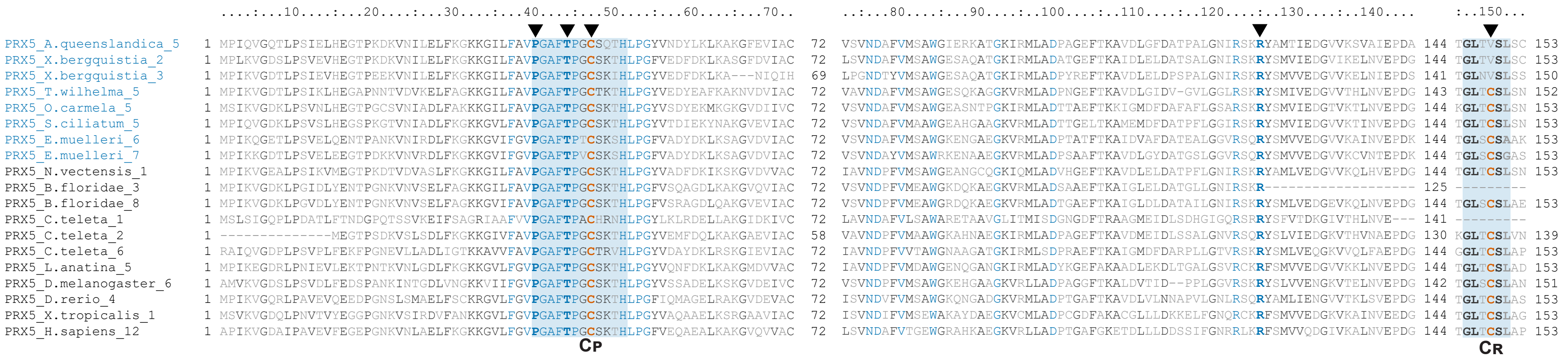

Figure S2. Multiple sequence alignment for PRX5 sequences identified from 19 metazoan. Blue: identical residues, Orange: conserved Peroxidatic (C_P) and resolving (C_R) Cysteine residues, Black: residues with 90% similarity. C_P and C_R conserved motifs highlighted in blue box. Arrowhead denotes absolutely conserved Arginine residue. Species names belong to phylum Porifera are in blue. Sequences aligned using MAFFT.

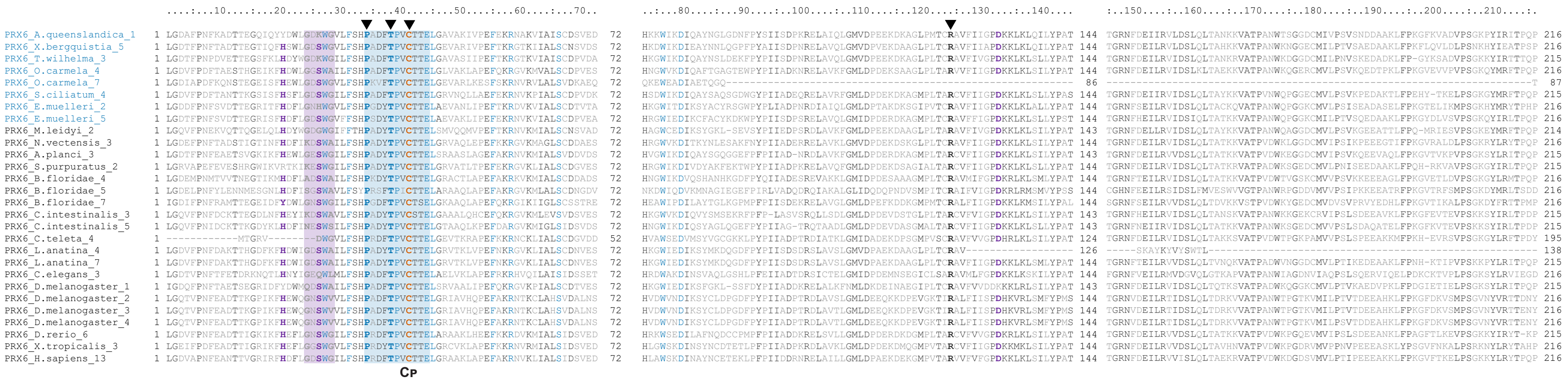

Figure S3. Multiple sequence alignment for PRX6 sequences identified from 19 metazoan species. *Blue*: identical residues, *Orange*: conserved Peroxidatic (C_P) Cysteine residue, *Purple*: Phospholipase A_2 (PLA_2) catalytic triad, *Black*: residues with 90% similarity. C_P conserved motifs highlighted in blue box. Black arrowheads denote peroxidase catalytic triad and active site. Motifs encoding phospholipase/esterase (lipase) activity highlighted in bold, in purple box, **GXSXG**. Species names belong to phylum Porifera are in blue. Sequences aligned using MAFFT.

Table S4. SRX identified from genome sequences of 19 metazoan species. ParBc (PF02195) is the SRX domain.

**Species**	**Phylum**	**Number of genes encoding ParBc domain**	**SRX**
Amphimedon queenslandica	Porifera	3	-
Xestospongia bergquistia	Porifera	-	-
Tethya wilhelma	Porifera	3	-
Ephydatia muelleri	Porifera	3	-
Oscarella carmela	Porifera	-	-
Sycon ciliatum	Porifera	3	-
Mnemiopsis leidyi	Ctenophora	1	-
Nematostella vectensis	Cnidaria	5	SRX
Acanthaster planci	Echinoderm	1	SRX
Strongylocentrotus purpuratus	Echinoderm	1	SRX
Branchiostoma floridae	Chordata	1	SRX
Ciona intestinalis	Chordata	3	SRX
Capitella teleta	Annelida	16	-
Lingula anatina	Brachipoda	2	SRX
Caenorhabditis elegans	Nematoda	-	-
Drosophila melanogaster	Arthropoda	2	SRX
Danio rerio	Chordata	1	SRX
Xenopus tropicalis	Chordata	2	-
Homo sapiens	Chordata	1	SRX

Table S5. Showing presence of motifs, *a* and *b* [61] encoded within sensitive PRX4 sequences belonging to subfamily AhpC-PRX1. Not having these amino acids makes the sequence more prone to hyperoxidation. Since, *C. elegans* does not encode PRX4, the motifs displayed for are for a PRX1/2 gene sequence. Rows highlighted in yellow denote species that do not encode SRX.

**PRX4**	**A MOTIF**						**B MOTIF**	
**Species**	**D**	**N/G**		**H**	**S/G**		**T**	**S/T**
Amphimedon queenslandica	D	N		**K**	S		T	S
Xestospongia bergquistia	D	N		**K**	S		T	S
Tethya wilhelma	D	N		**K**	S		T	S
Ephydatia muelleri	D	N		**K**	**A**		T	S
Oscarella carmela	D	N		**P**	S		T	S
Sycon ciliatum	D	N		**K**	S		T	S
Mnemiopsis leidyi	D	N		**K**	S		T	S
Nematostella vectensis	D	N		**E**	S		T	S
Acanthaster planci	D	N		**Q**	S		T	S
Strongylocentrotus purpuratus	D	N		**Q**	S		T	**A**
Branchiostoma floridae	**E**	N		**Q**	S		T	S
Ciona intestinalis	D	N		H	S		T	S
Capitella teleta	D	N		**P**	S		**S**	S
Lingula anatina	D	N		**Q**	S		T	S
Caenorhabditis elegans prx1/2	D	N		**V**	**A**		**N**	S
Drosophila melanogaster	D	**K**		H	S		T	S
Danio rerio	D	N		**Q**	S		T	S
Xenopus tropicalis	D	N		**Q**	S		T	S
Homo sapiens	D	N		**Q**	S		T	S
