# Supplementary material for: Antioxidant enzymes that target hydrogen peroxide are conserved across the animal kingdom, from sponges to mammals
Source: Sci Rep. 2023 Feb 13;13:2510. doi: 10.1038/s41598-023-29304-6 (PMC9925728; doi:10.1038/s41598-023-29304-6)
Supplement: Supplementary file 4 — Supplementary Information 4. [file 41598_2023_29304_MOESM4_ESM.docx]

**Included within this file:**

Metazoan protein sequences in fasta format for Catalase (CAT), Peroxiredoxin (PRX), and Glutathione peroxidase (GPX)

Alignment for Fig 2A – CAT unrooted tree including non-metazoan sequences p.

Alignment for Fig 2B – CAT rooted tree including metazoan sequences

Alignment for Fig 4 – PRX unrooted tree including non-metazoan sequences

Alignment for Fig 5 – GPX unrooted tree including non-metazoan sequences

**FASTA**

**Catalase (CAT) sequences**

>Acanthaster.planci_4_CAT_gbr.4.17.t1

MASRDKATNQMEEYKKTLDKMDRLTTSTGMPIDNKQATLTAGPRGPVLMQDFAFSDEMSHFGRERIPERVVHAKGAGAFGYFEVTHDISEYTKACVFESIGKKTPVAVRFSTVGGESGSADTARDPRGFAVKFYTEDGNWDLVGNNTPIFFIRDPIFFPSFIHTQKRNPVTHLKDPDMFWDFITLRPESTHQVSFLFSDRGTPDGYRHMNGYGSHTFKLVNKDGKGVYCKFHLKTDQGIRNLNAAQAEALASGDPDYAIRDLYNAISKEDFPSWSVKIQVMTFEQAEQHKDNPFDLTKVWPQAEYPLIPVGKMVLNLNPRNYFAEVEQIAFAPAHMIPGIEPSPDKMLQGRLFSYPDTHRHRLGTNYLQIPVNCPYKAKTRNYQRDGPQCVTDNQNGAPNYYPNSFNGPTDDLKYAQQTFSISGDVARYNTKDDDNFTQPGIFWSKVLTPKDQDALVSNMAGHLKNAQEFIQKRAVDNWSQCSAEWGKRLQAALDEHKAVAAKAAVAKM

>Amphimedon.queenslandica_6_CAT_Aqu3.1.17907_001

MAASKGSTSQLVNFAAYKKSPDILTTSHGHPVDCKTAILTAGAKGPVLLQDYVFLDEMAHFDRERIPERVVHAKGAGAFGYFEVTHDITNYCKAKVFNKIGKRTPIAVRFSTVGGESGSADTVRDPRGFAVKFYTEDGNWDLVGNNTPIFFIRDPILFPSFIHTQKRNPSTHLK

>Amphimedon.queenslandica_5_CAT_Aqu3.1.17905_001

MFWDFISLRPETTHQVSFLFSDRGIPDGYRHMNGYGSHTFKLVNNEGEPVYCKFHYKTDQGIANLSVEKAGILAGSDPDYAIKDLYDAIAAKNYPSWTLYIQVMTFEQAKEFEWNPFDLTKIWPQKEFPLIPVGRMVLNRNPANYFAEVEQLAFSPAHMVAGIEPSPDKMLQGRLFSYDDTHRHRLGPNYHQIPVNCPYATRTRNYQRDGPMTVDGNQGGAPNYFPNSFSGPVDNPEYTISPITLSTCDVKKYNTRDDDNFSQVKNFWLKVLTVEEQSRLVFNIASHLKDAQPFIQSRVIRNFSSVHPDYGSRISDLLQQFKKKKQVSSNL

>Amphimedon.queenslandica_7_CAT_Aqu3.1.27574_001

MAHFDRERIPERVVHAKGAGVFGYFKVSHDITNYCKAKVFNKVGKRAPIAVHFSTVGGESGSADTVRDPRGFAVKFYTEDGNWDLIGNNSPLFFIRDPILFPSFMHTQKRNPSTHLK

>Amphimedon.queenslandica_8_CAT_Aqu3.1.41530_001

MSSKRPACFSQLEEYAKKTQPGEVLTTTHGNPIDFKTAIQTFGPRGPMLMQDGVYLDEMAHFDRERIPERVVHAKGAGAYGVFEVTHDITKYCCAKLFSEVGKKTDLFIRFSTVGGESGSADTARDPRGFAVKFYTEDGNWDLVGNNTPIFFIRDPFLFPSFIHTQKRNPVTHLKDPDMFWDFISLRPETTHQVSFLFSDRGIPDGYRHMNGYGSHTFKLVNSKGEPVYCKFHYKTDQGIKNMPVGKAAELAGTNPDYSIQDLYEAIATGNFPSWTLSIQVMTYEQAEKCSFNPFDLTKVWPHADYPLIPVGKITLNRNPSNYFFDVEQSAFSPAHMPPGIEASPDKMLQGRLFSYDDTHFHRLGPNFQMIPVNCPYAGKPRNYVRDGPMCVDGNQGGAPNYYPNSFNGPKDMGKHDVTIFPGPAGDVKRYNAADDDNFSQVGIFYNKVLNEEERTRLAQNIAGHMKNASPKIQERAIANFSKADPDYGARIKKYISQ

>Branchiostoma.floridae_10_CAT_XP_002607743.1

PSWTLKIQVMTFEEAEKFRFNPFDLTKVWPQGEFPLIPVGKMVLNRNPKNYFAEVEQIAFSPIHMVPGIEASPDKMLQGRLFSYSDTHRHRLGSNYLQIPVNCPYRARVTNYQRDGPQCVDDNQAGAPNCYPNSFSGPKQKETIIQRHLVEPGAPNYYPNSFSGPEQNETITPPAIKTTGDLQRYNTADEDNFTQVGTFWRNVLSEYDREHLVDNLASHMTAAQEFLQKRAVKNFSQCDPEYGRRLQEKLDKYNAAKTIQLQFVSKQERQILNKNPTYRGTLIQEYRKDGDPKIKVFRAENSESVNKENAILTVYSYRSIPPGGGTYVVLQFKASKMYFTAKKTKPGKLILQEPGEFDPEDAEDITTTADRRVFLVKPSQPGSSDVVFASQHKSGDNKNKARVITLLKNRGVPAKFKREGKGPLESQWFQIEHVLAGGASEQRDEGQVQGVY

>Branchiostoma.floridae_9_CAT_XP_002607742.1

MAGRDKAGNQLEEYKKLQNGNASTVTTGTGAPVDNKLAVLTVGPRGPMLMQDFTYMDEMAHFNRERIPERVVHAKGHGAFGYFECTHDISQYCKAKPFEHVGKRTPLGIRFSTVGGESGSADTARDPRGFAVKMYTEDGNWDLVGNNTPIFFIRDPILFPSFIHTQKRNPATHLKDPDMFWDFISLRPETCHQVSFLFSDRGTPNGYRHMNGYGSHTFKMVNNNNEAVYCKFHWKTDQGIKNLTRQQADDLAGSDPDYAGRDLFNAIAEGNYVST

>Caenorhabditis.elegans_15_CAT_Y54G11A.6.1

MPNDPSDNQLKTYKETYPKPQVITTSNGAPIYSKTAVLTAGRRGPMLMQDVVYMDEMAHFDRERIPERVVHAKGAGAHGYFEVTHDITKYCKADMFNKVGKQTPLLVRFSTVAGESGSADTVRDPRGFSLKFYTEEGNWDLVGNNTPIFFIRDAIHFPNFIHALKRNPQTHMRDPNALFDFWMNRPESIHQVMFLYSDRGIPDGFRFMNGYGAHTFKMVNKEGNPIYCKFHFKPAQGSKNLDPTDAGKLASSDPDYAIRDLFNAIESRNFPEWKMFIQVMTFEQAEKWEFNPFDVTKVWPHGDYPLIEVGKMVLNRNVKNYFAEVEQAAFCPAHIVPGIEFSPDKMLQGRIFSYTDTHYHRLGPNYIQLPVNCPYRSRAHTTQRDGAMAYESQGDAPNYFPNSFRGYRTRDDVKESTFQTTGDVDRYETGDDHNYEQPRQFWEKVLKEEERDRLVGNLASDLGGCLEEIQNGMVKEFTKVHPDFGNALRHQLCQKKH

>Caenorhabditis.elegans_16_CAT_Y54G11A.6.2

MPNDPSDNQLKTYKETYPKPQVITTSNGAPIYSKTAVLTAGRRGPMLMQDVVYMDEMAHFDRERIPERVVHAKGAGAHGYFEVTHDITKYCKADMFNKVGKQTPLLVRFSTVAGESGSADTVRDPRGFSLKFYTEEGNWDLVGNNTPIFFIRDAIHFPNFIHALKRNPQTHMRDPNALFDFWMNRPESIHQVMFLYSDRGIPDGFRFMNGYGAHTFKMVNKEGNPIYCKFHFKPAQGSKNLDPTDAGKLASSDPDYAIRDLFNAIESRNFPEWKMFIQVMTFEQAEKWEFNPFDVTKVWPHGDYPLIEVGKMVLNRNVKNYFAEVEQAAFCPAHIVPGIEFSPDKMLQGRIFSYTDTHYHRLGPNYIQLPVNCPYRSRAHTTQRDGAMAYESQGDAPNYFPNSFRGYRTRDDVKESTFQTTGDVDRYETGDDHNYEQPRQFWEKVLKEEERDRLVGNLASDLGGCLEEIQNGMVKEFTKVHPDFGNALRHQLCQKKH

>Caenorhabditis.elegans_13_CAT_Y54G11A.5.1

MPNDPSDNQLKTYKETYPKPQVITTSNGAPIYSKTAVLTAGRRGPMLMQDVVYMDEMAHFDRERIPERVVHAKGAGAHGYFEVTHDISKYCKADIFNKVGKQTPLLIRFSTVGGESGSADTARDPRGFAIKFYTEEGNWDLVGNNTPIFFIRDPIHFPNFIHTQKRNPQTHLKDPNMIFDFWLHRPEALHQVMFLFSDRGLPDGYRHMNGYGSHTFKMVNKDGKAIYVKFHFKPTQGVKNLTVEKAGQLASSDPDYSIRDLFNAIEKGDFPVWKMFIQVMTFEQAEKWEFNPFDVTKVWPHGDYPLIEVGKMVLNRNPRNYFAEVEQSAFCPAHIVPGIEFSPDKMLQGRIFSYTDTHFHRLGPNYIQLPVNCPYRSRAHNTQRDGAMAYDNQQHAPNFFPNSFNYGKTRPDVKDTTFPATGDVDRYESGDDNNYDQPRQFWEKVLDTGARERMCQNFAGPLGECHDFIIKGMIDHFSKVHPDFGARVKALIQKQARSHI

>Caenorhabditis.elegans_14_CAT_Y54G11A.5.2

MPNDPSDNQLKTYKETYPKPQVITTSNGAPIYSKTAVLTAGRRGPMLMQDVVYMDEMAHFDRERIPERVVHAKGAGAHGYFEVTHDISKYCKADIFNKVGKQTPLLIRFSTVGGESGSADTARDPRGFAIKFYTEEGNWDLVGNNTPIFFIRDPIHFPNFIHTQKRNPQTHLKDPNMIFDFWLHRPEALHQVMFLFSDRGLPDGYRHMNGYGSHTFKMVNKDGKAIYVKFHFKPTQGVKNLTVEKAGQLASSDPDYSIRDLFNAIEKGDFPVWKMFIQVMTFEQAEKWEFNPFDVTKVWPHGDYPLIEVGKMVLNRNPRNYFAEVEQSAFCPAHIVPGIEFSPDKMLQGRIFSYTDTHFHRLGPNYIQLPVNCPYRSRAHNTQRDGAMAYDNQQHAPNFFPNSFNYGKTRPDVKDTTFPATGDVDRYESGDDNNYDQPRQFWEKVLDTGARERMCQNFAGPLGECHDFIIKGMIDHFSKVHPDFGARVKALIQKQARSHI

>Caenorhabditis.elegans_11_CAT_Y54G11A.13a

MPMLPHMVNLTVNDTKPGPMAEDQLKAYRDRNQEPHLLTTSNGAPIYSKTAVLTAGRRGPMLMQDIVYMDEMAHFDRERIPERVVHAKGGGAHGYFEVTHDITKYCKADMFNKVGKQTPLLVRFSTVAGESGSADTVRDPRGFSLKFYTEEGNWDLVGNNTPIFFIRDAIHFPNFIHALKRNPQTHMRDPNALFDFWMNRPESIHQVMFLYSDRGIPDGFRFMNGYGAHTFKMVNKEGNPIYCKFHFKPAQGSKNLDPTDAGKLASSDPDYAIRDLFNAIESRNFPEWKMFIQVMTFEQAEKWEFNPFDVTKVWPHGDYPLIEVGKMVLNRNVKNYFAEVEQAAFCPAHIVPGIEFSPDKMLQGRIFSYTDTHYHRLGPNYIQLPVNCPYRSRAHTTQRDGAMAYESQGDAPNYFPNSFRGYRTRDDVKESTFQTTGDVDRYETGDDHNYEQPRQFWEKVLKEEERDRLVGNLASDLGGCLEEIQNGMVKEFTKVHPDFGNALRHQLCQKKH

>Caenorhabditis.elegans_12_CAT_Y54G11A.13b

MAEDQLKAYRDRNQEPHLLTTSNGAPIYSKTAVLTAGRRGPMLMQDIVYMDEMAHFDRERIPERVVHAKGGGAHGYFEVTHDITKYCKADMFNKVGKQTPLLVRFSTVAGESGSADTVRDPRGFSLKFYTEEGNWDLVGNNTPIFFIRDAIHFPNFIHALKRNPQTHMRDPNALFDFWMNRPESIHQVMFLYSDRGIPDGFRFMNGYGAHTFKMVNKEGNPIYCKFHFKPAQGSKNLDPTDAGKLASSDPDYAIRDLFNAIESRNFPEWKMFIQVMTFEQAEKWEFNPFDVTKVWPHGDYPLIEVGKMVLNRNVKNYFAEVEQAAFCPAHIVPGIEFSPDKMLQGRIFSYTDTHYHRLGPNYIQLPVNCPYRSRAHTTQRDGAMAYESQGDAPNYFPNSFRGYRTRDDVKESTFQTTGDVDRYETGDDHNYEQPRQFWEKVLKEEERDRLVGNLASDLGGCLEEIQNGMVKEFTKVHPDFGNALRHQLCQKKH

>Capitella.teleta_17_CAT_CapteP163635

MPFLDRLREVLQDEDKPRRNGSDMANRDKASEQLNEYKQSNGTPGVLTTATGAPIGNKTAIQTVGPRGPALLQDFVFQDEMSHFGRERIPERVVHAKGAGAFGFFEVTHDITKYSKAKVFEHIGKKTPIVARLSTVGGEKGSADTARDPRGFAVKFYTDEGNWDLVGNNTPIFFIRDPMLFPSFIHTQKRNPKTNLKDPDAFWDFLTLRPESCHQVSFLFSNRGTPDGYRNMNGYGSHTFKLVNKEGVAHYCKFHYKTNQGIKNLTGAQADALAGSDPDYATRDLYNAIAEGNFPSYTLFIQVMTFEEAEKHRFNPFDLTKVWSHKEFPLIPVGRLTFNRNPKNYFAEVEQVAFSPAHMVPGIEASPDKMLQGRLYSYSDTHRHRLGTNYQQIPVNCPFSTRARNYQRDGPQNVDDNQEGAPNYFPNSFGGPQDSPAFLEHETTFPGDVARYNTKDDDNFTQVGIFWRETLTEEDRKHLIINMSGHLKNAQEFLQKRAVANFSKCDPEYGRRLQEALDQHKKDAQSVSMAAQL

>Ciona.intestinalis_18_CAT_ENSCINT00000010672

MGRTKSDNQLKEYAEKNKDKTVLMTGTGAPIEDKLNVLTVGERGPLLMQDFTFTDEMAHFNRERIPERVVHAKGGGAKGYLEITHDISNFCKADIFSSIGKRTPLAVRFSTVGGESGSADSARDPRGFAIKFYTEEGIWDLVGNNTPIFFIRDPIFFPSFIHTQKRNPQTHLKDPDMFWDFISLRPETTHQVSHILVMRVNTPQGVIHFNTTDNWCNISITLYQCNSTIGRFKTDQGIKNLTADEADTLAATDPDHAIRDLYNAIADGNNPSWTMYIQVMTYQQATTHKWNPFDLTKTWPQGEFPLIQVGKMVLNENPSNYFAEVEQIAFSPSHMVPGIEASPDKMLQGRLFSYPDTHRHRLGSNYLQIPINCPFNVRGGKVNNYQRDGPQCVTDNSKGAPNYYPNSFNGPLDGAHTCPASRKQTSLHVDRQPASDVKKYNSADDDNFTQVGTFWRKVLNEAERKRLAENIGNHMKAAQPFLQKRAIANFAAADPEFGAMIQAVIDKAAAKILTKVALNNQ

>Danio.rerio_20_CAT_XP_021326116.1

MADDREKSTDQMKLWKEGRGSQRPDVLTTGAGVPIGDKLNAMTAGPRGPLLVQDVVFTDEMAHFDRERIPERVVHAKGAGAFGYFEVTHDITRYSKAKVFEHIGKTTPIAVRFSTVAGEAGSSDTVRDPRGFAVKFYTDEGNWDLTGNNTPIFFIRDTLLFPSFIHSQKRNPQTHLKDPDMVWDFWSLRPESLHQVSFLFSDRGIPDGYRHMNGYGSHTFKLVNAQGQPVYCKFHYKTNQGIKNIPVEEADRLAATDPDYSIRDLYNAIANGNFPSWTFYIQVMTFEQAENWKWNPFDLTKVWSHKEFPLIPVGRFVLNRNPVNYFAEVEQLAFDPSNMPPGIEPSPDKMLQGRLFSYPDTHRHRLGANYLQLPVNCPYRTRVANYQRDGPMCMHDNQGGAPNYYPNSFSAPDVQPRFLESKCKVSPDVARYNSADDDNVTQVRTFFTQVLNEAERERLCQNMAGHLKGAQLFIQKRMVQNLMAVHSDYGNRVQALLDKHNAEGKKNTVHVYSRGGASAVAAASKM

>Danio.rerio_21_CAT_XP_021326117.1

MADDREKSTDQMKLWKEGRGSQRPDVLTTGAGVPIGDKLNAMTAGPRGPLLVQDVVFTDEMAHFDRERIPERVVHAKGAGAFGYFEVTHDITRYSKAKVFEHIGKTTPIAVRFSTVAGEAGSSDTVRDPRGFAVKFYTDEGNWDLTGNNTPIFFIRDTLLFPSFIHSQKRNPQTHLKDPDMVWDFWSLRPESLHQVSFLFSDRGIPDGYRHMNGYGSHTFKLVNAQGQPVYCKFHYKTNQGIKNIPVEEADRLAATDPDYSIRDLYNAIANGNFPSWTFYIQVMTFEQAENWKWNPFDLTKVWSHKEFPLIPVGRFVLNRNPVNYFAEVEQLAFDPSNMPPGIEPSPDKMLQGRLFSYPDTHRHRLGANYLQLPVNCPYRTRVANYQRDGPMCMHDNQGGAPNYYPNSFSAPDVQPRFLESKCKVSPDVARYNSADDDNVTQVRTFFTQVLNEAERERLCQNMAGHLKGAQLFIQKRMVQNLMAVHSDYGNRVQALLDKHNAEGKKNTVHVYSRGGASAVAAASKM

>Danio.rerio_19_CAT_NP_570987.2

MADDREKSTDQMKLWKEGRGSQRPDVLTTGAGVPIGDKLNAMTAGPRGPLLVQDVVFTDEMAHFDRERIPERVVHAKGAGAFGYFEVTHDITRYSKAKVFEHIGKTTPIAVRFSTVAGEAGSSDTVRDPRGFAVKFYTDEGNWDLTGNNTPIFFIRDTLLFPSFIHSQKRNPQTHLKDPDMVWDFWSLRPESLHQVSFLFSDRGIPDGYRHMNGYGSHTFKLVNAQGQPVYCKFHYKTNQGIKNIPVEEADRLAATDPDYSIRDLYNAIANGNFPSWTFYIQVMTFEQAENWKWNPFDLTKVWSHKEFPLIPVGRFVLNRNPVNYFAEVEQLAFDPSNMPPGIEPSPDKMLQGRLFSYPDTHRHRLGANYLQLPVNCPYRTRVANYQRDGPMCMHDNQGGAPNYYPNSFSAPDVQPRFLESKCKVSPDVARYNSADDDNVTQVRTFFTQVLNEAERERLCQNMAGHLKGAQLFIQKRMVQNLMAVHSDYGNRVQALLDKHNAEGKKNTVHVYSRGGASAVAAASKM

>Drosophila.melanogaster_23_CAT_FBgn0032061

MCSRDTASNQLIDYKNNDSEVQREITTSSGTPVGVKDAIQTVGPRGPALLQDFQFLDEVMHFDSERIPERVAYAKGAGAFGYFECTHDISKFCAASIFDKVRKRTAVAMRFSVACGEQGSADTVREQRGFAVKFYTDDGIWDIVGCNMPVHYVRDPMLFPSLVHAQKRNPQTHLKDPDMFWDFMTLRPETLHALLMYFSDRGTPDGYRHLHGYGVHTYRMINASGETQYVRFHFKTDQGIKNLDARRCEELMSHDPDYAIRDLYNSIKKGNYPSWSMYIQVMLNEEAKKCRFNPFDVTKVWPQKDFPLLPVGKIVLDRNPTNYFTEVEQLAFSPAHMVPGIEPSPDKMLQGRLFAYGDSQRHRLGVNYMQIPVNCPYRVNVRNFQRDGAMTVTDNQNGAPNYFPNSFCGPRESPRALGLQTCCPLSGDVYRFMSGDTEDNFSQVTDFWTYTLDNCGRKRLVRNLSEHLTEASQFLQERAVKLFTMVHSDFGRLMTEALNTARISKF

>Drosophila.melanogaster_22_CAT_FBgn0000261

MAGRDAASNQLIDYKNSQTVSPGAITTGNGAPIGIKDASQTVGPRGPILLQDVNFLDEMSHFDRERIPERVVHAKGAGAFGYFEVTHDITQYCAAKIFDKVKKRTPLAVRFSTVGGESGSADTARDPRGFAVKFYTEDGVWDLVGNNTPVFFIRDPILFPSFIHTQKRNPQTHLKDPDMFWDFLTLRPESAHQVCILFSDRGTPDGYCHMNGYGSHTFKLINAKGEPIYAKFHFKTDQGIKNLDVKTADQLASTDPDYSIRDLYNRIKTCKFPSWTMYIQVMTYEQAKKFKYNPFDVTKVWSQKEYPLIPVGKMVLDRNPKNYFAEVEQIAFSPAHLVPGVEPSPDKMLHGRLFSYSDTHRHRLGPNYLQIPVNCPYKVKIENFQRDGAMNVTDNQDGAPNYFPNSFNGPQECPRARALSSCCPVTGDVYRYSSGDTEDNFGQVTDFWVHVLDKCAKKRLVQNIAGHLSNASQFLQERAVKNFTQVHADFGRMLTEELNLAKSSKF

>Ephydatia.muelleri_24_CAT_Em0014g600a

MAENRSNCANQLIDHAHSKSVADADPITTSHGCPVDSKTASLTVGERGLIPIQDFTFLDEMAHFDRERIPERVVHAKGAGAFGYFEVTHDITKYCKAKIFSHVGKKTPIAIRFSTVGGENGSADTVRDPRGFAIKFYTEEGNWDLVGNNTPIFFIRDPILFPSFIHTQKRNPATHLKDPDMFWDFITLRPETTHQVSFLFSDRGIPDGYRHMNGYGSHTFKLVNKAGEAVYCKFHFKTDQGIKNLSVQKAGELSMNDPDYSIRDLYESIATGNFPSWTLFLQVMTFEQAEKFRLNPFDLTKVWPHSEYPLIPVGKLVLNRNPKNYFAEVEQIAFCPAHLVPGVEPSPDKMLQGRLFSYSDTHRHRLGSNYHMIPVNCPYATKANTYHRDGSMCVDSNQGGAPNYYPNSFQGPVDSQIHALSKTTVVTGDVKRYNTADHDNFSQVTNFWTKVLSEEEKTRLVENIAGHLKDATEFIQKRAVQNFTAVHRDYGGRLNLLLQQYKKKVSSPSHL

>Ephydatia.muelleri_25_CAT_Em0021g409a

MADTRPKCQRQLEEYYSRQKAPEILTTSHGHPIDDKKATMTVGARGGVALEDFVFLDEMAHFDRERIPERVVHAKGAGAFGYFEVTHDITKYTKAKVFSQIGKKTPLAVRFSTVGGENGSADTVRDPRGFAVKFYTEDGNWDLVGNNTPIFFIRDPIFFPSFIHTQKRNPATHLKDPDMFWDFISLRPETTHQVSFLFSDRGIPDGYRHMNGYGSHTFKLVNSDGTPVYCKFHYKTDQGIKNLPVDQANKLAGDNPDYAIEDLYESIANGKYPSWTMYIQVMTFEQAEKVAFNPFDLTKVWSHADFPLIPVGKLVLDRNPRNYFAEVEQLAFSPAHMPPGIEASPDKMLQGRLFSYSDTHRHRLGPNSHLIPVNSAQCCRPRNYQRDSFMCTDGNQSDAPNYFPNSFNGPKDNPAVACSKHSFSGDVQRHRSDDEDNFTQAGIFYRKVLTDDQRTRLVEKHSWSCEVCCSIHPGACG

>Homo.sapiens_1_CAT_CAT

QDVVFTDEMAHFDRERIPERVVHAKGAGAC

>Homo.sapiens_2_CAT_CAT.2

MADSRDPASDQMQHWKEQRAAQKADVLTTGAGNPVGDKLNVITVGPRGPLLVQDVVFTDEMAHFDRERIPERVVHAKGAGAFGYFEVTHDITKYSKAKVFEHIGKKTPIAVRFSTVAGESGSADTVRDPRGFAVKFYTEDGNWDLVGNNTPIFFIRDPILFPSFIHSQKRNPQTHLKDPDMVWDFWSLRPESLHQVSFLFSDRGIPDGHRHMNGYGSHTFKLVNANGEAVYCKFHYKTDQGIKNLSVEDAARLSQEDPDYGIRDLFNAIATGKYPSWTFYIQVMTFNQAETFPFNPFDLTKVWPHKDYPLIPVGKLVLNRNPVNYFAEVEQIAFDPSNMPPGIEASPDKMLQGRLFAYPDTHRHRLGPNYLHIPVNCPYRARVANYQRDGPMCMQDNQGGAPNYYPNSFGAPEQQPSALEHSIQYSGEVRRFNTANDDNVTQVRAFYVNVLNEEQRKRLCENIAGHLKDAQIFIQKKAVKNFTEVHPDYGSHIQALLDKYNAEKPKNAIHTFVQSGSHLAAREKANL

>Homo.sapiens_3_CAT_CAT.3

MADSRDPASDQMQHWKEQRAAQKADVLTTGAGNPVGDKLNVITVGPRGPLLVQDVVFTDEMAHFDRERIPERVVHAKGAGAFGYFEVTHDITKYSKAKVFEHIGKKTPIAVRFSTVAGESGSADTVRDPRGFAVKFYTEDGNWDLVGNNTPIFFIRDPILFPSFIHSQKRNPQTHLKDPDMVWDFWSLRPESLHQVSFLFSDRGIPDGHRHMNGYGSHTFKLVNANGEAVYCKFHYKTDQGIKNLSVEDAARLSQEDPDYGIRDLFNAIATGKYPSWTFYIQVMTFNQAETFPFNPFDLTKVWPHKDYPLIPVGKLVLNRNPVNYFAEVEQIAFDPSNMPPGIEASPDKMLQGRLFAYPDTHRHRLGPNYLHIPVNCPYRARVANYQRDGPMCMQDNQGGAPNYYPNSFGAPEQQPSALEHSIQYSGEVRRFNTANDDNVTQVRAFYVNVLNEEQRKRLCENIAGHLKDAQIFIQKKAVKNFTEVHPDYGSHIQALLDKYNAEKPKNAIHTFVQSGSHLAAREKANL

>Lingula.anatina_26_CAT_g3728.t1

MQDFVFMDEMAHFQRERIPERVVHAKGAGAFGFFEVTHDISKYCKAKVFEHIGKRTPVAVRFSTVGGESGSADTARDPRGFAVKMYTDEGNWDCVGNNTPIFFIRDPIFFPSFIHTQKRNPVTHCKDPDMFWDFITLRPESTHQVSFLFSDRGTPDGYRQMNGYGSHTFKLVNKDGEAVYCKFHYKTDQGIKCLMADQAGELAGSDPDYAIRDLYNNIAAGNYPSWTWYIQVMTFEEAEKFRWNPFDLTKVWPQGEFPLIPVGRMVLNRNPKNYFAEVEQIAFSPAHLVPGIEASPDKMLQGRLFSYSDTHRHRLGTNYLQIPVNCPFNTRVKNYQRDGPQCVTDNQEGAPNYFPNSFNGPLDDKRHLESVFQTTGDVKRYNTRDDDNFSQVGLFWRNVLKPDERTRLVENIANHLKDAQEFIQQRAVKNFGQCDPEYGRRIQELLDQFKAKKVSMGGMAQANL

>Nematostella.vectensis_27_CAT_EDO41684

QDANMFWDFITLRPETTHQTSFLFSDRGIPDGFRHMNGYGSHTFKMVNSKGKAVYCKFHVKTDQGIKNCPVERATELAGTDPDYSTRDLYNAIAEGNY

>Oscarella.carmela_28_CAT_m.309601

RCRAHLPPTISSFGSQVEIRPGPKLDHEMSATAKASEQLLNYSKEHGKNAGVCTTGDGIPVDTTTATMTAGHRGPVLLQDYNFLDVMAHFDHERIPERVVHAKGAGAFGYFEVTHDITQYCKAKVFERVGKRTPLAVRFSTVGGESGSADTVRDPRGFAVKFYTDDGNWDLVGNNTPIFFIRDPILFPSFIHTQKRNPVTHLKDPDMFWDFISLRPETTHQVSFLFSDRGIPDGHRHMNGYGSHTFKMVNKDNKPVYVKFHYKTDQGIKNLPVGKAGELAGSDPDYSIRDLYNAIAEGNPPSWTLYIQVMTAEQAEKYKWNPFDVTKVWCHKDAPLIPVGRMVLDRNPVNYFAEVEQIAFSPAHMPPGIEASPDKMLQGRLFSYDDTHRHRVGVNFQQLPVNRPHVKVVNYQRDGPAAIDGNQAGAPNYFPNSFQGPQIQPSVAHSSFGVKGDAVRIETHDDDNFSQAGTFWSDVLNDEERQRLVENIAGHLKDAKEFIQERVVANFSKAHPDYGKRIEELLKKYKA

>Oscarella.carmela_29_CAT_m.309963

MSSRTKASEQLSDYASFRSTPENCTTGDGTPVDVKTATMTAGPRGPVLLQDYNFLDEMAHFDRERVPERIVHAKGAGAFGYFEVTHDITRFCKAKVFESVGKKTPLAIRFSTVGGESGSADTVRDPRGFAVKFYTEEGNWDLVGNNTPIFFIRDPILFPSFIHTQKRNPVTHLKDPDMFWDFISLRPETTHQVSFLFSDRGIPDGYRHMNGYGSHTFKMVNKKDEPVYAKFHYKTDQGIQCLPVDKAGDLSGSDPDYGIRDLYNAIAQGKFPSWTLYIQVMTFEQAEKHRWNPFDVTKVWPHSEFPLIQVGRITLNRNPVNYFAEVEQIGFSPAHMPPGIEPSPDKMLQGRLFSYDDTHRHRIGANYLQLPVNCSQNVKVRNYQRDGPMTIDNNQAGAPNYFPNSFQGPAEKRQAGPTKFHVSGDVARYNSADDDNFTQAGTFWTKVLNEEERQRLVENIAGHLKDAKDFIQARAVRNFSQAHPDYGRRIADLLAKYSQRLFSSNL*

>Strongylocentrotus.purpuratus_30_CAT_SPU_000281

MTAGARGPVLIQDFVFTDEMSHFGRERIPERVVHAKGAGAFGYFETTHDISKYCKAAPFESVGKKTPVAIRFSTVGGESGSADTARDPRGFAVKFYSEDGNWDLVGNNTPIFFIRDPMFFPSFIHTQKRNPVTHLKHSYQYNFYDGKFQFCPGGESGSADTARDPRGFAVKFYSEDGNWDLVGNNTPIFFIRDPMFFPSFIHTQKRNPVTHLKDPDMFWDFITLRPEATHQVSFLFSDRGTPDGYRHMNGYGSHTFKLRNKDGEYVFCKFHFKCDQGIKNLNRHRAGDLSATDPDYAIRDLYNSIATGNFPSWSLHIQVMTQEQADKHRDNPFDLTKGRLFSYSDTHRHRLGTNYLQIPVNCPFAARTRSYQRDGPQCVTDNQGGAPNYFPNSFTGPTDSKSYEQTKFTCPGEAARYETGDDDNYTQAGIFWRDVLSEADREATVDNMASHIKDAAEYLQKRTVVQWGKCDEDWGKRLEAKLAEYKTQASATANL

>Sycon.ciliatum_31_CAT_scpid35212

MAAKSKCPNQLQDYAKGKKNSDKLTTSAGCPVDSKTSTMTVGPRGPVLLQDQVFLDDMAHFDRERIPERVVHAKGAGAFGYFEVTDDITKYCKAKIFEQVGKRTPMAVRFSTVGGESGSADTVRDPRGFAVKFYTEEGNWDLVGNNTPIFFIRDPILFPSFIHTQKRNPQTHLKDADMFWDFISLRPETTHQVSFLFSDRGIPNGYRFMNGYGSHTFKLVNARNEPVYCKFHYKTDQGIQNLPVDQAGSLASSDPDYSIRDLFESIAQGNFPSWTMYVQVMTYEQAEKWKWNPFDLTKIWPHKEFPLIKAGRFVLNRNPKNYFAEVEQLAFSPAHLVPGIEPSPDKMLQGRLFSYTDTHRHRLGANYQQLPVNCPFATRPSNYQRDGPMAMGDNQGGAPNYYPNSFSGPRDDPKYAACPERVSGDVKKYNTADDDNFTQAGLFYRSVLNEAEKQRLVENIAGHMKDAKPFIQQRAVRNFAMADPDYGARISRLLAKYQSSSRQQSQL

>Sycon.ciliatum_32_CAT_scpid56677

MFLQASCRRFLSTSAYAMAGRCPAANQLLNYREEQKESTTTSTSWGAPVDIATASQTVGPRGPLLLQDANFIDNLAHFDRERIPERVVHAKGAGAFGELVITEDISKYTKALALQKGTSTPVAVRFSTVGGEAGSADTVRDPRGFAVKHYTDDGVWDLVGNNTPIFFIRDPILFPNFIHTQKRNPRTHLKDPDMFWDFISLRPETTHQVSFLFSDRGIPDGYRHMNGYGSHTFKLVNSEGVAHYCKFHYKTSESKNLLPNKAGPISGDDPDYAIRDLYNAIERKEFPTWKFQIQVMTFEQAETYKYNPFDLTKVWSHKDFPLIDVGTMTLNRNPEDYFSEVEQIAFSPSHMVPGIEPSPDKMLQGRLFSYTDTHRHRLGANYQHLPVNASKKTPQANSHRDGPMCMFNQAGAPNYFPNSFKGPVDRPDARHSKISYSGDVEKYNSADEDNFSQVGVFYREVLNAEERQRLVENIAGHLKDALPEIQKRTVANFSQADADYGAGIQKLLDGYNK

>Tethya.wilhelma_37_CAT_Twilhelma_g1595.t1

MASDAKRPKHATQLEEYAKEQKTRPVMTTGHGIPIDSKTASMTVGPRGPITLQDVFYLDEMSHFDRERIPERVVHAKGAGAFGYFEVTQDITKYCKADLFSEVGKKTPIGIRFSTVGGESGSADTVRDPRGFAVKFYTQEGNWDLVGNNTPIFFIRDPLHFPNFIHTQKRNPVTHLKDHDMFWDFLTLRQESAHQVSFLFSDRGIPDGYRHMNGYGSHTFKLVNKDGEPVYCKFHYKTNQGIKNILPEEAEKMAGRDPDYAIRDLYDAIANKDFPSWTLSIQVMTFEQAEKTSFNPFDLTKIWPHKDYPLIEVGKMVLDRNPTNYFCEIEQIAFSPSNMVPGVEPSPDKMLQGRLFSYPDTHLYRLGPNYHQLPVNCPYMTKGGCRNYQRDGNMPLEGNQAGAPNYFPNSFQGPVDDRKYRWSKTTVQTCDIDRYETADEDNYSQVGNFYRNVLSEVEKDRLTSNIAGHVKGAAKFIQERVVEMFTKCDPDYGQRIAKKLQG

>Tethya.wilhelma_38_CAT_Twilhelma_g5262.t1

MILNRNPTNYFCEVEQIAFCPAHLVPGIEPSPDKMLQGRLFSYSDTHRHRLGANYHQIPVNCPYATRCKNYQRDGPQQRWIIKQLVEWTTETVGEIVWSTT

>Tethya.wilhelma_33_CAT_Twilhelma_g10792.t1

MADRTKCAKQLTDFSNRKRVPDVLTTSHGHPIDSKTATLTVGEKGPVLLQDFTFLDEMAHFDRERIPERVVHAKGAGAFGYFEVTHDITKYCKAKVFSHVGKRTPIAIRFSTVDPS

>Tethya.wilhelma_34_CAT_Twilhelma_g10792.t2

MADRTKCAKQLTDFSNRKRVPDVLTTSHGHPIDSKTATLTVGEKGPVLLQDFTFLDEMAHFDRERIPERVVHAKGAGAFGYFEVTHDITKYCKAKVFSHVGKRTPIAIRFSTVGKLHCISSN

>Tethya.wilhelma_35_CAT_Twilhelma_g10794.t1

MDVDMFWDFISLRPETTHQVSFLFSDRGIPDGYRHMNGYGSHTFKLVNKDDEAFYCKFHYKTDQGIKCLDVDKAAKLSGTDPDYGIKDLFEAIATGNFPSWTLYIQVMTFEQAENFKWNPFDLTKTTCDVKRYNTADMDNFSQVTTFWRKVLNEQEKSRLVRNIAGHLKDAAEFIQQRAVRNFMQVDPEYGGRIAKLLQEYKKQVLN

>Tethya.wilhelma_36_CAT_Twilhelma_g10794.t2

MDVDMFWDFISLRPETTHQVSFLFSDRGIPDGYRHMNGYGSHTFKLVNKDDEAFYCKFHYKTDQGIKCLDVDKAAKLSGTDPDYGIKDLFEAIATGNFPSWTLYIQVMTFEQAENFKWNPFDLTKTTCDVKRYNTADMDNFSQVTTFWRKVLNEQEKSRLVRNIAGHLKDAAEFIQQRAVRNFMQVDPEYGGRIAKLLQEYKKQGRLFSYSDTHRHRLGANYHQIPVNCPYATRCKNYQRDGPMNVDENQGFGYWLGNSLLESWQCRAIRDESVLA

>Xenopus.tropicalis_42_CAT_NP_001016716.1

MAGNKEKAPTQMNQWKEARGSQKPSVLTTGAGHPVGDKLNLLTAGPRGPLLVQDVVFTDEMAHFDRERIPERVVHAKGAGAFGYFEVTHDITQYCKANVFGKVGKRTPVAARFSTVAGEAGSPDTIRDPRGFAVKMYTEEGNWDLTGNNTPIFFIRDAILFPSFVHSQKRNPQTHMKDPDMVWDFWSLRPESLHQVSFLFSDRGIPDGHRHMNGYGSHTFKLVNCKDEAVYCKFHFKTDQGIRNLTLEKAEQLAASDPDYGIRDLYEAIAAGNYPSWTFYIQIMTFEQAEKFPFNPFDVTKVWPHGDYPLIPVGKMVLSRNPTNYFAEVEQLAFDPSNMPPGIEPSPDKMLQGRLFSYPDTHRHRLGANYLQLPVNCPYKARVANYQRDGPMCFTDNQGGAPNYYPNSFSAPEQQPQFREHRFRVSADVERYNSANDDNVTQVREFYLKVLNEEERQRLCENIVGHLKECQLFIQKRTVKNFSDVHPDYGSRIQALLDKHNAKCPKKESVGPNTQHSSYLTSKEKSNL

>Xenopus.tropicalis_41_CAT_NP_001011417.1

MAQQTQPILTTGAGIPAGDKLNVLTAGPRGPMLMQDVVFVDEMAHFDRERIPERVVHAKGAGAFGYFEVTHDITKYCRAKVFERVGKRTDVAVRFSTVAGEAGSADSVRDPRGFALKFYTDDGIWDLVGNNTPIFFIRDPMMFPSFIHSQKRNPQTHLKDPDTVWDFWSLRPETLHQVTFLFSDRGIPDGHRHMNGYGSHTFKLVNAEGKAVYCKFHYKTDQGIKNLSVEEADRLVVSDPDYGIRDLFQSIAKKNFPSWTMYLQVMTFEEAEKCPFNPFDLTKVWPHRDYPLIPVGKLVLNRNPENYFAEVEQIAFDPSNMPPGIEASPDKMLQGRLFSYPDTHRYRLGPNYLHLPVNCPRGVQVAHYQRDGPMCMFNNPSHMPNYYPNSFSSPRDDPKCKDSTFVAAGDVGRHDCSEEDNVSQVRMFYTQTLTEGERKRLCENLARHLSEAQIFIQERAVKNFTDVHPDYGARIKSLLDKYNGDGGEKEPLHNYIRYTECP

>Xenopus.tropicalis_40_CAT_XP_002934129.2

MAGRYGHQQTKIHEPELSLLTTASGVPIGDKRNSLTVGPRGPLLIQDAAFMEEMAHFNRERIPERVVHARGAGAFGYFEVTNDITQYCKAKVFSHVGKKTPIAVRFSTTTGELGSNDTVREPHGFAIKFYTEEGNWDLVGNHTPAFFIKDPILFPSLAHAQKKNPQTHLKDPNMFWDFVSLCPETLHEITHLFTDRGLPDGYRHMHGFGNHAFKLVNADGKPVYCKFHYKTNQGIKNLSSEQAKVIAGSDPDHALRDLLEAIAKGDYPSWTFSIQIMTFEQAEKMPFNPFDVTKVWYQKEFPLIPVGKLVLNRNPTNYFADVEQIALEPKNLVPGIEPSPDRVLQGRLFAYSDALRYRLGVNYTQIPVNRPQGVKVANYERDGHMVIDNQGNAPSYYPNSFGGPKDKAEYKEMVFHVSGDVDRYHNAETDDNFQVRKFYQKVLNDKQKQELCQNIASSLTGALQFIQDKSVKNFAAIDPDYGARVQKELDKLRAASQKKEESKPIFYQQKHY

>Xenopus.tropicalis_39_CAT_NP_001072167.1

MADKRDNAADQMKLWKESRGSQKPDVLTTGGGNPISDKLNLLTVGPRGPLLVQDVVFTDEMAHFDRERIPERVVHAKGAGAFGYFEITHDITKYSKAKVFEHIGKRTPIAIRFSTVAGESGSADTVRDPRGFAVKMYTEDGNWDLTGNNTPVFFIRDAMLFPSFIHSQKRNPQTHLKDPDMVWDFWALRPESLHQVSFLFSDRGIPDGHRHMNGYGSHTFKLVNAKDEAVYCKFHYKTDQGIRNLTVEEANRLSASDPDYGIHDLYESIAAGNYPSWSFYIQVMTFQQAEKFKFNPFDLTKIWPHGDYPLIPVGKLVLNRNPTNYFAEVEQLAFDPSNMPPGIEPSPDKMLQGRLFSYPDTHRHRLGPNYLQLPVNCPYRTRVANYQRDGPMCFTDNQGGAPNYYPNSFCAPENQPQVREHRFHVSADVARYNSADEDNVSQVRDFYVKVLSEEQRLRLCENIAGHLKDAQLFIQKRAVKNFTDVHPEYGARIQALLDKYNAEGAKKKTVKTYTQHSTYATAKDKANL

>Xestospongia.bergquistia_43_CAT_Xb.01372.mrna

MDTRDKAANQLTDFAKSKKSPDVLTTSHGHPVDNKTATLTAGPKGPVLIQDYIFLDEMAHFDRERIPERVVHAKGAGAFGYFEVTHDISKYCKAKVFSQIGKRCPIAVRFSTVGGESGSADTVRDPRGFAVKFYTEDGNWDLVGNNTPIFFIRDPILFPSFIHTQKRNPTTHLKDPDMFWDFISLRPETTHQVSFLFSDRGIPDGYRHMNGYGSHTFKLVNINGEPVYCKFHYKTDQGIKNLSVEKAGILAGDEPDYGIKDLYVAIATRNFPSWTLYIQVMTFEQAERYRWNPFDLTKIWPHKEFPLIPVGKLVLNRNPSNYFAEVEQVAFSPAHMVPGIEPSPDKMLQGRLFSYDDTHRHRLGVNYHQIPVNCPYATRTRNYQRDGFMTVDGNQGGAPNYFPNSFSGPLDNPSHAISKTIVTTGDVRKYNTKDDDNFSQVTNFWLHVLSTDEKTRLVSIIAGHLKNAADFIQKRAVRNFTEVHPEYGGRIAALLEKYKQIHLIPYVCHW

>Xestospongia.bergquistia_44_CAT_Xb.07299.mrna

MDTDPPRTKRAREMEGDNIGAYIPDNGGPHASIMFSLKEGKGALVRALKPFEELGINMTHIESRPSKKNPGVEYDFYIDCEVPKDIMENLLQQLKTTTVGVSVHARSPGKEEYPWFPRRVRDLDRFANQILSYGAELDSDHPGFTDSVYRARRKYFADIAFNYKHGQPIPRVEYTEAEVNTWKTVYRELTKLYPTHACQEHNHVFPLLIENCGYREDNIPQLQDVSDYLKECTGFTLRPVAGLLSSRDFLAGLAFRVFHSTQYIRHSSKPMYTPEPDVCHELLGHAPLFCDPTFAQFSQEIGLASLGAPDDYVEKLATLYWFTIEFGLCRQNGEMRAYGAGLLSSFGELQHCLSDVPEVRPFDPSKTSVQKYIITDMQQVYFLADSFSDAKEKLIEWGKHIPKPFTLHYDPYTQSVDVLDSASSLKKLASGIKYDMSVLAEAIVNVMAKRPKAFSQLEEYGSSQKRQEPLTTSHGHPVDFKTGVQTFGPRGPMLMQDFVYMDEMAHFDRERIPERVVHAKGAGAFGYFEVTHDITKYCSAKIFSEIGKKTPLVVRFSTVGGESGSADTVRDPRGFAVKFYTEDGNWDLVGNDTPIFFIRDPFLFPSFIHTQKRNPTTHLKDPDMFWDFISLRPETTHQVSFLFSDRGIPDGYRHMNGYGSHTFKLVNKDGEAVYCKFHYKTDQGIKNIPVDEAGRLAGSNPDYSIQDLYEAIATGNFPSWTLYIQVMTFKEAETHRFNPFDLTKVWPQKEFPLIPVGKIVLDRNPANYFADVEQAAFCPAHMPPGIEASPDKMLQGRLFSYSDTHRHRLGTNYHQIPVNCPYATKHRSYLRDGFMCVDGNQDGAPNYYPNSFNGPVDEGKHDIPIMKCPASDVARYNSADEDNFTQVGIFYREVLSEAERIRLTENIAGHLKNAKEFIQKRAVENFRKADPDYGERIAKLLEQYKTQEALTTSHGHPVDFKTGVQTFGPRGPMLMQDFVYMDEMAHFDRERIPERVVHAKGAGAFGYFEVTHDITKYCSAKIFSEIGKKTPLVVRFSTVGGESGSADTVRDPRGFAVKFYTEDGNWDLVGNNTPIFFIRDPFLFPSFIHTQKRNPTTHLKDPDMFWDFISLRPETTHQVSFLFSDRGIPDGYRHMNGYGSHTFKLVNKDGEAVYCKFHYKTDQGIKNIPVDKAGHLAGGNPDYSIQDLYEAIATGNFPSWTMYIQVMSFEEAETHRFNPFDLTKVWPQKEFPLIPVGKIVLDRNPANYFADVEQAAFCPAHMPPGIEASPDKMLQGRLFSYSDTHRHRLGTNYHQIPVNCPYATKHRSYLRDGFMCVDGNQDGAPNYYPNSFNGPVDEDKHDIPIMKCPASDVARYNSADEDNFTQVGIFYREVLSEEERARLTENIAGHLINAKDFIQKRAVDNFSAADPDYGGRIAKLLSVS

**Peroxiredoxin (PRX) sequences**

**>**PRX1/2_A.planci_1_gbr.1.257.t1

MSAGKCQITKPAPDFSGPAVMPSGEFKDIKLSDYKGKYLVFFFYPLDFTFVCPTELIAFSDRAKEFRDIGCELLACSCDSQYSHLAWTNTPRKKGGIGNMSIPLLADKSCRIAKDYGVLIEEDGVSFRGLFIIDNKGILRQITINDLPVGRSVDETLRLVQAFQFTDKHGEVCPAGWRPGAETIKPDVKKSQDYFSKN

>PRX3_A.planci_2_gbr.17.59.t1

MQMYADRRGMRGLNFERSFLSGAEGMCPPKTSSCPCNIKGTTNFKLCVQNLQTAGSPVEHKLNVGVQHAAPSFVGTAVVNGDFKTVRLEDYRGKYLVLFFYPLDFTFVCPTEIIAFSERASEFHQLNTEVVGVSVDSHFSHLAWINTPRKEGGLGPMNIPLLSDFKKEISKDYGVLLYEEGVALRGLFIIDPDGIVRHTSVNDLPVGRSVDETLRLVKAFQFVAKHGEVCPAGWTPDSPTIKPDPHGSKTYFEEVNK

>PRX4_A.planci_4_gbr.67.9.t1

MALVMSCASRFVWGFLLVVAVSSLGGADEACYSYGGGHVYPGEGLRSSEHAVHWSKVQISKPAPTFEGTAVVNGEFKELKLTDYKGKYLVLVFYPLDFTFVCPTEIIAFSDRISEFKEINAEVVAVSVDSQFTHLAWINTPRKQGGLGPIQIPLLSDLTHQISKDYGVLLEDLGHTLRGLFIIDGNGVLRQITMNDLPVGRSVDETLRLVQAFQYTDKNAEVCPAGWKPGSDTIIPNPEDKLKYFSKLPENDD

>PRX4_B.floridae_1_gi|260782386

MAGLQRFVLSVALVLTACWAAEDQKDEICFQQPQRGTLYPGMQWPGSTEHALTGSKAQISKPAPDFQGTAVVNGKFEEIKLSDYKGKYLVFFFYPLDFTFVCPTEIIAFNERVEEFRKVNTEVVGVSVDSQFTHLAWINTPRKAGGLGPMNFPLLSDLTHKISRDYGVLLEDVGHTLRGLFIIDDKGILRQITMNDLPVGRSVDETLRLVQAFQYTDQHGEVCPAGWTPGADTIIPNPNDKLKYFEKANKDL

>PRX4_B.floridae_2_ gi|260782530

MNVLYLCFQKLPLYFPVSKPAPDFQGTAVVNGKFEEIKLSDYKGKYLVFFFYPLDFTFVCPTEIIAFNERVEEFRKVNTEVVGVSVDSQFTHLAWINTPRKAGGLGPMNFPLLSDLTHKISRDYGVLLEDVGHTLRGLFIIDDKGILRQITMNDLPVGRSVDETLRLVQAFQYTDQHGEVCPAGWTPGADTIIPNPNDKLKYFEKANKDL

>PRX1/2_B.floridae_6_gi|260821348

MSAGKAKLQHPAPNFESTAVLPSGEFGTIKLSDYKGKYLIIFFYPMDFTFVCPTEIIAFSDRVEEFKKINCEVLACSTDSQFSHLAWTNTPRKQGGLGQMKIPLMADKAMTISRDYGVLMEDAGIAFRGLFIIDDKGTLRQITINDLPVGRSVDETLRLVQAFQFTDKHGEVCPAGWKPGADTIKPDVKDSKEYFSKQ

>PRX3_C.teleta_3_CapteP17785

MASFLRSLSRVGSSVCRVARAGNARQFSTGSPLLAARVQHAAPFFKGQAVVDGQFQEVNLEDFKGKYLVLFFYPLDFTFVCPTEIIAFSDRINEFKELNAEVVGVSTDSHFSHLAWINMPRKQGGLGGLQYPLLSDFSKNISKDYGVLVENAGIALRGLFLIDPTGTVRQVTINDLPVGRSVDETLRLIKAFQFVEKHGEVCPANWTSESETIKPNPTDSLEYFGKVNE

>PRX1/2_C.teleta_5_CapteP180369

MSKRSGDDLQAQPAPKRLNASDRSELTSGQTARLVKSACIAMSELKLTKPAPAFSGTAIVDGDFKEISISDYKGKYLVFFFYPLDFTFVCPTEIIAFSDRVEEFRSINCEVVACSTDSAFSHLAWTQQPRNKGGLGNMNIPLLADKTLDIATRYGVLKEDEGIAFRGLFIIDDKGNLRQVTINDLPVGRSVDEVLRLVQAFQFTDKHGEVCPAGWKPGAATMKPDTKESKSYFQKNN

>PRX4_C.teleta_7_CapteP228025

MIVFAFMSVLLVSCVVSEEACHSFAEGNVYPHFNRYGGDHSLVYSKAQISKPAPEWKGTAVINGEFKDLSLSDYKGKYLVFFFYPLDFTFVCPTEIIAFSDRVKEFQAINAEIVAASVDSPFTHLAWMNTPRNQGGLGKMNIPLLSDLSHKISKDYGVYLENVGHTLRGLFIIDPKGILRQITMNDLPVGRSVDETMRLVQAFQYTDQHGEVCPAGWKPGSDTIIPDPSDKLKYFKKVNA

>PRX3_L.anatina_1_g10990.t1

LFTRIGCHIKRNLQTSLAPKLVPRVGVITGSQRYLHVTPNRFEAKVQQPAPDFKGEAAVDGAIKELNLTDYRGKYLILLFYPQDFTFVCPTELIAFSDRIAEFHAINTEVIGVSTDSVFSHLAWINTPKKEGGLGECKYPLLSDFKKTIATDYGVLLEEKGVALRGLFLIDPNGLVRHMTVNDLPVGRSVDEALRTVQAFQFVEEHGEVCPANWQPDSPTITPTLEGSREYFTQVN

>PRX4_L.anatina_2_g13237.t1

MAKYCLVLLLLIAHQCLSADDEACQVFGGGHVYPQETMKSAGHALHWSKAQISKAAPHFEGTAVVDGEFKELKLTDFKGKYLVFFFYPLDFTFVCPTEIIAFSDRVEEFRAINAEVVACSVDSQFTHLAWINTPRNQGGLGPLKIPLLSDLTHKISKDYGVYLEDNGHTLR

>PRX1/2_L.anatina_3_g18858.t1

MSEAIFVEVRRGGYMTVGKTLDGKEPEPKVAVTHEVVEMQKAGLKLRTNKSAQVSSASSSASASSKSRGRKNETMNTKKAAEKSGSGGSRHENHSPATPAAPCTCNCASELAVKMTELLGHLSLERTPSSSQSANMTSTKEGLRIGKPAPNFSGTAVIDGDFKTVKLDDYRGKYLVFFFYPLDFTFVCPTEIIAFSDRSEEFRSIGCEVVACSTDSHFSHLAWVNTPRKKGGLGQMKIPLLADKTMEIAKAYGVLKEEDGVTFRGLFIIDGQGILRQVTVNDLPVGRSVDETLRLVQAFQFTDKHGEVCPAGWRPGADTMKPDPKGSQTYFEKTNK

>PRX1/2_L.anatina_6_g32086.t1

MAWERQDNSWVKRTPSSSQSANMTSTKEGLRIGKPAPNFSGTAVIDGDFKTVKLDDYRGKYLVFFFYPLDFTFVCPTEIIAFSDRSEEFRSIGCEVVACSTDSHFSHLAWVNTPRKKGGLGQMKIPLLADKTMEIAKAYGVLKEEDGVTFRGLFIIDGQGILRQVTVNDLPVGRSVDETLRLVQAFQFTDKHGEVCPAGWRPGADTMKPDPKGSQTYFEKTNK

>PRX4_N.vectensis_2_EDO38694

MATGVLFVFCFALFAFCSSANKVEDQDQCRVYAGGQVYPERTKISEHAVHWSKAQISKPAPFWEGTAVVNGEFKELKLSDFEGKYLVFFFYPLDFTFVCPTEIIAFSDRIEEFRAINTEVVGCSVDSVFTHLAWINSPRKEGGLGNLKYPLLSDINHQVSKDYGVLLENEGHTLRGLFIIDDKGVLRQITMNDLPVGRSVDETLRLVQAFQYTDKHGEVCPAGWKPGKDTIIPDPTQKKKYFEKQAQKEDL

>PRX4_N.vectensis_4_EDO48197

MSKTAIQKPAPAFSGTAVNKHGEFIDLKLSDYKGKYVVLFFYPLDFTFVCPTEIIAFSDRVDEFKAINCEVIACSVDSEYSHLAWTNVPRKKGGIGNINIPILSDLTKQISKDYGVLLEDQGVALRGLFIIDDKGILRQITINDLPVGRSVDETLRLIQAFQFTDKHGEVCPAGWRPGADTIIPEPQKSSSYFSKQ

>PRX2_X.tropicalis_2_gi|45360655

MSCPVGPGVRAVKTHIGQPAPAFKATAVVNGEFKDIQLSDYLGKYVVLFFYPLDFTFVCPTEIIAFSDHAGDFSKINCQLIAVSVDSQFTHLAWTNVPRKEGGLGPINIPLVSDLTHSIAKDYGVLKEEDGVAYRGLFIIDGKGNLRQITINDLPVGRSVEETLRLVQAFQYTDQHGEVCPAGWKPGSSTIKPNVKDSKEFFSKEY

>PRX4_X.tropicalis_4_gi|55742316

MAVLLRHYLRGSPVLGLCLLLLSAAAVTCEPQEEKEQPQGRPGRAAPDGECHFYAGGQVYPGEATRVPVSDHSLHLSKAKISKPAPYWEGTAVINGEFKELKLTDYKGKYLVFFFYPLDFTFVCPTEIIAFGDRIEEFRSINTEVVACSVDSQFTHLAWINTPRKQGGLGPMKIPLLSDLTHQISKDYGVYLEDQGHTLRGLFIIDDKGVLRQITMNDLPVGRSVDETLRLVQAFQYTDTHGEVCPAGWKPGSETIIPDPAGKLKYFHKQH

>PRX1/2_X.tropicalis_5_gi|58331972

MSSGNAKIGHPAPDFTAKAVMPDGQFKDLKVSDYKGKYVVFFFYPLDFTFVCPTEIIAFSDRVEEFKKLNCEVIGASGDSHFCHLAWISQPRKEGGLGKMNIPLVSDVQHTIAKDYGVFEEKEGVSFRGLFIIDEKGILRQITINDLPVGRSVDETLRLVQAFQFTDKYGEVCPAGWQPGSDTIKPDVKKSKEYFNKQK

>PRX3_X.tropicalis_6_gi|71896087

MAACGRLLASWVRRSGRLAGSPVLRNAAAATPSRCAIHKLQFSTSSVRFLPAVTQHAPHFKGTAVVNGEFKELSLEDYKGKYLVLFFYPLDFTFVCPTEIVAFSNKANEFHDVNCEVVAVSVDSHFCHLAWTNTPRKSGGLGQMNIPLLSDLNKQISRDYGVLLETPGIALRGLFIIDPNGIIKHMSVNDLPVGRSVEETLRLVKAFQFVETHGEVCPANWTPDSPTIKPSPEGSKDYFEKVH

>PRX1/2_D.rerio_1_ref|NP_001002468.1

MSAGNAKIGQPAPQFKATAVVDGQFKDIQLSDYRGKYVVLFFYPLDFTFVCPTEIIAFSERAAEFRKIGVELIAASTDSHFSHLAWINTPRKQGGLGSMNIPLVADLTQSISRDYGVLKEDEGIAYRGLFVIDDKGILRQITINDLPVGRSVDETLRLVQAFQHTDKYGEVCPAGWKPGSDTIVPDVQKSKEFFSKQ

>PRX3_D.rerio_2_ref|NP_001013478.3

MAATIGRLLGASARGAAVCGLKTLVPRNGASVIRAPQPLACIAAQKACFSISAARWAPAVTQAAPHFKGTAVINGEFKEISLGDFKGKYLVLFFYPLDFTFVCPTEIVAFSDKANEFHDVNCAVVGVSVDSHFTHLAWTNTPRKSGGLGKIQIPLLADLTKQVSRDYGVLLEGPGIALRGLFIIDPNGIVRHMSVNDLPVGRSVEETLRLVKAFQFVETHGEVCPASWTPKSPTIKPTPDGSKEYFEKVN

>PRX1/2_D.rerio_3_ref|NP_001013489.2

MAAGNAHIGKPAPDFTAKAVMPDGQFGDVRLSDYKGKYVVLFFYPLDFTFVCPTEIIAFSDAAEEFRKINCEIIGASVDSHFCHLAWTKTPRKQGGLGPMNVPLVADTLRSISKDYGVLKEDEGIAYRGLFIIDDKGILRQITINDLPVGRSIDETLRLVQAFQFTDKHGEVCPAGWKPGKDTIKPDVNQSKDFFSKQN

>PRX4_D.rerio_5_ref|NP_001082894.1

MDVSRCVKTPREWLGVLWALLLLSESVVCDGANGKREQECYNYAGGHVYPGEAFRVPVSDHSLHLSKAKISKPAPHWEGTAVINGEFKELKLSDYKGKYLVFFFYPLDFTFVCPTEIIAFSDRVHEFQAINAEVVACSVDSQFTHLAWINTPRKQGGLGPMKIPLLSDLTHQISKDYGVFLEDQGHTLRGLFIIDGKGVLRQITMNDLPVGRSVDETLRLVQAFQYTDKHGEVCPAGWKPGSDTIIPDPAGKLKYFDKLN

>PRX1_H.sapiens_2_PRDX1.3

MSSGNAKIGHPAPNFKATAVMPDGQFKDISLSDYKGKYVVFFFYPLDFTFVCPTEIIAFSDRAEEFKKLNCQVIGASVDSHFCHLAWVNTPKKQGGLGPMNIPLVSDPKRTIAQDYGVLKADEGISFRGLFIIDDKGILRQITVNDLPVGRSVDETLRLVQAFQFTDKHGEVCPAGWKPGSDTIKPDVQKSKEYFSKQK

>PRX1_H.sapiens_3_PRDX1.4

MSSGNAKIGHPAPNFKATAVMPDGQFKDISLSDYKGKYVVFFFYPLDFTFVCPTEIIAFSDRAEEFKKLNCQVIGASVDSHFCHLAWVNTPKKQGGLGPMNIPLVSDPKRTIAQDYGVLKADEGISFRGLFIIDDKGILRQITVNDLPVGRSVDETLRLVQAFQFTDKHGEVCPAGWKPGSDTIKPDVQKSKEYFSKQK

>PRX2_H.sapiens_5_PRDX2.2

MASGNARIGKPAPDFKATAVVDGAFKEVKLSDYKGKYVVLFFYPLDFTFVCPTEIIAFSNRAEDFRKLGCEVLGVSVDSQFTHLAWINTPRKEGGLGPLNIPLLADVTRRLSEDYGVLKTDEGIAYRGLFIIDGKGVLRQITVNDLPVGRSVDEALRLVQAFQYTDEHGEVCPAGWKPGSDTIKPNVDDSKEYFSKHN

>PRX3_H.sapiens_7_PRDX3

MAAAVGRLLRASVARHVSAIPWGISATAALRPAACGRTSLTNLLCSGSSQAKLFSTSSSCHAPAVTQHAPYFKGTAVVNGEFKDLSLDDFKGKYLVLFFYPLDFTFVCPTEIVAFSDKANEFHDVNCEVVAVSVDSHFSHLAWINTPRKNGGLGHMNIALLSDLTKQISRDYGVLLEGSGLALRGLFIIDPNGVIKHLSVNDLPVGRSVEETLRLVKAFQYVETHGEVCPANWTPDSPTIKPSPAASKEYFQKVNQ

>PRX4_H.sapiens_8_PRDX4.2

MEALPLLAATTPDHGRHRRLLLLPLLLFLLPAGAVQGWETEERPRTREEECHFYAGGQVYPGEASRVSVADHSLHLSKAKISKPAPYWEGTAVIDGEFKELKLTDYRGKYLVFFFYPLDFTFVCPTEIIAFGDRLEEFRSINTEVVACSVDSQFTHLAWINTPRRQGGLGPIRIPLLSDLTHQISKDYGVYLEDSGHTLRGLFIIDDKGILRQITLNDLPVGRSVDETLRLVQAFQYTDKHGEVCPAGWKPGSETIIPDPAGKLKYFDKLN

>PRX1/2_C.elegans_1a_WBGene00006434

MSKAFIGKPAPQFKTQAVVDGEFVDVSLSDYKGKYVVLFFYPLDFTFVCPTEIIAFSDRAEEFKAINTVVLAASTDSVFSHLAWINQPRKHGGLGEMNIPVLADTNHQISRDYGVLKEDEGIAFRGLFIIDPSQNLRQITINDLPVGRSVDETLRLVQAFQFVEKHGEVCPAGWTPGSDTIKPGVKESQEYFKKH

>PRX1/2_C.elegans_1b_WBGene00006434

MYRQMSKAFIGKPAPQFKTQAVVDGEFVDVSLSDYKGKYVVLFFYPLDFTFVCPTEIIAFSDRAEEFKAINTVVLAASTDSVFSHLAWINQPRKHGGLGEMNIPVLADTNHQISRDYGVLKEDEGIAFRGLFIIDPSQNLRQITINDLPVGRSVDETLRLVQAFQFVEKHGEVCPAGWTPGSDTIKPGVKESQEYFKKH

>PRX1/2_C.elegans_1c_WBGene00006434

MSLAPKMSKAFIGKPAPQFKTQAVVDGEFVDVSLSDYKGKYVVLFFYPLDFTFVCPTEIIAFSDRAEEFKAINTVVLAASTDSVFSHLAWINQPRKHGGLGEMNIPVLADTNHQISRDYGVLKEDEGIAFRGLFIIDPSQNLRQITINDLPVGRSVDETLRLVQAFQFVEKHGEVCPAGWTPGSDTIKPGVKESQEYFKKH

>PRX3_C.elegans_2_WBGene00011110

MFSSAVRALCRTVPTVATRQLSTSRALLSLRPLGPKNTVPAFKGTAVVDGDFKVISDQDYKGKWLVMFFYPLDFTFVCPTEIIAYGDRANEFRSLGAEVVACSCDSHFSHLAWVNTPRKDGGLGDMDIPLLADFNKKIADSFGVLDKESGLSYRGLFLIDPSGTVRHTTCNDLPVGRSVDETLRVLKAFQFSDKHGEVCPADWHEDSPTIKPGVATSKEYFNKVNK

>PRX1/2_C.intestinalis_1_ENSCINT00000006141

MSAGKACIQKSAPDFTATAVVNGDFRDISLSEYKGKYVVLFFYPLDFTFVCPTEIIAFSDRVSEFRDIGCEVLACSTDSHFSHLAWTNIPRKKGGIGNMKIPLIADKNCAISKDYGVLMEGSGIAFRGLFIIDTMGILRQITINDLPVGRSVDETLRLVKAFQFTDQHGEVCPAGWKPGDDTIKPDVQDSQKYFSKQ

>PRX4_C.intestinalis_2_ENSCINT00000013246

LQSTKAQISKPAPDWEGTAIVDGEIKTIKLGDYKGKYLIFFFYPLDFTFVCPTEIIAFSDRVAEFKKINAEVVAASVDSHFTHLAWLNTHRSEGGLGKLNIPLLSDLTHKISRDYGVLLEDLGHTLRGLFIIDPKGILRQITMNDLPVGRSVDETLRLVQAFQHTDEHGEVCPAGWEPGKDTIIPDPKDKLKYFHKTSKAKG

>PRX3_C.intestinalis_4_ENSCINT00000031576

MFINRLVHGFRRTHTLQSSLNPWISKWNPNSASISTSCRSMVAQVTQPAPPFKGMSVVEGKFKEISLEDYKGKYLVLFFYPLDFTFVCPTEIISFSDKSPEFEKLDTVVVGASVDSHFSHLAWINTPRKQGGLGEMKIPLLSDLTKNISRDYGVLLENAGIALRGLFIIDPSGTIRHASVNDLPVGRSVDEVLRLVQAFQFVDKHGEVCPASWTPGSKTIKPSVDGSKTYFEEAN

>PRX3_D.melanogaster_5_FBgn0038519

MSFVARSLIRNVPLMGKAILSQQKQIAARLLHQTAPLAAVRVQQPAPDFKGLAVVDNSFQEVKLEDYRGKYLVLFFYPLDFTFVCPTEIVAFSERIKEFHDINTEVLGVSVDSHFSHLTWCNVDRKNGGVGQLKYPLLSDLTKKISADYDVLLDKEGISLRGTFIIDPNGILRQYSINDLPVGRSVDEVLRLIKAFQFVEQHGEVCPANWNPNSNPATIKPDVEESKKYFSKHG

>PRX4_D.melanogaster_7_FBgn0040308

MSKYLSVLLLSAALVGAAKPEDNESCYSFAGGSVYPDQPKGDHQLQYTKAVISKPAPQFEGTAVVNKEIVKLSLSQYLGKYVVLLFYPLDFTFVCPTEIIAFSDRIAEFKKIKTEVIGVSVDSHFTHLAWINTPRKEGGLGDVKIPLLSDLTHKISKDYGVYLESSGHALRGLFIIDQTGVLRQITMNDLPVGRSVDETIRLVQAFQYTDTHGEVCPAGWRPGADTIVPNPEEKTKYFAKNN

>PRX1/2_D.melanogaster_8_FBgn0040309

MPQLQKPAPAFAGTAVVNGVFKDIKLSDYKGKYLVLFFYPLDFTFVCPTEIIAFSESAAEFRKINCEVIGCSTDSQFTHLAWINTPRKQGGLGSMDIPLLADKSMKVARDYGVLDEETGIPFRGLFIIDDKQNLRQITVNDLPVGRSVEETLRLVQAFQYTDKYGEVCPANWKPGQKTMVADPTKSKEYFETTS

>PRX4_M.leidyi_1_ML04054a

MMSTLILTLLVGITLASDEACPMNGFFGYGPKGYDHALGVVKARISKPAPDFTAAAVVNGKFEDITLSSFKGKYVVLFFYPLDFTFVCPTEIIAFNDRVGEFKKINTEVIAASVDSKFTHLAWINTPRNKGGLGDMDIPIISDLTRQISRDYGVMLEDEGHTLRGLFIIDDKGILRQITMNDLPVGRSVDETLRLVQAFQYTDQHGEVCPANWTPGKDTLKPDPEGKLEYFEKNCNKKSGEL

>PRX3_M.leidyi_3_ML1541105a

MYITLTFTHSLIKPITNDTPTFLAAMFSSLSRVGSRLVQTAARRALAAPSLTRHVASIPNPAPAFTAQAVMPNGTFQEVSLSNYEGKYVILFFYPLDFTFVCPTEITAFSDRAAEFQELGCELLACSVDSHFSHLAWMKQSRKEGGLGEMKIPILADLNKTIAKDYGVLLEPVGIALRGLFIIDPAGNLRHITVNDLPIGRSVDETLRTLRAIQFVEEHGEVCPANWQPGKSTIKPGVDSSKEYFEKEN

>PRX4_M.leidyi_4_ML216312a

MSKAFITKPAPDFDLEAVLPTEEFERVKLSDYKGKYLVLFFYPMDFTFVCPTEIIAFSDRIEEFRKLDCEVVAASCDSVYCHYAWCCQSRKAGGLGKMNIPLLADVTHSLSKDYGVYLEDEGVSLRGLFIIDGKGILRQSTINDLPVGRSVDEVLRLLQAFQFTDKYGEVCPAERCNPQILPQGRPARAQQTTSLG

>PRX4_S.purpuratus_1_SPU_006211

MIRILSLILAVSTLVAAEEACHSFAGGHVYPGETPRSTAHAVHWSKVQISKPAPVFEGTAVVEGEFKAMKLSDFAGKYLVLVFYPLDFTFVCPTEIIAFSDRVDEFRAINTEVVAISVDSQFTHLAWISTPRTQGGLGPIKLINTPRTQGGLGPIKLPILSDLTHQIAKDYGVLLEDLGHTLRGLFIIDDKGVLRQITMNDLPVGRSVDETLRLVQAFQYTDKHGEVCPAGWKPGSDTIIPNPADKLKYFAKQPNGGNA

>PRX3_S.purpuratus_3_SPU_022529

MSLFHQICRLGVTGARATRFCSGSFSTVCCRATVPKPLASQLLPPYAAAAELGQRAWSCIQRQLSTSCQLNVAIQEPAPDFEGTAVIDGQFKEIKLSDYKGKYLVLFFYPLDFTFVCPTEIIAFSDRADEFGAINTEVVAASIDSHFSHLAWINTPRKQGGLGPMKIPLLSDMKKQIAEDYGVLLKDAGVALRGLFLIDPEGVVRHMSINDLPVGRSVDETLRLVKAFQFVAEHGEVCPAGWTPDSETIKPDPEGSKTYFEKVN

> PRX4_A.queenslandica _4_Aqu3.1.40005_001 MAFLLMLSLLFTLTLTGAEPGAATFEKCPLLNLNGLVFPDYSSRPVEHALHWSKAQIAKPAPDWNGTAVVGAAFKELRLSDFKGKYLVFFFYPLDFTFVCPTEITAFSDRVGEFKAINTEVVACSVDSKYTHLAWIKTPRDKGGLGELNIPLLSDITKQISRDYGVLLEDEGISLRGLFIIDARGILRQITMNDLPVGRSVDETLRLVQAFQYTDQYGEVCPAGWKPGEQTIVPDPEGAQKYFKKCNDKN

>PRX4_E.muelleri_1_Em0023g430a

MSLTTCLFLVSLGLLARASNDDQCYEYYGGLVYPQGWRRTVEHSMHWSKTQISKPAPDWNGTAVVNRDFVELKLSDFKGKYLVFFFYPLDFTFVCPTELLAFSDRLDEFKALNTAVVACSVDSKYTHLAWLNTKRAEGGLEGLRIPLLADLTKQISKDYGVLLEDLGHTLRGLFIIDDRGILRQITMNDLPVGRSVDETLRLVQAFQYTDVKGEVCPAGWKPGEKAIIPDPVAKKEYFTYKENCKKEL

>PRX1/2_E.muelleri_4_Em0011g118a

MSAPVARVQGKAPHWSGTAVINGDFEEISLDKYAGEYLVFFFYPNDFTFVCPTEIIAFSNRLGEFRKNKCNIVACSCDSQYVHLAWVETPTSKGGVGQLDIPLLADVTKKISRDYGVLLEDQGVSLRGLFIIDGKGTIRHMSINDIQVGRCVDEVLRLVRAFQYTDIHGEVCFLTNGYHD

>PRX4_O.carmela_1_m.307493

MGSTLHIFVLLCCIGLARAAEHKDASEACYTYAGGQVYPGERMRGGEHTLSWSKTQISKPAPYFEATAVVNGEFKEIKLSDYKGKYLVFFFYPLDFTFVCPTEIIAFSDRAEEFRAINAEVVACSVDSPFTHLAWINTPRKEGGLGKVDIPILSDMTHQISKDYGVLLENQGHTLRGLFIIDDKGTLRQITMNDLPVGRSVDETLRLVQAFQYTDNHAEVCPAGWKPGSDTIIPDPNKKLTYFGKQDPDQQ*

>PRX1/2_O.carmela_2_m.3083

RNCIDSDCAMFASLRRAFSATARRMSKAFVQREAPAWSGTAVMPDGTFKDLKLSDYRGKYLVFFFYPLDFTFVCPTEIIAYSERADEFKKRDCEVIACSVDSEFSHLAWTNTPRKKGGLGPMKIPLLSDLTKSISRDYGVLLEDAGCSLRGLFIIDGKGVLRQITVNDLPVGRSVDETLRLVEAFQFTDENGEVCPANWKPGDDTIKPDPSGSKDYFSKQ

>PRX4_S.ciliatum_1_scpid69360|

MKSFLLLCFVATTLLGVCNGAGKDADCPYRTYAGGEVYPMEWPRSHDHSLSYSKAKIGQPAPFWSGTAVTPAGKFQDMNLTDFKGKYLVMLFYPLDFTFVCPTEILAFSDRLAEFRQINAEIVAISVDSKFTHLAWTKMSRREGGLGKVHIPLLSDLTHQISKDYGVFLEKEGHTLRGLFIIDNHGVLRQITMNDLPVGRSVDETIRLIQAFRYTDEHGEVCPAGWKPGAATIVPDPEGKKAYFEKANPGN

>PRX1/2_S.ciliatum_2_scpid91469|

TVQGACSASFRVPFAPHVGSTRSGLFLAPLVYLRVENNWRFIMSGRRFLFSLQNLSGCVRPVFQTRLHHCSTMASRAQIQQAAPVFKGQAVVDGQFKEISLSDYAGKYVVFFFYPLDFTFVCPTEIVAFSDRVEEFRKINCEVVACSVDSHFSHLAWTKVPRKQGGLGDMNIPLLADLTKGISRDYGVLLEDQGIALRGLFIIDNKGVLRQSTVNDLPVGRSVDETLRLVKAFQFTDEHGEVCPANWTPGKNTIKPSVDASQEFFSKAE

>PRX1/2_S.ciliatum_3_scpid56902|

MGTGAARLKRTGRNAGARPESREGDSARILPVRDGPALRWYEIEPGKQIIPAEIPFSKDAVARKSRKPTVEKEVEPDSVSTTTTYACQVTQTSVDGVKRLSVIDERTDNLQQVSKRSSSSRGQAESQEAPTRIDTEATRRSSIMFKTPETHPVLPSPKPKPSEDKSGGQSRSQSLTSMLPMLSNLPTINPSAMVGSVDSRFPVAAMPRARVQKSAPAFKGKALMPNGIFRTISLEQYVGKYLVLFFYPGDFTFVCPTEIIEFSERCEEFKNLHCEIVACSVDSEYCHKAWTGVPRSKGGLGKMNIPILADPTRQIAQAYDVLVEDDGVALRGLFIIDPKGIVRQITVNDLEVGRNVDEVKRLVEAFQYSDKHGSVCPQGWTAGKKTIKPDPQGALEFFETVPI

>PRX4_T.wilhelma_1_Twilhelma_g21185.t1

MRSLTFLLCLLATAALVAAQSQEQCAVYYGGLVFPEGARRTVEHGMHWSKTQISKPAPDWNGTAVINGEFKELKLSDFRGKYLVFFFYPLDFTFVCPTEILAFSDRVEEFKAINADVVACSVDSKYTHFAWISMPREEGGLKGLKIPLLSDLTKQISKDYGVLLEDAGHTLRHVVDVAIHECVRGLFIIDHKGILRQITMNDLPVGRSVDETLRLVQAFQFTDQKGEVCPAGWKPGEMTIKPDPVGKNEYFSKAFCADPNPPKPTPTSDSASDDEEDDSD

>PRX4_T.wilhelma_2_Twilhelma_g21185.t2

MHWSKTQISKPAPDWNGTAVINGEFKELKLSDFRGKYLVFFFYPLDFTFVCPTEILAFSDRVEEFKAINADVVACSVDSKYTHFAWISMPREEGGLKGLKIPLLSDLTKQISKDYGVLLEDAGHTLRHVVDVAIHECVRGLFIIDHKGILRQITMNDLPVGRSVDETLRLVQAFQFTDQKGEVCPAGWKPGEMTIKPDPVGKNEYFSKAFCADPNPPKPTPTSDSASDDEEDDSD

>PRX4_X.bergquistia_1_Xb.09444.mrna

MAGEDLCHFLFLFSSLLMVTVGVESGSTYEQCPILNLNGLVFPEYSRRPVEHALHWSKAQIAKAAPDWNGTAVVGDPPSFKDLKLVDFKGKYLVFFFYPLDFTFVCPTEIIAFSDRIKEFKDINTEVVACSVDSKYTHLAWIKQPRDKGGLGHLEIPLLSDITKQISRDYGVLLEDEGISLRGLFIIDPRGILRQITMNDLPVGRSVDETLRLVQAFQYTDKHGEVCPAGWKPGEKTIIPDPEGSQKYFEQCNNN

>Prx5_B.floridae_3_gi|260783931

MPIKVGDKLPGIDLYENTPGNKVNVSELFAGKKGILFAVPGAFTPGCSKTHLPGFVSQAGDLKAKGVQVIACVSVNDPFVMEAWGKDQKAEGKVRMLADSAAEFTKAIGLELDATGLLGNIRSKR

>Prx5_B.floridae_8_gi|260837161

MMLIPVALGSVTHRLPSAIGALRTIFTATAYSMPIKVGDKLPGVDLYENTPGNKVNVSELFAGKKGVIFAVPGAFTPGCSKTHLPGFVSRAGDLQAKGVEVIACVSVNDPFVMEAWGRDQKAEGKVRMLADTGAEFTKAIGLDLDATAILGNIRSKRYSMLVEDGEVKQLNVEPDGTGLSCSLAEELKL

>Prx5_C.teleta_1_CapteP116279

MSLSIGQPLPDATLFLATNDGPQTSSVKEIFSAGRIAAFVVPGAFTPACHRNHLPGYLKLRDELLAKGIDKIVCLAVNDAFVLSAWARETAAVGLITMISDGNGDFTRAAGMEIDLSDHGIGQRSRRYSFVTDKGIVTHLNVE

>Prx5_C.teleta_2_CapteP147986

MEGTPSDKVSLSDLFKGKKGIVFAVPGAFTPGCSKTHLPGYVEMFDQLKAKGAEVIACVAVNDPFVMAAWGKAHNAEGKIRMLADPAGEFTKAVDMEIDLSSALGNVRSQRYSLVIEDGKVTHVNAEPDGKGLTCSLVNEVVSQL

>Prx5_C.teleta_6_CapteP21371

MRAIQVGDPLPSVPLFEKFPGNEVLLADLIGTKKAVVFAVPGAFTPGCTRVHLPGYVDAYDKLRSKGIEVIACIAVNDPFVVTAWGNAAGATGKIRMLSDPRAEFTKAIGMDFDARPLLGTVRSKRYSMLVEQGKVVQLFAEPDGGGLTCSLAPNLLSRL

>Prx5_L.anatina_5_g25263.t1

MRCCGKVLTQVNRQFIRGSRTFFTATVNSMPIKEGDRLPNIEVLEKTPNTKVNLGDLFKGKKGVLFGVPGAFTPGCSKTHLPGYVQNFDKLKAKGMDVVACIAVNDPFVMDAWGENQGANGKIRMLADYKGEFAKAADLEKDLTGALGSVRCKRFSMVVEDGVVKKLNVEPDGTGLTCSLADNVLSQL

>Prx5_N.vectensis_1_EDO37274

MPIKVGEALPSIKVMEGTPKDTVDVASLFKGKKGILFAVPGAFTPGCSKTHLPGYVADFDKIKSKGVDVVACIAVNDPFVMSAWGEANGCQGKIQMLADVHGEFTKAVDLELDATPFLGNIRSKRYAMLVEDGVVKQLHVEPDGTGLTCSLSNSILSQL

>Prx5_X.tropicalis_1_gi|163915087

MSVKVGDQLPNVTVYEGGPGNKVSIRDVFANKKGVLFGVPGAFTPGCSKTHLPGYVAQAAELKSRGAAVIACISVNDIFVMSEWAKAYDAEGKVCMLADPCGDFAKACGLLLDKKELSELFGNQRCKRFSMVVEDGKVKAINVEEDGTGLTCSLAGNIMSQL

>Prx5_D.rerio_4_ref|NP_001019577.1

MPIKVGQRLPAVEVQEEDPGNSLSMAELFSCKRGVLFGVPGAFTPGCSKTHLPGFIQMAGELRAKGVDEVACISVNDVFVMSAWGKQNGADGKVRMLADPTGAFTKAVDLVLNNAQLIPVLGNLRSQRYAMLIENGVVTKLSVEPDGTGLTCSLASNFLAEV

>Prx5_H.sapiens_12_PRDX5

MGLAGVCALRRSAGYILVGGAGGQSAAAAARRYSEGEWASGGVRSFSRAAAAMAPIKVGDAIPAVEVFEGEPGNKVNLAELFKGKKGVLFGVPGAFTPGCSKTHLPGFVEQAEALKAKGVQVVACLSVNDAFVTGEWGRAHKAEGKVRLLADPTGAFGKETDLLLDDSLVSIFGNRRLKRFSMVVQDGIVKALNVEPDGTGLTCSLAPNIISQL

>Prx5_D.melanogaster_6_FBgn0038570

MRVLSCKFLGRVVNSALPQQIISLRSLSKTSAAMVKVGDSLPSVDLFEDSPANKINTGDLVNGKKVIIFGVPGAFTPGCSKTHLPGYVSSADELKSKQGVDEIVCVSVNDPFVMSAWGKEHGAAGKVRLLADPAGGFTKALDVTIDLPPLGGVRSKRYSLVVENGKVTELNVEPDGTGLSCSLANNIGKK

>Prx5_A.queenslandica_5_Aqu3.1.38453_001

MFSRHFSSRLLFFTRGLRTTASLKMPIQVGQTLPSIELHEGTPKDKVNILELFKGKKGILFAVPGAFTPGCSQTHLPGYVNDYLKLKAKGFEVIACVSVNDAFVMSAWGIERKATGKIRMLADPAGEFTKAVDLGFDATPALGNIRSKRYAMTIEDGVVKSVAIEPDATGLTVSLSCSILNE*

>Prx5_E.muelleri_6_Em0018g718a

MLRLSSVKLVRKFSSTVVSRMPIKQGETLPSVELQENTPANKVNIRDLFKGKKGVIFGVPGAFTPTCSKSHLPGFVADYDKLKSAGVDVIACVSVNDAFVMSAWGKENGAEGKVRMLADPTATFTKAIDVAFDATEALGGVRSQRYSMVVEDGVVKAINVEPDKTGLSCSAAKSIISQL

>Prx5_E.muelleri_7_Em0018g719a

MPIKKGDTLPSVELEEGTPDKKVNVRDLFKGKKGVIFGVPGAFTPVCSKSHLPGFVADYDKLKSAGVDVIACVSVNDAYVMSAWRKENAAEGKVRMLADPSAAFTKAVDLGYDATGSLGGVRSQRYSMVVEDGVVKCVNTEPDKTGLSCSGASSILSQV

>Prx5_E.muelleri_8_Em0018g725a

MLRLSSVKLVRKFSSTVVSRMPIKQGETLPSVELQENTPANKVNIRDLFKGKKGVIFGVPGAFTPTCSKSHLPGFVADYDKLKSAGVDVIACVSVNDAFVMSAWGKENGAEGKVRMLADPTATFTKAIDVAFDATEALGGVRSQRYSMVVEDGVVKAINVEPDKTGLSCSAAKSIISQL

>Prx5_E.muelleri_9_Em0018g726a

MPIKKGDTLPSVELEEGTPDKKVNVRDLFKGKKGVIFGVPGAFTPVCSKSHLPGFVADYDKLKSAGVDVIACVSVNDAYVMSAWRKENAAEGKVRMLADPSAAFTKAVDLGYDATGSLGGVRSQRYSMVVEDGVVKCVNTEPDKTGLSCSGASSILSQV

>Prx5_O.carmela_5_m.2809

MLSFRSLTSLSKRTFFLSSALRMSIKVGDKLPSVNLHEGTPGCSVNIADLFAKKKGILFAVPGAFTPGCSKTHLPGYVSDYEKMKGKGVDIIVCVSVNDAFVMSAWGEASNTPGKIRMLADTTAEFTKKIGMDFDAFAFLGSARSKRYSMVIEDGTVKTLNVEPDGKGLTCSLSNEILSQL*

>Prx5_S.ciliatum_5_scpid85348|

MLARFGFIQARRFSVSTASRMPIQVGDKLPSVSLHEGSPKGTVNIADLFKGKKGVLFAVPGAFTPGCSKTHLPGYVTDIEKYNAKGVEVIACVSVNDAFVMAAWGEAHGAAGKVRMLADTTGELTKAMEMDFDATPFLGGIRSKRYSMVIEDGVVKTINVEPDGTGLTCSLSNTILSQL

>Prx5_T.wilhelma_5_Twilhelma_g26829.t1

MLGVSRVAYRTLFTAPALRMPIKVGDTLPSIKLHEGAPNNTVDVKELFAGKKGILFAVPGAFTPGCTKTHLPGYVEDYEAFKAKNVDVIACVAVNDAFVMSAWGESQKAGGKVRMLADPNGEFTKAVDLGIDVGVLGGLRSKRYSMIVEDGVVTHLNVEPDGTGLTCSLSNELLNKM

>Prx5_X.bergquistia_2_Xb.08141.mrna

MKWTRFTCIFLALNFAPIHGCSISEILGDVSLNVSPKNCNGPHGISTSGLYKLRPNNNIGEFIGVCDMSLQGGGWTVILRRRDGTVDFNRNWNDYVTGFGHFGGEFFLGLDNIKHLTENGDMELWVGLQSHDVVFPPITLCSTIKWARYSNFKLEDPNYKLLVSGYDSQSTAGDSLSSHSMEEFSTFDRDNDRNLTHNCASSDFSGGWWYHDCLDSNLNGVWRSGGGDRTQIDGISWLFVFVKPRSYYIIVYDSHYVLSNLLKASSTYQGSTICAYAKQSIIFMFRRPLLLLSRNLTTTTRLKMPLKVGDSLPSVEVHEGTPKDKVNILELFKGKKGILFAVPGAFTPGCSKTHLPGFVEDFDKLKASGFDVIACLSVNDAFVMSAWGESAQATGKIRMLADATGEFTKAIDLELDATSALGNIRSKRYSMVIEDGVIKELNVEPDGTGLTVSLSCNLVKK*

>Prx5_X.bergquistia_3_Xb.08143.mrna

MPIKVGDTLPSIEVHEGTPEEKVNILELFKGKKGILFAVPGAFTPGCSKIHLPGYVEDFDKLKANIQIHLPGNDTYVMSAWGESAQATGKIRMLADPYREFTKAVDLELDPSLYPALGNIRSKRYSMVVEDGVVKELNIEPDSTGLNVSLSSMSTPSRRSPSQPSYDPNLVEVKSKFECEFRRFSVNRTQLTQYDEFNALVKESHELPSDMQFTLSYTDPRNGDLLPITNDENMIRAFVTAMPLLKLFVYREQDLMFEDMKLQERKKKNKSLFSESVKSTTTISAPKEFRKVGSIVDADLLPDTVRRVKLVKQSTDRPLGFYIRDGTSVRVTPYGLEKVPGIFISRLVPGGLAEGTGLLSVNDEIIEVNGIETLGKSLDQVTDMMVANSQNLIITVKPSTLPPTTPRSNTVARSRKGSPPPKSNRSSASEFPRPTKGDRSTPSRQQSTPETLNGTSNGPMKDLTKKRGTQEHEGVYNFK*

>Prx6_A.planci_3_gbr.41.68.t1

MVNLGDTFPNFEAETSVGKIKFHEWLGESWGILFSHPADYTPVCTTELSRAASLAGEFAKRNVKMIALSVDDVDSHVGWIKDIQAYSGQGGEFPFPIIADNRDLAVKFGMLDPDERDKAGMPLTARCVFIIGPDKKLKLSILYPATTGRNFDEILRVVDSLQLTATKKVATPVDWKEGGDCMVIPSVKQEEVAQLFPKGVTVKPVPSGKSYLRITPQP

>Prx6_B.floridae_4_gi|260811089

MPPLNLGDEMPNMTVVTNEGTIKMHDFLADSWAILFSHPKDYTPVCTTELGRACTLAPEFAKRKVKMIALSCDDADSHNGWIKDVQSHANHKGDFPYQIIADESREVAKKLGMIDPDESAAAGMPLTCRAVMIFGPDKRLKLSMLYPATTGRNFTEILRVIDSLQLTATKKVATPVDWTVGSKCMVVPSVKKEEEAGLFPKGVETLDVPSGKGYLRMTPQPE

>Prx6_B.floridae_5_gi|260811091

MPVMSLGDELPNFYLENNMESGNLHDFISGSWAVLFSYPRSFTPICTTELARAAQLAPEFAKRGVKMLALSCDNGDVNKDWIQDVKMNAGIEGEFPIRLVADQDRQIAKALGLIDQDQPNDVSMPITCRAIFVIGPDKRLRMSMVYPSSCGHNFEEILRSIDSLFMVESWVVGTPANWRPGDDVMVVPSIPKKEEATRFPKGVTRFSMPSGKDYMRLTSDDF

>Prx6_B.floridae_7_gi|260831656

MPNIGDIFPNFRAMTTEGEIDFYDWLGNSWGILFSHPGDFTPVCTSELGKAAQLAPEFQKRGIKIIGLSCSSTREHEAWIPDILAYTGLKGPMPFPIISDEKRELAVGLGMLDPEFKDDKGMPMTCRALFIIGPDKKLKMSILYPALSGRNFSEILRVVDSLQLTDVKKVSTPVDWKYGEDCMVDVSVPRVYEDHLFPKGVTIKALPSGKDYFRTTPMP

>Prx6_C.teleta_4_CapteP17912

MTGRVIRDWGVLFSHPRDFTPVCTTELGEVTKRAPEFKKRNCKLIALSCDGVDDHVAWSEDVMSYVGCNGKKLPYPIIADPTRDIATKLGMIDADEKDPSGMPVSCRAVFVVGPDHRLKLSILYPATTGRNFDEILRVIDSLQLTAKKSVATPVDWTPGKPAMVVPSLSPEEAKKMFPKHEVRSVPSGKGYLRFTPDY

>Prx6_L.anatina_4_g20032.t1

MVNLGDVFPNFDAKTTHGDFKFHDWIGDSWAILFSHPADYTPVCTTELGRVTKLVPEFNKRGVKLIALSCDNVESHKGWIEDIKSYMKDQGDFPYPIISDSDRSLAVSLGMVDPAEKDAAGLPLTCRAVSKAYKYVYSWTL

>Prx6_L.anatina_7_g649.t1

MVNLGDVFPNFDAKTTHGDFKFHDWIGDSWAILFSHPADYTPVCTTELGRVTKLVPEFNKRGVKLIALSCDNVESHKGWIEDIKSYMKDQGDFPYPIISDSDRSLAVSLGMVDPAEKDAAGLPLTCRAVFIIGPDKKLKLSMLYPATTGRNFTEILRVVDSLQLTVNQKVATPADWVNGGDCMVLPTIKEDEAAKLFPNHKTIPVPSKKPYLRITPQPK

>Prx6_N.vectensis_3_EDO47205

MPNLGDEFPNFTADSTIGTINFHDFIKDSWAILFSHPADYTPVCTTELGRVAQLEPEFKKRGVKMAGLSCDDAESHRGWVKDITKYNLEQNKSSAKFNYPIIADERRELAVKLGMVDPDEKDSKGLPLTCRAVFIIGPDKKLKLSILYPATTGRNFDEILRVIDSLQLTATKKVATPVDWKLGGDCMVIPSIKPEEEGTIFPKGVRALDLPSGKRYLRYTPQPE

>Prx6_X.tropicalis_3_gi|45360853

MPGLLLGEIFPDFEADTTIGRIKFHEFLGGSWGVLFSHPRDYTPVCTTELGRCVKLAPEFKKRNVRMIALSIDSVEDHLGWSKDINSYNCDEPTETLPFPIIADPKRDLAVKLGMLDPDEKDMQGMPVTARCVFIIGPDKKMKLSILYPATTGRNFDEILRVVDSLQLTAVHNVATPVDWKPGDRVMVPPNVPEEEASKLYPSGVFNKALPSRKNYLRYTAHPQ

>Prx6_D.rerio_6_ref|NP_957099.1

MPGILLGDVFPNFEADTTIGKIKFHEFLGNSWGILFSHPRDFTPVCTTELARAAKLHEEFKKRDVKMIALSIDSVEDHRKWSEDILAFNQDKACCPMPFPIIADDKRELSVLLGMLDPDERDKDGMPLTARCVFVVGPDKRLKLSILYPATTGRNFDEILRVVDSLQLTATKKVATPVDWKPGQEVMVIPSLSDEEANKLFPAGFTLKEVPSGKKYIRYTKP

>Prx6_H.sapiens_13_PRDX6

MPGGLLLGDVAPNFEANTTVGRIRFHDFLGDSWGILFSHPRDFTPVCTTELGRAAKLAPEFAKRNVKLIALSIDSVEDHLAWSKDINAYNCEEPTEKLPFPIIDDRNRELAILLGMLDPAEKDEKGMPVTARVVFVFGPDKKLKLSILYPATTGRNFDEILRVVISLQLTAEKRVATPVDWKDGDSVMVLPTIPEEEAKKLFPKGVFTKELPSGKKYLRYTPQP

>Prx6_C.elegans_3_WBGene00021401

MKLGDTVPNFTFETDLRKNQTLHNYIGEQWLMLFSHPADFTPVCTTELAELVKLAPEFRKRHVQILAISIDSSETHRDWAKDINSVAQLSNCGSHLPFEIIADTDRSICTELGMIDPDEMNSEGICLSARAVMLFGPDKKLKSKILYPATFGRNFVEILRMVDGVQLGTKAPVATPANWIAGDNVIAQPSLSQERVIQELCGGDPDKCKTVPLPSGKSYLRVIEGDAYLQN

>Prx6_C.intestinalis_3_ENSCINT00000019533

MGINLGQVFPNFDCKTTEGDLNFHEYIKDSWAVLFSHPADYTPVCSTELGAAALQHCEFQKRGVKMLEVSVDSVESHKGWVKDIQVLYSMSEKRFPFPLASVSRQLLSDLGMLDPDEVDSTGLPLTARCVFVIGPDKKLKLSILYPATTGRNFHEILRVIDSLQLTANSKVATPANWKKGEKCRVIPSLSDEEAVKLFPKGFEVTEVPSKKSYIRLTPDPSIMYFVNHVEKMTQYEHPLAPRQNAATKNEQQPLPPAEPSQQYDTWQHNQVVKANPYLSTAEIPRWLQVYSKAPQKFDNKLRWELFQVQELDCFSEMLTMLMKKELEQIVMSYESARQAIFAEVTSRKPEDEQNRFESNV

>Prx6_C.intestinalis_5_ENSCINT00000032511

MGVNLGQVFPNIDCKTTKGDYKLHDFINESWSILFSHPADYTPVCTTELGTAAQLKPEFDARGVKMIGLSIDSVDSHNGWIKDIQSYAGLQGEFPYPIIAGTRQTAADLGMLDPDEVDASGMALTARCVFIIGPDKKLKLSLLYPATTGRNFNEIIRVIDSLQLTATKKVATPANWKSGEDCMVVPSLSDAQATELFPKGFKVTEVPSKKSYIRLTPDPSNLP

>Prx6_D.melanogaster_1_FBgn0031479

MSGKALNIGDQFPNFTAETSEGRIDFYDWMQDSWAILFSHPADFTPVCTTELSRVAALIPEFQKRGVKPIALSCDTVESHKGWIEDIKSFGKLSSFDYPIIADDKRELALKFNMLDKDEINAEGIPLTCRAVFVVDDKKKLRLSILYPATTGRNFDEILRVIDSLQLTQTKSVATPADWKQGGKCMVLPTVKAEDVPKLFPDGIETIELPSGKSYLRITPQPMSHITRKRLLTLDEESQHINSTKRCGSNCIEKMLTKWSRQSIPGVNSISRKRSFPWEDNSPDIFPAKHSRKHIIKKKSKTIHEPEVPIDFLMGLPVLLDSDFSDSEECRPKSEIHVAKPETEDNRDLFTYQQLKLMCCEMMKQCEDRVVLEYEIALTQKMAEQYDTFIKFNHDQLQRECEHRASYLS

>Prx6_D.melanogaster_2_FBgn0033518

MRLGQTVPNFEADTTKGPIKFHEWQGNSWVVLFSHPADFTPVCTTELGRIAVHQPEFAKRNTKCLAHSVDALNSHVDWVNDIKSYCLDIPGDFPYPIIADPTRDLAVSLGMLDEEQKKDPEVGKTIRALFIISPDHKVRLSMFYPMSTGRNVDEILRTIDSLQLTDRLKVVATPANWTPGTKVMILPTVTDEEAHKLFPKGFDKVSMPSGVNYVRTTDNYMFEIESTKTVPPLPPKCPCRQPDHKDCGEGNRELQAEYHLLDIADVTDSVMKVMRICGLRPWEVKRAAGVIKRSLQHYEEIVQRIDRVPRKRFAKESFLHGLAHEAQMSRMDAQMAYSIVKRAFKAFYFATGIEGRRYICLADKMSHEMECLWLHAARRTAQIHAVLAGLMYSEELQFEERIECVYKNLFDVLKKRILWADNVTCTCGQHTAPISQAPSKTPSSATAQVEVLYGRYNMAVSSAPSKISTRGGTQKPSAAVSTASSRHNHILKKVDQPFSQKQTPRKGSSTTSASKTRCNCPHLRCCRANGEARESPEITAECSQGPYICRWLPYTEEDDEFPEHQVPFPPMEVVCPPCPKDDLSCDSECTCTCQVCTCQPEFDDGSDEGEDQGENLSKFGVEDQDTDFCYVAPFRGSSVERIARLKAKEQLLEVEEHLENEDKEEDEEFQCGCTCELKQHAYPHLFTYLTPFRIKEELVDKTHPKEERMQTVEDSESEDLLSKPPPPGVSLNTYRCWVKPPSSSHSSDEAKPPLVVVMGEKRKSNFQVTVKEQHHSNQASTVPPAAPLPKAPVLPNKPAPTPAPSKPPPVSQKAEEDKLTKEDILDIIGLRFR

>Prx6_D.melanogaster_3_FBgn0033520

MRLGQTVPNFEADTTKGPIKFHEWQGNSWVVLFSHPADFTPVCTTELGRIAVHQPEFAKRNTKCLAHSVDALNSHVDWVNDIKSYCLDIPGDFPYPIIADPTRDLAVTLGMLDEEQKKDPEVGKTIRALFIISPDHKVRLSMFYPMSTGRNVDEILRTIDSLQLTDRLKVVATPANWTPGTKVMILPTVTDEEAHKLFPKGFDKVSMPSGVNYVRTTENY

>Prx6_D.melanogaster_4_FBgn0033521

MRLGQTVPNFEADTTKGPIKFHEWQGNSWVVLFSHPADFTPVCTTELGRIAVHQPEFAKRNTKCLAHSVDALNSHVDWVNDIKSYCLDIPGDFPYPIIADPTRDLAVTLGMLDEEQKKDPEVGKTIRALFIISPDHKVRLSMFYPMSTGRNVDEILRTIDSLQLTDRLKVVATPANWTPGTKVMILPSVTDDEAHKLFPKGFDKVSMPSGVNYVRTTENY

>Prx6_M.leidyi_2_ML06033a

MKLGQVFPNEKVQTTQGELQLHDYWGDGWGIFFTHPADYTPVCTTELSMVQQMVPEFTKRNVKMIALSCNSVADHAGWCEDIKSYGKLSEVSYPIIEDPSRDLAVKFGMLDPEEKDAAGLPLTARAVFIVGPDKKLKLSILYPATTGRNFDELLRVIDSLQLTAYKKVATPANWQAGGDCMVLPSVKGEEATTLFPQMRIESVPSGKEYMRFTPQPQ

>Prx6_S.purpuratus_2_SPU_013368

MGGQNVRIANRTDRPSDDPCESRTIRASRKPAYYVRRTNETGRTNGAVTPVIKNKAGRITSKDNYRPIALASVLSKVLEKILLGRLEMYILTKDNQFGFKAKHGTDMCIFALKEIIASYRSRNSSLFLCFLDASKAFDRVNHEKLFTKMADRGIPKYLIRILVFCWGILFSHPADYTPVCTTELGRVATLTPEFEKRGVKLIALSCDGVESHRGWIKVRTKIKEEKKKQSNMSWGILFSHPADYTPVCTTELGRVATLTPEFEKRGVKLIALSCDGVESHRGWIKDIVDYAKFEKTWPYPIIADPKRELAVQFGMLDPDEKDSAGIALTARCVFIIGPDKKLKLSLLYPATTGRNFDEILRVIDSLQLTATKRVATPADWKSGEDCMVLPNISEEDAAKLFPQHRKVAVPSGKGYIRLTPQP

>Prx6_A.queenslandica_1_Aqu3.1.24376_001

MPLNLGDAFPNFKADTTEGQIQYYDWLGDKWGVLFSHPADFTPVCTTELGAVAKIVPEFEKRNAKVIAISCDSVEDHKKWIKDIQAYNGLGDNFPYSIISDPKRELAIQLGMVDPEEKDKAGLPMTCRAVFIIGPDKKLKLQILYPATTGRNFDEIIRVLDSLQLTANKKVATPANWTSGGDCMIVPSVSNDDAAKLFPKGFKVADVPSGKPYIRITPQPQ

>Prx6_E.muelleri_2_Em0006g1256a

MPLMNLGDDFPNFSVDTTEGRITFHDFLGNHWGVLFSHPGDYTPVCTTELAEVANLIPEFTKRDVKVIALSCDTVTAHKGWITDIKSYACYARSGWPYPLIADPNRDIAIQLGMLDPTAKDKSGIPVTCRAVFIIGPDKKLKLALLYPATTGRNFSEILRVIDSLQLTACKQVATPANWQPGGKCMVLPSISEADASELFPKGTELIKMPSGKHYMRYTPHPD

>Prx6_E.muelleri_3_Em0006g1269a

MPLMNLGDDFPNFSVDTTEGRITFHDFLGNHWGVLFSHPGDYTPVCTTELAEVANLIPEFTKRDVKVIALSCDTVTAHKGWITDIKSYACYARSGWPYPLIADPNRDIAIQLGMLDPTAKDKSGIPVTCRAVFIIGPDKKLKLALLYPATTGRNFSEILRVIDSLQLTACKQVATPANWQPGGKCMVLPSISEADASELFPKGTELIKMPSGKHYMRYTPHPD

>Prx6_E.muelleri_5_Em0016g274a

MPLNLGDTFPNFSVDTTEGRISFHDFLGDSWGVFFSHPSDYTPVCTTELAEVAKLIPEFEKRNVKVIALSCDPVEAHRGWIEDIKCFACYKDKWPYPIISDPSRELAVQLGMIDPDERDKAGMPLTCRAVFFIGPDKKLKLSLLYPSTTGRNFHEILRVIDSIQLTATKKVATPANWQPGGKCMILPTVSQEDAAKLFPKGYDLVSVPSGKQYIRLTPQPE

>Prx6_O.carmela_4_m.306381

MPNLGDVFPDFTAESTHGEIKFHDWLGGSWAILFSHPADFTPVCTTELGLVAKLAPEFQKRGVKMVALSCDPVESHNGWVKDIQAFTGAGTEWPYPIIADKNRELAVKFGMLDPDEKSAAGLPLTARVVFIIGPDKKLKLSILYPATTGRNFDEILRVIDSLKLTAEKKVATPANWKQGERCMVLPSVKQEDTPKLFPKGVEVVPVPSGKQYMRFTPQPE

>Prx6_O.carmela_7_m.316648

YIMTIRLGDIAPDFKQNSTEGEISFHEWLGDSWGVLFSHPKVFTPVCTTELGEVARLKESFSKRNVRVLALSVDKAEQQKEWEADIAETQGQT

>Prx6_S.ciliatum_4_scpid74385|

MGINLGDVFPDFTANTTKGSISFHSFLGDSWGILFSHPADFTPVCTTELGRVNQLLAEFEKRNVKPIALSCDPVDKHSDWIKDIQAYSAQSGDWGYPIIADEQRELAVRFGMLDPDEKDKAGMPLTARCVFIIGPDKKLKLSLLYPASTGRNFDEIIRVIDSLQLTAYKKVATPVNWQPGGECMVLPSVKPEDAKTLFPEHYTKELPSGKGYMRFTPQP

>Prx6_T.wilhelma_3_Twilhelma_g14367.t1

MPLNLGDTFPNPDVETTEGSFKLHDYWGDKWGILFSHPADFTPVCTTELGAVASIIPEFTKRGTKVIAISCDPVDAHKGWIKDIQAYNSLTDEKFPYPIISDPNRELAVQLGMVDPEEKDKAGLPLTCRAVFIVGPDKKLKLSLLYPATTGRNFDEILRVIDSLQLTANKRVATPANWKDGGDCMILPNVSKEDADKLFPGYKSADVPSGKKYIRTTTQPK

>Prx6_T.wilhelma_4_Twilhelma_g14367.t2

MPLNLGDTFPNPDVETTEGSFKLHDYWGDKWGILFSHPADFTPVCTTELGAVASIIPEFTKRGTKVIAISCDPVDAHKGWIKDIQAYNSLTDEKFPYPIISDPNRELAVQLGMVDPEEKDKAGLPLTCRAVFIVGPDKKLKLSLLYPATTGRNFDWLCDANLSEPVLIEILRVIDSLQLTANKRVATPANWKDGGDCMILPNVSKEDADKLFPGYKSADVPSGKKYIRTTTQPK

>Prx6_X.bergquistia_5_Xb.00785.mrna

MPLNLGDTFPNFTADTTEGTIQFHSWLGDSWGVLFSHPADFTPVCTTELGEVARIVPEFTKRGTKIIALSCNSVDSHKDWIKDIEAYNNLPQGPFPYAIISDPNRELAVQLGMVDPVEKDAAGLPLTCRAVFIIAPDKKLKLQILYPATTGRNFDEILRVLDSLQLTAHKKVATPANWKNGGECMILPTVSAEDAAKLFPGFKLFMILQAHKFIIDIEKYVSSLREEVVMIVSYHLMEESSCISGLVLLLQVLDLPVLLLPGELNMLLVAERGEPLLLRAPPTKFKEGCVSRWQKSQRGLLITAKLVIDNLINITLRHDISSSYLNRAIKVFPYNTKYLGEVHVEGGIYTLNLRKIYCDIDETQENRLLWTTEIERTIQAGTLSCLVQQLSPLKEEVDQSYWVCFLAVYRTFTTPYDLLTNLIDRYISHLNCTEYEEITHEKQQKCLKSILSFIKLWIDKYTEDFNCPPSFPELLMLETFLKDQLPQVTHHLLQELTELHRQVSTCHQKLSTAAMLSEQLCVCGHGGCDGSNKHYHWLYKPAELVAQDLTALDAEIFKDIIPYQCLHYVRHDKNKSPTILCAIDTFNKLTQQVLLSILEATDSKKMKTPLVRGQAISHWIDIAHFCRLLKNFSSLKAITSGLESSAIYRLKRSWEMVPNDKKVILTELHHLLSYESNFQVFRDVLDKEATGKRVAAEQSPKKPWKQIPEIIHGVVPYLGTFLTDLTMLHSALPDICSDGLINFEKRRKEFEILAKLSLFQTAASKYPFPLKVSLIHLLHTSPVMSDQESFLLSKKIEATPSPVGSHTSPSHLITPPCQILLQKSLQLFAKVAVGDITSTDINVQPKRLSVDLLKVEEEEQPSLHRSSSCQELQDYQVVRVSMTGSNSCNYKSIMISRCDHTAAVIKAALEKYDLTADPSSYELVQQLKDKGELKIPAGTSVYYALDNNIKEIPLLILKLKHR

**Glutathione peroxidase (GPX) sequences from this study**

>Ciona.intestinalis_20_GPx_ENSCINT00000003399

MMGHTGVALALVVALFCPALAHRAIGERSKCVSSSRTIYDTPFNFTMLNGTTVPLSKFRGEIHITINATYLSTHGANWQYPLFNALQELEGVTVLGFPCNQFGLQEPGANSEILKILEHVRPGGGFQPNFPMFEKLEVNGENAHPLFKFLKDQCNVVTSQFAPKARLFYEPIQPNDIEWNFHKFLVDQEGRARRRYHHNTPPDAAIVRKDIRFLQNN

>Ciona.intestinalis_21_GPx_ENSCINT00000024170

MAREVLRVLVLLAPLFGVFSENKHNGFYDYNVKTFDGETVSLKKYIGKVSLVVNVASECGYTDEHYKELTALQNELVQKEQPFTVLAFPCNQFGEQEPHDNHYIQEFASSEYKASFPIFAKIDVRDRDAHPAYEFLRRSTGQEPQWNFWKYLLDGSGNVINEWGPSISVSQVKDEILKAIQKLKNTHAEL

>Ciona.intestinalis_23_GPx_ENSCINT00000033901

QYSVYSNQVFNLHKQNVNLSRFHNEVTLLINVATYLNYSYLHQHFNGRNFSVLAFPCDQFHLEEPGEDSEILNGLMYVRPGNGYVPHPKLNIFGKIKVNGRHEHTIYKNVKASCPPTTLNLGSTRNMYWNPVKSTDITWNFNKFLLDKNGVPRYRISSDASPTSLIPYITTMLSE

>Ciona.intestinalis_22_GPx_ENSCINT00000033231

SSMAGKDVKDIYGFTVNDIDDQEVSLSKYKGHVCIIVNVASEFNYEQLQQLYGKYSQQGLKILAFPCNQFGKQEPKPNADIKKFATENYGVTFDLFSKINVNGDNAIPLYKFLKTHKNTTGTLVNAIKWNFTKFLVTKQGIPYKRFAPNAKPLDMVKDIEELLNQ

>Ciona.intestinalis_19_GPx_ENSCINT00000002066

MNALSEEYTQSSFVTLAFPCNQFGLQQPEANDEILNGVMYVRPGHGFVPNKKIYFFSKTQVNGGSEDPLFTSIKASCPPTTNNIGITSELYWTPIKANDIYWNWNKFLLDKNGMIRYRFGSAVTATQLKPWIDQLLNEK

>Capitella.teleta_9_GPx_CapteP229325

MGSWPTTGIAVLATALCCLATEDTRRLKFSECLDTRGDNIYGHNATELVSGNSIQMSGYTYQYKQLNALVRSFPHLKILAFPCHQFGHQEPGNESEILAGLKHVRPGGGFRPRFPLTSKTEVNGENEAPLYSFLKRSCPPTTDVIGNSSNLYFSPIKVTDVTWNFEKFLVDASGVPRFRFHPSVEPTEIVDFIEGLLFERARNQ

>Capitella.teleta_8_GPx_CapteP221397

MNQLMEQFGDRLQILAFPCNQFGHQENTTNDEILKSLKYVRPGNNYTPKFDMFKKVDVNGETAHPVFQFLREQLPTPSDDTVSLMSNPKFLIWSPVCRNDVSWNFEKFLIGPDGEPVKRYSRHFETINIASDIKKLM

>Capitella.teleta_7_GPx_CapteP172308

MKMAKCWFVCAVLSATLALVASVPGAEDLFSTPIVDANGRRLTLEEYRGRVLLIVNVASECGFTDGHYKSLVKLQMMFSRLQVLAFPCNQFGAQEPQDAASVQRWAKATYDVNFPIFGKVNVTGENASPLFRFLISATAKEPTWNFWKYLVNHEGRVLHAWGPWQDVEVVFPEIKAAVDAIPIDEAGSRRPRPPAPARVAEPRASLNGDTRVPAGPPEIKPPSEKPTGPKIGAPSRMKHDDL

>Oscarella.carmela_80_GPx_m.20201

MIIRNAFTRCFIYLIILVSVAGNFYKFSAKDIDGRMIQLSDFAGKVVNSFTVFVRTVCFFLQLSLVVNVASECGYTESNYEGLVDMQLRYERHDFTVLAFPCNQFGQQEPGSDSNIKTFVNRIYDVNFPLFSKIDVTGPNAHPIYQHLAKETGEVPTWNFAKYLINRSGKVVKFFDTRMDLKKVEAHIGHLLRERDEFX

>Oscarella.carmela_82_GPx_m.75808

MNALVDQYGSSGFAVLGFPCNQFGYQENFKEDEILRSLEYIRPGNGYSPKFDLFEKCSVNGEKTHPVFQFLKEKLPYPSDNQLSLMSDPKFIVWSPVTRADLSWNFEKFLIGADGEPFRRYGPSFHTKDLGPDIERLMKEKLKX

>Oscarella.carmela_81_GPx_m.310731

DLSDFDFRLAMPLFGKAKGFTPAQKTPRRKAASLSNLTSLDDSVYSQDELQLEQDGPVRIRLGGQELVFEDGNWIAENGAGAGSSREVARLKKQNQKLTEENNMLKYKNELLLDMLVASHADATVQARELEALKARQNGSKRRX

>Sycon.ciliatum_101_GPx_scpid40488

MAMASFVAGLLMQLLLFWSMGAERTMAVTHNVPETFYAHTAIDIHGQDIEMGQYRGKVVLIINVASECGYTDSNYKWLSKLSKRYSKHGLAILLFPCNQFGRQEPGSNPEIANFIGRQDVNEAKMFEKVDVLGEDAHPLFSFLEHRTGHSAKWNFAKYLVDGSGDYVRFYSTTEQLETVEDMIRQLLSMHTNLSDL

>Tethya.wilhelma_105_GPx_Twilhelma_g17718.t1

MSTTFITWWMNITLKTLISSSDSDRMKPVCFLPLAYFLVWRLTLATPLAIDLLPRLSPLTSERNVLFLSLSLSLSLSLSLSLSPEPPESAFKGPSVAYLRGQEGVLISLLAKKRLLIGWTYYVYILLPGTKKLYQYRTELESNPLSCVNLSGYSVQPESPPTSLIEDRGAIQRHILHFYNKEKVKCLSLSADSGDLQLSWLHALEAIGVEVLPAAEEVEDLVKNAKSIYEFEAKDIDGNATSMEIYRYEPGTEEEIKEFVSQFGVEFDMFSKVDVNGSSAHPLYKYLKSRVRSNLGSFIKWNFEKFLCDSEGKPVKRYLPTTQPLDIVPDIKTLWSDNVATP

>Tethya.wilhelma_106_GPx_Twilhelma_g17984.t1

MIVNVASNCGYTYSHYEGLVLLQEDYKSNSFNVLAFPCDQFGGQEPGSDADIEKFTREKFGINFPLFSKVSVENTNATLLYEYLYETTGSKPLWNFCKYLVDLNGEVVQYFSERDSFSVIRHSVEYVVNKNM

>Xestospongia.bergquistia_107_GPx_Xb.20769.mrna

MSRYLSSMSQGCACGISTKITEERSRDIRKAAALTDKIRSIGGLTIQDRTYRLRTYNTCFVASELVDWLISLGEAEDRKQAVALGQLLVDTDFIHHVVDEHNFEDSYLFFRFRQDEPPHEAMSGPSVAYMKGEQGALISLLAKRRPLALGWSYYVFALSSANKKIYQFRSELDSSPINCFSLEDVCVQPNVIKSNDRYILQFFVNHSQGREKTICLAADSYETQFTWIRSLGSCGVEILASTDEEDDLVRNATSIFDFEALDIEGKTVSLDKYRNGFRILAFPCNQFGKQEPGTLANVIEYAAKHRAEFDIFSKIEVNGPNAHPLFKYLKSKLKGNLGNFIKWNYAKFLCDSNGVPVKRYSPTTQPLDIVPDMESLWSSSX

**Glutathione peroxidase (GPX) sequences obtained by Trenz et al. (2021)**

Trenz et al. (2021) GPX abbreviation, followed by NCBI/Phytozome access code

>m.leidyi_gpx4a_MleiGPx04A_FC476807.1

MSPPALLIEEEATSTKWLLQTGDEGEILPWLKSMEQSGVSCLEPQEQICDEAECACNIYGFEVKDIDGNTVKLDKYKGYVTLIVNVASEXGLTELNYTQLQQIYSEYKDKGLRILAFPCNQFGKQEPGEHNEIIEFVSKYGVTFDMFSKVEVNGENAIPLYQYLKSRVSGSLGSLVRWNFQKFLVDRNGVPKFRYEPMFLQKQFLGH

>m.leidyi_gpx4b_MleiGPx04B_CF923339.1

MQDQYSSVFDFEAVDIHEQNQQLSKYKGNVTLVVNVASYXGLTPLNYEQLQQINEKYYDQGLRILGFPCNQFGNQEPGTDEDIIEFIKKYNVTFDMFHKINVNGANAIPLYKWLKEKLPGTITNAIKWNFTKFLSDRNGVPYKRYAPNFAPNDIIPDIEKLLAQEANKEES

>a.queenslandica_gpx7_AqueGPx07_XM_003388391.3

MEQVFFLSLFCLICGALASSGTETFYSLSAKDITGKMIGFERYSGKIVLVVNVASECGYTDYNYIQLNQLQLRYGEESLAILAFPCNQFGAQEPAKDSEINSMIRYKYKPQFPLFSKVNVTGESQSEVYRFLINSTNGLEPKWNFCKYLLDRDGLVVQFFDQGKTFDKIYDSIDGLLYRKRREL

>a.queenslandica_gpx4_AqueGPx04_XR_131726.3

MSVQPDAVKTSGRFSLQFYENHPQGKEKALCLAADNVDTQFMWIRALSSCGVEVLQSPEEETAQLKNATSIFEFSAVDIDKKTVSLDKYRGHVTLIVNVASQXGLAEKNYTQLVQLHSQYAHTRGLRILAFPCNQFKQEQGSEAEIKEFARRFGVEFDMFSKIDVNGPDAHPLYKFLKSRLKGSLGNFIKWNYAKFLCDANGKPFRRYSPTTQPLDIVPDMEALWSSETX

>a.planci_gpx1_AplaGPxIn01_XM_022250945.1

MATAASAAMPFSALLVVLWTVAALSTAATGPLESVCVREGSASVHLFSLGSLNDTSPPVPLSRYAGKVLLLVNVATYXGFTTLQYHQLNALAERYEGMLEILALPCNQFGLQEPGENDEILNGVKYVRPGGGFEPAFPVFAKIDVNGKKEHELYTHLKSVCPPVKLEIGDKSKLYWSDIKIGDITWNFEKFLVGGDGQAYKRYDPSIHPKGIEADIEGLILRERLRSEEERRDFEAFLHEKVY

>a.planci_gpx2_AplaGPxIn02_XM_022225248.1

MDRPSLAGLALALTLLALPGGSKAFLDSMCNSTQPIYDISVTALNGSSYKLSQYKGKVLVIVNVASFXGLATPQYPALNALRKAYEGKIEILAFPCNQFLLQEPEANGEILNLIKYVRPGGGFAPTFPMHQKIEVNGKNTHPLYKALKSVCPAVKEEIGDPSNFYWSPISNNDITWNFQKFLIDSNGIPYKRYDPLVGPSMLKTDIDLLLKKSS

>a.planci_gpx7_AplaGPx07_XM_022245999.1

MAAYTTRRSTCQKHLRFLCTIFVVIASTFPAPVSAAYDFYSFSADDPRGVEVSLNVFRGKPALVVNVASECGYTEGHYEDLVWLSKQPEMNERLHILAFPCNQFGQQEPASNQEILDFVQKTYGVTFPVFGKIDVIGKYAHPAYIYLSTDASGKQPTWNFWKYLVDRQGRVIDAWEPTTSVREIYDFLVAATQTRSGRRDEF

>a.planci_gpx4_AplaGPx04_XM_022254699.1

MSMTLVYDALYFAIQRPYTCLFVITLLFASYWWNLWGVFDSETMAMASGWITNFRALPSVVFGFTRMFSCTPRPAAADGPTDIYGFTVKDIDKNEVSLSKYKGHVVLIVNVASQXGLTNKNYAQLQELHATYAEPKGLRILAFPCNQFGGQEPGTNQEIKEFAAKKGAQFDLFDKIDVNGNNASPLYKFLKKKQHGTLTNAIKWNFSKFLINKKGVPVKRFGPQTNPKDIEKDIVKELDKPE

>c.elegans_gpx4a_CelGPx04A_NM_060197.3

MSSVYDFNVKNANGDDVSLSDYKGKVLIIVNVASQCGLTNKNYTQLKELLDVYKKDGLEVLAFPCNQFAGQEPSCEIDIQAFVADKFKFEPTLFQKIDVNGDKQSPLFKFLKNEKGGFMFDAIKWNFTKFLVGRDGKIIKRFGPTTDPKDMEKDIKEALGEKL

>c.elegans_gpx4b_CelGPx04B_NM_001381666.1

MASVHGITVKNAQGEDTPLSNYQGKVLIIVNVASQCGLTNSNYNQFKELLDVYKKDGLEVLAFPCNQFGGQEPSCEIDIAAFVADKFKFEPTLFQKIDVNGDNTAPLYKFLKQEKGGFLVDAIKWNFTKFLVGRDGHVIKRFSPTTEPKDMKKDIEAALQAKL

>c.elegans_gpx4c_CelGPx04C_NM_001028197.6

MNGRSLIFSILFVFSELKCDDTDENDQHGTIYQFQAKNIDGKMVSMEKYRDKVVLFTNVASYCGYTDSNYNAFKELDGIYREKGFRVAAFPCNQFEKQEPETEGKILDFVKSSYTYAPDMYSKIEVNGQNTHPLWKFLKKERGSSLSADIPWNFSKFLVDKNGHVVGRYSHSVNPIDLEEEISRLLNS

>c.elegans_gpx4d_CelGPx04D_NM_001129699.5

MYVQKLVNDDNAFHFFISLLNQRLKIDMSTGTIYDFSVRDNSGDLVSLDKYSGLVVIIVNVASYCGLTNSNYKELKSLNDKYHLRGLRVAAFPCNQFGFQEPHCEADINKFVNEKFSFEPDLYGKVTVNGGPLIGEEEPLWTFLKKEQGGTLFDAIKWNFTKFLVNRQGKVVARFGPSTNPKSFEEEIVKLLDENKL

>c.elegans_gpx1_CelGPxIn01_NM_077215.5

MAPGSVLSLAVALATIIGISCTATVDETMRWKECLNTNQSIFDFQIETLQGEYTDLSQYRGKVILLVNVATFCAYTQQYTDFNPMLEKYQAQGLTLVAFPCNQFYLQEPAENHELMNGLTYVRPGNGWTPHQELHIYGKIDVNGDNHHPLYEFVKESCPQTVDKIGKTDELMYNPVRPSDITWNFEKFLIDRNGQPRFRFHPTAWSHGDVVTPFIEQLLAEPAN

>c.elegans_gpx2_CelGPxIn02_NM_077214.6

MALWQLTFAALFAFVAAQPGPKMVDETTRWSQCKDTNQSIYDFQVETLQGEYTDLSQYRGQVLLMVNVATFCAYTQQYTDFNPLIEKYQSQGFTLIAFPCNQFYLQEPAENHELMNGIMYVRPGNGWKPHQNLHIYGKLDTNGDNQHPIYEFVKESCPQTVDKIGKTDELMYNPIRASDITWNFEKFLIDRNGQPRFRFHPTAWSHGDVVTPFIEQLLAEPAN

>c.elegans_gpx3_CelGPxIn03_NM_067841.5

MASNSLLTLAVSFTVILFAFCVEVDDTLRWKQCAVTNQSVFDFQIETLKGDYTDLSQYRGKVTLLVNVATFCAYTQQYTDFNPILDKYQKQGLVIAAFPCNQFYLQEPAENHELLNGLTHVRPGNG

>d.melanogaster_gpx4a_DmGPx04A_5638

MSANGDYKNAASIYEFTVKDTHGNDVSLEKYKGKVVLVVNIASKCGLTKNNYEKLTDLKEKYGERGLVILNFPCNQFGSQMPEADGEAMVCHLRDSKADIGEVFAKVDVNGDNAAPLYKYLKAKQTGTLGSGIKWNFTKFLVNKEGVPINRYAPTTDPMDIAKDIEKLL

>d.melanogaster_gpx4b_DmGPx04B_5641

MFDKEFLFPGLLVAVALVVVLQTRSRLQQDLQDMRWRLTIHALTVRDTFGNPVQLDTFAGHVLLIVNIASKCGLTLSQYNGLRYLLEEYEDQGLRILNFPCNQFGGQMPESDGQEMLDHLRREGANIGHLFAKIDVKGAQADPLYKLLTRHQHDIEWNFVKFLVDRKGNIHKRYGAELEPVALTDDIELLLGR

>n.vectensis_gpx1_ NvecGPxIn01_XM_032366687.1

MKPSASFSLLVMISLYISLSKAQDNMSGPCTAIAGSINEFQLEDLDGKVQDLRDKVVLVVNVASFXSLTKVHYEQLNALKERFKSDRCGLEIVGFPCNQFKLHEPGDTATEIRNCVKYVRPGGGFEPNFPLMKKTEVNGIKEHPLYTFLKTSCPSPDGVIREDRYKDVRVLWSPIKSDDISWNFEKFLIDHRGKPVRRYKPRLFPERMVQDIDSVINCCKSAAYCQKEGL

>n.vectensis_gpx4a_NvecGPx04A_XM_032387189.1

MAAGGKQTILDFEVKDIDGNDVALSKYKGFVTLIVNVASQXGFTKKNYTQLQELHSRYAENGLRILAFPCNQFGKQEPGTAEEIKEFVKQYNVEFDMFAKIEVNGKGADPLYVFLKEAKHGTLTNAIKWNFTKFLCDKEGVPVKRYSPNTAPLDIEKDIKEQLEK

>n.vectensis_gpx4b_NvecGPx04B_XM_032387188.1

MLNFSGRLLQAVKGSVTLTPKAFSGLLGFGASTALFGTRVMAAGGKQTILDFEVKDIDGNDVALSKYKGFVTLIVNVASQXGFTKKNYTQLQELHSRYAENGLRILAFPCNQFGKQEPGTAEEIKEFVKQYNVEFDMFAKIEVNGKGADPLYVFLKEAKHGTLTNAIKWNFTKFLCDKEGVPVKRYSPNTAPLDIEKDIKEQLEK

>n.vectensis_gpx4c_NvecGPx04C_XR_004290791.1

MWGTVVLVLKQGDETLYEFKTELDSSPAKTYLLKDASVKYDKSAKYCLLVSFPDIQISDLRLSFQSDDEQLSWVKAFAGSGANTSELKEDIEEQVAEAKSIFEFKAKDIDGALVELSKYMGLVTLIVNVASFXGLTQKNYSQLVELHSQYAEKGLRILAFPCNQFGKQEPDPEPVIKQFAAGYGVKFDMFAKVNVNGSSALPLYKYLKNELKGTLGSFIKWNFAKFLCNKDGKPIKRYAPTTAPLDIAKDIEELL

>n.vectensis_gpx7_NvecGPx07A_XM_001625762.2

MELCKQFLLLGIATLLLQPCFCSQFYSFTAKDIHGQDVSMEKYRGKVVLIVNVASECGFTDVNYRELVALHNKYSKEGLAILAFPCNQFGKQEPKRNYGIYRFAVDYYGVQFDMFSKIKTVGDGSHPLYNFLVESTGFPPIWNFNKYLVNRAGVVVKYFNHSFNPSSFESIILRHLRPGIHDGFESTSINDEL

>l.anatina_gpx4a_LanGPx04A_XM_024076110.1

MPSARIGVVIMYLLIPLLPILLFASCETDIVRSDGQEIRSVIMDLLIVLLPIVFLVSCDGSIIRYDGQEIFTLVIVGIKKVECLFDSQADAKEKDWWKKAGSIYEFTVKDIDGNDVSLEKYKDHVVLIVNVASQXGFTAKNYTQLQSLHAKYAESKGLRILGFPCNQFGGQEPGTEAEIKKFVEKFDVQFDMFSKIKVNGGDADPLWKYLKHKQGGTLIDAIKWNFTKFLVDKKGQPVKRYAPNTEPFTVEKDFDEYF

>l.anatina_gpx4b_LanGPx04B_XM_024076109.1

MTATPETRDHVVLIVNVASQXGFTAKNYTQLQSLHAKYAESKGLRILGFPCNQFGGQEPGTEAEIKKFVEKFDVQFDMFSKIKVNGGDADPLWKYLKHKQGGTLIDAIKWNFTKFLVDKKGQPVKRYAPNTEPFTVEKDFDEYF

>l.anatina_gpx1_LanGPxIn01_XM_013555629.2

METSLLLLLAAVVGVWAVSPPKLCRRKDDKTVYAFKQNSLLTGEEISLSRYRGNVLLIVNVATYXGLTSQYHGLNALQTKYSPLGLTVLAFPCNQFGMQEPGRNATEIWNGIKEVRPGHGFTPNFQMFTKTEVNGINENPLYTYLKTRCDSTVEDFATDDKLFYQPKRSQDIRWNFEKFLVDHRGFPLKRFHPRTNPDNAEFISTLEEALDMRNGRMEIRDQASSNGKTMQKAYCSLVQTICLKIH

>l.anatina_gpx2_LanGPxIn02_XM_013559495.2

MARSPVKIRNFFDFSAKLLSGEDINMSRYKGKVILVENVASLXGTTVRDFTQMNELNSKFKGQLVVLGFPCNQFGHQENASGQEILNSLKHVRPGNGFEPNFPIFDKVLVNGQDAHPIFQFLRESLTYPSDEPTEFISNAKLIIWEPVTRSDIAWNFEKFLIGPDGKPYKRYSRKFQTCNIGYDIAELIKLHNL

>l.anatina_gpx7_LanGPx07_XM_013554199.1

METSMSPKICPKNILNVVLFSFFFVNIFNEILCGPATIDPLKVKKKPGYRPSTGGLQPGEIVYEDVTPTTDEPEKDFYMFSVTDIKGKTVSLEEYRGMVTLVVNVASECGYTDSHYKALVKLQNTLAPSGKFTVLAFPCNQFGAQEPKDEPSIEKFAKEKYGVNFPMFSKINVVEKDIPEAWKFLEDFSRLVPNWNFWKYLINPSGHVIATWGPWIPVEDVIEQITEAVHAAADDLVVSDTIHEEL

>b.floridae_gpx4a_BflGPx04A_XM_035828545.1

MGGALLGLFAGATTVFLKRSLVQASRGVTNMATGGEEWKNATSIYEFSAKDIDGNEVSLEKYRDHVCLIVNVACKXGGTNVNYTQLQELHDKYAESKGLCILAFPCNQFGGQEPWPEPEIKKWVTDKFGVKFDMFSKINVNGKDAHPLWKYLKSKQGGTLIDAIKWNFSKFLINKEGQPVKRYGPNVKPLEIEKDFEPYW

>b.floridae_gpx4b_BflGPx04B_XM_035813181.1

MTEGWRVPALPIHAQTFLHSTTQSPHPPYLERPERTGRKSYPRDKFRTILFVTGGGRGEEILARYLALRRTNTSVTMPAEEISEETLAFIEVINIVVQHVAEIRSLLDYYRYITAEKQSKCERLANTAMDSLNELRDTLAEAKKQRELELEMMRGKLVTEMVDVTYDFHDDVIRNPASRSEIEEMLKGYNIVEYNIPPQGGDPDMDIVYKLTGLPEDVARAKFALQEKCDAMAAESRKKKQRGQQIDKYWTSVDPNKWRKTRFIYEFEAKDIDGNMISFEKYRGQPLLIVNVASRCGGTDRNYKQLMDLYRKYGEKGLRILAFPCNQFHNQEPYIERDIKEFVTTRYGVSFDMFSKIHVLGPETHPIYNWLVNTTRGTLGDIIKWNFTKFIVDKKGRAVNRYGPNVDPEKIDPDIPKYL

>b.floridae_gpx7_BflGPx07_XM_035807354.1

MRRSHVSPRMHRDTTGRMAAGQANFLLALLLSLASSAISQDDFYSFTAKDIKGKTVLLDKYRGKVSLVVNVASECGYTDGHYRELVRLQDHLAPTKHFNVLAFPCNQFGGQEPMGNSAIAQFAKSMYKANFPMFSKIDVVGREAHPAYKYLAESTQAPPTWNFWKYLVDPNGKVITAWPPHNAVVDIWSKVESAVNRARWDKGQRSTEL

>b.floridae_gpx1_

MAAATAVKSFFELSAKALSGEMVSFSRYQGKVVLVENVASLXGTTVRDFTQLNELAAMFGDKLAILGFPCNQFGHQENATNEEILNSLKYVRPGNGYEPKFDMFSKVQVNGSDAHPVFAYLREKLPIPADSENAFLIMNDPKCVIWSPVTRTDIAWNFEKFLIGPDGQPIKRFSRYFQTIDIKNDIEALLK

>b.floridae_gpx2_BflGPxIn02_XM_035840888.1

MAMMNNYHHSKMDGSWVYANITGPGPGHNTISTSTQHVRNFFDLSGQALSGAGAGDIIHFSRYTGKVVLVTNVASACYLTTREFTQLNNLMHLYGLHGLVILAFCCNQFGHSEPFENDEIVKCLRYVRPGPPFQPSFQLFVKCDVNGSRTHRVFDFLKDRLPYPSDDNTMLVAESSEITWNPVKRNDITYNFEKFLIGRDGQPYRRYSYKTPPSRLHQDIKRLLGIE

>b.floridae_gpx3_

MRLVSGLVAVLLCCGVISSEKSIYPKQRADCSCTAEHGSFHDHHAMLLDGSRNVSLAEYRGTTLLVVVVASFXGFTHQYPAMNALKEKMVGQLGFDFDILAFPTNQFGLQEPETNGEILNVLKYVRPGGGYVPNFPLFQKGDCNGENEQSLFTYLKSCCPPASDMIVDDKSSLYWKPLRAGDVRWNFEKFLVDPEGKAVMRFTPPVEPAEMEPVIEEFLRKWNVKVQREISAMEDLLSDLMIEQ

>b.floridae_gpx4_BflGPxIn04_XM_035819789.1

MGLSSGLLASLLCLGFLGPGLANKRTEQCSCAAEQGSLHDHHAMLLDGSRNVSLAEYRGTTLLVVTVATFXGFTQQYVGLNALRNKMVEENGHRFEILGFPTNQFGLEEPARNDEILNGIRYVRPGNDYVPNFTMFQKGDCNGENEQSLFTYLKSCCPPISDVMGIRGDKDRLYWKPLKVNDVRWNFEKFLVDPEGRGVKRFSSYVTPEDLESVIEEFIQSWNDTHGGDTSGARSARDTGLMSWLN

>s.purpuratus_gpx7_SpurGPx07_XM_030996353.1

MGFQALRLLFLVNIFMTWHVDYCHGRREWHDNFYDFTVKNIEGKRVRLEKYRGTPSLVVNVASDCGYTDRTYRDLVDLSKDPQFEDRLNILAFPCNQFGHQEPQSNEEIKHYVKALYDVEFPLFAKVEVKGYDADPAWQYLTSNAHQEPTWNFWKFLVDSEGKVKDAWGPDVPIHSIYGELLTEALMADPYHYHHAHFDDEF

>s.purpuratus_gpx1_SpurGPxIn01_XM_787470.4

MRGCSFSFFKMATYEKIILAFFALVVCSRLSGATMLDAVCYDDAESLSDVAMTKSLSLDDYRGKVVLVVNTASFCTYTYQYPYFNELKNEFGDQLAILGFPCNQFWLQEPGVGQEIPNTLRYVRPGGGYEPNFYLNEEKIDVNGPKAHPLFKKLKNSCPPVKMEIGDPSNLYWSPMTIGDVTWNFNKFLLDKEGVPFKRYDSVVEPLQLVSDIQLVVDGDYKTVKRPSRPKYATEEL

>e.muelleri_gpx4a_EmueGPx04A_scaffold_0011/

RGHVTLVVNVASEXGLTDKNYTQLVQLYTEHAGTHGLRILAFPCNQFGKQFDMFSKIDVNGANAHPLYKYLKSHISGSLGSFIKWNFEK

>e.muelleri_gpx4b_EmueGPx04B_scaffold_0011/

QEPGTEAEIKAFAASYGVQFDMFSKIDVNGANAHPLYKYLKSHISGSLGSFIKWNFEKVRKEGEAGX

>X.tropicalis_GPX1_XtGPx01_NM_001015740.3

MRLAMVSRTVYEFSARLLSAGENTALSQYKGRVLLIENVASLXGTTIRDYTQMSRLQSMYGPRGLQVLAFPCNQFGHQENSGNQEILNILKHVRPGGGFEPNFPLFEKVDVNGEKEHPLFTFLKGQLPYPSDDSISLMQDPKSIIWSPVRRNDIAWNFEKFLIARNGVPYKRYGRRFETFNIQQDIEKLLDETCE

>X.tropicalis_GPX2_XtGPx02_NM_001256315.1

MAYIAKSFYDLYATNIDGEKVDFNVFRGRVVLIENVASLXDTTVRDYTQLNELQTKYPRRLVVLGFPCNQFGYQENCKNEEILNSLKYVRPGKGFVPGFTLFQKCDVNGKDTHSVFAYLKDKLPVPDNEPAALISDPRYIVWNPVHRSDISWNFEKFLIGPEGEPFKRYNKNFQTISIEPDIQRLLKLTK

>X.tropicalis_GPX3_XtGPx03_NM_203630.2

MGVKLRGLLMLPCFLAALIHAQNEMDQKSVDCYSSIDGTIYDYGATTLDGTQFIPFKAYQGKYILFVNVATYXGLTMQYQELNALQEELKNNNFVILGFPSNQFGMQEPGRNDEILLGLEYVRPGGKFVPNFQLFEKGDINGRKEQKFYTFLKNSCPPVGDNFGSATNRLMWEPIKVNDVKWNFEKFLVGPDGRPVKRWLPRTPVAQVRREIMSYMKLQPGTQRLLMLGLEQK

>X.tropicalis_GPX4_XtGPx04_NM_001304772.1

MLNLLCRSIKRSVLLGSVIGGVQSRAMCAQVADWKAAKTIYEFSAVDIDGNEVSLEKYRGYVCIIVNVASKXGKTPVNYTQLVELHAKYAEKGLRILGFPCNQFGKQEPGDEAQIKDFAASYKVEFDMFSKIEVNGDGAHPLWKWMKEQPKGHGTLGNAIKWNFTKFLINREGAVVKRFSPMEDPVVIEKDLPNLL

>X.tropicalis_GPX7_XtGPx07_NM_001078936.1

MYLTALVLLLLLPPSLQKSRDFYTFKVVNIRGKLVSLEKYRGSVTLVVNVASECGFTDSHYKALQQLQRDLGSYHFNVLAFPCNQFGQQEPNSDREIENFVRKNYSASFPMFSKTAVTGTGVNSAFKYLIESSGKEPDWNFWKYLVGPDGKVVDAWGPSVSVAEVRPHITSLVRKIILKRKEEL

>X.tropicalis_GPX8_XtGPx08_XM_012956343.3

MEPLSPYPLKYSSPKAKVFLVFLSMVLCTGLVCVLQLKFLRAKGGDFYSYEVTDAKGRTVALSKYRGKASLVVNVASSCPHSETNYRSLQELHREFGPYHFTVLAFPCNQFGESEPGSNREIEALAKRNYGVTFPMFSKIKILGPEAEPAYKFLVDSTKTKPRWNFWKYLVNPEGQVVKYWRPDETAEIIRPEVASLVRQIIMKKKEDL

>H.sapiens_GPX1_HsGPx01_3600

MCAARLAAAAAAAQSVYAFSARPLAGGEPVSLGSLRGKVLLIENVASLXGTTVRDYTQMNELQRRLGPRGLVVLGFPCNQFGHQENAKNEEILNSLKYVRPGGGFEPNFMLFEKCEVNGAGAHPLFAFLREALPAPSDDATALMTDPKLITWSPVCRNDVAWNFEKFLVGPDGVPLRRYSRRFQTIDIEPDIEALLSQGPSCA

>H.sapiens_GPX2_HsGPx02_3601

MAFIAKSFYDLSAISLDGEKVDFNTFRGRAVLIENVASLXGTTTRDFTQLNELQCRFPRRLVVLGFPCNQFGHQENCQNEEILNSLKYVRPGGGYQPTFTLVQKCEVNGQNEHPVFAYLKDKLPYPYDDPFSLMTDPKLIIWSPVRRSDVAWNFEKFLIGPEGEPFRRYSRTFPTINIEPDIKRLLKVAI

>H.sapiens_GPX3_HsGPx03_3602

MARLLQASCLLSLLLAGFVSQSRGQEKSKMDCHGGISGTIYEYGALTIDGEEYIPFKQYAGKYVLFVNVASYXGLTGQYIELNALQEELAPFGLVILGFPCNQFGKQEPGENSEILPTLKYVRPGGGFVPNFQLFEKGDVNGEKEQKFYTFLKNSCPPTSELLGTSDRLFWEPMKVHDIRWNFEKFLVGPDGIPIMRWHHRTTVSNVKMDILSYMRRQAALGVKRK

>H.sapiens_GPX4_HsGPx04_3603

MSLGRLCRLLKPALLCGALAAPGLAGTMCASRDDWRCARSMHEFSAKDIDGHMVNLDKYRGFVCIVTNVASQXGKTEVNYTQLVDLHARYAECGLRILAFPCNQFGKQEPGSNEEIKEFAAGYNVKFDMFSKICVNGDDAHPLWKWMKIQPKGKGILGNAIKWNFTKFLIDKNGCVVKRYGPMEEPLVIEKDLPHYF

>H.sapiens_GPX5_HsGPx05_3604

MTTQLRVVHLLPLLLACFVQTSPKQEKMKMDCHKDEKGTIYDYEAIALNKNEYVSFKQYVGKHILFVNVATYCGLTAQYPELNALQEELKPYGLVVLGFPCNQFGKQEPGDNKEILPGLKYVRPGGGFVPSFQLFEKGDVNGEKEQKVFSFLKHSCPHPSEILGTFKSISWDPVKVHDIRWNFEKFLVGPDGIPVMRWSHRATVSSVKTDILAYLKQFKTK

>H.sapiens_GPX6_HsGPx06_3605

MFQQFQASCLVLFFLVGFAQQTLKPQNRKVDCNKGVTGTIYEYGALTLNGEEYIQFKQFAGKHVLFVNVAAYXGLAAQYPELNALQEELKNFGVIVLAFPCNQFGKQEPGTNSEILLGLKYVCPGSGFVPSFQLFEKGDVNGEKEQKVFTFLKNSCPPTSDLLGSSSQLFWEPMKVHDIRWNFEKFLVGPDGVPVMHWFHQAPVSTVKSDILEYLKQFNTH

>H.sapiens_GPX7_HsGPx07_3606

MVAATVAAAWLLLWAAACAQQEQDFYDFKAVNIRGKLVSLEKYRGSVSLVVNVASECGFTDQHYRALQQLQRDLGPHHFNVLAFPCNQFGQQEPDSNKEIESFARRTYSVSFPMFSKIAVTGTGAHPAFKYLAQTSGKEPTWNFWKYLVAPDGKVVGAWDPTVSVEEVRPQITALVRKLILLKREDL

>H.sapiens_GPX8_HsGPx08_UniProtKB/Swiss-Prot:

MEPLAAYPLKCSGPRAKVFAVLLSIVLCTVTLFLLQLKFLKPKINSFYAFEVKDAKGRTVSLEKYKGKVSLVVNVASDCQLTDRNYLGLKELHKEFGPSHFSVLAFPCNQFGESEPRPSKEVESFARKNYGVTFPIFHKIKILGSEGEPAFRFLVDSSKKEPRWNFWKYLVNPEGQVVKFWKPEEPIEVIRPDIAALVRQVIIKKKEDL

>D.rerio_GPX1_DrGPx01_5605

MAGTMKKFYDLSAKLLSGDLLNFSSLKGKVVLIENVASLXGTTVRDYTQMNELHSRYADQGLVVLGAPCNQFGHQENCKNEEILQSLKYVRPGNGFEPKFQILEKLEVNGENAHPLFAFLKEKLPQPSDDPVSLMGDPKFIIWSPVCRNDISWNFEKFLIGPDGEPFKRYSRRFLTIDIDADIKELLKRTK

>D.rerio_GPX2_DrGPx02_NP_001316688.1

MTFIAKTFYDLHATTLEGDTIDFNIYRGRVVLIENVASLXGTTTQDYTQLNELQSRYPHRLVVLGFPCNQFGYQENCSDGEILNSLKYVRPGEGYKPSFTIFQKCVVNGSDAHPVFSYLKDKLPYPDDDPVTLIQDPKYLVWNPVSRNDISWNFEKFLIGPEGEPFKRYSKKFQTINIEPDIQRLLKLTKN

>D.rerio_GPX3_DrGPx03_5613

MGTQSNPWTSVVLLLALMHKIAALSNTQACNSAAGDSFHNYGAKTINGTQFIPFSHYAGKHVLVVNVATYXGLTFQYVELNALHEELRHLGFTILGFPCDQFGKQEPGENNEILSALKYVRPGNGFVPNFQLFEKGDVNGDGEQALFTFLKNACPPVGESFGATSNRLFWEPLKVNDIKWNFEKFLLDPDGRPVMRWFPRVNVSEVRADILKYFHQ

>D.rerio_GPX4a_DrGPx04A

MRFLGSAVVFSLVLQTMSAQLEDWQTAKSIYEFTATDIDGNEVSLEKYRGKVVIITNVASKXGKTPVNYSQFAEMHAKYSERGLRILAFPSNQFGRQEPGTNSQIKEFAKSYNAEFDMFSKIDVNGDGAHPLWKWLKDQPNGKGFLGNGIKWNFTKFLINREGQIVKRYSPLQDPSVVEKDLSKYL

>D.rerio_GPX4b_DrGPx04B

MWLFQRALLVGAVGSKSFARAMCAQANDWQSAKSIYEFSAIDIDGNDVSLEKYRGYVCIITNVASKXGKTPVNYTQLAAMHVTYAEKGLRILGFPCNQFGKQEPGSEAEIKEFAKGYNAEFDLFSKIDVNGDAAHPLWKWMKEQPKGRGTLGNNIKWNFTKFLIDREGQVVKRYGPMDDPSVVEKDLPKYL

>D.rerio_GPX4c_DrGPx04C_NM_001346537.1

MQKMGFIHRFLLFGALSSSGIIGATSAQLEDWQTAKSIYEFTATDIDGNEVSLEKYRGKVVIITNVASKXGKTPVNYSQFAEMHAKYSERGLRILAFPSNQFGRQEPGTNSQIKEFAKSYNAEFDMFSKIDVNGDGAHPLWKWLKDQPNGKGFLGNGIKWNFTKFLINREGQVVKRYSPLQDPSVVEKDLSKYL

>D.rerio_GPX7_DrGPx07_5609

MGMFLRAFTLIILLCLLEAKQKDFYTFKVVNSRGRLVSLEKYRGSVSLAVNVASECGYTDEHYKDLQQLQKDFGPFHFNVLAFPCNQFGQQEPGSDKEIDSFVRRVYGVSFPIFSKIAVVGIGANNAYKYLVEASRKEPTWNFWKYLIDTDGKVVDAWGPEVSVKEIRPRITEMVRKLIIKRKEEL

>D.rerio_GPX8_DrGPx08_NM_200222.1

MEALGGYPSKSSASRAGLFKVLLSVALCMGSLYLLQNKLSKSRKTKDFYSYEVKDARGRTVSLEKYRGKVSLVVNVASGSELTEQSYRALQELHRELGTSHFNVLAFPCSQYGDTESGTSREIEAFAKSNYGVTFPIFNKIKIMGSEAEPAFRFLTDSVQKIPRWNFWKFLVSPEGQVVRFWKPEEPVSDIRKEATTLVRNIILKKRQEL

**Alignments**

**Alignment for Fig 2A – CAT unrooted tree including non-metazoan sequences**

>Acanthaster.planci_4_CAT

MASRDKATNQMEEYKKTLDKMDRLTTSTGMPIDNKQATLTAGPRGPVLMQDFAFSDEMSHFGRERIPERVVHAKGAGAFGYFEVTHDISEYTKACVFESIGKKTPVAVRFSTVGGESGSADTARDPRGFAVKFYTEDGNWDLVGNNTPIFFIRDPIFFPSFIHTQKRNPVTHLKDPDMFWDFIT-LRPESTHQVSFLFSDR-GTPDGYRHMNGYGSHTFKLVNKDGKGVYCKFHLKTDQGIRNLNAAQAEALASGDPDYAIRDLYNAISKEDFPSWSVKIQVMTFEQAEQHKDNPFDLTKVWPQAEYPLIPVGKMVLNLNPRNYFAEVEQIAFAPAHMIPG-IEPSPDKMLQGRLFSYPDTHRHRLGTNYLQIPVNCPYKAKTRNYQRDGPQCVTDNQNGAPNYYPNSFNGPTDDLKYAQQTFSISGDVARYNTKD-DDNFTQPGIFWSKVLTPKDQDALVSNMAGHLKNAQ-EFIQKRAVDNWSQCSAEWGKRLQAALDEHKAVA

>Amphimedon.queenslandica_6_CAT

MAASKGSTSQLVNFAAYKKSPDILTTSHGHPVDCKTAILTAGAKGPVLLQDYVFLDEMAHFDRERIPERVVHAKGAGAFGYFEVTHDITNYCKAKVFNKIGKRTPIAVRFSTVGGESGSADTVRDPRGFAVKFYTEDGNWDLVGNNTPIFFIRDPILFPSFIHTQKRNPSTHLK--------------------------------------------------------------------------------------------------------------------------------------------------------------------------------------------------------------------------------------------------------------------------------------------------------------------------------------------

>Amphimedon.queenslandica_7_CAT

---------------------------------------------------------MAHFDRERIPERVVHAKGAGVFGYFKVSHDITNYCKAKVFNKVGKRAPIAVHFSTVGGESGSADTVRDPRGFAVKFYTEDGNWDLIGNNSPLFFIRDPILFPSFMHTQKRNPSTHLK--------------------------------------------------------------------------------------------------------------------------------------------------------------------------------------------------------------------------------------------------------------------------------------------------------------------------------------------

>Xestospongia.bergquistia_43_CAT

MDTRDKAANQLTDFAKSKKSPDVLTTSHGHPVDNKTATLTAGPKGPVLIQDYIFLDEMAHFDRERIPERVVHAKGAGAFGYFEVTHDISKYCKAKVFSQIGKRCPIAVRFSTVGGESGSADTVRDPRGFAVKFYTEDGNWDLVGNNTPIFFIRDPILFPSFIHTQKRNPTTHLKDPDMFWDFIS-LRPETTHQVSFLFSDR-GIPDGYRHMNGYGSHTFKLVNINGEPVYCKFHYKTDQGIKNLSVEKAGILAGDEPDYGIKDLYVAIATRNFPSWTLYIQVMTFEQAERYRWNPFDLTKIWPHKEFPLIPVGKLVLNRNPSNYFAEVEQVAFSPAHMVPG-IEPSPDKMLQGRLFSYDDTHRHRLGVNYHQIPVNCPYATRTRNYQRDGFMTVDGNQGGAPNYFPNSFSGPLDNPSHAISKTITTGDVRKYNTKD-DDNFSQVTNFWLHVLSTDEKTRLVSIIAGHLKNAA-DFIQKRAVRNFTEVHPEYGGRIAALLEKYKQIH

>Oscarella.carmela_28_CAT

MSATAKASEQLLNYSKHGKNAGVCTTGDGIPVDTTTATMTAGHRGPVLLQDYNFLDVMAHFDHERIPERVVHAKGAGAFGYFEVTHDITQYCKAKVFERVGKRTPLAVRFSTVGGESGSADTVRDPRGFAVKFYTDDGNWDLVGNNTPIFFIRDPILFPSFIHTQKRNPVTHLKDPDMFWDFIS-LRPETTHQVSFLFSDR-GIPDGHRHMNGYGSHTFKMVNKDNKPVYVKFHYKTDQGIKNLPVGKAGELAGSDPDYSIRDLYNAIAEGNPPSWTLYIQVMTAEQAEKYKWNPFDVTKVWCHKDAPLIPVGRMVLDRNPVNYFAEVEQIAFSPAHMPPG-IEASPDKMLQGRLFSYDDTHRHRVGVNFQQLPVNRPH-VKVVNYQRDGPAAIDGNQAGAPNYFPNSFQGPQIQPSVAHSSFGVKGDAVRIETHD-DDNFSQAGTFWSDVLNDEERQRLVENIAGHLKDAK-EFIQERVVANFSKAHPDYGKRIEELLKKYKA--

>Ephydatia.muelleri_24_CAT

AENRSNCANQLIDHAHSSADADPITTSHGCPVDSKTASLTVGERGLIPIQDFTFLDEMAHFDRERIPERVVHAKGAGAFGYFEVTHDITKYCKAKIFSHVGKKTPIAIRFSTVGGENGSADTVRDPRGFAIKFYTEEGNWDLVGNNTPIFFIRDPILFPSFIHTQKRNPATHLKDPDMFWDFIT-LRPETTHQVSFLFSDR-GIPDGYRHMNGYGSHTFKLVNKAGEAVYCKFHFKTDQGIKNLSVQKAGELSMNDPDYSIRDLYESIATGNFPSWTLFLQVMTFEQAEKFRLNPFDLTKVWPHSEYPLIPVGKLVLNRNPKNYFAEVEQIAFCPAHLVPG-VEPSPDKMLQGRLFSYSDTHRHRLGSNYHMIPVNCPYATKANTYHRDGSMCVDSNQGGAPNYYPNSFQGPVDSQIHALSKTTVTGDVKRYNTAD-HDNFSQVTNFWTKVLSEEEKTRLVENIAGHLKDAT-EFIQKRAVQNFTAVHRDYGGRLNLLLQQYKKKV

>Sycon.ciliatum_31_CAT

MAAKSKCPNQLQDYAKGKKNSDKLTTSAGCPVDSKTSTMTVGPRGPVLLQDQVFLDDMAHFDRERIPERVVHAKGAGAFGYFEVTDDITKYCKAKIFEQVGKRTPMAVRFSTVGGESGSADTVRDPRGFAVKFYTEEGNWDLVGNNTPIFFIRDPILFPSFIHTQKRNPQTHLKDADMFWDFIS-LRPETTHQVSFLFSDR-GIPNGYRFMNGYGSHTFKLVNARNEPVYCKFHYKTDQGIQNLPVDQAGSLASSDPDYSIRDLFESIAQGNFPSWTMYVQVMTYEQAEKWKWNPFDLTKIWPHKEFPLIKAGRFVLNRNPKNYFAEVEQLAFSPAHLVPG-IEPSPDKMLQGRLFSYTDTHRHRLGANYQQLPVNCPFATRPSNYQRDGPMAMGDNQGGAPNYYPNSFSGPRDDPKYAACPERVSGDVKKYNTAD-DDNFTQAGLFYRSVLNEAEKQRLVENIAGHMKDAK-PFIQQRAVRNFAMADPDYGARISRLLAKYQSSS

>Oscarella.carmela_29_CAT

MSSRTKASEQLSDYASFRSTPENCTTGDGTPVDVKTATMTAGPRGPVLLQDYNFLDEMAHFDRERVPERIVHAKGAGAFGYFEVTHDITRFCKAKVFESVGKKTPLAIRFSTVGGESGSADTVRDPRGFAVKFYTEEGNWDLVGNNTPIFFIRDPILFPSFIHTQKRNPVTHLKDPDMFWDFIS-LRPETTHQVSFLFSDR-GIPDGYRHMNGYGSHTFKMVNKKDEPVYAKFHYKTDQGIQCLPVDKAGDLSGSDPDYGIRDLYNAIAQGKFPSWTLYIQVMTFEQAEKHRWNPFDVTKVWPHSEFPLIQVGRITLNRNPVNYFAEVEQIGFSPAHMPPG-IEPSPDKMLQGRLFSYDDTHRHRIGANYLQLPVNCSQNVKVRNYQRDGPMTIDNNQAGAPNYFPNSFQGPAEKRQAGPTKFHVSGDVARYNSAD-DDNFTQAGTFWTKVLNEEERQRLVENIAGHLKDAK-DFIQARAVRNFSQAHPDYGRRIADLLAKYSQRL

>Danio.rerio_20_CAT

ADDREKSTDQMKLWKEGGQRPDVLTTGAGVPIGDKLNAMTAGPRGPLLVQDVVFTDEMAHFDRERIPERVVHAKGAGAFGYFEVTHDITRYSKAKVFEHIGKTTPIAVRFSTVAGEAGSSDTVRDPRGFAVKFYTDEGNWDLTGNNTPIFFIRDTLLFPSFIHSQKRNPQTHLKDPDMVWDFWS-LRPESLHQVSFLFSDR-GIPDGYRHMNGYGSHTFKLVNAQGQPVYCKFHYKTNQGIKNIPVEEADRLAATDPDYSIRDLYNAIANGNFPSWTFYIQVMTFEQAENWKWNPFDLTKVWSHKEFPLIPVGRFVLNRNPVNYFAEVEQLAFDPSNMPPG-IEPSPDKMLQGRLFSYPDTHRHRLGANYLQLPVNCPYRTRVANYQRDGPMCMHDNQGGAPNYYPNSFSAPDVQPRFLESKCKVSPDVARYNSAD-DDNVTQVRTFFTQVLNEAERERLCQNMAGHLKGAQ-LFIQKRMVQNLMAVHSDYGNRVQALLDKHNAEG

>Danio.rerio_21_CAT

ADDREKSTDQMKLWKEGGQRPDVLTTGAGVPIGDKLNAMTAGPRGPLLVQDVVFTDEMAHFDRERIPERVVHAKGAGAFGYFEVTHDITRYSKAKVFEHIGKTTPIAVRFSTVAGEAGSSDTVRDPRGFAVKFYTDEGNWDLTGNNTPIFFIRDTLLFPSFIHSQKRNPQTHLKDPDMVWDFWS-LRPESLHQVSFLFSDR-GIPDGYRHMNGYGSHTFKLVNAQGQPVYCKFHYKTNQGIKNIPVEEADRLAATDPDYSIRDLYNAIANGNFPSWTFYIQVMTFEQAENWKWNPFDLTKVWSHKEFPLIPVGRFVLNRNPVNYFAEVEQLAFDPSNMPPG-IEPSPDKMLQGRLFSYPDTHRHRLGANYLQLPVNCPYRTRVANYQRDGPMCMHDNQGGAPNYYPNSFSAPDVQPRFLESKCKVSPDVARYNSAD-DDNVTQVRTFFTQVLNEAERERLCQNMAGHLKGAQ-LFIQKRMVQNLMAVHSDYGNRVQALLDKHNAEG

>Danio.rerio_19_CAT

ADDREKSTDQMKLWKEGGQRPDVLTTGAGVPIGDKLNAMTAGPRGPLLVQDVVFTDEMAHFDRERIPERVVHAKGAGAFGYFEVTHDITRYSKAKVFEHIGKTTPIAVRFSTVAGEAGSSDTVRDPRGFAVKFYTDEGNWDLTGNNTPIFFIRDTLLFPSFIHSQKRNPQTHLKDPDMVWDFWS-LRPESLHQVSFLFSDR-GIPDGYRHMNGYGSHTFKLVNAQGQPVYCKFHYKTNQGIKNIPVEEADRLAATDPDYSIRDLYNAIANGNFPSWTFYIQVMTFEQAENWKWNPFDLTKVWSHKEFPLIPVGRFVLNRNPVNYFAEVEQLAFDPSNMPPG-IEPSPDKMLQGRLFSYPDTHRHRLGANYLQLPVNCPYRTRVANYQRDGPMCMHDNQGGAPNYYPNSFSAPDVQPRFLESKCKVSPDVARYNSAD-DDNVTQVRTFFTQVLNEAERERLCQNMAGHLKGAQ-LFIQKRMVQNLMAVHSDYGNRVQALLDKHNAEG

>Homo.sapiens_1_CAT

-------------------------------------------------QDVVFTDEMAHFDRERIPERVVHAKGAGA----------------------------------------------------------------------------------------------------------------------------------------------------------------------------------------------------------------------------------------------------------------------------------------------------------------------------------------------------------------------------------------------C---------------------------------------------

>Xenopus.tropicalis_39_CAT

ADKRDNAADQMKLWKESGQKPDVLTTGGGNPISDKLNLLTVGPRGPLLVQDVVFTDEMAHFDRERIPERVVHAKGAGAFGYFEITHDITKYSKAKVFEHIGKRTPIAIRFSTVAGESGSADTVRDPRGFAVKMYTEDGNWDLTGNNTPVFFIRDAMLFPSFIHSQKRNPQTHLKDPDMVWDFWA-LRPESLHQVSFLFSDR-GIPDGHRHMNGYGSHTFKLVNAKDEAVYCKFHYKTDQGIRNLTVEEANRLSASDPDYGIHDLYESIAAGNYPSWSFYIQVMTFQQAEKFKFNPFDLTKIWPHGDYPLIPVGKLVLNRNPTNYFAEVEQLAFDPSNMPPG-IEPSPDKMLQGRLFSYPDTHRHRLGPNYLQLPVNCPYRTRVANYQRDGPMCFTDNQGGAPNYYPNSFCAPENQPQVREHRFHVSADVARYNSAD-EDNVSQVRDFYVKVLSEEQRLRLCENIAGHLKDAQ-LFIQKRAVKNFTDVHPEYGARIQALLDKYNAEG

>Xenopus.tropicalis_42_CAT

AGNKEKAPTQMNQWKEAGQKPSVLTTGAGHPVGDKLNLLTAGPRGPLLVQDVVFTDEMAHFDRERIPERVVHAKGAGAFGYFEVTHDITQYCKANVFGKVGKRTPVAARFSTVAGEAGSPDTIRDPRGFAVKMYTEEGNWDLTGNNTPIFFIRDAILFPSFVHSQKRNPQTHMKDPDMVWDFWS-LRPESLHQVSFLFSDR-GIPDGHRHMNGYGSHTFKLVNCKDEAVYCKFHFKTDQGIRNLTLEKAEQLAASDPDYGIRDLYEAIAAGNYPSWTFYIQIMTFEQAEKFPFNPFDVTKVWPHGDYPLIPVGKMVLSRNPTNYFAEVEQLAFDPSNMPPG-IEPSPDKMLQGRLFSYPDTHRHRLGANYLQLPVNCPYKARVANYQRDGPMCFTDNQGGAPNYYPNSFSAPEQQPQFREHRFRVSADVERYNSAN-DDNVTQVREFYLKVLNEEERQRLCENIVGHLKECQ-LFIQKRTVKNFSDVHPDYGSRIQALLDKHNAKC

>Homo.sapiens_2_CAT

ADSRDPASDQMQHWKEQAQKADVLTTGAGNPVGDKLNVITVGPRGPLLVQDVVFTDEMAHFDRERIPERVVHAKGAGAFGYFEVTHDITKYSKAKVFEHIGKKTPIAVRFSTVAGESGSADTVRDPRGFAVKFYTEDGNWDLVGNNTPIFFIRDPILFPSFIHSQKRNPQTHLKDPDMVWDFWS-LRPESLHQVSFLFSDR-GIPDGHRHMNGYGSHTFKLVNANGEAVYCKFHYKTDQGIKNLSVEDAARLSQEDPDYGIRDLFNAIATGKYPSWTFYIQVMTFNQAETFPFNPFDLTKVWPHKDYPLIPVGKLVLNRNPVNYFAEVEQIAFDPSNMPPG-IEASPDKMLQGRLFAYPDTHRHRLGPNYLHIPVNCPYRARVANYQRDGPMCMQDNQGGAPNYYPNSFGAPEQQPSALEHSIQYSGEVRRFNTAN-DDNVTQVRAFYVNVLNEEQRKRLCENIAGHLKDAQ-IFIQKKAVKNFTEVHPDYGSHIQALLDKYNAEK

>Homo.sapiens_3_CAT

ADSRDPASDQMQHWKEQAQKADVLTTGAGNPVGDKLNVITVGPRGPLLVQDVVFTDEMAHFDRERIPERVVHAKGAGAFGYFEVTHDITKYSKAKVFEHIGKKTPIAVRFSTVAGESGSADTVRDPRGFAVKFYTEDGNWDLVGNNTPIFFIRDPILFPSFIHSQKRNPQTHLKDPDMVWDFWS-LRPESLHQVSFLFSDR-GIPDGHRHMNGYGSHTFKLVNANGEAVYCKFHYKTDQGIKNLSVEDAARLSQEDPDYGIRDLFNAIATGKYPSWTFYIQVMTFNQAETFPFNPFDLTKVWPHKDYPLIPVGKLVLNRNPVNYFAEVEQIAFDPSNMPPG-IEASPDKMLQGRLFAYPDTHRHRLGPNYLHIPVNCPYRARVANYQRDGPMCMQDNQGGAPNYYPNSFGAPEQQPSALEHSIQYSGEVRRFNTAN-DDNVTQVRAFYVNVLNEEQRKRLCENIAGHLKDAQ-IFIQKKAVKNFTEVHPDYGSHIQALLDKYNAEK

>Xenopus.tropicalis_41_CAT

-----------------AQTQPILTTGAGIPAGDKLNVLTAGPRGPMLMQDVVFVDEMAHFDRERIPERVVHAKGAGAFGYFEVTHDITKYCRAKVFERVGKRTDVAVRFSTVAGEAGSADSVRDPRGFALKFYTDDGIWDLVGNNTPIFFIRDPMMFPSFIHSQKRNPQTHLKDPDTVWDFWS-LRPETLHQVTFLFSDR-GIPDGHRHMNGYGSHTFKLVNAEGKAVYCKFHYKTDQGIKNLSVEEADRLVVSDPDYGIRDLFQSIAKKNFPSWTMYLQVMTFEEAEKCPFNPFDLTKVWPHRDYPLIPVGKLVLNRNPENYFAEVEQIAFDPSNMPPG-IEASPDKMLQGRLFSYPDTHRYRLGPNYLHLPVNCPRGVQVAHYQRDGPMCMFNNPSHMPNYYPNSFSSPRDDPKCKDSTFVAAGDVGRHDCSE-EDNVSQVRMFYTQTLTEGERKRLCENLARHLSEAQ-IFIQERAVKNFTDVHPDYGARIKSLLDKYNGDG

>Xestospongia.bergquistia_44_CAT

MAKRPKAFSQLEEYGSSQKRQEPLTTSHGHPVDFKTGVQTFGPRGPMLMQDFVYMDEMAHFDRERIPERVVHAKGAGAFGYFEVTHDITKYCSAKIFSEIGKKTPLVVRFSTVGGESGSADTVRDPRGFAVKFYTEDGNWDLVGNDTPIFFIRDPFLFPSFIHTQKRNPTTHLKDPDMFWDFIS-LRPETTHQVSFLFSDR-GIPDGYRHMNGYGSHTFKLVNKDGEAVYCKFHYKTDQGIKNIPVDEAGRLAGSNPDYSIQDLYEAIATGNFPSWTLYIQVMTFKEAETHRFNPFDLTKVWPQKEFPLIPVGKIVLDRNPANYFADVEQAAFCPAHMPPG-IEASPDKMLQGRLFSYSDTHRHRLGTNYHQIPVNCPYATKHRSYLRDGFMCVDGNQDGAPNYYPNSFNGPVDEGKHDIPIMKPASDVARYNSAD-EDNFTQVGIFYREVLSEAERIRLTENIAGHLKNAK-EFIQKRAVENFRKADPDYGERIAKLLEQYKTQE

>Trichoplax.adhaerens_CAT

KNSRDKAADQLKEFRE-NNDPTTLTTSHGAPAESITDSVTVGPRGPILLQDITLIDHMAHFDRERIPERVVHAKGAGAFGYFEVTHDITKYCKAKVFENVGKRTPMAVRFSTVGGESGSADTARDPRGFALKFYTEEGNWDLVGNNTPIFFIRDPILFPSFIHTQKRNPVTHLKDPDMFWDFIS-LRRETTHQVSFLFTDR-GTPDGYRHMNGYGSHTFKLVNADNEAVYCKFHLKTDQGIRNLTGEKAQEISGLDPDYAMRDLYNAIASGNSPSWTMYLQIMTFEQAEKWRFNPFDVTKIWPHSEFPLIPVGKMVLNRNPKNYFAEVEQIAFAPANFVPG-IEPSPDKMLQGRLFSYNDTHRHRLGTNYAQLPVNCPYATKVGNYQRDGPQTFNDNHSGVPNYYPNSFNGPIANVNATPHTVQVIGDVKRYNTAD-DDNFTQVTVFWRKVLNEQERTALVNNIVGHLCMAQ-SFLQERAIENFSKVDPEYGRRIREGLNRKEEGA

>Ephydatia.muelleri_25_CAT

ADTRPKCQRQLEEYYSRQKAPEILTTSHGHPIDDKKATMTVGARGGVALEDFVFLDEMAHFDRERIPERVVHAKGAGAFGYFEVTHDITKYTKAKVFSQIGKKTPLAVRFSTVGGENGSADTVRDPRGFAVKFYTEDGNWDLVGNNTPIFFIRDPIFFPSFIHTQKRNPATHLKDPDMFWDFIS-LRPETTHQVSFLFSDR-GIPDGYRHMNGYGSHTFKLVNSDGTPVYCKFHYKTDQGIKNLPVDQANKLAGDNPDYAIEDLYESIANGKYPSWTMYIQVMTFEQAEKVAFNPFDLTKVWSHADFPLIPVGKLVLDRNPRNYFAEVEQLAFSPAHMPPG-IEASPDKMLQGRLFSYSDTHRHRLGPNSHLIPVNSAQCCRPRNYQRDSFMCTDGNQSDAPNYFPNSFNGPKDNPAVACSKHSFSGDVQRHRSDD-EDNFTQAGIFYRKVLTDDQRTRLVEKHSWSCEVCC-------------SIHP-------------GACG

>Branchiostoma.floridae_9_CAT

MAGRDKAGNQLEEYKKQNGNASTVTTGTGAPVDNKLAVLTVGPRGPMLMQDFTYMDEMAHFNRERIPERVVHAKGHGAFGYFECTHDISQYCKAKPFEHVGKRTPLGIRFSTVGGESGSADTARDPRGFAVKMYTEDGNWDLVGNNTPIFFIRDPILFPSFIHTQKRNPATHLKDPDMFWDFIS-LRPETCHQVSFLFSDR-GTPNGYRHMNGYGSHTFKMVNNNNEAVYCKFHWKTDQGIKNLTRQQADDLAGSDPDYAGRDLFNAIAEGNYVST--------------------------------------------------------------------------------------------------------------------------------------------------------------------------------------------------------------------------------------

>Drosophila.melanogaster_22_CAT

MAGRDAASNQLIDYKNQTVSPGAITTGNGAPIGIKDASQTVGPRGPILLQDVNFLDEMSHFDRERIPERVVHAKGAGAFGYFEVTHDITQYCAAKIFDKVKKRTPLAVRFSTVGGESGSADTARDPRGFAVKFYTEDGVWDLVGNNTPVFFIRDPILFPSFIHTQKRNPQTHLKDPDMFWDFLT-LRPESAHQVCILFSDR-GTPDGYCHMNGYGSHTFKLINAKGEPIYAKFHFKTDQGIKNLDVKTADQLASTDPDYSIRDLYNRIKTCKFPSWTMYIQVMTYEQAKKFKYNPFDVTKVWSQKEYPLIPVGKMVLDRNPKNYFAEVEQIAFSPAHLVPG-VEPSPDKMLHGRLFSYSDTHRHRLGPNYLQIPVNCPYKVKIENFQRDGAMNVTDNQDGAPNYFPNSFNGPQECPRRASSCCPVTGDVYRYSSGDTEDNFGQVTDFWVHVLDKCAKKRLVQNIAGHLSNAS-QFLQERAVKNFTQVHADFGRMLTEELNLAKSSK

>Lingula.anatina_26_CAT

------------------------------------------------MQDFVFMDEMAHFQRERIPERVVHAKGAGAFGFFEVTHDISKYCKAKVFEHIGKRTPVAVRFSTVGGESGSADTARDPRGFAVKMYTDEGNWDCVGNNTPIFFIRDPIFFPSFIHTQKRNPVTHCKDPDMFWDFIT-LRPESTHQVSFLFSDR-GTPDGYRQMNGYGSHTFKLVNKDGEAVYCKFHYKTDQGIKCLMADQAGELAGSDPDYAIRDLYNNIAAGNYPSWTWYIQVMTFEEAEKFRWNPFDLTKVWPQGEFPLIPVGRMVLNRNPKNYFAEVEQIAFSPAHLVPG-IEASPDKMLQGRLFSYSDTHRHRLGTNYLQIPVNCPFNTRVKNYQRDGPQCVTDNQEGAPNYFPNSFNGPLDDKRHLESVFQTTGDVKRYNTRD-DDNFSQVGLFWRNVLKPDERTRLVENIANHLKDAQ-EFIQQRAVKNFGQCDPEYGRRIQELLDQFKAKK

>Amphimedon.queenslandica_8_CAT

SSKRPACFSQLEEYAKKTQPGEVLTTTHGNPIDFKTAIQTFGPRGPMLMQDGVYLDEMAHFDRERIPERVVHAKGAGAYGVFEVTHDITKYCCAKLFSEVGKKTDLFIRFSTVGGESGSADTARDPRGFAVKFYTEDGNWDLVGNNTPIFFIRDPFLFPSFIHTQKRNPVTHLKDPDMFWDFIS-LRPETTHQVSFLFSDR-GIPDGYRHMNGYGSHTFKLVNSKGEPVYCKFHYKTDQGIKNMPVGKAAELAGTNPDYSIQDLYEAIATGNFPSWTLSIQVMTYEQAEKCSFNPFDLTKVWPHADYPLIPVGKITLNRNPSNYFFDVEQSAFSPAHMPPG-IEASPDKMLQGRLFSYDDTHFHRLGPNFQMIPVNCPYAGKPRNYVRDGPMCVDGNQGGAPNYYPNSFNGPKDMGKHDVTIFPPAGDVKRYNAAD-DDNFSQVGIFYNKVLNEEERTRLAQNIAGHMKNAS-PKIQERAIANFSKADPDYGARIKKYISQ-----

>Nematostella.vectensis_27_CAT

-----------------------------------------------------------------------------------------------------------------------------------------------------------------------------QDANMFWDFIT-LRPETTHQTSFLFSDR-GIPDGFRHMNGYGSHTFKMVNSKGKAVYCKFHVKTDQGIKNCPVERATELAGTDPDYSTRDLYNAIAEGNY-----------------------------------------------------------------------------------------------------------------------------------------------------------------------------------------------------------------------------------------

>Capitella.teleta_17_CAT

MANRDKASEQLNEYKQSNGTPGVLTTATGAPIGNKTAIQTVGPRGPALLQDFVFQDEMSHFGRERIPERVVHAKGAGAFGFFEVTHDITKYSKAKVFEHIGKKTPIVARLSTVGGEKGSADTARDPRGFAVKFYTDEGNWDLVGNNTPIFFIRDPMLFPSFIHTQKRNPKTNLKDPDAFWDFLT-LRPESCHQVSFLFSNR-GTPDGYRNMNGYGSHTFKLVNKEGVAHYCKFHYKTNQGIKNLTGAQADALAGSDPDYATRDLYNAIAEGNFPSYTLFIQVMTFEEAEKHRFNPFDLTKVWSHKEFPLIPVGRLTFNRNPKNYFAEVEQVAFSPAHMVPG-IEASPDKMLQGRLYSYSDTHRHRLGTNYQQIPVNCPFSTRARNYQRDGPQNVDDNQEGAPNYFPNSFGGPQDSPAFLEHETTFPGDVARYNTKD-DDNFTQVGIFWRETLTEEDRKHLIINMSGHLKNAQ-EFLQKRAVANFSKCDPEYGRRLQEALDQHKKDA

>Strongylocentrotus.purpuratus_30_CAT

--------------------------------------MTAGARGPVLIQDFVFTDEMSHFGRERIPERVVHAKGAGAFGYFETTHDISKYCKAAPFESVGKKTPVAIRFSTVGGESGSADTARDPRGFAVKFYSEDGNWDLVGNNTPIFFIRDPMFFPSFIHTQKRNPVTHLKDPDMFWDFIT-LRPEATHQVSFLFSDR-GTPDGYRHMNGYGSHTFKLRNKDGEYVFCKFHFKCDQGIKNLNRHRAGDLSATDPDYAIRDLYNSIATGNFPSWSLHIQVMTQEQADKHRDNPFDLTK----------------------------------------------------GRLFSYSDTHRHRLGTNYLQIPVNCPFAARTRSYQRDGPQCVTDNQGGAPNYFPNSFTGPTDSKSYEQTKFTCPGEAARYETGD-DDNYTQAGIFWRDVLSEADREATVDNMASHIKDAA-EYLQKRTVVQWGKCDEDWGKRLEAKLAEYKTQA

>Tethya.wilhelma_37_CAT

DAKRPKHATQLEEYAKEQKTRPVMTTGHGIPIDSKTASMTVGPRGPITLQDVFYLDEMSHFDRERIPERVVHAKGAGAFGYFEVTQDITKYCKADLFSEVGKKTPIGIRFSTVGGESGSADTVRDPRGFAVKFYTQEGNWDLVGNNTPIFFIRDPLHFPNFIHTQKRNPVTHLKDHDMFWDFLT-LRQESAHQVSFLFSDR-GIPDGYRHMNGYGSHTFKLVNKDGEPVYCKFHYKTNQGIKNILPEEAEKMAGRDPDYAIRDLYDAIANKDFPSWTLSIQVMTFEQAEKTSFNPFDLTKIWPHKDYPLIEVGKMVLDRNPTNYFCEIEQIAFSPSNMVPG-VEPSPDKMLQGRLFSYPDTHLYRLGPNYHQLPVNCPYMTKCRNYQRDGNMPLEGNQAGAPNYFPNSFQGPVDDRKYRWSKTTQTCDIDRYETAD-EDNYSQVGNFYRNVLSEVEKDRLTSNIAGHVKGAA-KFIQERVVEMFTKCDPDYGQRIAKKLQG-----

>Caenorhabditis.elegans_15_CAT

M-PNDPSDNQLKTYKETYPKPQVITTSNGAPIYSKTAVLTAGRRGPMLMQDVVYMDEMAHFDRERIPERVVHAKGAGAHGYFEVTHDITKYCKADMFNKVGKQTPLLVRFSTVAGESGSADTVRDPRGFSLKFYTEEGNWDLVGNNTPIFFIRDAIHFPNFIHALKRNPQTHMRDPNALFDFWM-NRPESIHQVMFLYSDR-GIPDGFRFMNGYGAHTFKMVNKEGNPIYCKFHFKPAQGSKNLDPTDAGKLASSDPDYAIRDLFNAIESRNFPEWKMFIQVMTFEQAEKWEFNPFDVTKVWPHGDYPLIEVGKMVLNRNVKNYFAEVEQAAFCPAHIVPG-IEFSPDKMLQGRIFSYTDTHYHRLGPNYIQLPVNCPYRSRAHTTQRDGAMAY-ESQGDAPNYFPNSFRGYRTRDDVKESTFQTTGDVDRYETGD-DHNYEQPRQFWEKVLKEEERDRLVGNLASDLGGCL-EEIQNGMVKEFTKVHPDFGNALRHQLCQKKH--

>Caenorhabditis.elegans_16_CAT

M-PNDPSDNQLKTYKETYPKPQVITTSNGAPIYSKTAVLTAGRRGPMLMQDVVYMDEMAHFDRERIPERVVHAKGAGAHGYFEVTHDITKYCKADMFNKVGKQTPLLVRFSTVAGESGSADTVRDPRGFSLKFYTEEGNWDLVGNNTPIFFIRDAIHFPNFIHALKRNPQTHMRDPNALFDFWM-NRPESIHQVMFLYSDR-GIPDGFRFMNGYGAHTFKMVNKEGNPIYCKFHFKPAQGSKNLDPTDAGKLASSDPDYAIRDLFNAIESRNFPEWKMFIQVMTFEQAEKWEFNPFDVTKVWPHGDYPLIEVGKMVLNRNVKNYFAEVEQAAFCPAHIVPG-IEFSPDKMLQGRIFSYTDTHYHRLGPNYIQLPVNCPYRSRAHTTQRDGAMAY-ESQGDAPNYFPNSFRGYRTRDDVKESTFQTTGDVDRYETGD-DHNYEQPRQFWEKVLKEEERDRLVGNLASDLGGCL-EEIQNGMVKEFTKVHPDFGNALRHQLCQKKH--

>Caenorhabditis.elegans_11_CAT

TKPGPMAEDQLKAYRDRNQEPHLLTTSNGAPIYSKTAVLTAGRRGPMLMQDIVYMDEMAHFDRERIPERVVHAKGGGAHGYFEVTHDITKYCKADMFNKVGKQTPLLVRFSTVAGESGSADTVRDPRGFSLKFYTEEGNWDLVGNNTPIFFIRDAIHFPNFIHALKRNPQTHMRDPNALFDFWM-NRPESIHQVMFLYSDR-GIPDGFRFMNGYGAHTFKMVNKEGNPIYCKFHFKPAQGSKNLDPTDAGKLASSDPDYAIRDLFNAIESRNFPEWKMFIQVMTFEQAEKWEFNPFDVTKVWPHGDYPLIEVGKMVLNRNVKNYFAEVEQAAFCPAHIVPG-IEFSPDKMLQGRIFSYTDTHYHRLGPNYIQLPVNCPYRSRAHTTQRDGAMAY-ESQGDAPNYFPNSFRGYRTRDDVKESTFQTTGDVDRYETGD-DHNYEQPRQFWEKVLKEEERDRLVGNLASDLGGCL-EEIQNGMVKEFTKVHPDFGNALRHQLCQKKH--

>Caenorhabditis.elegans_12_CAT

-----MAEDQLKAYRDRNQEPHLLTTSNGAPIYSKTAVLTAGRRGPMLMQDIVYMDEMAHFDRERIPERVVHAKGGGAHGYFEVTHDITKYCKADMFNKVGKQTPLLVRFSTVAGESGSADTVRDPRGFSLKFYTEEGNWDLVGNNTPIFFIRDAIHFPNFIHALKRNPQTHMRDPNALFDFWM-NRPESIHQVMFLYSDR-GIPDGFRFMNGYGAHTFKMVNKEGNPIYCKFHFKPAQGSKNLDPTDAGKLASSDPDYAIRDLFNAIESRNFPEWKMFIQVMTFEQAEKWEFNPFDVTKVWPHGDYPLIEVGKMVLNRNVKNYFAEVEQAAFCPAHIVPG-IEFSPDKMLQGRIFSYTDTHYHRLGPNYIQLPVNCPYRSRAHTTQRDGAMAY-ESQGDAPNYFPNSFRGYRTRDDVKESTFQTTGDVDRYETGD-DHNYEQPRQFWEKVLKEEERDRLVGNLASDLGGCL-EEIQNGMVKEFTKVHPDFGNALRHQLCQKKH--

>Caenorhabditis.elegans_13_CAT

M-PNDPSDNQLKTYKETYPKPQVITTSNGAPIYSKTAVLTAGRRGPMLMQDVVYMDEMAHFDRERIPERVVHAKGAGAHGYFEVTHDISKYCKADIFNKVGKQTPLLIRFSTVGGESGSADTARDPRGFAIKFYTEEGNWDLVGNNTPIFFIRDPIHFPNFIHTQKRNPQTHLKDPNMIFDFWL-HRPEALHQVMFLFSDR-GLPDGYRHMNGYGSHTFKMVNKDGKAIYVKFHFKPTQGVKNLTVEKAGQLASSDPDYSIRDLFNAIEKGDFPVWKMFIQVMTFEQAEKWEFNPFDVTKVWPHGDYPLIEVGKMVLNRNPRNYFAEVEQSAFCPAHIVPG-IEFSPDKMLQGRIFSYTDTHFHRLGPNYIQLPVNCPYRSRAHNTQRDGAMAY-DNQQHAPNFFPNSFNYGKTRPDVKDTTFPATGDVDRYESGD-DNNYDQPRQFWEKVLDTGARERMCQNFAGPLGECH-DFIIKGMIDHFSKVHPDFGARVKALIQKQARSH

>Caenorhabditis.elegans_14_CAT

M-PNDPSDNQLKTYKETYPKPQVITTSNGAPIYSKTAVLTAGRRGPMLMQDVVYMDEMAHFDRERIPERVVHAKGAGAHGYFEVTHDISKYCKADIFNKVGKQTPLLIRFSTVGGESGSADTARDPRGFAIKFYTEEGNWDLVGNNTPIFFIRDPIHFPNFIHTQKRNPQTHLKDPNMIFDFWL-HRPEALHQVMFLFSDR-GLPDGYRHMNGYGSHTFKMVNKDGKAIYVKFHFKPTQGVKNLTVEKAGQLASSDPDYSIRDLFNAIEKGDFPVWKMFIQVMTFEQAEKWEFNPFDVTKVWPHGDYPLIEVGKMVLNRNPRNYFAEVEQSAFCPAHIVPG-IEFSPDKMLQGRIFSYTDTHFHRLGPNYIQLPVNCPYRSRAHNTQRDGAMAY-DNQQHAPNFFPNSFNYGKTRPDVKDTTFPATGDVDRYESGD-DNNYDQPRQFWEKVLDTGARERMCQNFAGPLGECH-DFIIKGMIDHFSKVHPDFGARVKALIQKQARSH

>Branchiostoma.floridae_10_CAT

---------------------------------------------------------------------------------------------------------------------------------------------------------------------------------------------------------------------------------------------------------------------------------PSWTLKIQVMTFEEAEKFRFNPFDLTKVWPQGEFPLIPVGKMVLNRNPKNYFAEVEQIAFSPIHMVPG-IEASPDKMLQGRLFSYSDTHRHRLGSNYLQIPVNCPYRARVTNYQRDGPQCVDDNQAGAPNCYPNSFSGPKQKETITPPAIKTTGDLQRYNTAD-EDNFTQVGTFWRNVLSEYDREHLVDNLASHMTAAQ-EFLQKRAVKNFSQCDPEYGRRLQEKLDKYNAAK

>Amphimedon.queenslandica_5_CAT

---------------------------------------------------------------------------------------------------------------------------------------------------------------------------------MFWDFIS-LRPETTHQVSFLFSDR-GIPDGYRHMNGYGSHTFKLVNNEGEPVYCKFHYKTDQGIANLSVEKAGILAGSDPDYAIKDLYDAIAAKNYPSWTLYIQVMTFEQAKEFEWNPFDLTKIWPQKEFPLIPVGRMVLNRNPANYFAEVEQLAFSPAHMVAG-IEPSPDKMLQGRLFSYDDTHRHRLGPNYHQIPVNCPYATRTRNYQRDGPMTVDGNQGGAPNYFPNSFSGPVDNPEYTISPITSTCDVKKYNTRD-DDNFSQVKNFWLKVLTVEEQSRLVFNIASHLKDAQ-PFIQSRVIRNFSSVHPDYGSRISDLLQQFKKKK

>Tethya.wilhelma_38_CAT

--------------------------------------------------------------------------------------------------------------------------------------------------------------------------------------------------------------------------------------------------------------------------------------------------------------------------MILNRNPTNYFCEVEQIAFCPAHLVPG-IEPSPDKMLQGRLFSYSDTHRHRLGANYHQIPVNCPYATRCKNYQRDGPQQ--------------------------------------------------------------------------------RWIIKQLVEW-----------------------

>Ciona.intestinalis_18_CAT

M-GRTKSDNQLKEYAEKNKDKTVLMTGTGAPIEDKLNVLTVGERGPLLMQDFTFTDEMAHFNRERIPERVVHAKGGGAKGYLEITHDISNFCKADIFSSIGKRTPLAVRFSTVGGESGSADSARDPRGFAIKFYTEEGIWDLVGNNTPIFFIRDPIFFPSFIHTQKRNPQTHLKDPDMFWDFIS-LRPETTHQVSHILVMRVNTPQGVIHFNT----TDNWCNISITLYQCNSRFKTDQGIKNLTADEADTLAATDPDHAIRDLYNAIADGNNPSWTMYIQVMTYQQATTHKWNPFDLTKTWPQGEFPLIQVGKMVLNENPSNYFAEVEQIAFSPSHMVPG-IEASPDKMLQGRLFSYPDTHRHRLGSNYLQIPINCPFNVRVNNYQRDGPQCVTDNSKGAPNYYPNSFNGPLDGASRKQTSLHPASDVKKYNSAD-DDNFTQVGTFWRKVLNEAERKRLAENIGNHMKAAQ-PFLQKRAIANFAAADPEFGAMIQAVIDKAAAKI

>Sycon.ciliatum_32_CAT

MAGRCPAANQLLNYREEQKESTTTSTSWGAPVDIATASQTVGPRGPLLLQDANFIDNLAHFDRERIPERVVHAKGAGAFGELVITEDISKYTKALALQK-GTSTPVAVRFSTVGGEAGSADTVRDPRGFAVKHYTDDGVWDLVGNNTPIFFIRDPILFPNFIHTQKRNPRTHLKDPDMFWDFIS-LRPETTHQVSFLFSDR-GIPDGYRHMNGYGSHTFKLVNSEGVAHYCKFHYKTSES-KNLLPNKAGPISGDDPDYAIRDLYNAIERKEFPTWKFQIQVMTFEQAETYKYNPFDLTKVWSHKDFPLIDVGTMTLNRNPEDYFSEVEQIAFSPSHMVPG-IEPSPDKMLQGRLFSYTDTHRHRLGANYQHLPVNASKKTPQANSHRDGPMCM-FNQAGAPNYFPNSFKGPVDRPDARHSKISYSGDVEKYNSAD-EDNFSQVGVFYREVLNAEERQRLVENIAGHLKDAL-PEIQKRTVANFSQADADYGAGIQKLLDGYNK--

>Drosophila.melanogaster_23_CAT

MCSRDTASNQLIDYKNDSEVQREITTSSGTPVGVKDAIQTVGPRGPALLQDFQFLDEVMHFDSERIPERVAYAKGAGAFGYFECTHDISKFCAASIFDKVRKRTAVAMRFSVACGEQGSADTVREQRGFAVKFYTDDGIWDIVGCNMPVHYVRDPMLFPSLVHAQKRNPQTHLKDPDMFWDFMT-LRPETLHALLMYFSDR-GTPDGYRHLHGYGVHTYRMINASGETQYVRFHFKTDQGIKNLDARRCEELMSHDPDYAIRDLYNSIKKGNYPSWSMYIQVMLNEEAKKCRFNPFDVTKVWPQKDFPLLPVGKIVLDRNPTNYFTEVEQLAFSPAHMVPG-IEPSPDKMLQGRLFAYGDSQRHRLGVNYMQIPVNCPYRVNVRNFQRDGAMTVTDNQNGAPNYFPNSFCGPRESPRLGQTCCPLSGDVYRFMSGDTEDNFSQVTDFWTYTLDNCGRKRLVRNLSEHLTEAS-QFLQERAVKLFTMVHSDFGRLMTEALNTARISK

>Xenopus.tropicalis_40_CAT

MAGR---------YQTHEPELSLLTTASGVPIGDKRNSLTVGPRGPLLIQDAAFMEEMAHFNRERIPERVVHARGAGAFGYFEVTNDITQYCKAKVFSHVGKKTPIAVRFSTTTGELGSNDTVREPHGFAIKFYTEEGNWDLVGNHTPAFFIKDPILFPSLAHAQKKNPQTHLKDPNMFWDFVS-LCPETLHEITHLFTDR-GLPDGYRHMHGFGNHAFKLVNADGKPVYCKFHYKTNQGIKNLSSEQAKVIAGSDPDHALRDLLEAIAKGDYPSWTFSIQIMTFEQAEKMPFNPFDVTKVWYQKEFPLIPVGKLVLNRNPTNYFADVEQIALEPKNLVPG-IEPSPDRVLQGRLFAYSDALRYRLGVNYTQIPVNRPQGVKVANYERDGHMVI-DNQGNAPSYYPNSFGGPKDKAEYKEMVFHVSGDVDRYHNAE-TDDNFQVRKFYQKVLNDKQKQELCQNIASSLTGAL-QFIQDKSVKNFAAIDPDYGARVQKELDKLRAAS

>Dictyostelium.discoideum_CAT_A

M------------------SAPVLTTSSGSPIDNNLNSMTAGVNGPILIQDFTLIDKLAHFDRERIPERVVHAKGAGAHGYFEVSSDVPKWCKAKFLNKVGKRTPIFTRFSTVGGEKGSSDSERDPRGFAVKFYTEEGNFDMVGNNTPVFFIRDPSKFPDFIHTQKRNPQTNCKDPNMFWDFLG-QTPESTHQVSILFSDR-GTPKSYRHMHGFSSHTLKFVNAQGKPYWVKLHFTSETGIQNYTAEEAAKMSMNDPDSATRDLFETIAKGGEPAWKVSIQLMEFEDALKYRFNPFDVTKIWSHKDYPLIQIGRMVLNRNPENYFAEVEQAAFSPSHMVPG-IEPSPDKMLQGRLFSYPDTHRHRLGVNYQQIPVNCPFAVKVKNYQRDGFMAVNGNGGKGPNYQPNSFGGPEPHPEFAQHKFDVSGFAARQPYNHPNDDFVQPGDLYR-LMSEDAKSRFVSNLVGHMSGVTIKEIQVRAVSNFYKADKDLGARLCKGLGIDVNDV

>Tethya.wilhelma_33_CAT

MADRTKCAKQLTDFSNRKRVPDVLTTSHGHPIDSKTATLTVGEKGPVLLQDFTFLDEMAHFDRERIPERVVHAKGAGAFGYFEVTHDITKYCKAKVFSHVGKRTPIAIRFSTVD--------------------------------------------------------------------------------------------------------------------------------------------------------------------------------------------------------------------------------------------------------------------------------------------------------------------------------------------------------------------------------------------------------

>Tethya.wilhelma_34_CAT

MADRTKCAKQLTDFSNRKRVPDVLTTSHGHPIDSKTATLTVGEKGPVLLQDFTFLDEMAHFDRERIPERVVHAKGAGAFGYFEVTHDITKYCKAKVFSHVGKRTPIAIRFSTVGHCISSN--------------------------------------------------------------------------------------------------------------------------------------------------------------------------------------------------------------------------------------------------------------------------------------------------------------------------------------------------------------------------------------------------

>Tethya.wilhelma_35_CAT

M-----------------------------------------------------------------------------------------------------------------------------------------------------------------------------DVDMFWDFIS-LRPETTHQVSFLFSDR-GIPDGYRHMNGYGSHTFKLVNKDDEAFYCKFHYKTDQGIKCLDVDKAAKLSGTDPDYGIKDLFEAIATGNFPSWTLYIQVMTFEQAENFKWNPFDLTKT----------------------------------------------------------------------------------------------------------------------------TCDVKRYNTAD-MDNFSQVTTFWRKVLNEQEKSRLVRNIAGHLKDAA-EFIQQRAVRNFMQVDPEYGGRIAKLLQEYKKQ-

>Tethya.wilhelma_36_CAT

M-----------------------------------------------------------------------------------------------------------------------------------------------------------------------------DVDMFWDFIS-LRPETTHQVSFLFSDR-GIPDGYRHMNGYGSHTFKLVNKDDEAFYCKFHYKTDQGIKCLDVDKAAKLSGTDPDYGIKDLFEAIATGNFPSWTLYIQVMTFEQAENFKWNPFDLTKT----------------------------------------------------------------------------------------------------------------------------TCDVKRYNTAD-MDNFSQVTTFWRKVLNEQEKSRLVRNIAGHLKDAA-EFIQQRAVRNFMQVDPEYGGRIAKLLQEYKKQG

>Phaeosphaeria.nodorum_1_CAT

MNG-TATGDFFRSYRNAKSQDTVYTTSNGVPYPHPYETQRVGENGPLLLQDHHLVDLLSHFDRERIPERVVHAKGAGAHGVYKTTDPLDDLCLADLFSKAGKECPISVRFSTVGGESGSHDCARDPRGFSVKFRTEEGNWDVVANNTPVFFLRD------------------------------------LHQFMVLFGDR-GIPKGYRKMHGYIGHTHKLVNKNGDWVYMQLHFKSQQGTDFITQEDSFNYG---PDFSTKDLYQAIEKGDFPKWDVKVQTMTAKEAEKQGINVFDLTHVWPQSQFPLRKVGEFTLNENPQNYFAEIEQIAFNPAHLVPG-IEPSADPVLQSRLFSYPDTHRHRIGVNYQQLPVNQPRVPYMANFQRDGSMAF-YNQGSRPNFF--SSIEPVRSVNIKVHGTFTGEAVTFLSEIR-PEDFNAPRALWEKVFDDGAKERFISNISGHMENCSDKEIIKRQISIFREVSEDLASRLEKATGVKGYAG

>Monosiga.brevicollis_CAT

------------------SKCPYTTDAAGHPLDDQNAALRAGSTGPLVV-DPRLYEHNAIFNRERIPERVVHANGAGAFGTFRVTKDVSQYTKASFLSSVGKETKIAARFSTVAGERGFADADRDVRGFGVRFYTDEGNFDLVGNNTPIFFVRDPNKFQDFIHSQKRNPQTDLRDWEARWDFWS-LSPESVHQVLILMSDR-GVPRTYRHEHGYGSHTFSLINDKDERVWCKWHMRTNQGYETLAEEEAQKVRGANPDSHKDDLFHAIERGEYPSWKVCLQIMTDEQAKKMDFDIFDLTKVWSHKEFPLIEVGEMTLNRNPQNYFAEVEQLALSPGNLVPG-IGASPDPMLQIRLIAYTDAANYRLGVNHYQLPVNAPVCPVSHKKYRDGLMNPSANSGGHPNYQPNSMDKYPTFGSWEPPLPLDQAVVQRKRQGQPDDVYFQPRALFQ-MFEKDHKERVYKNVAGTMKGIK-QEIIERQLEVFGKVDAELEQGIRVALEQEA---

>Arabidopsis.thaliana_CAT3

MDP--------YKYRPSSYNAPFYTTNGGAPVSNNISSLTIGERGPVLLEDYHLIEKVANFTRERIPERVVHARGISAKGFFEVTHDISNLTCADFLRAPGVQTPVIVRFSTVVHERASPETMRDIRGFAVKFYTREGNFDLVGNNTPVFFIRDGIQFPDVVHALKPNPKTNIQEYWRILDYMS-HLPESLLTWCWMFDDV-GIPQDYRHMEGFGVHTYTLIAKSGKVLFVKFHWKPTCGIKNLTDEEAKVVGGANHSHATKDLHDAIASGNYPEWKLFIQTMDPADEDKFDFDPLDVTKIWPEDILPLQPVGRLVLNRTIDNFFNETEQLAFNPGLVVPG-IYYSDDKLLQCRIFAYGDTQRHRLGPNYLQLPVNAPKCA-HHNNHHEGFMNF-MHRDEEINYYPSKFDPVRCAEKVPTPTNSYTGIRTKCVIKK-ENNFKQAGDRYR-SWAPDRQDRFVKRWVEILSEPRTHEIRGIWISYWSQADRSLGQKLASRLNVRPSI-

>Arabidopsis.thaliana_CAT2

MDP--------YKYRPASYNSPFFTTNSGAPVWNNNSSMTVGPRGPILLEDYHLVEKLANFDRERIPERVVHARGASAKGFFEVTHDISNLTCADFLRAPGVQTPVIVRFSTVIHERGSPETLRDPRGFAVKFYTREGNFDLVGNNFPVFFIRDGMKFPDMVHALKPNPKSHIQENWRILDFFS-HHPESLNMFTFLFDDI-GIPQDYRHMDGSGVNTYMLINKAGKAHYVKFHWKPTCGVKSLLEEDAIRVGGTNHSHATQDLYDSIAAGNYPEWKLFIQIIDPADEDKFDFDPLDVTKTWPEDILPLQPVGRMVLNKNIDNFFAENEQLAFCPAIIVPG-IHYSDDKLLQTRVFSYADTQRHRLGPNYLQLPVNAPKCA-HHNNHHEGFMNF-MHRDEEVNYFPSRYDQVRHAEKYPTPPAVCSGKRERCIIEK-ENNFKEPGERYR-TFTPERQERFIQRWIDALSDPRTHEIRSIWISYWSQADKSLGQKLASRLNVRPSI-

>Arabidopsis.thaliana_CAT1

MDP--------YRVRPSSHDSPFFTTNSGAPVWNNNSSLTVGTRGPILLEDYHLLEKLANFDRERIPERVVHARGASAKGFFEVTHDITQLTSADFLRGPGVQTPVIVRFSTVIHERGSPETLRDPRGFAVKFYTREGNFDLVGNNFPVFFVRDGMKFPDMVHALKPNPKSHIQENWRILDFFS-HHPESLHMFSFLFDDL-GIPQDYRHMEGAGVNTYMLINKAGKAHYVKFHWKPTCGIKCLSDEEAIRVGGANHSHATKDLYDSIAAGNYPQWNLFVQVMDPAHEDKFDFDPLDVTKIWPEDILPLQPVGRLVLNKNIDNFFNENEQIAFCPALVVPG-IHYSDDKLLQTRIFSYADSQRHRLGPNYLQLPVNAPKCA-HHNNHHDGFMNF-MHRDEEVNYFPSRLDPVRHAEKYPTTPIVCSGNREKCFIGK-ENNFKQPGERYR-SWDSDRQERFVKRFVEALSEPRTHEIRSIWISYWSQADKSLGQKLATRLNVRPNF-

>Chondrus.crispus_2_CAT

MDT--------EKLQPRDFDTEHLTTNSGAPVDDSLNSLTLGERGPVLLSDFHLVEKLANFDREVIPERRVHARGIAAKGVFICTNDVSKYTMADPFTEVGKRTPMAVRFSTVVHSRGSPETLRDPRGFATKFYTGKGNWDLVGNLEPVFFIRDAMQFPEMVHAFKPSPKTEKQEWHRILDFLS-FHPECMHMMTWLLDDV-GIPKNYTTMNGAGVHTFVMINAAGKETYVKFHWISEQGEHNLLDDEAKQVAGEDFSHATSHLIDSIEMGNYPAWRLKIQVMDPATELNHSWDPLDPTKTWPEKDFPMIQVGRMVLNQNMDNQFLQTESLAFSPGNVIPG-ITFSQDKLLQGRIFSYADTQRYRLGANYLQIPINAPRCP-FMNRQFDGAGAT-MHRKEEVNYFPSTLHNAETVSHVPNVKRPVSGTQTRRALSK-QNNFGQAGERWR-SFDEPRRQRLVVRAAEKLNAPRPRHLKNTWIEYWTKCDRDLGARVAALVKMSSL--

>Dictyostelium.discoideum_CAT_B

MNK---KLEQLEKFKTNDKNPVYSTTNTGVSLSDDANSLKAGPRGPTLLEDFVLREKITHFDHERIPERIVHARGTGAHGYFLSYKDHSKLTKADFLSKQDKKTPVFIRISTVQGPRGSADTVRDVHGFAVKFYTDEGNYDLVGNNMPVFFIQDASSFPDFVHAVKMEPQNEMPTHDTFYDFCG-LKPESAHSVLWVMSDR-GIPISLRHQQGFGVHSYRFINQEGKSTFVKLHWKPLSGTCSLLWDEAQKIAGKDCDYHRRRFWEDIESGDFPQWELGAQLLDEDLQKKFDFDILDPTKLIPEELTPVIPLGRMVIDRNPDNFFAETEQVAFCVSHVVPG-IDFSNDPLLQGRIFSYLDTQLSRLGPNFNEIPINRPVCP-FANNQRDGIHRM-TINKGGASYFPNSIDKGYPLGGFRPYPENISGTKSYDRSETFEDHFSQATMFWN-SMSQHEKNHIIAAYTFEISKCSRPEVRTRYVNNLVNIDSVLAEKVAKNLGVKIEPT

>Podospora.anserina_CAT_B

SMS-AQQTENILEEYEVDDSNAYMSSDVGGPMED-QNSLKAGYRGSTLMEDWIFRQKIQHFDHERIPERAVHARGAGAHGTFTSYADWSNITAASFLGGAGKKTPVFVRFSTVAGSRGSADTARDVHGFATRFYTDEGNFDIVGNNIPVFFIQDAIRFPDLIHSVKPSPDNEVPQHDSAWDFFS-QQPSTMHTLFWAMSGN-GIPRSYRHMDGFGVHTFRFVTDDGNSKLIKWHFKTKQGKASLVWEEAQVLAGKNADFHRQDLWDAIESGNGPEWELSVQIVDEEKALAFGFDLLDPTKIIPEELAPLVPLGIMKLDRNPTNYFAETEQVMYQPGHIVRG-VDFTEDPLLQGRLFSYLDTQLNRNGPNFEQIPINRPVSP-VHNNNRDGAGQM-LIHKNVYPYTPNTLNGGYPLGFFTAPNRIVDGKLVRALSPTFDDHWSQPRLFYN-SLTRVEQQFLINAIRFEASHLKNEQVKKNVLEQINRVSNDVAKRVAVALGLEAPAP

>Podospora.anserina_CAT strain S / ATCC MYA-4624

SMS-AQQTENILEEYEVDDSNAYMSSDVGGPMED-QNSLKAGYRGSTLMEDWIFRQKIQHFDHERIPERAVHARGAGAHGTFTSYADWSNITAASFLGGAGKKTPVFVRFSTVAGSRGSADTARDVHGFATRFYTDEGNFDIVGNNIPVFFIQDAIRFPDLIHSVKPSPDNEVPQHDSAWDFFS-QQPSTMHTLFWAMSGN-GIPRSYRHMDGFGVHTFRFVTDDGNSKLIKWHFKTKQGKASLVWEEAQVLAGKNADFHRQDLWDAIESGNGPEWELSVQIVDEEKALAFGFDLLDPTKIIPEELAPLVPLGIMKLDRNPTNYFAETEQVMYQPGHIVRG-VDFTEDPLLQGRLFSYLDTQLNRNGPNFEQIPINRPVSP-VHNNNRDGAGQM-LIHKNVYPYTPNTLNGGYPLGFFTAPNRIVDGKLVRALSPTFDDHWSQPRLFYN-SLTRVEQQFLINAIRFEASHLKNEQVKKNVLEQINRVSNDVAKRVAVALGLEAPAP

>Phaeosphaeria.nodorum_2_CAT

LAA-PKSTDEFMAQYEVDDKDVYLTNGFGGPVED-MESLSAGERGPTLLEDFIFREKIMHF---------------GAHGVFTSYADWSNITGASFLNKPSKETPVFIRFSTVAGSRGSPDSVRDVRGFAVRFYTDEGNFDIVGNNIPVFFIQDAIKFPDLIHAVKPRQDNEIPQHDSAWDFFS-QQPSSMHTLFWAMSGH-GTVRSYRHMDGWGVHTFRLVTDEGKTKLVKFRFRTQQGLASRLWEEQQHAAGMNADTDRQDLWDAIENGYYPEWIFEAQIMDEEDQLRFGFDLLDPTKIVPEDLVPFTPLGKLTLNRNPRNYFAETEQV------MTPS-STATEDQTLN----SYQSTN---------------PAFQ-YHTNQRDGAGQM-YIPLNNAPYSPNTLNAGSPKGFFSAPNRSTGGRLVRAVSSTFADVWSQPRLFFN-SLLPVEQQMVINAMRFETAQLTSEVVKNNVLIQLNRVSHDIAVRVAEALDMTAPAA

>Podospora.anserina_CAT_A

MASGGDKVADLQKETKEMSDKARLTTDYGVKADDWLKIVNNDKTGPMLLEDPFARERIHRFDHERIPERVVHARGSGAFGKFKLFESAEDVTFAPILTDTSRETPIFIRFSTVLGSRGSADTVRDVRGFAIKFYTQEGNWDIVANNIPVFFIQDAIKFPDVIHAGKPEPHNEVPQHNNFWDFQY-NHTEATHMFMWAMSDR-GIPRSYRMMQGFGVNTFTLINAKGERHFVKFIFTPELGVHSLIWDEALKLAGQDPDFHRKDLWEAIENGVFPKWKFGIQVIPEADEHKFDFDILDATKIWPEDLVPVRYIGEFELNRNPDEFFPQTEQVAFCTSHIVPG-IGFSDDPLLQGRNFSYFDTQLSRLGINWQELPINRPVCP-VMNFNRDGAMRH-TITKGTVNYWPNRFEKVKPAGGYVEYAEKVAGIKARARSAKFKEHFAQAQLFWN-SMSAVEKNHIINALGFELDHCEDPVVYERMVTRLADIDLGLAQTVAEMVGGEPPKE

>Phaeosphaeria.nodorum_3_CAT

MAS---KVAQLQADTKDVDPKWKMTSDYGVKTDDWLKVATEDQQGPMLLEDHFAREKIQRFDHERIPERVVHARGSGAFGKFTLFESAADVTSAGVLTDTSRETPVFLRFSTVLGSRGSADTVRDVRGFAVKFYTEEGNWDIVGNNIPVFFIQDSMKFPDVIHAGKPEPDNEMPQHNNFWDFQY-MHPEATHMFFWAMSDR-TIPRSYRMMQGFGVNTFTLTNDKGERSFVKFIFTPELGVHSFVWDEALKIAGQDPDFHRKDLWTAIENGAYPKWKFGIQVIPESKEHDFEFDILDATKVWPEELVPIRYIGQLELNKNPDEFFTQTEQVAFCTGHVVPG-IGFSDDPLLQGRNFSYNDTQLSRLGVNWQELPINKPVCP-VMNFNRDGAMRH-GITKGKVNYWPNRFESAPPAGAYIDFPAKVAGIKARTQSKKFREHKNQAELFYN-SLSEPEKMHAMNAFAFELDHCDDPLVYNRICERLCEIDLELAQKVSEMVGGDIPTT

>Pyropia.yezoensis_CAT

MTAAAAAVVVAAATAAASHAEGPYTSEFGTLVGDDRNTLSAGPRGPQLVQDTRAFEKLARFNRERIPERVVHARGTGAHGVFESYGDQSALTRAGFLGGAGRKTEVFVRFSTVIHSKGSPETLRDPRGFAVKFKIAEGIWDLVGNNLDVFFIRDQVSFPDMVHSLKPDPVTNIQDPNRFFDFFGALGGAATNMLTTLYSDL-GTPATLREMNGHSVHAYKLISAERKVTYVKFQWTSMQGIRNFTAAEAMHMQARDFNHATRDLYDSIHSGKNPSWELRVQVMPSDRMYKLDFDPLDATKRWPEWIAPFKTIGRMTLNRVPDNFFQATESVAMSPGTFLPGAIEPSEDKLLQGRLVSYPDTQRYRLGANYADLPINRPVSP-VRSYAQDGAGNN-GAMKGTLNYGLSMTMPTFPTTHALFSESKVCDVVTQAPIPV-TADFAQAGELYN-SYNWRERANLIANLAGDLGQVRSMLVRNTMCSHFYKAHKQFGRRIARAVDCDMKVV

>Chondrus.crispus_1_CAT

LTS----AFLLVILSLSLAAREFLTGDTGHHVGSNQNSQTATKSGGILLQDIYALQKLRRFNTERIPERVVHGRGAGAHGEFRSFGNFSDLTAAEFLSKRGIETEVFVRFSTVIHGKHSPETVRDPRGFAVKFKTATGNYDLVGNNLPVFFIRDHLKFPDMVHSLKPDPVTNLQDPNRYFDFFSALGGMATHMLTYLYSDL-GIPRGYRFMDGHSVNAYKMVNAKKQVKYVKFRWLSKQGVQNLTRAEAAQVQGADFSHATRDLYDAIRRGDFPTWELGVQVMDPSQLDDFDFNPLDASKDWPARQFPFTALGELKLNRVPDNFHLASEQSAFSPGNFLPGKIEPSEDRLLQGRLVSYHESQMHRHGSNFQYLPVNRAKSA-VRNYNQDGVMVMEHAWKGSVNYEPSNDADAYVEDKSLYSTREICGKNEQGPIEK-TLNFRQAGELYR-AFSEQQRANLVGNLAVELRTLRSQKILHTMCAHLYKADEEYGRRVAEAAGCTLAKV

**Alignment for Fig 2B – CAT rooted tree including metazoan sequences and Choanoflagellata outgroup**

>Acanthaster.planci_4_CAT

MASRDKATNQMEEYKKTLDKMDRLTTSTGMPIDNKQATLTAGPRGPVLMQDFAFSDEMSHFGRERIPERVVHAKGAGAFGYFEVTHDISEYTKACVFESIGKKTPVAVRFSTVGGESGSADTARDPRGFAVKFYTEDGNWDLVGNNTPIFFIRDPIFFPSFIHTQKRNPVTHLKDPDMFWDFITLRPESTHQVSFLFSDRGTPDGYRHMNGYGSHTFKLVNKDGKGVYCKFHLKTDQGIRNLNAAQAEALASGDPDYAIRDLYNAISKEDFPSWSVKIQVMTFEQAEQHKDNPFDLTKVWPQAEYPLIPVGKMVLNLNPRNYFAEVEQIAFAPAHMIPGIEPSPDKMLQGRLFSYPDTHRHRLGTNYLQIPVNCPYKAKTRNYQRDGPQCVTDNQNGAPNYYPNSFNGPTDDLKYAQQTFSISGDVARYNTKDDDNFTQPGIFWSKVLTPKDQDALVSNMAGHLKNAQEFIQKRAVDNWSQCSAEWGKRLQAALDEHKAVA

>Amphimedon.queenslandica_6_CAT

MAASKGSTSQLVNFAAYKKSPDILTTSHGHPVDCKTAILTAGAKGPVLLQDYVFLDEMAHFDRERIPERVVHAKGAGAFGYFEVTHDITNYCKAKVFNKIGKRTPIAVRFSTVGGESGSADTVRDPRGFAVKFYTEDGNWDLVGNNTPIFFIRDPILFPSFIHTQKRNPSTHLK---------------------------------------------------------------------------------------------------------------------------------------------------------------------------------------------------------------------------------------------------------------------------------------------------------------------------------------

>Amphimedon.queenslandica_7_CAT

---------------------------------------------------------MAHFDRERIPERVVHAKGAGVFGYFKVSHDITNYCKAKVFNKVGKRAPIAVHFSTVGGESGSADTVRDPRGFAVKFYTEDGNWDLIGNNSPLFFIRDPILFPSFMHTQKRNPSTHLK---------------------------------------------------------------------------------------------------------------------------------------------------------------------------------------------------------------------------------------------------------------------------------------------------------------------------------------

>Xestospongia.bergquistia_43_CAT

MDTRDKAANQLTDFAKSKKSPDVLTTSHGHPVDNKTATLTAGPKGPVLIQDYIFLDEMAHFDRERIPERVVHAKGAGAFGYFEVTHDISKYCKAKVFSQIGKRCPIAVRFSTVGGESGSADTVRDPRGFAVKFYTEDGNWDLVGNNTPIFFIRDPILFPSFIHTQKRNPTTHLKDPDMFWDFISLRPETTHQVSFLFSDRGIPDGYRHMNGYGSHTFKLVNINGEPVYCKFHYKTDQGIKNLSVEKAGILAGDEPDYGIKDLYVAIATRNFPSWTLYIQVMTFEQAERYRWNPFDLTKIWPHKEFPLIPVGKLVLNRNPSNYFAEVEQVAFSPAHMVPGIEPSPDKMLQGRLFSYDDTHRHRLGVNYHQIPVNCPYATRTRNYQRDGFMTVDGNQGGAPNYFPNSFSGPLDNPSHAISKTITTGDVRKYNTKDDDNFSQVTNFWLHVLSTDEKTRLVSIIAGHLKNAADFIQKRAVRNFTEVHPEYGGRIAALLEKYKQIH

>Oscarella.carmela_28_CAT

MSATAKASEQLLNYSKHGKNAGVCTTGDGIPVDTTTATMTAGHRGPVLLQDYNFLDVMAHFDHERIPERVVHAKGAGAFGYFEVTHDITQYCKAKVFERVGKRTPLAVRFSTVGGESGSADTVRDPRGFAVKFYTDDGNWDLVGNNTPIFFIRDPILFPSFIHTQKRNPVTHLKDPDMFWDFISLRPETTHQVSFLFSDRGIPDGHRHMNGYGSHTFKMVNKDNKPVYVKFHYKTDQGIKNLPVGKAGELAGSDPDYSIRDLYNAIAEGNPPSWTLYIQVMTAEQAEKYKWNPFDVTKVWCHKDAPLIPVGRMVLDRNPVNYFAEVEQIAFSPAHMPPGIEASPDKMLQGRLFSYDDTHRHRVGVNFQQLPVNRPH-VKVVNYQRDGPAAIDGNQAGAPNYFPNSFQGPQIQPSVAHSSFGVKGDAVRIETHDDDNFSQAGTFWSDVLNDEERQRLVENIAGHLKDAKEFIQERVVANFSKAHPDYGKRIEELLKKYKA--

>Ephydatia.muelleri_24_CAT

AENRSNCANQLIDHAHSSADADPITTSHGCPVDSKTASLTVGERGLIPIQDFTFLDEMAHFDRERIPERVVHAKGAGAFGYFEVTHDITKYCKAKIFSHVGKKTPIAIRFSTVGGENGSADTVRDPRGFAIKFYTEEGNWDLVGNNTPIFFIRDPILFPSFIHTQKRNPATHLKDPDMFWDFITLRPETTHQVSFLFSDRGIPDGYRHMNGYGSHTFKLVNKAGEAVYCKFHFKTDQGIKNLSVQKAGELSMNDPDYSIRDLYESIATGNFPSWTLFLQVMTFEQAEKFRLNPFDLTKVWPHSEYPLIPVGKLVLNRNPKNYFAEVEQIAFCPAHLVPGVEPSPDKMLQGRLFSYSDTHRHRLGSNYHMIPVNCPYATKANTYHRDGSMCVDSNQGGAPNYYPNSFQGPVDSQIHALSKTTVTGDVKRYNTADHDNFSQVTNFWTKVLSEEEKTRLVENIAGHLKDATEFIQKRAVQNFTAVHRDYGGRLNLLLQQYKKKV

>Sycon.ciliatum_31_CAT

MAAKSKCPNQLQDYAKGKKNSDKLTTSAGCPVDSKTSTMTVGPRGPVLLQDQVFLDDMAHFDRERIPERVVHAKGAGAFGYFEVTDDITKYCKAKIFEQVGKRTPMAVRFSTVGGESGSADTVRDPRGFAVKFYTEEGNWDLVGNNTPIFFIRDPILFPSFIHTQKRNPQTHLKDADMFWDFISLRPETTHQVSFLFSDRGIPNGYRFMNGYGSHTFKLVNARNEPVYCKFHYKTDQGIQNLPVDQAGSLASSDPDYSIRDLFESIAQGNFPSWTMYVQVMTYEQAEKWKWNPFDLTKIWPHKEFPLIKAGRFVLNRNPKNYFAEVEQLAFSPAHLVPGIEPSPDKMLQGRLFSYTDTHRHRLGANYQQLPVNCPFATRPSNYQRDGPMAMGDNQGGAPNYYPNSFSGPRDDPKYAACPERVSGDVKKYNTADDDNFTQAGLFYRSVLNEAEKQRLVENIAGHMKDAKPFIQQRAVRNFAMADPDYGARISRLLAKYQSSS

>Oscarella.carmela_29_CAT

MSSRTKASEQLSDYASFRSTPENCTTGDGTPVDVKTATMTAGPRGPVLLQDYNFLDEMAHFDRERVPERIVHAKGAGAFGYFEVTHDITRFCKAKVFESVGKKTPLAIRFSTVGGESGSADTVRDPRGFAVKFYTEEGNWDLVGNNTPIFFIRDPILFPSFIHTQKRNPVTHLKDPDMFWDFISLRPETTHQVSFLFSDRGIPDGYRHMNGYGSHTFKMVNKKDEPVYAKFHYKTDQGIQCLPVDKAGDLSGSDPDYGIRDLYNAIAQGKFPSWTLYIQVMTFEQAEKHRWNPFDVTKVWPHSEFPLIQVGRITLNRNPVNYFAEVEQIGFSPAHMPPGIEPSPDKMLQGRLFSYDDTHRHRIGANYLQLPVNCSQNVKVRNYQRDGPMTIDNNQAGAPNYFPNSFQGPAEKRQAGPTKFHVSGDVARYNSADDDNFTQAGTFWTKVLNEEERQRLVENIAGHLKDAKDFIQARAVRNFSQAHPDYGRRIADLLAKYSQRL

>Danio.rerio_20_CAT

ADDREKSTDQMKLWKEGGQRPDVLTTGAGVPIGDKLNAMTAGPRGPLLVQDVVFTDEMAHFDRERIPERVVHAKGAGAFGYFEVTHDITRYSKAKVFEHIGKTTPIAVRFSTVAGEAGSSDTVRDPRGFAVKFYTDEGNWDLTGNNTPIFFIRDTLLFPSFIHSQKRNPQTHLKDPDMVWDFWSLRPESLHQVSFLFSDRGIPDGYRHMNGYGSHTFKLVNAQGQPVYCKFHYKTNQGIKNIPVEEADRLAATDPDYSIRDLYNAIANGNFPSWTFYIQVMTFEQAENWKWNPFDLTKVWSHKEFPLIPVGRFVLNRNPVNYFAEVEQLAFDPSNMPPGIEPSPDKMLQGRLFSYPDTHRHRLGANYLQLPVNCPYRTRVANYQRDGPMCMHDNQGGAPNYYPNSFSAPDVQPRFLESKCKVSPDVARYNSADDDNVTQVRTFFTQVLNEAERERLCQNMAGHLKGAQLFIQKRMVQNLMAVHSDYGNRVQALLDKHNAEG

>Danio.rerio_21_CAT

ADDREKSTDQMKLWKEGGQRPDVLTTGAGVPIGDKLNAMTAGPRGPLLVQDVVFTDEMAHFDRERIPERVVHAKGAGAFGYFEVTHDITRYSKAKVFEHIGKTTPIAVRFSTVAGEAGSSDTVRDPRGFAVKFYTDEGNWDLTGNNTPIFFIRDTLLFPSFIHSQKRNPQTHLKDPDMVWDFWSLRPESLHQVSFLFSDRGIPDGYRHMNGYGSHTFKLVNAQGQPVYCKFHYKTNQGIKNIPVEEADRLAATDPDYSIRDLYNAIANGNFPSWTFYIQVMTFEQAENWKWNPFDLTKVWSHKEFPLIPVGRFVLNRNPVNYFAEVEQLAFDPSNMPPGIEPSPDKMLQGRLFSYPDTHRHRLGANYLQLPVNCPYRTRVANYQRDGPMCMHDNQGGAPNYYPNSFSAPDVQPRFLESKCKVSPDVARYNSADDDNVTQVRTFFTQVLNEAERERLCQNMAGHLKGAQLFIQKRMVQNLMAVHSDYGNRVQALLDKHNAEG

>Danio.rerio_19_CAT

ADDREKSTDQMKLWKEGGQRPDVLTTGAGVPIGDKLNAMTAGPRGPLLVQDVVFTDEMAHFDRERIPERVVHAKGAGAFGYFEVTHDITRYSKAKVFEHIGKTTPIAVRFSTVAGEAGSSDTVRDPRGFAVKFYTDEGNWDLTGNNTPIFFIRDTLLFPSFIHSQKRNPQTHLKDPDMVWDFWSLRPESLHQVSFLFSDRGIPDGYRHMNGYGSHTFKLVNAQGQPVYCKFHYKTNQGIKNIPVEEADRLAATDPDYSIRDLYNAIANGNFPSWTFYIQVMTFEQAENWKWNPFDLTKVWSHKEFPLIPVGRFVLNRNPVNYFAEVEQLAFDPSNMPPGIEPSPDKMLQGRLFSYPDTHRHRLGANYLQLPVNCPYRTRVANYQRDGPMCMHDNQGGAPNYYPNSFSAPDVQPRFLESKCKVSPDVARYNSADDDNVTQVRTFFTQVLNEAERERLCQNMAGHLKGAQLFIQKRMVQNLMAVHSDYGNRVQALLDKHNAEG

>Homo.sapiens_1_CAT

-------------------------------------------------QDVVFTDEMAHFDRERIPERVVHAKGAGA--------------------------------------------------------------------------------------------------------------------------------------------------------------------------------------------------------------------------------------------------------------------------------------------------------------------------------------------------------------------------------------------------------------------------------------C

>Xenopus.tropicalis_39_CAT

ADKRDNAADQMKLWKESGQKPDVLTTGGGNPISDKLNLLTVGPRGPLLVQDVVFTDEMAHFDRERIPERVVHAKGAGAFGYFEITHDITKYSKAKVFEHIGKRTPIAIRFSTVAGESGSADTVRDPRGFAVKMYTEDGNWDLTGNNTPVFFIRDAMLFPSFIHSQKRNPQTHLKDPDMVWDFWALRPESLHQVSFLFSDRGIPDGHRHMNGYGSHTFKLVNAKDEAVYCKFHYKTDQGIRNLTVEEANRLSASDPDYGIHDLYESIAAGNYPSWSFYIQVMTFQQAEKFKFNPFDLTKIWPHGDYPLIPVGKLVLNRNPTNYFAEVEQLAFDPSNMPPGIEPSPDKMLQGRLFSYPDTHRHRLGPNYLQLPVNCPYRTRVANYQRDGPMCFTDNQGGAPNYYPNSFCAPENQPQVREHRFHVSADVARYNSADEDNVSQVRDFYVKVLSEEQRLRLCENIAGHLKDAQLFIQKRAVKNFTDVHPEYGARIQALLDKYNAEG

>Xenopus.tropicalis_42_CAT

AGNKEKAPTQMNQWKEAGQKPSVLTTGAGHPVGDKLNLLTAGPRGPLLVQDVVFTDEMAHFDRERIPERVVHAKGAGAFGYFEVTHDITQYCKANVFGKVGKRTPVAARFSTVAGEAGSPDTIRDPRGFAVKMYTEEGNWDLTGNNTPIFFIRDAILFPSFVHSQKRNPQTHMKDPDMVWDFWSLRPESLHQVSFLFSDRGIPDGHRHMNGYGSHTFKLVNCKDEAVYCKFHFKTDQGIRNLTLEKAEQLAASDPDYGIRDLYEAIAAGNYPSWTFYIQIMTFEQAEKFPFNPFDVTKVWPHGDYPLIPVGKMVLSRNPTNYFAEVEQLAFDPSNMPPGIEPSPDKMLQGRLFSYPDTHRHRLGANYLQLPVNCPYKARVANYQRDGPMCFTDNQGGAPNYYPNSFSAPEQQPQFREHRFRVSADVERYNSANDDNVTQVREFYLKVLNEEERQRLCENIVGHLKECQLFIQKRTVKNFSDVHPDYGSRIQALLDKHNAKC

>Homo.sapiens_2_CAT

ADSRDPASDQMQHWKEQAQKADVLTTGAGNPVGDKLNVITVGPRGPLLVQDVVFTDEMAHFDRERIPERVVHAKGAGAFGYFEVTHDITKYSKAKVFEHIGKKTPIAVRFSTVAGESGSADTVRDPRGFAVKFYTEDGNWDLVGNNTPIFFIRDPILFPSFIHSQKRNPQTHLKDPDMVWDFWSLRPESLHQVSFLFSDRGIPDGHRHMNGYGSHTFKLVNANGEAVYCKFHYKTDQGIKNLSVEDAARLSQEDPDYGIRDLFNAIATGKYPSWTFYIQVMTFNQAETFPFNPFDLTKVWPHKDYPLIPVGKLVLNRNPVNYFAEVEQIAFDPSNMPPGIEASPDKMLQGRLFAYPDTHRHRLGPNYLHIPVNCPYRARVANYQRDGPMCMQDNQGGAPNYYPNSFGAPEQQPSALEHSIQYSGEVRRFNTANDDNVTQVRAFYVNVLNEEQRKRLCENIAGHLKDAQIFIQKKAVKNFTEVHPDYGSHIQALLDKYNAEK

>Homo.sapiens_3_CAT

ADSRDPASDQMQHWKEQAQKADVLTTGAGNPVGDKLNVITVGPRGPLLVQDVVFTDEMAHFDRERIPERVVHAKGAGAFGYFEVTHDITKYSKAKVFEHIGKKTPIAVRFSTVAGESGSADTVRDPRGFAVKFYTEDGNWDLVGNNTPIFFIRDPILFPSFIHSQKRNPQTHLKDPDMVWDFWSLRPESLHQVSFLFSDRGIPDGHRHMNGYGSHTFKLVNANGEAVYCKFHYKTDQGIKNLSVEDAARLSQEDPDYGIRDLFNAIATGKYPSWTFYIQVMTFNQAETFPFNPFDLTKVWPHKDYPLIPVGKLVLNRNPVNYFAEVEQIAFDPSNMPPGIEASPDKMLQGRLFAYPDTHRHRLGPNYLHIPVNCPYRARVANYQRDGPMCMQDNQGGAPNYYPNSFGAPEQQPSALEHSIQYSGEVRRFNTANDDNVTQVRAFYVNVLNEEQRKRLCENIAGHLKDAQIFIQKKAVKNFTEVHPDYGSHIQALLDKYNAEK

>Xenopus.tropicalis_41_CAT

-----------------AQTQPILTTGAGIPAGDKLNVLTAGPRGPMLMQDVVFVDEMAHFDRERIPERVVHAKGAGAFGYFEVTHDITKYCRAKVFERVGKRTDVAVRFSTVAGEAGSADSVRDPRGFALKFYTDDGIWDLVGNNTPIFFIRDPMMFPSFIHSQKRNPQTHLKDPDTVWDFWSLRPETLHQVTFLFSDRGIPDGHRHMNGYGSHTFKLVNAEGKAVYCKFHYKTDQGIKNLSVEEADRLVVSDPDYGIRDLFQSIAKKNFPSWTMYLQVMTFEEAEKCPFNPFDLTKVWPHRDYPLIPVGKLVLNRNPENYFAEVEQIAFDPSNMPPGIEASPDKMLQGRLFSYPDTHRYRLGPNYLHLPVNCPRGVQVAHYQRDGPMCMFNNPSHMPNYYPNSFSSPRDDPKCKDSTFVAAGDVGRHDCSEEDNVSQVRMFYTQTLTEGERKRLCENLARHLSEAQIFIQERAVKNFTDVHPDYGARIKSLLDKYNGDG

>Xestospongia.bergquistia_44_CAT

MAKRPKAFSQLEEYGSSQKRQEPLTTSHGHPVDFKTGVQTFGPRGPMLMQDFVYMDEMAHFDRERIPERVVHAKGAGAFGYFEVTHDITKYCSAKIFSEIGKKTPLVVRFSTVGGESGSADTVRDPRGFAVKFYTEDGNWDLVGNDTPIFFIRDPFLFPSFIHTQKRNPTTHLKDPDMFWDFISLRPETTHQVSFLFSDRGIPDGYRHMNGYGSHTFKLVNKDGEAVYCKFHYKTDQGIKNIPVDEAGRLAGSNPDYSIQDLYEAIATGNFPSWTLYIQVMTFKEAETHRFNPFDLTKVWPQKEFPLIPVGKIVLDRNPANYFADVEQAAFCPAHMPPGIEASPDKMLQGRLFSYSDTHRHRLGTNYHQIPVNCPYATKHRSYLRDGFMCVDGNQDGAPNYYPNSFNGPVDEGKHDIPIMKPASDVARYNSADEDNFTQVGIFYREVLSEAERIRLTENIAGHLKNAKEFIQKRAVENFRKADPDYGERIAKLLEQYKTQE

>Trichoplax.adhaerens_CAT

KNSRDKAADQLKEFRE-NNDPTTLTTSHGAPAESITDSVTVGPRGPILLQDITLIDHMAHFDRERIPERVVHAKGAGAFGYFEVTHDITKYCKAKVFENVGKRTPMAVRFSTVGGESGSADTARDPRGFALKFYTEEGNWDLVGNNTPIFFIRDPILFPSFIHTQKRNPVTHLKDPDMFWDFISLRRETTHQVSFLFTDRGTPDGYRHMNGYGSHTFKLVNADNEAVYCKFHLKTDQGIRNLTGEKAQEISGLDPDYAMRDLYNAIASGNSPSWTMYLQIMTFEQAEKWRFNPFDVTKIWPHSEFPLIPVGKMVLNRNPKNYFAEVEQIAFAPANFVPGIEPSPDKMLQGRLFSYNDTHRHRLGTNYAQLPVNCPYATKVGNYQRDGPQTFNDNHSGVPNYYPNSFNGPIANVNATPHTVQVIGDVKRYNTADDDNFTQVTVFWRKVLNEQERTALVNNIVGHLCMAQSFLQERAIENFSKVDPEYGRRIREGLNRKEEGA

>Ephydatia.muelleri_25_CAT

ADTRPKCQRQLEEYYSRQKAPEILTTSHGHPIDDKKATMTVGARGGVALEDFVFLDEMAHFDRERIPERVVHAKGAGAFGYFEVTHDITKYTKAKVFSQIGKKTPLAVRFSTVGGENGSADTVRDPRGFAVKFYTEDGNWDLVGNNTPIFFIRDPIFFPSFIHTQKRNPATHLKDPDMFWDFISLRPETTHQVSFLFSDRGIPDGYRHMNGYGSHTFKLVNSDGTPVYCKFHYKTDQGIKNLPVDQANKLAGDNPDYAIEDLYESIANGKYPSWTMYIQVMTFEQAEKVAFNPFDLTKVWSHADFPLIPVGKLVLDRNPRNYFAEVEQLAFSPAHMPPGIEASPDKMLQGRLFSYSDTHRHRLGPNSHLIPVNSAQCCRPRNYQRDSFMCTDGNQSDAPNYFPNSFNGPKDNPAVACSKHSFSGDVQRHRSDDEDNFTQAGIFYRKVLTDDQRTRLVEKHSWSCEVC------------CSIHP-------------GACG

>Branchiostoma.floridae_9_CAT

MAGRDKAGNQLEEYKKQNGNASTVTTGTGAPVDNKLAVLTVGPRGPMLMQDFTYMDEMAHFNRERIPERVVHAKGHGAFGYFECTHDISQYCKAKPFEHVGKRTPLGIRFSTVGGESGSADTARDPRGFAVKMYTEDGNWDLVGNNTPIFFIRDPILFPSFIHTQKRNPATHLKDPDMFWDFISLRPETCHQVSFLFSDRGTPNGYRHMNGYGSHTFKMVNNNNEAVYCKFHWKTDQGIKNLTRQQADDLAGSDPDYAGRDLFNAIAEGNYVST-----------------------------------------------------------------------------------------------------------------------------------------------------------------------------------------------------------------------------------

>Drosophila.melanogaster_22_CAT

MAGRDAASNQLIDYKNQTVSPGAITTGNGAPIGIKDASQTVGPRGPILLQDVNFLDEMSHFDRERIPERVVHAKGAGAFGYFEVTHDITQYCAAKIFDKVKKRTPLAVRFSTVGGESGSADTARDPRGFAVKFYTEDGVWDLVGNNTPVFFIRDPILFPSFIHTQKRNPQTHLKDPDMFWDFLTLRPESAHQVCILFSDRGTPDGYCHMNGYGSHTFKLINAKGEPIYAKFHFKTDQGIKNLDVKTADQLASTDPDYSIRDLYNRIKTCKFPSWTMYIQVMTYEQAKKFKYNPFDVTKVWSQKEYPLIPVGKMVLDRNPKNYFAEVEQIAFSPAHLVPGVEPSPDKMLHGRLFSYSDTHRHRLGPNYLQIPVNCPYKVKIENFQRDGAMNVTDNQDGAPNYFPNSFNGPQECPRRASSCCPVTGDVYRYSSGDEDNFGQVTDFWVHVLDKCAKKRLVQNIAGHLSNASQFLQERAVKNFTQVHADFGRMLTEELNLAKSSK

>Lingula.anatina_26_CAT

------------------------------------------------MQDFVFMDEMAHFQRERIPERVVHAKGAGAFGFFEVTHDISKYCKAKVFEHIGKRTPVAVRFSTVGGESGSADTARDPRGFAVKMYTDEGNWDCVGNNTPIFFIRDPIFFPSFIHTQKRNPVTHCKDPDMFWDFITLRPESTHQVSFLFSDRGTPDGYRQMNGYGSHTFKLVNKDGEAVYCKFHYKTDQGIKCLMADQAGELAGSDPDYAIRDLYNNIAAGNYPSWTWYIQVMTFEEAEKFRWNPFDLTKVWPQGEFPLIPVGRMVLNRNPKNYFAEVEQIAFSPAHLVPGIEASPDKMLQGRLFSYSDTHRHRLGTNYLQIPVNCPFNTRVKNYQRDGPQCVTDNQEGAPNYFPNSFNGPLDDKRHLESVFQTTGDVKRYNTRDDDNFSQVGLFWRNVLKPDERTRLVENIANHLKDAQEFIQQRAVKNFGQCDPEYGRRIQELLDQFKAKK

>Amphimedon.queenslandica_8_CAT

SSKRPACFSQLEEYAKKTQPGEVLTTTHGNPIDFKTAIQTFGPRGPMLMQDGVYLDEMAHFDRERIPERVVHAKGAGAYGVFEVTHDITKYCCAKLFSEVGKKTDLFIRFSTVGGESGSADTARDPRGFAVKFYTEDGNWDLVGNNTPIFFIRDPFLFPSFIHTQKRNPVTHLKDPDMFWDFISLRPETTHQVSFLFSDRGIPDGYRHMNGYGSHTFKLVNSKGEPVYCKFHYKTDQGIKNMPVGKAAELAGTNPDYSIQDLYEAIATGNFPSWTLSIQVMTYEQAEKCSFNPFDLTKVWPHADYPLIPVGKITLNRNPSNYFFDVEQSAFSPAHMPPGIEASPDKMLQGRLFSYDDTHFHRLGPNFQMIPVNCPYAGKPRNYVRDGPMCVDGNQGGAPNYYPNSFNGPKDMGKHDVTIFPPAGDVKRYNAADDDNFSQVGIFYNKVLNEEERTRLAQNIAGHMKNASPKIQERAIANFSKADPDYGARIKKYISQ-----

>Nematostella.vectensis_27_CAT

-----------------------------------------------------------------------------------------------------------------------------------------------------------------------------QDANMFWDFITLRPETTHQTSFLFSDRGIPDGFRHMNGYGSHTFKMVNSKGKAVYCKFHVKTDQGIKNCPVERATELAGTDPDYSTRDLYNAIAEGNY--------------------------------------------------------------------------------------------------------------------------------------------------------------------------------------------------------------------------------------

>Capitella.teleta_17_CAT

MANRDKASEQLNEYKQSNGTPGVLTTATGAPIGNKTAIQTVGPRGPALLQDFVFQDEMSHFGRERIPERVVHAKGAGAFGFFEVTHDITKYSKAKVFEHIGKKTPIVARLSTVGGEKGSADTARDPRGFAVKFYTDEGNWDLVGNNTPIFFIRDPMLFPSFIHTQKRNPKTNLKDPDAFWDFLTLRPESCHQVSFLFSNRGTPDGYRNMNGYGSHTFKLVNKEGVAHYCKFHYKTNQGIKNLTGAQADALAGSDPDYATRDLYNAIAEGNFPSYTLFIQVMTFEEAEKHRFNPFDLTKVWSHKEFPLIPVGRLTFNRNPKNYFAEVEQVAFSPAHMVPGIEASPDKMLQGRLYSYSDTHRHRLGTNYQQIPVNCPFSTRARNYQRDGPQNVDDNQEGAPNYFPNSFGGPQDSPAFLEHETTFPGDVARYNTKDDDNFTQVGIFWRETLTEEDRKHLIINMSGHLKNAQEFLQKRAVANFSKCDPEYGRRLQEALDQHKKDA

>Strongylocentrotus.purpuratus_30_CAT

--------------------------------------MTAGARGPVLIQDFVFTDEMSHFGRERIPERVVHAKGAGAFGYFETTHDISKYCKAAPFESVGKKTPVAIRFSTVGGESGSADTARDPRGFAVKFYSEDGNWDLVGNNTPIFFIRDPMFFPSFIHTQKRNPVTHLKDPDMFWDFITLRPEATHQVSFLFSDRGTPDGYRHMNGYGSHTFKLRNKDGEYVFCKFHFKCDQGIKNLNRHRAGDLSATDPDYAIRDLYNSIATGNFPSWSLHIQVMTQEQADKHRDNPFDLTK---------------------------------------------------GRLFSYSDTHRHRLGTNYLQIPVNCPFAARTRSYQRDGPQCVTDNQGGAPNYFPNSFTGPTDSKSYEQTKFTCPGEAARYETGDDDNYTQAGIFWRDVLSEADREATVDNMASHIKDAAEYLQKRTVVQWGKCDEDWGKRLEAKLAEYKTQA

>Tethya.wilhelma_37_CAT

DAKRPKHATQLEEYAKEQKTRPVMTTGHGIPIDSKTASMTVGPRGPITLQDVFYLDEMSHFDRERIPERVVHAKGAGAFGYFEVTQDITKYCKADLFSEVGKKTPIGIRFSTVGGESGSADTVRDPRGFAVKFYTQEGNWDLVGNNTPIFFIRDPLHFPNFIHTQKRNPVTHLKDHDMFWDFLTLRQESAHQVSFLFSDRGIPDGYRHMNGYGSHTFKLVNKDGEPVYCKFHYKTNQGIKNILPEEAEKMAGRDPDYAIRDLYDAIANKDFPSWTLSIQVMTFEQAEKTSFNPFDLTKIWPHKDYPLIEVGKMVLDRNPTNYFCEIEQIAFSPSNMVPGVEPSPDKMLQGRLFSYPDTHLYRLGPNYHQLPVNCPYMTKCRNYQRDGNMPLEGNQAGAPNYFPNSFQGPVDDRKYRWSKTTQTCDIDRYETADEDNYSQVGNFYRNVLSEVEKDRLTSNIAGHVKGAAKFIQERVVEMFTKCDPDYGQRIAKKLQG-----

>Caenorhabditis.elegans_15_CAT

-MPNDPSDNQLKTYKETYPKPQVITTSNGAPIYSKTAVLTAGRRGPMLMQDVVYMDEMAHFDRERIPERVVHAKGAGAHGYFEVTHDITKYCKADMFNKVGKQTPLLVRFSTVAGESGSADTVRDPRGFSLKFYTEEGNWDLVGNNTPIFFIRDAIHFPNFIHALKRNPQTHMRDPNALFDFWMNRPESIHQVMFLYSDRGIPDGFRFMNGYGAHTFKMVNKEGNPIYCKFHFKPAQGSKNLDPTDAGKLASSDPDYAIRDLFNAIESRNFPEWKMFIQVMTFEQAEKWEFNPFDVTKVWPHGDYPLIEVGKMVLNRNVKNYFAEVEQAAFCPAHIVPGIEFSPDKMLQGRIFSYTDTHYHRLGPNYIQLPVNCPYRSRAHTTQRDGAMAY-ESQGDAPNYFPNSFRGYRTRDDVKESTFQTTGDVDRYETGDDHNYEQPRQFWEKVLKEEERDRLVGNLASDLGGCLEEIQNGMVKEFTKVHPDFGNALRHQLCQKKH--

>Caenorhabditis.elegans_16_CAT

-MPNDPSDNQLKTYKETYPKPQVITTSNGAPIYSKTAVLTAGRRGPMLMQDVVYMDEMAHFDRERIPERVVHAKGAGAHGYFEVTHDITKYCKADMFNKVGKQTPLLVRFSTVAGESGSADTVRDPRGFSLKFYTEEGNWDLVGNNTPIFFIRDAIHFPNFIHALKRNPQTHMRDPNALFDFWMNRPESIHQVMFLYSDRGIPDGFRFMNGYGAHTFKMVNKEGNPIYCKFHFKPAQGSKNLDPTDAGKLASSDPDYAIRDLFNAIESRNFPEWKMFIQVMTFEQAEKWEFNPFDVTKVWPHGDYPLIEVGKMVLNRNVKNYFAEVEQAAFCPAHIVPGIEFSPDKMLQGRIFSYTDTHYHRLGPNYIQLPVNCPYRSRAHTTQRDGAMAY-ESQGDAPNYFPNSFRGYRTRDDVKESTFQTTGDVDRYETGDDHNYEQPRQFWEKVLKEEERDRLVGNLASDLGGCLEEIQNGMVKEFTKVHPDFGNALRHQLCQKKH--

>Caenorhabditis.elegans_11_CAT

TKPGPMAEDQLKAYRDRNQEPHLLTTSNGAPIYSKTAVLTAGRRGPMLMQDIVYMDEMAHFDRERIPERVVHAKGGGAHGYFEVTHDITKYCKADMFNKVGKQTPLLVRFSTVAGESGSADTVRDPRGFSLKFYTEEGNWDLVGNNTPIFFIRDAIHFPNFIHALKRNPQTHMRDPNALFDFWMNRPESIHQVMFLYSDRGIPDGFRFMNGYGAHTFKMVNKEGNPIYCKFHFKPAQGSKNLDPTDAGKLASSDPDYAIRDLFNAIESRNFPEWKMFIQVMTFEQAEKWEFNPFDVTKVWPHGDYPLIEVGKMVLNRNVKNYFAEVEQAAFCPAHIVPGIEFSPDKMLQGRIFSYTDTHYHRLGPNYIQLPVNCPYRSRAHTTQRDGAMAY-ESQGDAPNYFPNSFRGYRTRDDVKESTFQTTGDVDRYETGDDHNYEQPRQFWEKVLKEEERDRLVGNLASDLGGCLEEIQNGMVKEFTKVHPDFGNALRHQLCQKKH--

>Caenorhabditis.elegans_12_CAT

-----MAEDQLKAYRDRNQEPHLLTTSNGAPIYSKTAVLTAGRRGPMLMQDIVYMDEMAHFDRERIPERVVHAKGGGAHGYFEVTHDITKYCKADMFNKVGKQTPLLVRFSTVAGESGSADTVRDPRGFSLKFYTEEGNWDLVGNNTPIFFIRDAIHFPNFIHALKRNPQTHMRDPNALFDFWMNRPESIHQVMFLYSDRGIPDGFRFMNGYGAHTFKMVNKEGNPIYCKFHFKPAQGSKNLDPTDAGKLASSDPDYAIRDLFNAIESRNFPEWKMFIQVMTFEQAEKWEFNPFDVTKVWPHGDYPLIEVGKMVLNRNVKNYFAEVEQAAFCPAHIVPGIEFSPDKMLQGRIFSYTDTHYHRLGPNYIQLPVNCPYRSRAHTTQRDGAMAY-ESQGDAPNYFPNSFRGYRTRDDVKESTFQTTGDVDRYETGDDHNYEQPRQFWEKVLKEEERDRLVGNLASDLGGCLEEIQNGMVKEFTKVHPDFGNALRHQLCQKKH--

>Caenorhabditis.elegans_13_CAT

-MPNDPSDNQLKTYKETYPKPQVITTSNGAPIYSKTAVLTAGRRGPMLMQDVVYMDEMAHFDRERIPERVVHAKGAGAHGYFEVTHDISKYCKADIFNKVGKQTPLLIRFSTVGGESGSADTARDPRGFAIKFYTEEGNWDLVGNNTPIFFIRDPIHFPNFIHTQKRNPQTHLKDPNMIFDFWLHRPEALHQVMFLFSDRGLPDGYRHMNGYGSHTFKMVNKDGKAIYVKFHFKPTQGVKNLTVEKAGQLASSDPDYSIRDLFNAIEKGDFPVWKMFIQVMTFEQAEKWEFNPFDVTKVWPHGDYPLIEVGKMVLNRNPRNYFAEVEQSAFCPAHIVPGIEFSPDKMLQGRIFSYTDTHFHRLGPNYIQLPVNCPYRSRAHNTQRDGAMAY-DNQQHAPNFFPNSFNYGKTRPDVKDTTFPATGDVDRYESGDDNNYDQPRQFWEKVLDTGARERMCQNFAGPLGECHDFIIKGMIDHFSKVHPDFGARVKALIQKQARSH

>Caenorhabditis.elegans_14_CAT

-MPNDPSDNQLKTYKETYPKPQVITTSNGAPIYSKTAVLTAGRRGPMLMQDVVYMDEMAHFDRERIPERVVHAKGAGAHGYFEVTHDISKYCKADIFNKVGKQTPLLIRFSTVGGESGSADTARDPRGFAIKFYTEEGNWDLVGNNTPIFFIRDPIHFPNFIHTQKRNPQTHLKDPNMIFDFWLHRPEALHQVMFLFSDRGLPDGYRHMNGYGSHTFKMVNKDGKAIYVKFHFKPTQGVKNLTVEKAGQLASSDPDYSIRDLFNAIEKGDFPVWKMFIQVMTFEQAEKWEFNPFDVTKVWPHGDYPLIEVGKMVLNRNPRNYFAEVEQSAFCPAHIVPGIEFSPDKMLQGRIFSYTDTHFHRLGPNYIQLPVNCPYRSRAHNTQRDGAMAY-DNQQHAPNFFPNSFNYGKTRPDVKDTTFPATGDVDRYESGDDNNYDQPRQFWEKVLDTGARERMCQNFAGPLGECHDFIIKGMIDHFSKVHPDFGARVKALIQKQARSH

>Branchiostoma.floridae_10_CAT

-------------------------------------------------------------------------------------------------------------------------------------------------------------------------------------------------------------------------------------------------------------------------------PSWTLKIQVMTFEEAEKFRFNPFDLTKVWPQGEFPLIPVGKMVLNRNPKNYFAEVEQIAFSPIHMVPGIEASPDKMLQGRLFSYSDTHRHRLGSNYLQIPVNCPYRARVTNYQRDGPQCVDDNQAGAPNCYPNSFSGPKQKETITPPAIKTTGDLQRYNTADEDNFTQVGTFWRNVLSEYDREHLVDNLASHMTAAQEFLQKRAVKNFSQCDPEYGRRLQEKLDKYNAAK

>Amphimedon.queenslandica_5_CAT

---------------------------------------------------------------------------------------------------------------------------------------------------------------------------------MFWDFISLRPETTHQVSFLFSDRGIPDGYRHMNGYGSHTFKLVNNEGEPVYCKFHYKTDQGIANLSVEKAGILAGSDPDYAIKDLYDAIAAKNYPSWTLYIQVMTFEQAKEFEWNPFDLTKIWPQKEFPLIPVGRMVLNRNPANYFAEVEQLAFSPAHMVAGIEPSPDKMLQGRLFSYDDTHRHRLGPNYHQIPVNCPYATRTRNYQRDGPMTVDGNQGGAPNYFPNSFSGPVDNPEYTISPITSTCDVKKYNTRDDDNFSQVKNFWLKVLTVEEQSRLVFNIASHLKDAQPFIQSRVIRNFSSVHPDYGSRISDLLQQFKKKK

>Tethya.wilhelma_38_CAT

------------------------------------------------------------------------------------------------------------------------------------------------------------------------------------------------------------------------------------------------------------------------------------------------------------------------MILNRNPTNYFCEVEQIAFCPAHLVPGIEPSPDKMLQGRLFSYSDTHRHRLGANYHQIPVNCPYATRCKNYQRDGPQ------------------------------------------------------------------------------QRWIIKQLVEW-----------------------

>Ciona.intestinalis_18_CAT

-MGRTKSDNQLKEYAEKNKDKTVLMTGTGAPIEDKLNVLTVGERGPLLMQDFTFTDEMAHFNRERIPERVVHAKGGGAKGYLEITHDISNFCKADIFSSIGKRTPLAVRFSTVGGESGSADSARDPRGFAIKFYTEEGIWDLVGNNTPIFFIRDPIFFPSFIHTQKRNPQTHLKDPDMFWDFISLRPETTHQVSHILVMRNTPQGVIHFNT----TDNWCNISITLYQCNSRFKTDQGIKNLTADEADTLAATDPDHAIRDLYNAIADGNNPSWTMYIQVMTYQQATTHKWNPFDLTKTWPQGEFPLIQVGKMVLNENPSNYFAEVEQIAFSPSHMVPGIEASPDKMLQGRLFSYPDTHRHRLGSNYLQIPINCPFNVRVNNYQRDGPQCVTDNSKGAPNYYPNSFNGPLDGASRKQTSLHPASDVKKYNSADDDNFTQVGTFWRKVLNEAERKRLAENIGNHMKAAQPFLQKRAIANFAAADPEFGAMIQAVIDKAAAKI

>Sycon.ciliatum_32_CAT

MAGRCPAANQLLNYREEQKESTTTSTSWGAPVDIATASQTVGPRGPLLLQDANFIDNLAHFDRERIPERVVHAKGAGAFGELVITEDISKYTKALALQK-GTSTPVAVRFSTVGGEAGSADTVRDPRGFAVKHYTDDGVWDLVGNNTPIFFIRDPILFPNFIHTQKRNPRTHLKDPDMFWDFISLRPETTHQVSFLFSDRGIPDGYRHMNGYGSHTFKLVNSEGVAHYCKFHYKTSES-KNLLPNKAGPISGDDPDYAIRDLYNAIERKEFPTWKFQIQVMTFEQAETYKYNPFDLTKVWSHKDFPLIDVGTMTLNRNPEDYFSEVEQIAFSPSHMVPGIEPSPDKMLQGRLFSYTDTHRHRLGANYQHLPVNASKKTPQANSHRDGPMCM-FNQAGAPNYFPNSFKGPVDRPDARHSKISYSGDVEKYNSADEDNFSQVGVFYREVLNAEERQRLVENIAGHLKDALPEIQKRTVANFSQADADYGAGIQKLLDGYNK--

>Drosophila.melanogaster_23_CAT

MCSRDTASNQLIDYKNDSEVQREITTSSGTPVGVKDAIQTVGPRGPALLQDFQFLDEVMHFDSERIPERVAYAKGAGAFGYFECTHDISKFCAASIFDKVRKRTAVAMRFSVACGEQGSADTVREQRGFAVKFYTDDGIWDIVGCNMPVHYVRDPMLFPSLVHAQKRNPQTHLKDPDMFWDFMTLRPETLHALLMYFSDRGTPDGYRHLHGYGVHTYRMINASGETQYVRFHFKTDQGIKNLDARRCEELMSHDPDYAIRDLYNSIKKGNYPSWSMYIQVMLNEEAKKCRFNPFDVTKVWPQKDFPLLPVGKIVLDRNPTNYFTEVEQLAFSPAHMVPGIEPSPDKMLQGRLFAYGDSQRHRLGVNYMQIPVNCPYRVNVRNFQRDGAMTVTDNQNGAPNYFPNSFCGPRESPRLGQTCCPLSGDVYRFMSGDEDNFSQVTDFWTYTLDNCGRKRLVRNLSEHLTEASQFLQERAVKLFTMVHSDFGRLMTEALNTARISK

>Xenopus.tropicalis_40_CAT

MAGR---------YQTHEPELSLLTTASGVPIGDKRNSLTVGPRGPLLIQDAAFMEEMAHFNRERIPERVVHARGAGAFGYFEVTNDITQYCKAKVFSHVGKKTPIAVRFSTTTGELGSNDTVREPHGFAIKFYTEEGNWDLVGNHTPAFFIKDPILFPSLAHAQKKNPQTHLKDPNMFWDFVSLCPETLHEITHLFTDRGLPDGYRHMHGFGNHAFKLVNADGKPVYCKFHYKTNQGIKNLSSEQAKVIAGSDPDHALRDLLEAIAKGDYPSWTFSIQIMTFEQAEKMPFNPFDVTKVWYQKEFPLIPVGKLVLNRNPTNYFADVEQIALEPKNLVPGIEPSPDRVLQGRLFAYSDALRYRLGVNYTQIPVNRPQGVKVANYERDGHMVI-DNQGNAPSYYPNSFGGPKDKAEYKEMVFHVSGDVDRYHNAETDDNFQVRKFYQKVLNDKQKQELCQNIASSLTGALQFIQDKSVKNFAAIDPDYGARVQKELDKLRAAS

>Tethya.wilhelma_33_CAT

MADRTKCAKQLTDFSNRKRVPDVLTTSHGHPIDSKTATLTVGEKGPVLLQDFTFLDEMAHFDRERIPERVVHAKGAGAFGYFEVTHDITKYCKAKVFSHVGKRTPIAIRFSTVD---------------------------------------------------------------------------------------------------------------------------------------------------------------------------------------------------------------------------------------------------------------------------------------------------------------------------------------------------------------------------------------------------

>Tethya.wilhelma_34_CAT

MADRTKCAKQLTDFSNRKRVPDVLTTSHGHPIDSKTATLTVGEKGPVLLQDFTFLDEMAHFDRERIPERVVHAKGAGAFGYFEVTHDITKYCKAKVFSHVGKRTPIAIRFSTVG------------------------------------------------------------------------------------------------------------------------------------------------------------------------------------------------------------------------------------------------------HCISSN---------------------------------------------------------------------------------------------------------------------------------------

>Tethya.wilhelma_35_CAT

-----------------------------------------------------------------------------------------------------------------------------------------------------------------------------MDVDMFWDFISLRPETTHQVSFLFSDRGIPDGYRHMNGYGSHTFKLVNKDDEAFYCKFHYKTDQGIKCLDVDKAAKLSGTDPDYGIKDLFEAIATGNFPSWTLYIQVMTFEQAENFKWNPFDLTK---------------------------------------------------------------------------------------------------------------------------TTCDVKRYNTADMDNFSQVTTFWRKVLNEQEKSRLVRNIAGHLKDAAEFIQQRAVRNFMQVDPEYGGRIAKLLQEYKKQ-

>Tethya.wilhelma_36_CAT

-----------------------------------------------------------------------------------------------------------------------------------------------------------------------------MDVDMFWDFISLRPETTHQVSFLFSDRGIPDGYRHMNGYGSHTFKLVNKDDEAFYCKFHYKTDQGIKCLDVDKAAKLSGTDPDYGIKDLFEAIATGNFPSWTLYIQVMTFEQAENFKWNPFDLTK---------------------------------------------------------------------------------------------------------------------------TTCDVKRYNTADMDNFSQVTTFWRKVLNEQEKSRLVRNIAGHLKDAAEFIQQRAVRNFMQVDPEYGGRIAKLLQEYKKQG

>Monosiga.brevicollis_CAT

------------------SKCPYTTDAAGHPLDDQNAALRAGSTGPLVV-DPRLYEHNAIFNRERIPERVVHANGAGAFGTFRVTKDVSQYTKASFLSSVGKETKIAARFSTVAGERGFADADRDVRGFGVRFYTDEGNFDLVGNNTPIFFVRDPNKFQDFIHSQKRNPQTDLRDWEARWDFWSLSPESVHQVLILMSDRGVPRTYRHEHGYGSHTFSLINDKDERVWCKWHMRTNQGYETLAEEEAQKVRGANPDSHKDDLFHAIERGEYPSWKVCLQIMTDEQAKKMDFDIFDLTKVWSHKEFPLIEVGEMTLNRNPQNYFAEVEQLALSPGNLVPGIGASPDPMLQIRLIAYTDAANYRLGVNHYQLPVNAPVCPVSHKKYRDGLMNPSANSGGHPNYQPNSMDKYPTFGSWEPPLPLDQAVVQRKRQGQDDVYFQPRALF-QMFEKDHKERVYKNVAGTMKGIKQEIIERQLEVFGKVDAELEQGIRVALEQEA---

**Alignment for Fig 4 – PRX unrooted tree including non-metazoan sequences**

>Dictyostelium.discoideum_PRX4

QQIRIRKPAPAFKGQAVVNGEFKEISLDDY-KGKYLYLFFYPLDFTFVCP-TEIIAFSNAAEEFKKAGCELVG-CSIDSPFTHLAWINTPRKEGGL-GGINIPLLSDLTHQISKDYGVYIEED------GHTIRGSILIDKEGLVRVITMNDNPVGRSVDEAIRTLKALKFTDQFGEVCPANWSEGDKSMK-ADPKGS------------------KEYFEAVNK-

>Trichoplax.adhaerens_4

SKAQVQKAAPNFAAKAVVDGKFKDIKLSDY-LGKYLVLFFYPMDFTFVCP-TEIIAFSERVEDFRSRNCEVIA-CSTDTEFSHLAWINQPRKEGGL-GSMNIPILADPTHTLAKDYGVLLEDQ------GIALRGLFIIDGKGILRQITVNDLPVGRSVDETIRLVEAFQFTDEYGEVCPANWKPGKSTIK-PNPNDS------------------KAFFASQ---

>PRX1/2_S.ciliatum_2

SRAQIQQAAPVFKGQAVVDGQFKEISLSDY-AGKYVVFFFYPLDFTFVCP-TEIVAFSDRVEEFRKINCEVVA-CSVDSHFSHLAWTKVPRKQGGL-GDMNIPLLADLTKGISRDYGVLLEDQ------GIALRGLFIIDNKGVLRQSTVNDLPVGRSVDETLRLVKAFQFTDEHGEVCPANWTPGKNTIK-PSVDAS------------------QEFFSKAE--

>Trichoplax.adhaerens_5

SKVKISKPAPHFEGTAVINGAFKDIKLSDY-KGKYLVFFFYPMDFTFVCP-TEIIAFSDRVSEFHAINAEVVA-CSTDSKFTHLAWVKTTRKQGGL-GSMKIPLLSDITHQIARDYGVYLEKE------GHALRGLFIIDDKGILRQITMNDLPVGRSVDETLRLVQAFQYTDKHGEVCPANWKPGGATIV-PDPEKK------------------LDYFSEQNKD

>PRX4_O.carmela_1

SKTQISKPAPYFEATAVVNGEFKEIKLSDY-KGKYLVFFFYPLDFTFVCP-TEIIAFSDRAEEFRAINAEVVA-CSVDSPFTHLAWINTPRKEGGL-GKVDIPILSDMTHQISKDYGVLLENQ------GHTLRGLFIIDDKGTLRQITMNDLPVGRSVDETLRLVQAFQYTDNHAEVCPAGWKPGSDTII-PDPNKK------------------LTYFGQDP--

>PRX4_N.vectensis_2

SKAQISKPAPFWEGTAVVNGEFKELKLSDF-EGKYLVFFFYPLDFTFVCP-TEIIAFSDRIEEFRAINTEVVG-CSVDSVFTHLAWINSPRKEGGL-GNLKYPLLSDINHQVSKDYGVLLENE------GHTLRGLFIIDDKGVLRQITMNDLPVGRSVDETLRLVQAFQYTDKHGEVCPAGWKPGKDTII-PDPTQK------------------KKYFEQAQ--

>PRX4_A.planci_4

SKVQISKPAPTFEGTAVVNGEFKELKLTDY-KGKYLVLVFYPLDFTFVCP-TEIIAFSDRISEFKEINAEVVA-VSVDSQFTHLAWINTPRKQGGL-GPIQIPLLSDLTHQISKDYGVLLEDL------GHTLRGLFIIDGNGVLRQITMNDLPVGRSVDETLRLVQAFQYTDKNAEVCPAGWKPGSDTII-PNPEDK------------------LKYFSLPE--

>PRX4_S.purpuratus_1

SKVQISKPAPVFEGTAVVEGEFKAMKLSDF-AGKYLVLVFYPLDFTFVCP-TEIIAFSDRVDEFRAINTEVVA-ISVDSQFTHLAWINTPRTQGGL-GPIKLPILSDLTHQIAKDYGVLLEDL------GHTLRGLFIIDDKGVLRQITMNDLPVGRSVDETLRLVQAFQYTDKHGEVCPAGWKPGSDTII-PNPADK------------------LKYFAQPN--

>PRX4_L.anatina_2

SKAQISKAAPHFEGTAVVDGEFKELKLTDF-KGKYLVFFFYPLDFTFVCP-TEIIAFSDRVEEFRAINAEVVA-CSVDSQFTHLAWINTPRNQGGL-GPLKIPLLSDLTHKISKDYGVYLEDN------GHTLR--------------------------------------------------------------------------------------------

>PRX4_D.rerio_5

SKAKISKPAPHWEGTAVINGEFKELKLSDY-KGKYLVFFFYPLDFTFVCP-TEIIAFSDRVHEFQAINAEVVA-CSVDSQFTHLAWINTPRKQGGL-GPMKIPLLSDLTHQISKDYGVFLEDQ------GHTLRGLFIIDGKGVLRQITMNDLPVGRSVDETLRLVQAFQYTDKHGEVCPAGWKPGSDTII-PDPAGK------------------LKYFDLN---

>PRX4_X.tropicalis_4

SKAKISKPAPYWEGTAVINGEFKELKLTDY-KGKYLVFFFYPLDFTFVCP-TEIIAFGDRIEEFRSINTEVVA-CSVDSQFTHLAWINTPRKQGGL-GPMKIPLLSDLTHQISKDYGVYLEDQ------GHTLRGLFIIDDKGVLRQITMNDLPVGRSVDETLRLVQAFQYTDTHGEVCPAGWKPGSETII-PDPAGK------------------LKYFHQH---

>PRX4_H.sapiens_8

SKAKISKPAPYWEGTAVIDGEFKELKLTDY-RGKYLVFFFYPLDFTFVCP-TEIIAFGDRLEEFRSINTEVVA-CSVDSQFTHLAWINTPRRQGGL-GPIRIPLLSDLTHQISKDYGVYLEDS------GHTLRGLFIIDDKGILRQITLNDLPVGRSVDETLRLVQAFQYTDKHGEVCPAGWKPGSETII-PDPAGK------------------LKYFDLN---

>PRX4_C.teleta_7

SKAQISKPAPEWKGTAVINGEFKDLSLSDY-KGKYLVFFFYPLDFTFVCP-TEIIAFSDRVKEFQAINAEIVA-ASVDSPFTHLAWMNTPRNQGGL-GKMNIPLLSDLSHKISKDYGVYLENV------GHTLRGLFIIDPKGILRQITMNDLPVGRSVDETMRLVQAFQYTDQHGEVCPAGWKPGSDTII-PDPSDK------------------LKYFKVNA--

>PRX4_B.floridae_1

SKAQISKPAPDFQGTAVVNGKFEEIKLSDY-KGKYLVFFFYPLDFTFVCP-TEIIAFNERVEEFRKVNTEVVG-VSVDSQFTHLAWINTPRKAGGL-GPMNFPLLSDLTHKISRDYGVLLEDV------GHTLRGLFIIDDKGILRQITMNDLPVGRSVDETLRLVQAFQYTDQHGEVCPAGWTPGADTII-PNPNDK------------------LKYFEANK--

>PRX4_B.floridae_2

----VSKPAPDFQGTAVVNGKFEEIKLSDY-KGKYLVFFFYPLDFTFVCP-TEIIAFNERVEEFRKVNTEVVG-VSVDSQFTHLAWINTPRKAGGL-GPMNFPLLSDLTHKISRDYGVLLEDV------GHTLRGLFIIDDKGILRQITMNDLPVGRSVDETLRLVQAFQYTDQHGEVCPAGWTPGADTII-PNPNDK------------------LKYFEANK--

>PRX4_C.intestinalis_2

TKAQISKPAPDWEGTAIVDGEIKTIKLGDY-KGKYLIFFFYPLDFTFVCP-TEIIAFSDRVAEFKKINAEVVA-ASVDSHFTHLAWLNTHRSEGGL-GKLNIPLLSDLTHKISRDYGVLLEDL------GHTLRGLFIIDPKGILRQITMNDLPVGRSVDETLRLVQAFQHTDEHGEVCPAGWEPGKDTII-PDPKDK------------------LKYFHTSK--

>PRX4_M.leidyi_1

VKARISKPAPDFTAAAVVNGKFEDITLSSF-KGKYVVLFFYPLDFTFVCP-TEIIAFNDRVGEFKKINTEVIA-ASVDSKFTHLAWINTPRNKGGL-GDMDIPIISDLTRQISRDYGVMLEDE------GHTLRGLFIIDDKGILRQITMNDLPVGRSVDETLRLVQAFQYTDQHGEVCPANWTPGKDTLK-PDPEGK------------------LEYFENCN--

>PRX4_A.queenslandica

SKAQIAKPAPDWNGTAVVGAAFKELRLSDF-KGKYLVFFFYPLDFTFVCP-TEITAFSDRVGEFKAINTEVVA-CSVDSKYTHLAWIKTPRDKGGL-GELNIPLLSDITKQISRDYGVLLEDE------GISLRGLFIIDARGILRQITMNDLPVGRSVDETLRLVQAFQYTDQYGEVCPAGWKPGEQTIV-PDPEGA------------------QKYFKCND--

>PRX4_X.bergquistia_1

SKAQIAKAAPDWNGTAVVDPSFKDLKLVDF-KGKYLVFFFYPLDFTFVCP-TEIIAFSDRIKEFKDINTEVVA-CSVDSKYTHLAWIKQPRDKGGL-GHLEIPLLSDITKQISRDYGVLLEDE------GISLRGLFIIDPRGILRQITMNDLPVGRSVDETLRLVQAFQYTDKHGEVCPAGWKPGEKTII-PDPEGS------------------QKYFECNN--

>PRX4_T.wilhelma_1

SKTQISKPAPDWNGTAVINGEFKELKLSDF-RGKYLVFFFYPLDFTFVCP-TEILAFSDRVEEFKAINADVVA-CSVDSKYTHFAWISMPREEGGL-KGLKIPLLSDLTKQISKDYGVLLEDA------GHTLRGLFIIDHKGILRQITMNDLPVGRSVDETLRLVQAFQFTDQKGEVCPAGWKPGEMTIK-PDPVGK------------------NEYFSAFC--

>PRX4_T.wilhelma_2

SKTQISKPAPDWNGTAVINGEFKELKLSDF-RGKYLVFFFYPLDFTFVCP-TEILAFSDRVEEFKAINADVVA-CSVDSKYTHFAWISMPREEGGL-KGLKIPLLSDLTKQISKDYGVLLEDA------GHTLRGLFIIDHKGILRQITMNDLPVGRSVDETLRLVQAFQFTDQKGEVCPAGWKPGEMTIK-PDPVGK------------------NEYFSAFC--

>PRX4_E.muelleri_1

SKTQISKPAPDWNGTAVVNRDFVELKLSDF-KGKYLVFFFYPLDFTFVCP-TELLAFSDRLDEFKALNTAVVA-CSVDSKYTHLAWLNTKRAEGGL-EGLRIPLLADLTKQISKDYGVLLEDL------GHTLRGLFIIDDRGILRQITMNDLPVGRSVDETLRLVQAFQYTDVKGEVCPAGWKPGEKAII-PDPVAK------------------KEYFTENC--

>PRX4_N.vectensis_4

SKTAIQKPAPAFSGTAVNHGEFIDLKLSDY-KGKYVVLFFYPLDFTFVCP-TEIIAFSDRVDEFKAINCEVIA-CSVDSEYSHLAWTNVPRKKGGI-GNINIPILSDLTKQISKDYGVLLEDQ------GVALRGLFIIDDKGILRQITINDLPVGRSVDETLRLIQAFQFTDKHGEVCPAGWRPGADTII-PEPQKS------------------SSYFSQ----

>PRX4_D.melanogaster_7

TKAVISKPAPQFEGTAVVNKEIVKLSLSQY-LGKYVVLLFYPLDFTFVCP-TEIIAFSDRIAEFKKIKTEVIG-VSVDSHFTHLAWINTPRKEGGL-GDVKIPLLSDLTHKISKDYGVYLESS------GHALRGLFIIDQTGVLRQITMNDLPVGRSVDETIRLVQAFQYTDTHGEVCPAGWRPGADTIV-PNPEEK------------------TKYFANN---

>PRX1/2_O.carmela_2

SKAFVQREAPAWSGTAVMDGTFKDLKLSDY-RGKYLVFFFYPLDFTFVCP-TEIIAYSERADEFKKRDCEVIA-CSVDSEFSHLAWTNTPRKKGGL-GPMKIPLLSDLTKSISRDYGVLLEDA------GCSLRGLFIIDGKGVLRQITVNDLPVGRSVDETLRLVEAFQFTDENGEVCPANWKPGDDTIK-PDPSGS------------------KDYFSQ----

>PRX1/2_A.planci_1

GKCQITKPAPDFSGPAVMSGEFKDIKLSDY-KGKYLVFFFYPLDFTFVCP-TELIAFSDRAKEFRDIGCELLA-CSCDSQYSHLAWTNTPRKKGGI-GNMSIPLLADKSCRIAKDYGVLIEED------GVSFRGLFIIDNKGILRQITINDLPVGRSVDETLRLVQAFQFTDKHGEVCPAGWRPGAETIK-PDVKKS------------------QDYFSKN---

>PRX1/2_C.intestinalis_1

GKACIQKSAPDFTATAVVNGDFRDISLSEY-KGKYVVLFFYPLDFTFVCP-TEIIAFSDRVSEFRDIGCEVLA-CSTDSHFSHLAWTNIPRKKGGI-GNMKIPLIADKNCAISKDYGVLMEGS------GIAFRGLFIIDTMGILRQITINDLPVGRSVDETLRLVKAFQFTDQHGEVCPAGWKPGDDTIK-PDVQDS------------------QKYFSKQ---

>PRX1/2_B.floridae_6

GKAKLQHPAPNFESTAVLSGEFGTIKLSDY-KGKYLIIFFYPMDFTFVCP-TEIIAFSDRVEEFKKINCEVLA-CSTDSQFSHLAWTNTPRKQGGL-GQMKIPLMADKAMTISRDYGVLMEDA------GIAFRGLFIIDDKGTLRQITINDLPVGRSVDETLRLVQAFQFTDKHGEVCPAGWKPGADTIK-PDVKDS------------------KEYFSKQ---

>PRX2_D.rerio_1

GNAKIGQPAPQFKATAVVDGQFKDIQLSDY-RGKYVVLFFYPLDFTFVCP-TEIIAFSERAAEFRKIGVELIA-ASTDSHFSHLAWINTPRKQGGL-GSMNIPLVADLTQSISRDYGVLKEDE------GIAYRGLFVIDDKGILRQITINDLPVGRSVDETLRLVQAFQHTDKYGEVCPAGWKPGSDTIV-PDVQKS------------------KEFFSKQ---

>PRX1_D.rerio_3

GNAHIGKPAPDFTAKAVMDGQFGDVRLSDY-KGKYVVLFFYPLDFTFVCP-TEIIAFSDAAEEFRKINCEIIG-ASVDSHFCHLAWTKTPRKQGGL-GPMNVPLVADTLRSISKDYGVLKEDE------GIAYRGLFIIDDKGILRQITINDLPVGRSIDETLRLVQAFQFTDKHGEVCPAGWKPGKDTIK-PDVNQS------------------KDFFSKQN--

>PRX1_X.tropicalis_5

GNAKIGHPAPDFTAKAVMDGQFKDLKVSDY-KGKYVVFFFYPLDFTFVCP-TEIIAFSDRVEEFKKLNCEVIG-ASGDSHFCHLAWISQPRKEGGL-GKMNIPLVSDVQHTIAKDYGVFEEKE------GVSFRGLFIIDEKGILRQITINDLPVGRSVDETLRLVQAFQFTDKYGEVCPAGWQPGSDTIK-PDVKKS------------------KEYFNKQK--

>PRX1_H.sapiens_2

GNAKIGHPAPNFKATAVMDGQFKDISLSDY-KGKYVVFFFYPLDFTFVCP-TEIIAFSDRAEEFKKLNCQVIG-ASVDSHFCHLAWVNTPKKQGGL-GPMNIPLVSDPKRTIAQDYGVLKADE------GISFRGLFIIDDKGILRQITVNDLPVGRSVDETLRLVQAFQFTDKHGEVCPAGWKPGSDTIK-PDVQKS------------------KEYFSKQK--

>PRX2_H.sapiens_5

GNARIGKPAPDFKATAVVDGAFKEVKLSDY-KGKYVVLFFYPLDFTFVCP-TEIIAFSNRAEDFRKLGCEVLG-VSVDSQFTHLAWINTPRKEGGL-GPLNIPLLADVTRRLSEDYGVLKTDE------GIAYRGLFIIDGKGVLRQITVNDLPVGRSVDEALRLVQAFQYTDEHGEVCPAGWKPGSDTIK-PNVDDS------------------KEYFSKHN--

>PRX1/2_C.teleta_5

SELKLTKPAPAFSGTAIVDGDFKEISISDY-KGKYLVFFFYPLDFTFVCP-TEIIAFSDRVEEFRSINCEVVA-CSTDSAFSHLAWTQQPRNKGGL-GNMNIPLLADKTLDIATRYGVLKEDE------GIAFRGLFIIDDKGNLRQVTINDLPVGRSVDEVLRLVQAFQFTDKHGEVCPAGWKPGAATMK-PDTKES------------------KSYFQKNN--

>PRX1/2_L.anatina_3

TKLRIGKPAPNFSGTAVIDGDFKTVKLDDY-RGKYLVFFFYPLDFTFVCP-TEIIAFSDRSEEFRSIGCEVVA-CSTDSHFSHLAWVNTPRKKGGL-GQMKIPLLADKTMEIAKAYGVLKEED------GVTFRGLFIIDGQGILRQVTVNDLPVGRSVDETLRLVQAFQFTDKHGEVCPAGWRPGADTMK-PDPKGS------------------QTYFEKTN--

>PRX2_X.tropicalis_2

VKTHIGQPAPAFKATAVVNGEFKDIQLSDY-LGKYVVLFFYPLDFTFVCP-TEIIAFSDHAGDFSKINCQLIA-VSVDSQFTHLAWTNVPRKEGGL-GPINIPLVSDLTHSIAKDYGVLKEED------GVAYRGLFIIDGKGNLRQITINDLPVGRSVEETLRLVQAFQYTDQHGEVCPAGWKPGSSTIK-PNVKDS------------------KEFFSKEY--

>PRX4_S.ciliatum_1

SKAKIGQPAPFWSGTAVTAGKFQDMNLTDF-KGKYLVMLFYPLDFTFVCP-TEILAFSDRLAEFRQINAEIVA-ISVDSKFTHLAWTKMSRREGGL-GKVHIPLLSDLTHQISKDYGVFLEKE------GHTLRGLFIIDNHGVLRQITMNDLPVGRSVDETIRLIQAFRYTDEHGEVCPAGWKPGAATIV-PDPEGK------------------KAYFEANP--

>PRX1/2_C.elegans_1a

SKAFIGKPAPQFKTQAVVDGEFVDVSLSDY-KGKYVVLFFYPLDFTFVCP-TEIIAFSDRAEEFKAINTVVLA-ASTDSVFSHLAWINQPRKHGGL-GEMNIPVLADTNHQISRDYGVLKEDE------GIAFRGLFIIDPSQNLRQITINDLPVGRSVDETLRLVQAFQFVEKHGEVCPAGWTPGSDTIK-PGVKES------------------QEYFKKH---

>PRX1/2_C.elegans_1b

SKAFIGKPAPQFKTQAVVDGEFVDVSLSDY-KGKYVVLFFYPLDFTFVCP-TEIIAFSDRAEEFKAINTVVLA-ASTDSVFSHLAWINQPRKHGGL-GEMNIPVLADTNHQISRDYGVLKEDE------GIAFRGLFIIDPSQNLRQITINDLPVGRSVDETLRLVQAFQFVEKHGEVCPAGWTPGSDTIK-PGVKES------------------QEYFKKH---

>PRX1/2_C.elegans_1c

SKAFIGKPAPQFKTQAVVDGEFVDVSLSDY-KGKYVVLFFYPLDFTFVCP-TEIIAFSDRAEEFKAINTVVLA-ASTDSVFSHLAWINQPRKHGGL-GEMNIPVLADTNHQISRDYGVLKEDE------GIAFRGLFIIDPSQNLRQITINDLPVGRSVDETLRLVQAFQFVEKHGEVCPAGWTPGSDTIK-PGVKES------------------QEYFKKH---

>PRX1/2_D.melanogaster_8

--PQLQKPAPAFAGTAVVNGVFKDIKLSDY-KGKYLVLFFYPLDFTFVCP-TEIIAFSESAAEFRKINCEVIG-CSTDSQFTHLAWINTPRKQGGL-GSMDIPLLADKSMKVARDYGVLDEET------GIPFRGLFIIDDKQNLRQITVNDLPVGRSVEETLRLVQAFQYTDKYGEVCPANWKPGQKTMV-ADPTKS------------------KEYFETTS--

>PRX3_A.planci_2

LNVGVQHAAPSFVGTAVVNGDFKTVRLEDY-RGKYLVLFFYPLDFTFVCP-TEIIAFSERASEFHQLNTEVVG-VSVDSHFSHLAWINTPRKEGGL-GPMNIPLLSDFKKEISKDYGVLLYEE------GVALRGLFIIDPDGIVRHTSVNDLPVGRSVDETLRLVKAFQFVAKHGEVCPAGWTPDSPTIK-PDPHGS------------------KTYFEEVN--

>PRX3_S.purpuratus_3

LNVAIQEPAPDFEGTAVIDGQFKEIKLSDY-KGKYLVLFFYPLDFTFVCP-TEIIAFSDRADEFGAINTEVVA-ASIDSHFSHLAWINTPRKQGGL-GPMKIPLLSDMKKQIAEDYGVLLKDA------GVALRGLFLIDPEGVVRHMSINDLPVGRSVDETLRLVKAFQFVAEHGEVCPAGWTPDSETIK-PDPEGS------------------KTYFEKVN--

>PRX3_D.rerio_2

WAPAVTQAAPHFKGTAVINGEFKEISLGDF-KGKYLVLFFYPLDFTFVCP-TEIVAFSDKANEFHDVNCAVVG-VSVDSHFTHLAWTNTPRKSGGL-GKIQIPLLADLTKQVSRDYGVLLEGP------GIALRGLFIIDPNGIVRHMSVNDLPVGRSVEETLRLVKAFQFVETHGEVCPASWTPKSPTIK-PTPDGS------------------KEYFEKVN--

>PRX3_X.tropicalis_6

FLPAVTQHAPHFKGTAVVNGEFKELSLEDY-KGKYLVLFFYPLDFTFVCP-TEIVAFSNKANEFHDVNCEVVA-VSVDSHFCHLAWTNTPRKSGGL-GQMNIPLLSDLNKQISRDYGVLLETP------GIALRGLFIIDPNGIIKHMSVNDLPVGRSVEETLRLVKAFQFVETHGEVCPANWTPDSPTIK-PSPEGS------------------KDYFEKVH--

>PRX3_H.sapiens_7

HAPAVTQHAPYFKGTAVVNGEFKDLSLDDF-KGKYLVLFFYPLDFTFVCP-TEIVAFSDKANEFHDVNCEVVA-VSVDSHFSHLAWINTPRKNGGL-GHMNIALLSDLTKQISRDYGVLLEGS------GLALRGLFIIDPNGVIKHLSVNDLPVGRSVEETLRLVKAFQYVETHGEVCPANWTPDSPTIK-PSPAAS------------------KEYFQKVN--

>PRX3_C.intestinalis_4

MVAQVTQPAPPFKGMSVVEGKFKEISLEDY-KGKYLVLFFYPLDFTFVCP-TEIISFSDKSPEFEKLDTVVVG-ASVDSHFSHLAWINTPRKQGGL-GEMKIPLLSDLTKNISRDYGVLLENA------GIALRGLFIIDPSGTIRHASVNDLPVGRSVDEVLRLVQAFQFVDKHGEVCPASWTPGSKTIK-PSVDGS------------------KTYFEEAN--

>PRX3_C.teleta_3

LAARVQHAAPFFKGQAVVDGQFQEVNLEDF-KGKYLVLFFYPLDFTFVCP-TEIIAFSDRINEFKELNAEVVG-VSTDSHFSHLAWINMPRKQGGL-GGLQYPLLSDFSKNISKDYGVLVENA------GIALRGLFLIDPTGTVRQVTINDLPVGRSVDETLRLIKAFQFVEKHGEVCPANWTSESETIK-PNPTDS------------------LEYFGKVN--

>PRX3_L.anatina_1

FEAKVQQPAPDFKGEAAVDGAIKELNLTDY-RGKYLILLFYPQDFTFVCP-TELIAFSDRIAEFHAINTEVIG-VSTDSVFSHLAWINTPKKEGGL-GECKYPLLSDFKKTIATDYGVLLEEK------GVALRGLFLIDPNGLVRHMTVNDLPVGRSVDEALRTVQAFQFVEEHGEVCPANWQPDSPTIT-PTLEGS------------------REYFTQVN--

>Trichoplax.adhaerens_6

RFCGVSQTAPDFKGTAVINGEFQEIQLSDY-AGKYVVLFFYPMDFTFVCP-TEILAFSDRAKEFEELNTQVIA-CSIDSEYSHLAWTTASRKDGGLGGNLNIPLLADITKKISNDYGVLLQNA------GISLRGLFIIDGNGTLRQATVNDLPVGRSVDETLRLVKAFQFTDKHGEVCPANWQPGSQTIK-PDPKDS------------------KEYFSKQ---

>PRX3_D.melanogaster_5

AAVRVQQPAPDFKGLAVVDNSFQEVKLEDY-RGKYLVLFFYPLDFTFVCP-TEIVAFSERIKEFHDINTEVLG-VSVDSHFSHLTWCNVDRKNGGV-GQLKYPLLSDLTKKISADYDVLLDKE------GISLRGTFIIDPNGILRQYSINDLPVGRSVDEVLRLIKAFQFVEQHGEVCPANWNPNSATIK-PDVEES------------------KKYFSKHG--

>PRX3_M.leidyi_3

HVASIPNPAPAFTAQAVMNGTFQEVSLSNY-EGKYVILFFYPLDFTFVCP-TEITAFSDRAAEFQELGCELLA-CSVDSHFSHLAWMKQSRKEGGL-GEMKIPILADLNKTIAKDYGVLLEPV------GIALRGLFIIDPAGNLRHITVNDLPIGRSVDETLRTLRAIQFVEEHGEVCPANWQPGKSTIK-PGVDSS------------------KEYFEKEN--

>PRX3_C.elegans_2

LSLGPKNTVPAFKGTAVVDGDFKVISDQDY-KGKWLVMFFYPLDFTFVCP-TEIIAYGDRANEFRSLGAEVVA-CSCDSHFSHLAWVNTPRKDGGL-GDMDIPLLADFNKKIADSFGVLDKES------GLSYRGLFLIDPSGTVRHTTCNDLPVGRSVDETLRVLKAFQFSDKHGEVCPADWHEDSPTIK-PGVATS------------------KEYFNKVN--

>PRX1/2_E.muelleri_4

PVARVQGKAPHWSGTAVINGDFEEISLDKY-AGEYLVFFFYPNDFTFVCP-TEIIAFSNRLGEFRKNKCNIVA-CSCDSQYVHLAWVETPTSKGGV-GQLDIPLLADVTKKISRDYGVLLEDQ------GVSLRGLFIIDGKGTIRHMSINDIQVGRCVDEVLRLVRAFQYTDIHGEVCFLNG-------------------------------------------

>PRX1/2_S.ciliatum_3

PRARVQKSAPAFKGKALMNGIFRTISLEQY-VGKYLVLFFYPGDFTFVCP-TEIIEFSERCEEFKNLHCEIVA-CSVDSEYCHKAWTGVPRSKGGL-GKMNIPILADPTRQIAQAYDVLVEDD------GVALRGLFIIDPKGIVRQITVNDLEVGRNVDEVKRLVEAFQYSDKHGSVCPQGWTAGKKTIK-PDPQGA------------------LEFFEVP---

>PRX4_M.leidyi_4

SKAFITKPAPDFDLEAVLTEEFERVKLSDY-KGKYLVLFFYPMDFTFVCP-TEIIAFSDRIEEFRKLDCEVVA-ASCDSVYCHYAWCCQSRKAGGL-GKMNIPLLADVTHSLSKDYGVYLEDE------GVSLRGLFIIDGKGILRQSTINDLPVGRSVDEVLRLLQAFQFTDKYGEVCPAERC--NPQIL-PQGRPA------------------RAQTT-----

>Monosiga brevicollis_5

--------------------------------------MFVPLAFTFVCP-TEVLAFERAIDQFKAHNTQLLI-VTVDSVYTLKVWTQTPTNEGGL-GAVSMPLVSDLTHSISADYGVLLRDA------GHSLRGLFIIDDKGILRQSTVNDLPVGRSVEETLRLVKAFQHTDEHGVVCPANWQPGDDTII-PTPDDK------------------LDYFKRH---

>Monosiga.brevicollis_4

PTARIGKPAPDFKLPACLNSEVGEISLEQF-KGKYLVIAVYPLDWTFVCP-TEILAFNDRVQEFRDANCEVIV-GSIDSEFSHLAWAQHPRKDGGL-APMSIPMFADKAHTFTKALGCYVEEE------GCALRGLYIIDDKGILRNITMNDFPVGRNVDEVLRLVKAFQFTDKHGEVCPANWTPGADTIV-PHPAKK------------------AKYFNKVNE-

>Chondrus.crispus_1

NGIRVGQEAPDFSAIAVYDQEFKHIQLSDY-IGKYVILLFYPLDFTFVCP-TEITAFSDVYSQLKALNTEILG-ISVDSQYSHLAWLQTNRDSGGL-GDLKYPLVSDLTKQISLSYNILTEQ-------GTSLRGLFIIDKEGIIQHSLVNNLDVGRSVDETLRTLQAIQYVQVNDEVCPANWQPGNATII-SSPEKS------------------REYFSSV---

>Pyropia.yezoensis_1

NCLRVGQLAPDFSATAVYDQEFKTLKLSDL-KNKYIVLFFYPLDFTFVCP-TEITAFSDKYNAFSELNTEVLG-VSVDSEYSHLAWLQTDRESGGL-GDLSYPLVSDLKKEISAAYNVLNSD-------GVALRGLFIIDPKGIIQYSTINNLEFGRSVEETLRVLQAIQYVQSHDEVCPANWKPGDKTMN-PDPIKS------------------KNYFAAA---

>Pyropia.yezoensis_2

NCLRVGQLAPDFSATAVYDQEFKTLKLSDL-KNKYIVLFFYPLDFTFVCP-TEITAFSDKYNAFSELNTEVLG-VSVDSEYSHLAWLQTDRESGGL-GDLSYPLVSDLKKEISAAYNVLNSD-------GVALRGLFIIDPKGIIQYSTINNLEFGRSVEETLRVLQAIQYVQSHDEVCPANWKPGDKTMN-PDPIKS------------------KNYFAAA---

>Pyropia.haitanensis_3

NCLRVGQLAPDFSATAVYDQEFKTLKLSDL-KNKYIILFFYPLDFTFVCP-TEITAFSDKYDAFSELNTEVLG-VSVDSEYSHLAWLQTDRESGGL-GDLAYPLVSDLKKEISAAYNVLNSD-------GVALRGLFIIDPKGIIQYSTINNLEFGRSVEETLRVLQAIQYVQSHDEVCPANWKPGDKTMN-PDPIKS------------------KNYFAAA---

>Monosiga.brevicollis_3

AEARVTSPAPFFKADALVNTEFKTVSLDDY-KDKYLVLLFYPLDWTFVCP-TEITAHADAQEEFAKLNAEVVA-VSTDNKFSHYAWAQHPRKEGGL-APITMTMIADQTRAMSRTYGCYVPDD------GFNLRATYIIDKSGVLRHAQITDRSVGRSVDETLRIIKALLFAEEHGEVCPANWQPGSATIK-GDPELK------------------KEYFSTNA--

>Algoriphagus.machipongonensis_2

M-ALVGKKAPAFSAGAVINGEVENFNLDQYLGKKNVVLFFYPKDFTFVCP-TELHAFQSKLAEFEKRDTVVIG-CSTDTEETHLAWLMTPKAEGGI-ESVTYPIIADASKTISLNYGVLAGEYSSFDGAPVAYRGTFLIDKEGVVRHESINDLPLGRNIDEYLRILDAQIHVEKFGEVCPANWEEGKEAMQ-ATNEGV------------------ATYLSNN---

>Dictyostelium.discoideum_1Cys

MSLRIGDVVPDFSQDS----SVGQINLYKTLGDSWGLFVSHPKDFTPICT-TELGRLAKLKPEFEKRNCKILA-LSVDSVKDHLEWMKDIEETQKV--KINYPIIADQDRKVADLYGMIHPNADNT----FTVRSVFFISPDKRLRAQITLPASTGRNFNEIIRILDSFQLTDKYKVATPADWVDGDDCIIVPTVFDEDAKKLFPKGFPKI-----KSYLRVTPQP

>Chondrus.crispus_PRX3

MTLNLGDTVPNFTQKS----SEGDIEFYKYLGDSWGILFSHPKDFTPVCT-TELGTVAKLKPEFEKRNVKVIA-LSVDSVEDHAGWIKDINETQST--EVNYPILADQDRSVANLYGMIPPAAENT----LTVRSVFIIDPNKKLRLSLTYPASTGRNFDELLRVIDSLQLTEYEKVATPANWTQGDDVVIVPSLQDKELEKRFPQGWTEV-----KPYLRLTAQP

>Algoriphagus.machipongonensis_1

MALRLGDVAPNFTAET----SEGKIDFYEYLGDGWGVLFSHPADYTPVCT-TELGTVAKLKDEFAKRNTKVLA-LSVDGLESHKGWISDINETQNC--TVNFPIIADEDKKVSSLYDMIHPNSNEK----FTVRSVFVIGNDKKIKLIITYPASTGRNFDELLRVIDSLQLTANYSVATPANWKQGEDVVIAPAISNEEIPSKFPKGHKVV-----KPYLRTTPQP

>Prx6_O.carmela_7

MTIRLGDIAPDFKQNS----TEGEISFHEWLGDSWGVLFSHPKVFTPVCT-TELGEVARLKESFSKRNVRVLA-LSVDKAEQQKEWEADIAETQGQ---------------------------------------------------------------------------------------------------------------------------------T

>Podospora.anserina_1

MALRLGTIAPNFQAET----TKGPIDFHEFIGDNWVILFSHPEDYTPVCT-TELGEMARLEPEFSKRGVKLIG-LSANTLGSHEGWISDIKDVTGS--QVNFPIIADKERKVAYLYDMIDYQDTDEKGIAFTIRSVFFIDPKKTIRCILSYPASTGRNSAEILRVIQSLQTGDKHKVTTPINWVPGDDVIVHPSIKGDEATKLFPN------LRAVKPYLRFTPLP

>Chondrus.crispus_2

MAPLLGEIAPNFSAET----THGPIDFHTYIESSWAVLFSHPADFTPVCT-TELARVSQLLPEFEARNVKVLA-LSCDDLQSHTGWLADISAYGKGCPMEGFPIIADASRDVARLYGMIPADHPDDKGMPMTVRSVFVVGLDKKIKLSLTYPASTGRSFDEILRVIDSLQLAVSHKVATPVDWTKGKDCVVLPSVSNDKAQELFPKGFKPAELPSGKQYLRMTPQP

>Monosiga.brevicollis_2

APPNLGDVMPNAELET----TEGTFKFHDFIGDKWAILFSHPADYTPVCT-TELGTLAKLVPEFDKRNTKVIA-LSVDSIEDHKGWSKDVSAFACI-DTLPFPIIADKNRDLAVALGMLDPDEQTKEGLPATARCVFIVGPDKKLKLSILYPATTGRNFNEVLRVLDSLQLTAYHRVATPANWQPGDECMVTPGVKVEEQEKVFPKGVRTQEVPSGKAYLRFTPQP

>Prx6_D.rerio_6

PGILLGDVFPNFEADT----TIGKIKFHEFLGNSWGILFSHPRDFTPVCT-TELARAAKLHEEFKKRDVKMIA-LSIDSVEDHRKWSEDILAFNQDACCMPFPIIADDKRELSVLLGMLDPDERDKDGMPLTARCVFVVGPDKRLKLSILYPATTGRNFDEILRVVDSLQLTATKKVATPVDWKPGQEVMVIPSLSDEEANKLFPAGFTLKEVPSGKKYIRYT-KP

>Prx6_X.tropicalis_3

PGLLLGEIFPDFEADT----TIGRIKFHEFLGGSWGVLFSHPRDYTPVCT-TELGRCVKLAPEFKKRNVRMIA-LSIDSVEDHLGWSKDINSYNCDPTELPFPIIADPKRDLAVKLGMLDPDEKDMQGMPVTARCVFIIGPDKKMKLSILYPATTGRNFDEILRVVDSLQLTAVHNVATPVDWKPGDRVMVPPNVPEEEASKLYPSGVFNKALPSRKNYLRYTAHP

>PRDX6_MOUSE_NCBI

PGLLLGDEAPNFEANT----TIGRIRFHDFLGDSWGILFSHPRDFTPVCT-TELGRAAKLAPEFAKRNVKLIA-LSIDSVEDHLAWSKDINAYNGEPTELPFPIIDDKGRDLAILLGMLDPVEKDDNNMPVTARVVFIFGPDKKLKLSILYPATTGRNFDEILRVVDSLQLTGTKPVATPVDWKKGESVMVVPTLSEEEAKQCFPKGVFTKELPSGKKYLRYTPQP

>Prx6_H.sapiens_13

PGLLLGDVAPNFEANT----TVGRIRFHDFLGDSWGILFSHPRDFTPVCT-TELGRAAKLAPEFAKRNVKLIA-LSIDSVEDHLAWSKDINAYNCEPTELPFPIIDDRNRELAILLGMLDPAEKDEKGMPVTARVVFVFGPDKKLKLSILYPATTGRNFDEILRVVISLQLTAEKRVATPVDWKDGDSVMVLPTIPEEEAKKLFPKGVFTKELPSGKKYLRYTPQP

>Trichoplax.adhaerens_3

-MVNLGDQLPNFKANT----TQGNIEFHSWLNGKWGILFSHPDDYTPVCT-TELGRAAKLAPEFEKRGVKLIG-LSCNSASSHSGWIKDIEAYSSLSGQFPFPIIADEKRELAVQLGMLDPDEKDSAGLPLTCRAVFIVDQNAKLKLSLLYPATTGRNFDEILRVVDSLKLTVEKKVATPVDWKAGDKCMVIPSVKAEDIPKLFPKGVEIANVPSGKQYIRLTPQP

>Prx6_O.carmela_4

-MPNLGDVFPDFTAES----THGEIKFHDWLGGSWAILFSHPADFTPVCT-TELGLVAKLAPEFQKRGVKMVA-LSCDPVESHNGWVKDIQAFTGAGTEWPYPIIADKNRELAVKFGMLDPDEKSAAGLPLTARVVFIIGPDKKLKLSILYPATTGRNFDEILRVIDSLKLTAEKKVATPANWKQGERCMVLPSVKQEDTPKLFPKGVEVVPVPSGKQYMRFTPQP

>Prx6_A.planci_3

-MVNLGDTFPNFEAET----SVGKIKFHEWLGESWGILFSHPADYTPVCT-TELSRAASLAGEFAKRNVKMIA-LSVDDVDSHVGWIKDIQAYSGQGGEFPFPIIAD-NRDLAVKFGMLDPDERDKAGMPLTARCVFIIGPDKKLKLSILYPATTGRNFDEILRVVDSLQLTATKKVATPVDWKEGGDCMVIPSVKQEEVAQLFPKGVTVKPVPSGKSYLRITPQP

>Prx6_N.vectensis_3

-MPNLGDEFPNFTADS----TIGTINFHDFIKDSWAILFSHPADYTPVCT-TELGRVAQLEPEFKKRGVKMAG-LSCDDAESHRGWVKDITKYNLESSAFNYPIIADERRELAVKLGMVDPDEKDSKGLPLTCRAVFIIGPDKKLKLSILYPATTGRNFDEILRVIDSLQLTATKKVATPVDWKLGGDCMVIPSIKPEEEGTIFPKGVRALDLPSGKRYLRYTPQP

>Prx6_S.ciliatum_4

MGINLGDVFPDFTANT----TKGSISFHSFLGDSWGILFSHPADFTPVCT-TELGRVNQLLAEFEKRNVKPIA-LSCDPVDKHSDWIKDIQAYSAQSGDWGYPIIADEQRELAVRFGMLDPDEKDKAGMPLTARCVFIIGPDKKLKLSLLYPASTGRNFDEIIRVIDSLQLTAYKKVATPVNWQPGGECMVLPSVKPEDAKTLFPEHY-TKELPSGKGYMRFTPQP

>Prx6_A.queenslandica_1

MPLNLGDAFPNFKADT----TEGQIQYYDWLGDKWGVLFSHPADFTPVCT-TELGAVAKIVPEFEKRNAKVIA-ISCDSVEDHKKWIKDIQAYNGLGDNFPYSIISDPKRELAIQLGMVDPEEKDKAGLPMTCRAVFIIGPDKKLKLQILYPATTGRNFDEIIRVLDSLQLTANKKVATPANWTSGGDCMIVPSVSNDDAAKLFPKGFKVADVPSGKPYIRITPQP

>Prx6_X.bergquistia_5

MPLNLGDTFPNFTADT----TEGTIQFHSWLGDSWGVLFSHPADFTPVCT-TELGEVARIVPEFTKRGTKIIA-LSCNSVDSHKDWIKDIEAYNNLPQGFPYAIISDPNRELAVQLGMVDPVEKDAAGLPLTCRAVFIIAPDKKLKLQILYPATTGRNFDEILRVLDSLQLTAHKKVATPANWKNGGECMILPTVSAEDAAKLFPKFLQVLDLPSNKHYIEATPSP

>Prx6_T.wilhelma_3

MPLNLGDTFPNPDVET----TEGSFKLHDYWGDKWGILFSHPADFTPVCT-TELGAVASIIPEFTKRGTKVIA-ISCDPVDAHKGWIKDIQAYNSLTDEFPYPIISDPNRELAVQLGMVDPEEKDKAGLPLTCRAVFIVGPDKKLKLSLLYPATTGRNFDEILRVIDSLQLTANKRVATPANWKDGGDCMILPNVSKEDADKLFP-GYKSADVPSGKKYIRTTTQP

>Prx6_E.muelleri_2

PLMNLGDDFPNFSVDT----TEGRITFHDFLGNHWGVLFSHPGDYTPVCT-TELAEVANLIPEFTKRDVKVIA-LSCDTVTAHKGWITDIKSYACYARSWPYPLIADPNRDIAIQLGMLDPTAKDKSGIPVTCRAVFIIGPDKKLKLALLYPATTGRNFSEILRVIDSLQLTACKQVATPANWQPGGKCMVLPSISEADASELFPKGTELIKMPSGKHYMRYTPHP

>Prx6_E.muelleri_5

MPLNLGDTFPNFSVDT----TEGRISFHDFLGDSWGVFFSHPSDYTPVCT-TELAEVAKLIPEFEKRNVKVIA-LSCDPVEAHRGWIEDIKCFACYKDKWPYPIISDPSRELAVQLGMIDPDERDKAGMPLTCRAVFFIGPDKKLKLSLLYPSTTGRNFHEILRVIDSIQLTATKKVATPANWQPGGKCMILPTVSQEDAAKLFPKGYDLVSVPSGKQYIRLTPQP

>Prx6_L.anatina_4

-MVNLGDVFPNFDAKT----THGDFKFHDWIGDSWAILFSHPADYTPVCT-TELGRVTKLVPEFNKRGVKLIA-LSCDNVESHKGWIEDIKSYMKDQGDFPYPIISDSDRSLAVSLGMVDPAEKDAAGLPLTCRAV-----------------------SKAYKYVYSWTL-------------------------------------------------------

>Prx6_L.anatina_7

-MVNLGDVFPNFDAKT----THGDFKFHDWIGDSWAILFSHPADYTPVCT-TELGRVTKLVPEFNKRGVKLIA-LSCDNVESHKGWIEDIKSYMKDQGDFPYPIISDSDRSLAVSLGMVDPAEKDAAGLPLTCRAVFIIGPDKKLKLSMLYPATTGRNFTEILRVVDSLQLTVNQKVATPADWVNGGDCMVLPTIKEDEAAKLFPNH-KTIPVPSKKPYLRITPQP

>Prx6_B.floridae_4

PPLNLGDEMPNMTVVT----NEGTIKMHDFLADSWAILFSHPKDYTPVCT-TELGRACTLAPEFAKRKVKMIA-LSCDDADSHNGWIKDVQSHANHKGDFPYQIIADESREVAKKLGMIDPDESAAAGMPLTCRAVMIFGPDKRLKLSMLYPATTGRNFTEILRVIDSLQLTATKKVATPVDWTVGSKCMVVPSVKKEEEAGLFPKGVETLDVPSGKGYLRMTPQP

>Prx6_D.melanogaster_1

SALNIGDQFPNFTAET----SEGRIDFYDWMQDSWAILFSHPADFTPVCT-TELSRVAALIPEFQKRGVKPIA-LSCDTVESHKGWIEDIKSFGKL-SSFDYPIIADDKRELALKFNMLDKDEINAEGIPLTCRAVFVVDDKKKLRLSILYPATTGRNFDEILRVIDSLQLTQTKSVATPADWKQGGKCMVLPTVKAEDVPKLFPDGIETIELPSGKSYLRITPQP

>Prx6_M.leidyi_2

--MKLGQVFPNEKVQT----TQGELQLHDYWGDGWGIFFTHPADYTPVCT-TELSMVQQMVPEFTKRNVKMIA-LSCNSVADHAGWCEDIKSYGKL-SEVSYPIIEDPSRDLAVKFGMLDPEEKDAAGLPLTARAVFIVGPDKKLKLSILYPATTGRNFDELLRVIDSLQLTAYKKVATPANWQAGGDCMVLPSVKGEEATTLFPQ-MRIESVPSGKEYMRFTPQP

>Prx6_C.intestinalis_3

MGINLGQVFPNFDCKT----TEGDLNFHEYIKDSWAVLFSHPADYTPVCS-TELGAAALQHCEFQKRGVKMLE-VSVDSVESHKGWVKDIQVYSMSEKRFPFP-LASVSRQLLSDLGMLDPDEVDSTGLPLTARCVFVIGPDKKLKLSILYPATTGRNFHEILRVIDSLQLTANSKVATPANWKKGEKCRVIPSLSDEEAVKLFPKGFEVTEVPSKKSYIRLTPDP

>Prx6_C.intestinalis_5

MGVNLGQVFPNIDCKT----TKGDYKLHDFINESWSILFSHPADYTPVCT-TELGTAAQLKPEFDARGVKMIG-LSIDSVDSHNGWIKDIQSYAGLQGEFPYPIIAG-TRQTAADLGMLDPDEVDASGMALTARCVFIIGPDKKLKLSLLYPATTGRNFNEIIRVIDSLQLTATKKVATPANWKSGEDCMVVPSLSDAQATELFPKGFKVTEVPSKKSYIRLTPDP

>Prx6_S.purpuratus_2

GTTELGRVAPEFEVES----HRGWIKVRTKIKESWGILFSHPADYTPVCT-TELGRVATLTPEFEKRGVKLIA-LSCDGVESHRGWIKDIVDYAKFEKTWPYPIIADPKRELAVQFGMLDPDEKDSAGIALTARCVFIIGPDKKLKLSLLYPATTGRNFDEILRVIDSLQLTATKRVATPADWKSGEDCMVLPNISEEDAAKLFPQHRKVA-VPSGKGYIRLTPQP

>Prx6_B.floridae_7

-MPNIGDIFPNFRAMT----TEGEIDFYDWLGNSWGILFSHPGDFTPVCT-SELGKAAQLAPEFQKRGIKIIG-LSCSSTREHEAWIPDILAYTGLKGPMPFPIISDEKRELAVGLGMLDPEFKDDKGMPMTCRALFIIGPDKKLKMSILYPALSGRNFSEILRVVDSLQLTDVKKVSTPVDWKYGEDCMVDVSVPRVYEDHLFPKGVTIKALPSGKDYFRTTPMP

>Prx6_C.teleta_4

MT--------------------GRV--------DWGVLFSHPRDFTPVCT-TELGEVTKRAPEFKKRNCKLIA-LSCDGVDDHVAWSEDVMSYVGCNGKLPYPIIADPTRDIATKLGMIDADEKDPSGMPVSCRAVFVVGPDHRLKLSILYPATTGRNFDEILRVIDSLQLTAKKSVATPVDWTPGKPAMVVPSLSPEEAKKMFPKH-EVRSVPSGKGYLRFTPDY

>Prx6_D.melanogaster_2

--MRLGQTVPNFEADT----TKGPIKFHEWQGNSWVVLFSHPADFTPVCT-TELGRIAVHQPEFAKRNTKCLA-HSVDALNSHVDWVNDIKSYCLDIPGFPYPIIADPTRDLAVSLGMLDEEQKKDPEVGKTIRALFIISPDHKVRLSMFYPMSTGRNVDEILRTIDSLQLTDRKVVATPANWTPGTKVMILPTVTDEEAHKLFPKGFDKVSMPSGVNYVRTTDNY

>Prx6_D.melanogaster_3

--MRLGQTVPNFEADT----TKGPIKFHEWQGNSWVVLFSHPADFTPVCT-TELGRIAVHQPEFAKRNTKCLA-HSVDALNSHVDWVNDIKSYCLDIPGFPYPIIADPTRDLAVTLGMLDEEQKKDPEVGKTIRALFIISPDHKVRLSMFYPMSTGRNVDEILRTIDSLQLTDRKVVATPANWTPGTKVMILPTVTDEEAHKLFPKGFDKVSMPSGVNYVRTTENY

>Prx6_D.melanogaster_4

--MRLGQTVPNFEADT----TKGPIKFHEWQGNSWVVLFSHPADFTPVCT-TELGRIAVHQPEFAKRNTKCLA-HSVDALNSHVDWVNDIKSYCLDIPGFPYPIIADPTRDLAVTLGMLDEEQKKDPEVGKTIRALFIISPDHKVRLSMFYPMSTGRNVDEILRTIDSLQLTDRKVVATPANWTPGTKVMILPSVTDDEAHKLFPKGFDKVSMPSGVNYVRTTENY

>Chondrus.crispus_PRX1

MALRLGDVVPNFEANT----THGKIVFHDWLDGKWAILFSHPDDFTPVCT-TEIGRMALKYDSFKAKGVKVVA-LSCNDISSHNVWLKDVVAHCDNKIGIDFPMIADPTREIAIKFGMLDPTNKDTGSMPLTARSVFIIGPDKKLKLSINYPASVGRNMDEIERVVDALMLSWEKSIATPANWPHNHKVFLLPTVTKDDADKHFPKH-KELDMPSGKPYMRLTPSG

>Prx6_B.floridae_5

PVMSLGDELPNFYLEN----NMESGNLHDFISGSWAVLFSYPRSFTPICT-TELARAAQLAPEFAKRGVKMLA-LSCDNGDVNKDWIQDVKMNAGIEGEFPIRLVADQDRQIAKALGLIDQDQPNDVSMPITCRAIFVIGPDKRLRMSMVYPSSCGHNFEEILRSIDSLFMVESWVVGTPANWRPGDDVMVVPSIPKKEEATRFPKGVTRFSMPSGKDYMRLTSDD

>Monosiga.brevicollis_6

M-PNLGDIFPNFECTS----TRGTLRCHDYIGDGWALFLSHPGDFTPVCT-SELGTLAARYDEFEKRNCKIIC-LSCDPVDRHLEWEKDVMAQAGLDGDLPFPIIADEKRELSVRLGILDDDFKDGFGIPLSARGTFLIDPDRHIKFIATYPGPLGRTCDELLRIIDGLQLIEYRRVCIPMDWQYGQEVMTRPDVTPEEFGTVYPKGVSVQEMPSGKEYMYFTPQP

>Prx6_C.elegans_3

--MKLGDTVPNFTFET----DRKNQTLHNYIGEQWLMLFSHPADFTPVCT-TELAELVKLAPEFRKRHVQILA-ISIDSSETHRDWAKDINSVAQLCGSLPFEIIADTDRSICTELGMIDPDEMNSEGICLSARAVMLFGPDKKLKSKILYPATFGRNFVEILRMVDGVQLGTKAPVATPANWIAGDNVIAQPSLSQERVIQELPDKCKTVPLPSGKSYLRVIEGD

>Chondrus.crispus_3

MSVTVGSPAVDFSLPT---SGGGTLSAADLQAAEFVIIYFYPRDATPGCT-TEAKDFRDLAPQLEQLNATVVG-VSRDSVESHDAFVKDL--------DLNFPLITD-DGTLTEGYGVWKLRKLGDKEMMIVERSTFILRGGKVVKEWR------GVKSAAVVESLKSL---------------------------------------------------------

>Algoriphagus.machipongonensis_3

MGLKKGTQAPNFTLAS---TGDKKVNLSKDFKDQALVLYFYPKDFTPGCT-KEACEFRDQFEAFRDLNIPILG-ISKDDIPTHERFKKAHR--------LPFDLLSDPSGKVCKAYDALIPL------IKMPKRITYLLDENHMIIESFSDMFEAKKHINKMLRSLNA----------------------------------------------------------

>Chondrus.crispus_4

MLLSKGDKMPDVDVLK--DGAPTTVSTASIFQSKKVAVVTFPGAFTSTCQNGHVPLWIKAVDDFKAKGCDDVVALAVNDPFVMDAFANVIGNEGK------VTFLADGGAKLTKAIGIEVDT--DGFGGVRSYRGGYLVE-DGVFTQVNLEDGTSFEGPSKPETLLAQM---------------------------------------------------------

>Podospora.anserina_2

RFIKPGQPLPDVGLHE--SSPGNKVNLAEASKLNKMILIGVPAAFSPACSATHVPGFLAHP---KAEEYDQVAVVSVNDVFVMKAWGDVLNPEGKE----NVRFLADPSGEFTKALDMLWDGK-AIFGNERSKRFTIIVE-GGKVKSVAVEPDNTGTSVSLAENVLGKA---------------------------------------------------------

>Monosiga.brevicollis_1

MALKVGDTIPDATVSE--TDLYTTLKLRELFAGKKGILFGIPGAFTPGCHKTHLPGYVQRAEELKGKGIDVIACMGVNDPFVMAGWGETVGATGK------VRMLADKDASASKALGVYWEGSEAIFGSGRCKRFSMLIE-DNIIKVINVEPDNGGPSCSLVEPL-------------------------------------------------------------

>Trichoplax.adhaerens_PRX5a

LQIQVGDKLPSIALHQ--NSPGNKVDIRQLFANKKGILFAVPGAFTPGCSKTHLPGYLQHYDNFKSKGIDVIACVSVNDAFVVDAWSKSNNVDDR------LEMLADTSAQFTKSVGLDFDAT-PVLGNIRSKRYAMIIE-DTVVKQINVEPDGTGLSCSLAQNILEQLS--------------------------------------------------------

>Prx5_A.queenslandica_5

MPIQVGQTLPSIELHE--GTPKDKVNILELFKGKKGILFAVPGAFTPGCSQTHLPGYVNDYLKLKAKGFEVIACVSVNDAFVMSAWGIERKATGK------IRMLADPAGEFTKAVDLGFDAT-PALGNIRSKRYAMTIE-DGVVKSVAIEPDATGLTVSLSCSILNE----------------------------------------------------------

>Prx5_X.bergquistia_2

MPLKVGDSLPSVEVHE--GTPKDKVNILELFKGKKGILFAVPGAFTPGCSKTHLPGFVEDFDKLKASGFDVIACLSVNDAFVMSAWGESAQATGK------IRMLADATGEFTKAIDLELDAT-SALGNIRSKRYSMVIE-DGVIKELNVEPDGTGLTVSLSCNLVKK----------------------------------------------------------

>Prx5_X.bergquistia_3

MPIKVGDTLPSIEVHE--GTPEEKVNILELFKGKKGILFAVPGAFTPGCSKIHLPGYVEDFDKLKA---NIQIHLPGNDTYVMSAWGESAQATGK------IRMLADPYREFTKAVDLELDPS-PALGNIRSKRYSMVVE-DGVVKELNIEPDSTGLNVSLSSMFISRL---------------------------------------------------------

>Prx5_O.carmela_5

MSIKVGDKLPSVNLHE--GTPGCSVNIADLFAKKKGILFAVPGAFTPGCSKTHLPGYVSDYEKMKGKGVDIIVCVSVNDAFVMSAWGEASNTPGK------IRMLADTTAEFTKKIGMDFDAF-AFLGSARSKRYSMVIE-DGTVKTLNVEPDGKGLTCSLSNEILSQL---------------------------------------------------------

>Prx5_S.ciliatum_5

MPIQVGDKLPSVSLHE--GSPKGTVNIADLFKGKKGVLFAVPGAFTPGCSKTHLPGYVTDIEKYNAKGVEVIACVSVNDAFVMAAWGEAHGAAGK------VRMLADTTGELTKAMEMDFDAT-PFLGGIRSKRYSMVIE-DGVVKTINVEPDGTGLTCSLSNTILSQL---------------------------------------------------------

>Prx5_N.vectensis_1

MPIKVGEALPSIKVME--GTPKDTVDVASLFKGKKGILFAVPGAFTPGCSKTHLPGYVADFDKIKSKGVDVVACIAVNDPFVMSAWGEANGCQGK------IQMLADVHGEFTKAVDLELDAT-PFLGNIRSKRYAMLVE-DGVVKQLHVEPDGTGLTCSLSNSILSQL---------------------------------------------------------

>Prx5_L.anatina_5

MPIKEGDRLPNIEVLE--KTPNTKVNLGDLFKGKKGVLFGVPGAFTPGCSKTHLPGYVQNFDKLKAKGMDVVACIAVNDPFVMDAWGENQGANGK------IRMLADYKGEFAKAADLEKDLT-GALGSVRCKRFSMVVE-DGVVKKLNVEPDGTGLTCSLADNVLSQL---------------------------------------------------------

>Prx5_C.teleta_2

M--------------E--GTPSDKVSLSDLFKGKKGIVFAVPGAFTPGCSKTHLPGYVEMFDQLKAKGAEVIACVAVNDPFVMAAWGKAHNAEGK------IRMLADPAGEFTKAVDMEIDLS-SALGNVRSQRYSLVIE-DGKVTHVNAEPDGKGLTCSLVNEVVSQL---------------------------------------------------------

>Prx5_T.wilhelma_5

MPIKVGDTLPSIKLHE--GAPNNTVDVKELFAGKKGILFAVPGAFTPGCTKTHLPGYVEDYEAFKAKNVDVIACVAVNDAFVMSAWGESQKAGGK------VRMLADPNGEFTKAVDLGIDV--GVLGGLRSKRYSMIVE-DGVVTHLNVEPDGTGLTCSLSNELLNKM---------------------------------------------------------

>Prx5_B.floridae_3

MPIKVGDKLPGIDLYE--NTPGNKVNVSELFAGKKGILFAVPGAFTPGCSKTHLPGFVSQAGDLKAKGVQVIACVSVNDPFVMEAWGKDQKAEGK------VRMLADSAAEFTKAIGLELDAT-GLLGNIRSKR--------------------------------------------------------------------------------------------

>Prx5_B.floridae_8

MPIKVGDKLPGVDLYE--NTPGNKVNVSELFAGKKGVIFAVPGAFTPGCSKTHLPGFVSRAGDLQAKGVEVIACVSVNDPFVMEAWGRDQKAEGK------VRMLADTGAEFTKAIGLDLDAT-AILGNIRSKRYSMLVE-DGEVKQLNVEPDGTGLSCSLAEEL--KL---------------------------------------------------------

>Prx5_E.muelleri_6

MPIKQGETLPSVELQE--NTPANKVNIRDLFKGKKGVIFGVPGAFTPTCSKSHLPGFVADYDKLKSAGVDVIACVSVNDAFVMSAWGKENGAEGK------VRMLADPTATFTKAIDVAFDAT-EALGGVRSQRYSMVVE-DGVVKAINVEPDKTGLSCSAAKSIISQL---------------------------------------------------------

>Prx5_E.muelleri_7

MPIKKGDTLPSVELEE--GTPDKKVNVRDLFKGKKGVIFGVPGAFTPVCSKSHLPGFVADYDKLKSAGVDVIACVSVNDAYVMSAWRKENAAEGK------VRMLADPSAAFTKAVDLGYDAT-GSLGGVRSQRYSMVVE-DGVVKCVNTEPDKTGLSCSGASSILSQV---------------------------------------------------------

>Trichoplax.adhaerens_PRX5b

MVIAVGDKIPSYRLQQ--GSPGNNVDIAELVANKKAIIFGVPGAFTPGCHRSHLPGYVADYDKIVAKGVDIIICVSVNDAFVVDAWGKSVGAENK------VVMLADPVAAFTKAIGMDLDAT-PILGNIRSKRYSMILS-DGVLTNLNVEPDGTGLTCSLSNSILSQL---------------------------------------------------------

>Prx5_X.tropicalis_1

MSVKVGDQLPNVTVYE--GGPGNKVSIRDVFANKKGVLFGVPGAFTPGCSKTHLPGYVAQAAELKSRGAAVIACISVNDIFVMSEWAKAYDAEGK------VCMLADPCGDFAKACGLLLDKK-ELFGNQRCKRFSMVVE-DGKVKAINVEEDGTGLTCSLAGNIMSQL---------------------------------------------------------

>PRDX5_MOUSE_NCBI

APIKVGDAIPSVEVFE--GEPGKKVNLAELFKGKKGVLFGVPGAFTPGCSKTHLPGFVEQAGALKAKGAQVVACLSVNDVFVIEEWGRAHQAEGK------VRLLADPTGAFGKATDLLLDDS-SLFGNRRLKRFSMVID-NGIVKALNVEPDGTGLTCSLAPNILSQL---------------------------------------------------------

>Prx5_H.sapiens_12

APIKVGDAIPAVEVFE--GEPGNKVNLAELFKGKKGVLFGVPGAFTPGCSKTHLPGFVEQAEALKAKGVQVVACLSVNDAFVTGEWGRAHKAEGK------VRLLADPTGAFGKETDLLLDDS-SIFGNRRLKRFSMVVQ-DGIVKALNVEPDGTGLTCSLAPNIISQL---------------------------------------------------------

>Prx5_D.rerio_4

MPIKVGQRLPAVEVQE--EDPGNSLSMAELFSCKRGVLFGVPGAFTPGCSKTHLPGFIQMAGELRAKGVDEVACISVNDVFVMSAWGKQNGADGK------VRMLADPTGAFTKAVDLVLNNA-PVLGNLRSQRYAMLIE-NGVVTKLSVEPDGTGLTCSLASNFLAEV---------------------------------------------------------

>Prx5_D.melanogaster_6

AMVKVGDSLPSVDLFE--DSPANKINTGDLVNGKKVIIFGVPGAFTPGCSKTHLPGYVSSADELKSKGVDEIVCVSVNDPFVMSAWGKEHGAAGK------VRLLADPAGGFTKALDVTID---PPLGGVRSKRYSLVVE-NGKVTELNVEPDGTGLSCSLANNIGKK----------------------------------------------------------

>Prx5_C.teleta_6

RAIQVGDPLPSVPLFE--KFPGNEVLLADLIGTKKAVVFAVPGAFTPGCTRVHLPGYVDAYDKLRSKGIEVIACIAVNDPFVVTAWGNAAGATGK------IRMLSDPRAEFTKAIGMDFDAR-PLLGTVRSKRYSMLVE-QGKVVQLFAEPDGGGLTCSLAPNLLSRL---------------------------------------------------------

>Prx5_C.teleta_1

MSLSIGQPLPDATLFT--NDGPQTSSVKEIFSAGRIAAFVVPGAFTPACHRNHLPGYLKLRDELLAKGIDKIVCLAVNDAFVLSAWARETAAVGL------ITMISDGNGDFTRAAGMEIDLS-DHGIGQRSRRYSFVTD-KGIVTHLNVE---------------------------------------------------------------------------

>Podospora.anserina_3

MSLKAGDSFPNVTVTA--CGIGIQYDASKEFASKKVVLVAVPGAFTPTCQVSHVTSYLAKLDDLKAKGVDQVIFIASNDHWVMAAWGKANGVKDD-----SILFMSDAGLEFSKSIGWTQGD--------RTLRYAIVVD-HGKVTYAEVDSVRGSIENTGAEGVLAKL---------------------------------------------------------

**Alignment for Fig 5 – GPX unrooted tree including non-metazoan sequences and sequences obtained by Trenz et al. (2021)**

>m.leidyi_gpx4a

MSLPWLKSMEQSGVSC-LEPQEQICDEAECACNIYGFEVKDIDGNTVKLDKYKGYVTLIVNVASEXGLTELNYTQLQQIYSEYKDKGLRILAFPCNQFGKQEPGEHNEIIEFV------SKYGVTFDMFSKVEVNGENAIPLYQYLKSRVSGS-------------------LGSLVRWNFQKFLVDRNGVPKFRYEPM------------FL

>a.queenslandica_gpx4

MSFMWIRALSSCGVEV-LQSPEEETAQLKNATSIFEFSAVDIDKKTVSLDKYRGHVTLIVNVASQXGLAEKNYTQLVQLHSQYAHRGLRILAFPCNQF-KQEQGSEAEIKEFA------RRFGVEFDMFSKIDVNGPDAHPLYKFLKSRLKGS-------------------LGNFIKWNYAKFLCDANGKPFRRYSPTTQPLDIVPDMEALW

>e.muelleri_gpx4a

----------------------------------------------------RGHVTLVVNVASEXGLTDKNYTQLVQLYTEHAGHGLRILAFPCNQFGKQ------------------------FDMFSKIDVNGANAHPLYKYLKSHISGS-------------------LGSFIKWNFEK------------------------------

>Xestospongia.bergquistia_107_GPx_OHH

MSFTWIRSLGSCGVEI-LASTDEEDDLVRNATSIFDFEALDIEGKTVSLDKYR--------------------------------NGFRILAFPCNQFGKQEPGTLANVIEYA------AKHRAEFDIFSKIEVNGPNAHPLFKYLKSKLKGN-------------------LGNFIKWNYAKFLCDSNGVPVKRYSPTTQPLDIVPDMESLW

>e.muelleri_gpx4b

----------------------------------------------------------------------------------------------------QEPGTEAEIKAFA------ASYGVQFDMFSKIDVNGANAHPLYKYLKSHISGS-------------------LGSFIKWNFEK--VRKEGEA---------------------

>Tethya.wilhelma_105_GPx_OHH

MSLSWLHALEAIGVEV-LPAAEEVEDLVKNAKSIYEFEAKDIDGNATSMEIYR-----------------------------------------------YEPGTEEEIKEFV------SQFGVEFDMFSKVDVNGSSAHPLYKYLKSRVRSN-------------------LGSFIKWNFEKFLCDSEGKPVKRYLPTTQPLDIVPDIKTLW

>n.vectensis_gpx4c

MALSWVKAFAGSGANT-SELKEDIEEQVAEAKSIFEFKAKDIDGALVELSKYMGLVTLIVNVASFXGLTQKNYSQLVELHSQYAEKGLRILAFPCNQFGKQEPDPEPVIKQFA------AGYGVKFDMFAKVNVNGSSALPLYKYLKNELKGT-------------------LGSFIKWNFAKFLCNKDGKPIKRYAPTTAPLDIAKDIEELL

>a.planci_gpx4

MSM-RALPSVVFGFTR-MFSCTPRPAAADGPTDIYGFTVKDIDKNEVSLSKYKGHVVLIVNVASQXGLTNKNYAQLQELHATYAEKGLRILAFPCNQFGGQEPGTNQEIKEFA------AKKGAQFDLFDKIDVNGNNASPLYKFLKKKQHGT-------------------LTNAIKWNFSKFLINKKGVPVKRFGPQTNPKDIEKDIVKEL

>n.vectensis_gpx4a

M-------------------------AAGGKQTILDFEVKDIDGNDVALSKYKGFVTLIVNVASQXGFTKKNYTQLQELHSRYAENGLRILAFPCNQFGKQEPGTAEEIKEFV------KQYNVEFDMFAKIEVNGKGADPLYVFLKEAKHGT-------------------LTNAIKWNFTKFLCDKEGVPVKRYSPNTAPLDIEKDIKEQL

>n.vectensis_gpx4b

M----LNFSGLLGFGS-TALFGTRVMAAGGKQTILDFEVKDIDGNDVALSKYKGFVTLIVNVASQXGFTKKNYTQLQELHSRYAENGLRILAFPCNQFGKQEPGTAEEIKEFV------KQYNVEFDMFAKIEVNGKGADPLYVFLKEAKHGT-------------------LTNAIKWNFTKFLCDKEGVPVKRYSPNTAPLDIEKDIKEQL

>l.anatina_gpx4a

MPGQEIFTLVIVGIKD-SQADAKEKDWWKKAGSIYEFTVKDIDGNDVSLEKYKDHVVLIVNVASQXGFTAKNYTQLQSLHAKYAEKGLRILGFPCNQFGGQEPGTEAEIKKFV------EKFDVQFDMFSKIKVNGGDADPLWKYLKHKQGGT-------------------LIDAIKWNFTKFLVDKKGQPVKRYAPNTEPFTVEKDFDEYF

>l.anatina_gpx4b

M---------------------------------------------TATPETRDHVVLIVNVASQXGFTAKNYTQLQSLHAKYAEKGLRILGFPCNQFGGQEPGTEAEIKKFV------EKFDVQFDMFSKIKVNGGDADPLWKYLKHKQGGT-------------------LIDAIKWNFTKFLVDKKGQPVKRYAPNTEPFTVEKDFDEYF

>b.floridae_gpx4a

MGGKRSLVQASRGVTN----MATGGEEWKNATSIYEFSAKDIDGNEVSLEKYRDHVCLIVNVACKXGGTNVNYTQLQELHDKYAEKGLCILAFPCNQFGGQEPWPEPEIKKWVT-----DKFGVKFDMFSKINVNGKDAHPLWKYLKSKQGGT-------------------LIDAIKWNFSKFLINKEGQPVKRYGPNVKPLEIEKDFEPYW

>X.tropicalis_GPX4

MLNRSVLLGSVIGGVQ-SRAMCAQVADWKAAKTIYEFSAVDIDGNEVSLEKYRGYVCIIVNVASKXGKTPVNYTQLVELHAKYAEKGLRILGFPCNQFGKQEPGDEAQIKDFA------ASYKVEFDMFSKIEVNGDGAHPLWKWMKEQPKGT-------------------LGNAIKWNFTKFLINREGAVVKRFSPMEDPVVIEKDLPNLL

>D.rerio_GPX4b

M--RALLVGAVGSKSF-ARAMCAQANDWQSAKSIYEFSAIDIDGNDVSLEKYRGYVCIITNVASKXGKTPVNYTQLAAMHVTYAEKGLRILGFPCNQFGKQEPGSEAEIKEFA------KGYNAEFDLFSKIDVNGDAAHPLWKWMKEQPKGT-------------------LGNNIKWNFTKFLIDREGQVVKRYGPMDDPSVVEKDLPKYL

>D.rerio_GPX4a

MR---FLGSAVVFSLV-LQTMSAQLEDWQTAKSIYEFTATDIDGNEVSLEKYRGKVVIITNVASKXGKTPVNYSQFAEMHAKYSERGLRILAFPSNQFGRQEPGTNSQIKEFA------KSYNAEFDMFSKIDVNGDGAHPLWKWLKDQPNGF-------------------LGNGIKWNFTKFLINREGQIVKRYSPLQDPSVVEKDLSKYL

>D.rerio_GPX4c

MQKRFLLFGALSSSGI-IGATSAQLEDWQTAKSIYEFTATDIDGNEVSLEKYRGKVVIITNVASKXGKTPVNYSQFAEMHAKYSERGLRILAFPSNQFGRQEPGTNSQIKEFA------KSYNAEFDMFSKIDVNGDGAHPLWKWLKDQPNGF-------------------LGNGIKWNFTKFLINREGQVVKRYSPLQDPSVVEKDLSKYL

>H.sapiens_GPX4

MSLPALLCGALAAPGL-AGTMCASRDDWRCARSMHEFSAKDIDGHMVNLDKYRGFVCIVTNVASQXGKTEVNYTQLVDLHARYAECGLRILAFPCNQFGKQEPGSNEEIKEFA------AGYNVKFDMFSKICVNGDDAHPLWKWMKIQPKGI-------------------LGNAIKWNFTKFLIDKNGCVVKRYGPMEEPLVIEKDLPHYF

>Ciona.intestinalis_22_GPx_OHH

MAG-------------------------KDVKDIYGFTVNDIDDQEVSLSKYKGHVCIIVNVASEF-----NYEQLQQLYGKYSQQGLKILAFPCNQFGKQEPKPNADIKKFAT-----ENYGVTFDLFSKINVNGDNAIPLYKFLKTHKNGT-------------------LVNAIKWNFTKFLVTKQGIPYKRFAPNAKPLDMVKDIEELL

>m.leidyi_gpx4b

MQ--------------------------DQYSSVFDFEAVDIHEQNQQLSKYKGNVTLVVNVASYXGLTPLNYEQLQQINEKYYDQGLRILGFPCNQFGNQEPGTDEDIIEFI------KKYNVTFDMFHKINVNGANAIPLYKWLKEKLPGT-------------------ITNAIKWNFTKFLSDRNGVPYKRYAPNFAPNDIIPDIEKLL

>c.elegans_gpx4a

M------------------------------SSVYDFNVKNANGDDVSLSDYKGKVLIIVNVASQCGLTNKNYTQLKELLDVYKKDGLEVLAFPCNQFAGQEPSCEIDIQAFVA-----DKFKFEPTLFQKIDVNGDKQSPLFKFLKNEKGGF-------------------MFDAIKWNFTKFLVGRDGKIIKRFGPTTDPKDMEKDIKEAL

>c.elegans_gpx4b

M------------------------------ASVHGITVKNAQGEDTPLSNYQGKVLIIVNVASQCGLTNSNYNQFKELLDVYKKDGLEVLAFPCNQFGGQEPSCEIDIAAFVA-----DKFKFEPTLFQKIDVNGDNTAPLYKFLKQEKGGF-------------------LVDAIKWNFTKFLVGRDGHVIKRFSPTTEPKDMKKDIEAAL

>c.elegans_gpx4d

MY---------------ISLLNQRLKIDMSTGTIYDFSVRDNSGDLVSLDKYSGLVVIIVNVASYCGLTNSNYKELKSLNDKYHLRGLRVAAFPCNQFGFQEPHCEADINKFVN-----EKFSFEPDLYGKVTVNGGEEEPLWTFLKKEQGGT-------------------LFDAIKWNFTKFLVNRQGKVVARFGPSTNPKSFEEEIVKLL

>d.melanogaster_gpx4a

M-------------------------SYKNAASIYEFTVKDTHGNDVSLEKYKGKVVLVVNIASKCGLTKNNYEKLTDLKEKYGERGLVILNFPCNQFGSQMPEADGEMVCHLR-----DSKADIGEVFAKVDVNGDNAAPLYKYLKAKQTGT-------------------LGSGIKWNFTKFLVNKEGVPINRYAPTTDPMDIAKDIEKLL

>Algoriphagus.machipongonensis_1_GPx_OHH

M-------------------------------TFYDFEAQKLNGKCVSMEEYRGKTVVVVNTASKCGLTP-QYEGLENLYQKHKDEGLVILGFPCNQFANQESGSSEEIEEFCQ-----INYGVSFPMFEKVEVNGKNAHPIFKYLKSKLSGL-------------------LGSKIKWNFTKFVLDKEGNPVKRFAPTTKPEKMEKIILETL

>b.floridae_gpx4b

MTPDMDIVYKLTGLPD-KYWTSVDPNKWRKTRFIYEFEAKDIDGNMISFEKYRGQPLLIVNVASRCGGTDRNYKQLMDLYRKYGEKGLRILAFPCNQFHNQEPYIERDIKEFVT-----TRYGVSFDMFSKIHVLGPETHPIYNWLVNTTRGT-------------------LGDIIKWNFTKFIVDKKGRAVNRYGPNVDPEKIDPDIPKYL

>Podospora.anserina_GPx_OHH

MTS---------------------------------SPWTACKGQEYPLSNLKDKVILIVNVASKCGFTS-QYAGLQELYKNITADQFVILGFPCNQFGGQEPDAEAEIVTFCE-----RNFGVTFPIMQKIEVNGDNAHPLFEWLKEQKSGL-------------------LGKRIKWNFEKFLIGKDGQVKGRWASVTGPASLEKEILAEL

>c.elegans_gpx4c

MNS-------LISILFFSELKCDDTDENDQHGTIYQFQAKNIDGKMVSMEKYRDKVVLFTNVASYCGYTDSNYNAFKELDGIYREKGFRVAAFPCNQFEKQEPETEGKILDFVK-----SSYTYAPDMYSKIEVNGQNTHPLWKFLKKERGSS-------------------LSADIPWNFSKFLVDKNGHVVGRYSHSVNPIDLEEEISRLL

>d.melanogaster_gpx4b

MFE-FLGLLVAVALVT-RSRLQQDLQDMRWRLTIHALTVRDTFGNPVQLDTFAGHVLLIVNIASKCGLTLSQYNGLRYLLEEYEDQGLRILNFPCNQFGGQMPESDQEMLDHLR-----REGANIGHLFAKIDVKGAQADPLYKLLTRHQ------------------------HDIEWNFVKFLVDRKGNIHKRYGAELEPVALTDDIELLL

>Algoriphagus.machipongonensis_2_GPx_OHH

MKN-LITMLFSCQRTI-VQAGLITLPSFEKAPSFYDFKLKDLDGNEVDFSEYKGKKILVVNVASKCGYTP-QYEALQELNEKYGEK-VQVLGFPANNFGGQEPGSNEEIKSFCS-----ENYGVTFPVFEKISVKGFDKHPLYRWLTDKDQGW-------------------NNQEPTWNFCKYLINEKGELVKFYPSDVTPMS--EEILTAI

>a.queenslandica_gpx7

MEQ--------VFFLS-LFCLICGALASSGTETFYSLSAKDITGKMIGFERYSGKIVLVVNVASECGYTDYNYIQLNQLQLRYGEESLAILAFPCNQFGAQEPAKDSEINSMIR-----YKYKPQFPLFSKVNVTGESQSEVYRFLINST-----------------------GLEPKWNFCKYLLDRDGLVVQFFDQGKTFDKIYDSIDGLL

>a.planci_gpx7

MA------LRFLCTIF-VVIASTFPAPVSAAYDFYSFSADDPRGVEVSLNVFRGKPALVVNVASECGYTEGHYEDLVWLSKQPEMERLHILAFPCNQFGQQEPASNQEILDFVQ-----KTYGVTFPVFGKIDVIGKYAHPAYIYLSTDA-----------------------GKQPTWNFWKYLVDRQGRVIDAWEPTTSVREIYDFLVAAT

>s.purpuratus_gpx7

MGL-----LFLVNIFM-TWHVDYCHGRREWHDNFYDFTVKNIEGKRVRLEKYRGTPSLVVNVASDCGYTDRTYRDLVDLSKDPQFDRLNILAFPCNQFGHQEPQSNEEIKHYVK-----ALYDVEFPLFAKVEVKGYDADPAWQYLTSNA-----------------------HQEPTWNFWKFLVDSEGKVKDAWGPDVPIHSIYGELLTEA

>l.anatina_gpx7

MSP--------------IVYEDVTPTTDEPEKDFYMFSVTDIKGKTVSLEEYRGMVTLVVNVASECGYTDSHYKALVKLQNTLAPGKFTVLAFPCNQFGAQEPKDEPSIEKFAK-----EKYGVNFPMFSKINVVEKDIPEAWKFLEDFS-----------------------RLVPNWNFWKYLINPSGHVIATWGPWIPVEDVIEQITEAV

>b.floridae_gpx7

MAA-L------------LALLLSLASSAISQDDFYSFTAKDIKGKTVLLDKYRGKVSLVVNVASECGYTDGHYRELVRLQDHLAPKHFNVLAFPCNQFGGQEPMGNSAIAQFAK-----SMYKANFPMFSKIDVVGREAHPAYKYLAEST-----------------------QAPPTWNFWKYLVDPNGKVITAWPPHNAVVDIWSKVESAV

>X.tropicalis_GPX7

M-----------YLTA-LVLLLLLPPSLQKSRDFYTFKVVNIRGKLVSLEKYRGSVTLVVNVASECGFTDSHYKALQQLQRDLGSYHFNVLAFPCNQFGQQEPNSDREIENFVR-----KNYSASFPMFSKTAVTGTGVNSAFKYLIESS-----------------------GKEPDWNFWKYLVGPDGKVVDAWGPSVSVAEVRPHITSLV

>H.sapiens_GPX7

MVA--------ATVAA-AWLLLWAAACAQQEQDFYDFKAVNIRGKLVSLEKYRGSVSLVVNVASECGFTDQHYRALQQLQRDLGPHHFNVLAFPCNQFGQQEPDSNKEIESFAR-----RTYSVSFPMFSKIAVTGTGAHPAFKYLAQTS-----------------------GKEPTWNFWKYLVAPDGKVVGAWDPTVSVEEVRPQITALV

>D.rerio_GPX7

MG---------MFLRA-FTLIILLCLLEAKQKDFYTFKVVNSRGRLVSLEKYRGSVSLAVNVASECGYTDEHYKDLQQLQKDFGPFHFNVLAFPCNQFGQQEPGSDKEIDSFVR-----RVYGVSFPIFSKIAVVGIGANNAYKYLVEAS-----------------------RKEPTWNFWKYLIDTDGKVVDAWGPEVSVKEIRPRITEMV

>X.tropicalis_GPX8

LSP-FLVFLSMVLCTG-LVCVLQLKFLRAKGGDFYSYEVTDAKGRTVALSKYRGKASLVVNVASSCPHSETNYRSLQELHREFGPYHFTVLAFPCNQFGESEPGSNREIEALAK-----RNYGVTFPMFSKIKILGPEAEPAYKFLVDST-----------------------KTKPRWNFWKYLVNPEGQVVKYWRPDETAEIIRPEVASLV

>H.sapiens_GPX8

LAA-FAVLLSIVLCTV-TLFLLQLKFLKPKINSFYAFEVKDAKGRTVSLEKYKGKVSLVVNVASDCQLTDRNYLGLKELHKEFGPSHFSVLAFPCNQFGESEPRPSKEVESFAR-----KNYGVTFPIFHKIKILGSEGEPAFRFLVDSS-----------------------KKEPRWNFWKYLVNPEGQVVKFWKPEEPIEVIRPDIAALV

>D.rerio_GPX8

LGG-FKVLLSVALCMGSLYLLQNKLSKSRKTKDFYSYEVKDARGRTVSLEKYRGKVSLVVNVASGSELTEQSYRALQELHRELGTSHFNVLAFPCSQYGDTESGTSREIEAFAK-----SNYGVTFPIFNKIKIMGSEAEPAFRFLTDSV-----------------------QKIPRWNFWKFLVSPEGQVVRFWKPEEPVSDIRKEATTLV

>Ciona.intestinalis_21_GPx_OHH

MAV--------LRVLV-LLAPLFGVFSENKHNGFYDYNVKTFDGETVSLKKYIGKVSLVVNVASECGYTDEHYKELTALQNELVQQPFTVLAFPCNQFGEQEPHDNHYIQEFAS-----SEYKASFPIFAKIDVRDRDAHPAYEFLRRST-----------------------GQEPQWNFWKYLLDGSGNVINEWGPSISVSQVKDEILKAI

>Oscarella.carmela_80_GPx_OHH

MII-F------------TRCFIYLIILVSVAGNFYKFSAKDIDGRMIQLSDFAGKVSLVVNVASECGYTESNYEGLVDMQLRYERHDFTVLAFPCNQFGQQEPGSDSNIKTFVN-----RIYDVNFPLFSKIDVTGPNAHPIYQHLAKET-----------------------GEVPTWNFAKYLINRSGKVVKFFDTRMDLKKVEAHIGHLL

>Tethya.wilhelma_106_GPx_OHH

---------------------------------------------------------MIVNVASNCGYTYSHYEGLVLLQEDYKSNSFNVLAFPCDQFGGQEPGSDADIEKFTR-----EKFGINFPLFSKVSVENTNATLLYEYLYETT-----------------------GSKPLWNFCKYLVDLNGEVVQYFSERDSFSVIRHSVEYVV

>Capitella.teleta_7_GPx_OHH

MAK-------CWFVAV-LSATLALVASVPGAEDLFSTPIVDANGRRLTLEEYRGRVLLIVNVASECGFTDGHYKSLVKLQMMFSR--LQVLAFPCNQFGAQEPQDAASVQRWAK-----ATYDVNFPIFGKVNVTGENASPLFRFLISAT-----------------------AKEPTWNFWKYLVNHEGRVLHAWGPWQDVEVVFPEIKAAV

>n.vectensis_gpx7

ME---------------LLGIATLLLQPCFCSQFYSFTAKDIHGQDVSMEKYRGKVVLIVNVASECGFTDVNYRELVALHNKYSKEGLAILAFPCNQFGKQEPKRNYGIYRFAV-----DYYGVQFDMFSKIKTVGDGSHPLYNFLVEST-----------------------GFPPIWNFNKYLVNRAGVVVKYFNHSFNPSSFESIILRHL

>Sycon.ciliatum_101_GPx_OHH

MAM-VAGLLQLLLFWS-MGAERTMAVTHNVPETFYAHTAIDIHGQDIEMGQYRGKVVLIINVASECGYTDSNYKWLSKLSKRYSKHGLAILLFPCNQFGRQEPGSNPEIANFIG-----RQDVNEAKMFEKVDVLGEDAHPLFSFLEHRT-----------------------GHSAKWNFAKYLVDGSGDYVRFYSTTEQLETVEDMIRQLL

>Monosiga.brevicollis_2_GPx_OHH

MAS-----------------TASTNPDPKEAKSIFEFAAEDINGKPFSFQTLEGKVVMIVNTAGQCGLAQRNFTEMVELHDKYKDQGFEIVAFPSNSF-------------------------------NQIAVNGSDTHPLYTYLKGASPGW-------------------LTNSLKWNFTKFLCDRTGVPRKRFGPQEAPSTMATDIE---

>a.planci_gpx1

MASALLVVLWTVAALA-ATGPLESVCVREGSASVHLFSLGSLNSPPVPLSRYAGKVLLLVNVATYXGFTTLQYHQLNALAERYEGM-LEILALPCNQFGLQEPGENDEILNGVKYVRPGGGFEPAFPVFAKIDVNGKKEHELYTHLKSVCPPVKLEI----GDKSKLYWSDIKIGDITWNFEKFLVGGDGQAYKRYDPSIHPKGIEADIEGLI

>a.planci_gpx2

MDG--LALALTLLALP-GGSKAFLDSMCNSTQPIYDISVTALNGSSYKLSQYKGKVLVIVNVASFXGLATPQYPALNALRKAYEGK-IEILAFPCNQFLLQEPEANGEILNLIKYVRPGGGFAPTFPMHQKIEVNGKNTHPLYKALKSVCPAVKEEI----GDPSNFYWSPISNNDITWNFQKFLIDSNGIPYKRYDPLVGPSMLKTDIDLLL

>s.purpuratus_gpx1

MAI-----ILAFFALV-SGATMLDAVCYDDAESLSDMT------KSLSLDDYRGKVVLVVNTASFCTYTY-QYPYFNELKNEFGDQ-LAILGFPCNQFWLQEPGVGQEIPNTLRYVRPGGGYEPNFYLNEKIDVNGPKAHPLFKKLKNSCPPVKMEI----GDPSNLYWSPMTIGDVTWNFNKFLLDKEGVPFKRYDSVVEPLQLVSDIQLVV

>c.elegans_gpx1

MAL-----AVALATII-VDETMRWKECLNTNQSIFDFQIETLQGEYTDLSQYRGKVILLVNVATFCAYTQ-QYTDFNPMLEKYQAQGLTLVAFPCNQFYLQEPAENHELMNGLTYVRPGNGWTPELHIYGKIDVNGDNHHPLYEFVKESCPQTVDKI----GKTDELMYNPVRPSDITWNFEKFLIDRNGQPRFRFHPTAWSHGVTPFIEQLL

>c.elegans_gpx2

MAF-----AALFAFVA-VDETTRWSQCKDTNQSIYDFQVETLQGEYTDLSQYRGQVLLMVNVATFCAYTQ-QYTDFNPLIEKYQSQGFTLIAFPCNQFYLQEPAENHELMNGIMYVRPGNGWKPNLHIYGKLDTNGDNQHPIYEFVKESCPQTVDKI----GKTDELMYNPIRASDITWNFEKFLIDRNGQPRFRFHPTAWSHGVTPFIEQLL

>c.elegans_gpx3

MAL-----AVSFTVIL-VDDTLRWKQCAVTNQSVFDFQIETLKGDYTDLSQYRGKVTLLVNVATFCAYTQ-QYTDFNPILDKYQKQGLVIAAFPCNQFYLQEPAENHELLNGLTHVRPGNG--------------------------------------------------------------------------------------------

>Ciona.intestinalis_23_GPx_OHH

------------------------------QYSVYSNQVFNLHKQNVNLSRFHNEVTLLINVATYLNYSY--------LHQHFNGRNFSVLAFPCDQFHLEEPGEDSEILNGLMYVRPGNGYVPKLNIFGKIKVNGRHEHTIYKNVKASCPPTTLNL----GSTRNMYWNPVKSTDITWNFNKFLLDKNGVPRYRISSDASPTSLIPYITTML

>Ciona.intestinalis_19_GPx_OHH

---------------------------------------------------------------------------MNALSEEYTQSSFVTLAFPCNQFGLQQPEANDEILNGVMYVRPGHGFVPKIYFFSKTQVNGGSEDPLFTSIKASCPPTTNNI----GITSELYWTPIKANDIYWNWNKFLLDKNGMIRYRFGSAVTATQLKPWIDQLL

>Capitella.teleta_9_GPx_OHH

MGG-IALATALCCLAD-TRRLKFSECLDTRGDNIYGHNATELVGNSIQMS----------------GYTY-QYKQLNALVRSFPH--LKILAFPCHQFGHQEPGNESEILAGLKHVRPGGGFRPRFPLTSKTEVNGENEAPLYSFLKRSCPPTTDVI----GNSSNLYFSPIKVTDVTWNFEKFLVDASGVPRFRFHPSVEPTEIVDFIEGLL

>b.floridae_gpx3

MRG-LVAVLLCCGVIS-IYPKQRADCSTAEHGSFHDHHAMLLDGSRVSLAEYRGTTLLVVVVASFXGFTH-QYPAMNALKEKMVGQGFDILAFPTNQFGLQEPETNGEILNVLKYVRPGGGYVPNFPLFQKGDCNGENEQSLFTYLKSCCPPASDMI---VDDKSSLYWKPLRAGDVRWNFEKFLVDPEGKAVMRFTPPVEPAEMEPVIEEFL

>b.floridae_gpx4

MGG-LLASLLCLGFLP-LANKRTEQCSAAEQGSLHDHHAMLLDGSRVSLAEYRGTTLLVVTVATFXGFTQ-QYVGLNALRNKMVEEGFEILGFPTNQFGLEEPARNDEILNGIRYVRPGNDYVPNFTMFQKGDCNGENEQSLFTYLKSCCPPISDVM-GIRGDKDRLYWKPLKVNDVRWNFEKFLVDPEGRGVKRFSSYVTPEDLESVIEEFI

>X.tropicalis_GPX3

MGL-LMLPCFLAALIN-EMDQKSVDCYSSIDGTIYDYGATTLDGTQIPFKAYQGKYILFVNVATYXGLTM-QYQELNALQEELKNNNFVILGFPSNQFGMQEPGRNDEILLGLEYVRPGGKFVPNFQLFEKGDINGRKEQKFYTFLKNSCPPVGDNF----GSATRLMWEPIKVNDVKWNFEKFLVGPDGRPVKRWLPRTPVAQVRREIMSYM

>H.sapiens_GPX3

MAS-CLLSLLLAGFVR-GQEKSKMDCHGGISGTIYEYGALTIDGEEIPFKQYAGKYVLFVNVASYXGLTG-QYIELNALQEELAPFGLVILGFPCNQFGKQEPGENSEILPTLKYVRPGGGFVPNFQLFEKGDVNGEKEQKFYTFLKNSCPPTSELL----GTSDRLFWEPMKVHDIRWNFEKFLVGPDGIPIMRWHHRTTVSNVKMDILSYM

>H.sapiens_GPX6

MFS-CLVLFFLVGFAL-KPQNRKVDCNKGVTGTIYEYGALTLNGEEIQFKQFAGKHVLFVNVAAYXGLAA-QYPELNALQEELKNFGVIVLAFPCNQFGKQEPGTNSEILLGLKYVCPGSGFVPSFQLFEKGDVNGEKEQKVFTFLKNSCPPTSDLL----GSSSQLFWEPMKVHDIRWNFEKFLVGPDGVPVMHWFHQAPVSTVKSDILEYL

>H.sapiens_GPX5

MTV-HLLPLLLACFVP-KQEKMKMDCHKDEKGTIYDYEAIALNKNEVSFKQYVGKHILFVNVATYCGLTA-QYPELNALQEELKPYGLVVLGFPCNQFGKQEPGDNKEILPGLKYVRPGGGFVPSFQLFEKGDVNGEKEQKVFSFLKHSCPHPSEIL----GTFKSISWDPVKVHDIRWNFEKFLVGPDGIPVMRWSHRATVSSVKTDILAYL

>D.rerio_GPX3

MGV-----VLLLALMK-AALSNTQACNSAAGDSFHNYGAKTINGTQIPFSHYAGKHVLVVNVATYXGLTF-QYVELNALHEELRHLGFTILGFPCDQFGKQEPGENNEILSALKYVRPGNGFVPNFQLFEKGDVNGDGEQALFTFLKNACPPVGESF----GATSRLFWEPLKVNDIKWNFEKFLLDPDGRPVMRWFPRVNVSEVRADILKYF

>l.anatina_gpx1

MET-----LLLLAAV--WAVSPPKLCRRKDDKTVYAFKQNSLLGEEISLSRYRGNVLLIVNVATYXGLTS-QYHGLNALQTKYSPLGLTVLAFPCNQFGMQEPGRNTEIWNGIKEVRPGHGFTPNFQMFTKTEVNGINENPLYTYLKTRCDSTVEDF----ATDDKLFYQPKRSQDIRWNFEKFLVDHRGFPLKRFHPRTNPDNFISTLEEAL

>l.anatina_gpx2

M-----------------------ARSPVKIRNFFDFSAKLLSGEDINMSRYKGKVILVENVASLXGTTVRDFTQMNELNSKFKGQ-LVVLGFPCNQFGHQENASGQEILNSLKHVRPGNGFEPNFPIFDKVLVNGQDAHPIFQFLRESLTYPSDEPTEFISNAKLIIWEPVTRSDIAWNFEKFLIGPDGKPYKRYSRKFQTCNIGYDIAELI

>b.floridae_gpx1

------------------------MAAATAVKSFFELSAKALSGEMVSFSRYQGKVVLVENVASLXGTTVRDFTQLNELAAMFGDK-LAILGFPCNQFGHQENATNEEILNSLKYVRPGNGYEPKFDMFSKVQVNGSDAHPVFAYLREKLPIPADNAFLIMNDPKCVIWSPVTRTDIAWNFEKFLIGPDGQPIKRFSRYFQTIDIKNDIEALL

>Capitella.teleta_8_GPx_OHH

---------------------------------------------------------------------------MNQLMEQFGDR-LQILAFPCNQFGHQENTTNDEILKSLKYVRPGNNYTPKFDMFKKVDVNGETAHPVFQFLREQLPTPSDDTVSLMSNPKFLIWSPVCRNDVSWNFEKFLIGPDGEPVKRYSRHFETINIASDIKKLM

>D.rerio_GPX1

--------------------------MAGTMKKFYDLSAKLLSGDLLNFSSLKGKVVLIENVASLXGTTVRDYTQMNELHSRYADQGLVVLGAPCNQFGHQENCKNEEILQSLKYVRPGNGFEPKFQILEKLEVNGENAHPLFAFLKEKLPQPSDDPVSLMGDPKFIIWSPVCRNDISWNFEKFLIGPDGEPFKRYSRRFLTIDIDADIKELL

>H.sapiens_GPX1

M--------------------ARLAAAAAAAQSVYAFSARPLAGEPVSLGSLRGKVLLIENVASLXGTTVRDYTQMNELQRRLGPRGLVVLGFPCNQFGHQENAKNEEILNSLKYVRPGGGFEPNFMLFEKCEVNGAGAHPLFAFLREALPAPSDDATALMTDPKLITWSPVCRNDVAWNFEKFLVGPDGVPLRRYSRRFQTIDIEPDIEALL

>X.tropicalis_GPX2

--------------------------MAYIAKSFYDLYATNIDGEKVDFNVFRGRVVLIENVASLXDTTVRDYTQLNELQTKYPRR-LVVLGFPCNQFGYQENCKNEEILNSLKYVRPGKGFVPGFTLFQKCDVNGKDTHSVFAYLKDKLPVPDNEPAALISDPRYIVWNPVHRSDISWNFEKFLIGPEGEPFKRYNKNFQTISIEPDIQRLL

>D.rerio_GPX2

--------------------------MTFIAKTFYDLHATTLEGDTIDFNIYRGRVVLIENVASLXGTTTQDYTQLNELQSRYPHR-LVVLGFPCNQFGYQENCSDGEILNSLKYVRPGEGYKPSFTIFQKCVVNGSDAHPVFSYLKDKLPYPDDDPVTLIQDPKYLVWNPVSRNDISWNFEKFLIGPEGEPFKRYSKKFQTINIEPDIQRLL

>H.sapiens_GPX2

--------------------------MAFIAKSFYDLSAISLDGEKVDFNTFRGRAVLIENVASLXGTTTRDFTQLNELQCRFPRR-LVVLGFPCNQFGHQENCQNEEILNSLKYVRPGGGYQPTFTLVQKCEVNGQNEHPVFAYLKDKLPYPYDDPFSLMTDPKLIIWSPVRRSDVAWNFEKFLIGPEGEPFRRYSRTFPTINIEPDIKRLL

>X.tropicalis_GPX1

------------------------MRLAMVSRTVYEFSARLLSGENTALSQYKGRVLLIENVASLXGTTIRDYTQMSRLQSMYGPRGLQVLAFPCNQFGHQENSGNQEILNILKHVRPGGGFEPNFPLFEKVDVNGEKEHPLFTFLKGQLPYPSDDSISLMQDPKSIIWSPVRRNDIAWNFEKFLIARNGVPYKRYGRRFETFNIQQDIEKLL

>Oscarella.carmela_82_GPx_OHH

---------------------------------------------------------------------------MNALVDQYGSSGFAVLGFPCNQFGYQENFKEDEILRSLEYIRPGNGYSPKFDLFEKCSVNGEKTHPVFQFLKEKLPYPSDNQLSLMSDPKFIVWSPVTRADLSWNFEKFLIGADGEPFRRYGPSFHTKDLGPDIERLM

>Trichoplax.adhaerens_GPx_OHH

-----------------------------------------------------------MTLSSLEKIDIR----MNQLQAKYAHQGFAVLAFPCNQFGHQENLNGEEILHSLKHVRPGGGYQPDCVVMDKCDVNGSNAHPLFQFLKESLPTPSDDADSLMSDPKFIIWKPVKRSDISWNFEKFLITADGKPYKRYSRNFRTEAIANDIEHLL

>b.floridae_gpx2

MAS-KMDGSWVYANIP-GPGHNTISTSTQHVRNFFDLSGQALSGDIIHFSRYTGKVVLVTNVASACYLTTREFTQLNNLMHLYGLHGLVILAFCCNQFGHSEPFENDEIVKCLRYVRPGPPFQPSFQLFVKCDVNGSRTHRVFDFLKDRLPYPSDDNTMLVAESSEITWNPVKRNDITYNFEKFLIGRDGQPYRRYSYKTPPSRLHQDIKRLL

>Ciona.intestinalis_20_GPx_OHH

MGLALVVALFCPALA--HRAIGERSKCVSSSRTIYDFNFTMLNGTTVPLSKFRGEIHITINATSTHGANW-QYPLFNALQEL---EGVTVLGFPCNQFGLQEPGANSEILKILEHVRPGGGFQPNFPMFEKLEVNGENAHPLFKFLKDQCNVVTSQF----APKARLFYEPIQPNDIEWNFHKFLVDQEGRARRRYHHNTPPDAVRKDIRFLQ

>n.vectensis_gpx1

MKP----LLVMISLYI-KAQDNMSGPCTAIAGSINEFQLEDLDGK---VQDLRDKVVLVVNVASFXSLTKVHYEQLNALKERFKSDGLEIVGFPCNQFKLHEPGDTTEIRNCVKYVRPGGGFEPNFPLMKKTEVNGIKEHPLYTFLKTSCPSPDGVIREDRYKDVRVLWSPIKSDDISWNFEKFLIDHRGKPVRRYKPRLFPERMVQDIDSVI

>Chondrus.crispus_1_GPx_OHH

MSASVTRRTFLAATLS-LPAVLLPELVTAKTSVKSAYSVTSKDGAPLALDKFSGKVTLFVNVATYCALTP-QYEGLVSLFEKFQARGFEIIASPCDQFGHQEPGSNDEICKFAK-----EKFGARFLLLDKLNVNDGGVAPLYQFLRDTSPEN-------------------PGQRVGWNFEKFLVGSDGRVLRRYKPGVLPEQIDEDIGWAL

>Monosiga.brevicollis_1_GPx_OHH

-----------------------------------------------DLLITHWQVLLVVNVASECGYTDPRYEELSHLIRILGKRGIEVLAFPCNQFGGQEPGTAEEVRDFGL-----TNYEADFVYFDKVDVQGVRQAPIYKWLLDQT-----------------------GATIDWNFGMRVTLSPARLSRLYACNTVRFTIQCSTSGTC
